# Ru(II)-Catalyzed Asymmetric Transfer Hydrogenation of α-Alkyl-β-Ketoaldehydes via Dynamic Kinetic Resolution

**DOI:** 10.3390/molecules29143420

**Published:** 2024-07-21

**Authors:** Daiene P. Lapa, Leticia H. S. Araújo, Sávio R. Melo, Paulo R. R. Costa, Guilherme S. Caleffi

**Affiliations:** Laboratório de Química Bioorgânica, Instituto de Pesquisas de Produtos Naturais Walter Mors, Universidade Federal do Rio de Janeiro, Rio de Janeiro 21941-902, Brazil

**Keywords:** 2-alkyl-1-phenylpropane-1,3-diols, ATH-DKR, Noyori–Ikariya catalysts, ruthenium, reduction, asymmetric catalysis, contiguous stereocenters, hydrogen bonding, oxetanes

## Abstract

The (*R*,*R*)-Teth-TsDPEN-Ru(II) complex promoted the one-pot double C=O reduction of α-alkyl-β-ketoaldehydes through asymmetric transfer hydrogenation/dynamic kinetic resolution (ATH-DKR) under mild conditions. In this process, ten *anti*-2-benzyl-1-phenylpropane-1,3-diols (85:15 to 92:8 dr) were obtained in good yields (41–87%) and excellent enantioselectivities (>99% ee for all compounds). Notably, the preferential reduction of the aldehyde moiety led to the in situ formation of 2-benzyl-3-hydroxy-1-phenylpropan-1-one intermediates. These intermediates played a crucial role in enhancing both reactivity and stereoselectivity through hydrogen bonding.

## 1. Introduction

**Figure 1:**
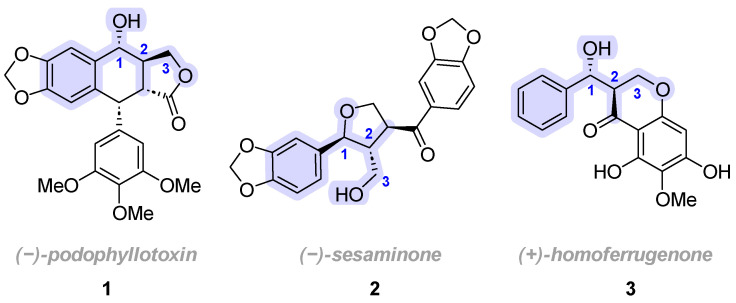
2-alkyl-1-phenylpropane-1,3-diols highlighted in the structure of natural products.

The 2-alkyl-1-phenylpropane-1,3-diols are structural motifs containing two adjacent stereocenters that are prevalent in various natural products. Notable examples include the lignans (−)-podophyllotoxin (**1**) and (−)-sesaminone (**2**), as well as the flavonoid (+)-homoferrugenone (**3**) (Figure 1) [1,2,3].

In the pursuit of synthetic strategies to access these key intermediates in natural products synthesis, transition metal (TM)-catalyzed asymmetric hydrogenation (AH) of α-alkyl-β-ketoesters stands out as a straightforward approach. This method allows for the creation of two contiguous stereocenters in one single step through a dynamic kinetic resolution (DKR) [4,5,6]. However, the α-alkyl-β-ketoesters pose challenges in TM-AH-DKR reactions when compared to cyclic β-ketoesters or acyclic α-heteroatom-substituted β-ketoesters. These challenges arise due to their low racemization rates and poor stereorecognition of the α-alkyl substituents [7,8,9,10].

**Scheme 1:**
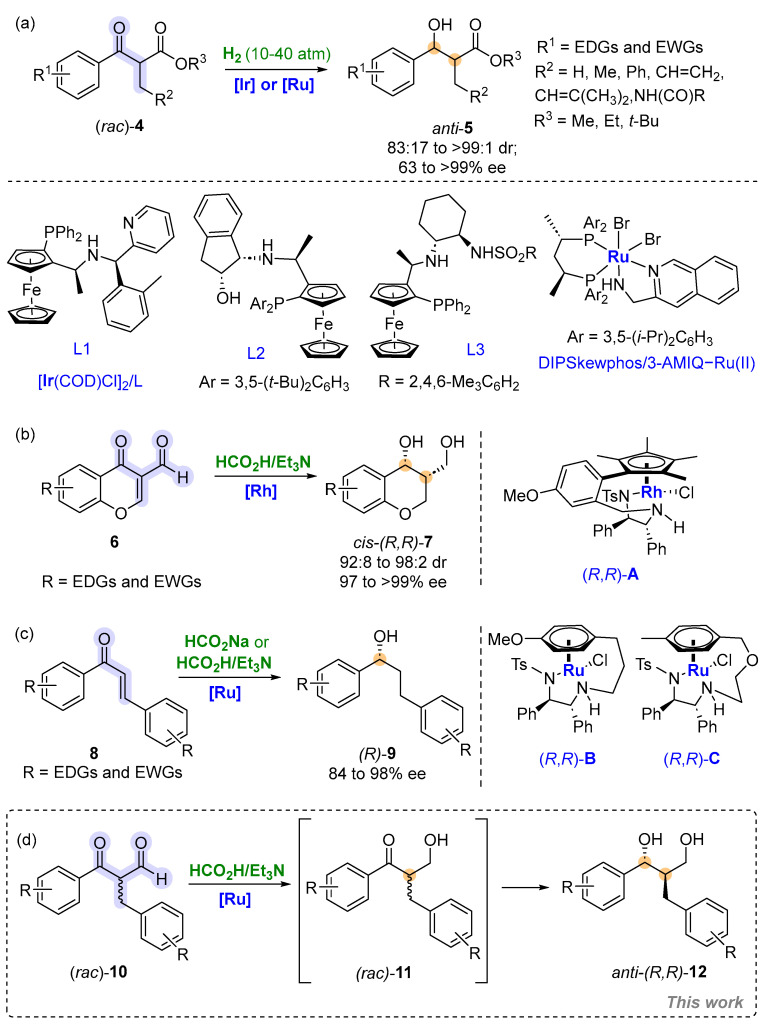
(**a**) Enantioselective synthesis of *anti*-α-alkyl-β-hydroxyesters (**5**) through AH-DKR; (**b**) Enantioselective synthesis of *cis*-3-(Hydroxymethyl)chroman-4-ols (**7**); (**c**) 1,3-diarylpropan-2-ols (**9**); and (**d**) 2-benzyl-1-phenylpropane-1,3-diols (**12**) through ATH-DKR.

To selectively obtain *anti*-α-alkyl-β-hydroxyesters (83:17 to 96:4 dr and 63 to >99% ee), chiral ferrocenyl-based tridentate ligands−Ir(I) complexes were developed. These complexes were employed under 10–40 atm of H_2_ with *t*-BuOK or NaOAc as the base in three seminal works [7,8,9]. More recently, a DIPSkewphos/3-AMIQ−Ru(II) catalyst, in combination with *t*-BuOK, efficiently promoted the AH-DKR of α-alkyl-β-ketoesters under 10 atm of H_2_. This method yielded *anti*-α-alkyl-β-hydroxyesters with high stereoselectivities (94:6 to >99:1 dr and 98 to >99% ee) (Scheme 1a) [10].

Despite the efficiency of the three previous works in synthesizing α-alkyl-β-hydroxyesters with high stereoselectivities, an additional ester reduction step would be required to obtain the 2-alkyl-1-phenylpropane-1,3-diols (Figure 1). Therefore, we wondered if these intermediates could be obtained in a straightforward and practical manner.

In this context, the asymmetric transfer hydrogenation (ATH) reactions emerge as interesting alternatives to the AH methods, particularly in the synthesis of natural products and pharmaceuticals [11,12,13]. Unlike AH, ATH avoids the use of hazardous pressurized H_2_ gas and high-pressure equipment. Instead, formic acid/triethylamine mixtures or sodium formate serve as hydrogen sources [14]. Noyori–Ikariya-type complexes play a pivotal role in ATH, efficiently promoting the reduction of a wide range of ketones, including substrates with labile α- and β-stereocenters. This process results in enantiomerically pure products containing up to three contiguous stereocenters through dynamic kinetic resolution (DKR). Notably, these phosphine-free catalysts are easy to handle, exhibit stability against air and moisture exposure, and remain compatible with both protic and aprotic organic solvents, as well as polar functionalities of the substrate [6,15,16,17]. 

In 2019, Ratovelomanana-Vidal et al. [18] reported the Rh(III)-catalyzed ATH-DKR of 3-formyl-chromones (**6**) using formic acid/triethylamine as hydrogen source (Scheme 1b). The substrate’s three unsaturated bonds were reduced in a one-pot reaction by the Rh(III)-tethered Noyori–Ikariya-type catalyst [19], yielding *cis*-3-(hydroxymethyl)chroman-4-ols (**7**) with high diastereo- and enantioselectivities (92:8 to 98:2 dr and 97 to >99% ee). Remarkably, monitoring studies showed that the aldehyde moiety is the first of the three unsaturated bonds to be reduced. 

In subsequent years, Wills et al. [20] reported the Ru(II)-catalyzed ATH of three chalcones (**8**), leading to the enantioselective synthesis of 1,3-diarylpropan-2-ols (**9**) through a one-pot C=C/C=O reduction sequence (Scheme 1c). Our group expanded the reaction scope to include 27 additional examples using sodium formate as the hydrogen source in water [21]. Furthermore, we demonstrated the applicability of the chiral 1,3-diarylpropan-2-ols (**9**) obtained in the total synthesis of two flavans: the antiviral (*S*)-BW683C and the natural flavan (*S*)-tephrowatsin E. 

Building upon the success of using ATH of chalcones (**8**) as a strategy to obtain 1,3-diarylpropan-2-ols (**9**) [21], we envisioned that acyclic α-benzyl-β-ketoaldehydes (*rac*)-**10** would undergo a one-pot double C=O reduction under ATH-DKR conditions promoted by Noyori–Ikariya-type catalysts. This transformation would afford 2-benzyl-1-phenylpropane-1,3-diols (**12**) with two contiguous stereocenters, exhibiting high diastereo- and enantioselectivities (Scheme 1d). The expected preferential reduction of the aldehyde group [18] would yield 2-benzyl-3-hydroxy-1-phenylpropan-1-one intermediates (**11**), potentially enhancing substrate reactivity and/or stereorecognition of the α-alkyl substituents by the chiral complex through hydrogen bonding. These effects have been increasingly explored in ATH-DKR of ketones bearing hydrogen bonding donors promoted by Noyori–Ikariya-type catalysts [21,22,23,24,25,26,27,28,29]. 

## 2. Results and Discussion

To the best of our knowledge, there are no reports in the literature regarding the synthesis of α-benzyl-β-ketoaldehydes (*rac*)-**10**. Therefore, we developed a synthetic route, as outlined in Table 1. The initial step involves an aldol condensation between commercially available acetophenones and aldehydes, using KOH as the base at room temperature (rt) in a mixture of MeOH and H_2_O [30]. The reactions proceeded for 2 to 27 h, monitored by TLC analysis until complete consumption of the acetophenones. Consequently, chalcones **8a**–**j** were obtained with yields ranging from 65% to 96%. Then, the chalcones **8a**–**j** underwent catalytic hydrogenation using 10% Pd/C in AcOEt at room temperature for 1 to 5 h [31]. The resulting dihydrochalcones **13a**–**j** were obtained in 37 to 96% yield after purification (Table 1). 

Then, the formylation of the dihydrochalcones **13a**–**j** was achieved through reaction with *N*,*N*-dimethylformamide dimethyl acetal (DMF-DMA), followed by hydrolysis of the crude products to yielded α-benzyl-β-ketoaldehydes (*rac*)-**10** (Table 1) [32]. During the optimization process (Table S1), we observed that increasing the solvent polarity—from toluene (PhMe) to dimethyl sulfoxide (DMSO)—resulted in higher conversions of **13** [23]. Additionally, lowering the reaction temperature from 110 to 60 °C was necessary to prevent product degradation. Consequently, reaction times ranging from 42 to 120 h were required to achieve complete conversion of **13**, as confirmed by TLC analysis (Table 1). After work-up, the reaction crudes were directly used in the subsequent step without further purification. 

Notably, initial attempts to promote the hydrolysis of enaminone **14** using 5% HCl aqueous [33] or 25% NaOH [34] resulted in the cleavage of the C=C bond, exclusively affording the dihydrochalcones under acid hydrolysis or in 38% yield under basic conditions (Table S2). Control experiments confirmed that the cleavage occurred at the enaminone **14** intermediate and not at the β-ketoaldehyde **10** (Figures S1 and S2) [33,35]. Subsequently, we discovered that milder acid conditions—specifically, acetic acid at room temperature for 1.5 h—proved optimal. This led to the formation of the tautomeric keto-enol mixtures (**10**/**15**) in yields ranging from 49% to 91% (Table 1).

The α-benzyl-β-ketoaldehyde **10j** was chosen as the substrate for optimizing the ATH-DKR reactions due to its methylenedioxy substituent at ring A. This electron-donating group is commonly found in natural products (Figure 1). The evaluated reaction conditions are listed in Table 2. 

We initiated our evaluations using sodium formate as the hydrogen source in a biphasic DCE/H_2_O mixture. This choice was based on previous reports of faster rates and higher turnover numbers under near-neutral initial conditions in ATH of challenging ketones using Noyori–Ikariya-type catalysts (**A**–**I**) (Table 2, entry 1) [23,36,37,38,39,40]. Additionally, we opted for Will’s 3-C-tethered-Ru(II) catalyst, (*R*,*R*)-**D**, due to its robustness, which often leads to enhanced reactivity and enantioselectivity compared to first-generation catalysts [17,41]. Then, after 16 h at room temperature using 2 mol% of (*R*,*R*)-**D** and 10 mol% of the phase transfer catalyst CTAB, the starting α-benzyl-β-ketoaldehyde **10j** was completely converted into the intermediate ketone **11j**. However, no reduction to the desired product **12j** was observed.

Subsequently, in our quest for higher conversions and stereoselectivities potentially enhanced by the intermediate ketone **11j**, which bears a β-substituted with a hydrogen bond donor hydroxyl group, we evaluated the use of the aprotic DCE as the sole solvent (Table 2, entry 2). Due to solubility issues with sodium formate, we resorted to a formic acid/triethylamine mixture (5:4) as the hydrogen source. Unfortunately, no reduction of the ketone **11j** was observed under these conditions either. 

Further exploration of aprotic solvents revealed that decreasing solvent polarity was beneficial for both enhancing the reduction of intermediate **11j** and improving the stereoselectivity of the reaction (Table 2, entries 3–6). Remarkably, using toluene as the solvent resulted in an **11j**/**12j** ratio of 44:56, with the *anti*-(1*R*,2*R*)-**12j** diol obtained in higher diastero- and enantioselectivity (*anti*/*syn* 92:8, >99% ee) (Table 2, entry 6). The absolute configuration was assigned by comparing optical rotation data from literature (Figure S3) [42].

To enhance the conversion of compound **11j** into *anti*-**12j** diastereoisomer, we raised the reaction temperature from room temperature (rt) to 45 °C. This change led to a substantial conversion of the intermediate ketone **11j** (**11j**/**12j** 7:93) (Table 2, entry 7). However, the higher yield of **12j** at 45 °C was accompanied by a decrease in the diastereoselectivity (*anti*/*syn* 87:13). 

We also evaluated other catalysts, including Ru(II), Rh(III), and Ir(III)-Noyori–Ikariya types, at 45 °C (Table 2, entries 8–13). Unfortunately, these catalysts showed minimal or no conversion of intermediate **11j**. To mitigate the negative impact of higher temperature on stereoselectivity in the of the (*R*,*R*)-**D** catalyzed ATH-DKR, we explored the use of copper(II) triflate or triflic acid as additives (Table 2, entries 14–15) [23,36]. Regrettably, none of these additives significantly improved the previously observed *anti*/*syn* ratio (Table 2, entry 7).

Finally, by increasing the catalyst loading, from 2 to 4 mol% at rt, we achieved a substantial improvement in the conversion of **11j** into **12j**. This yielded the diol **12j** with a good yield (75%) and maintained excellent stereoselectivity (*anti*/*syn* 92:8, >99% ee) (Table 2, entry 16). Notably, extending the reaction time from 16 to 24 h at rt did not further enhance the conversion of the remaining **11j** (Table 2, entry 17). 

**Scheme 2:**
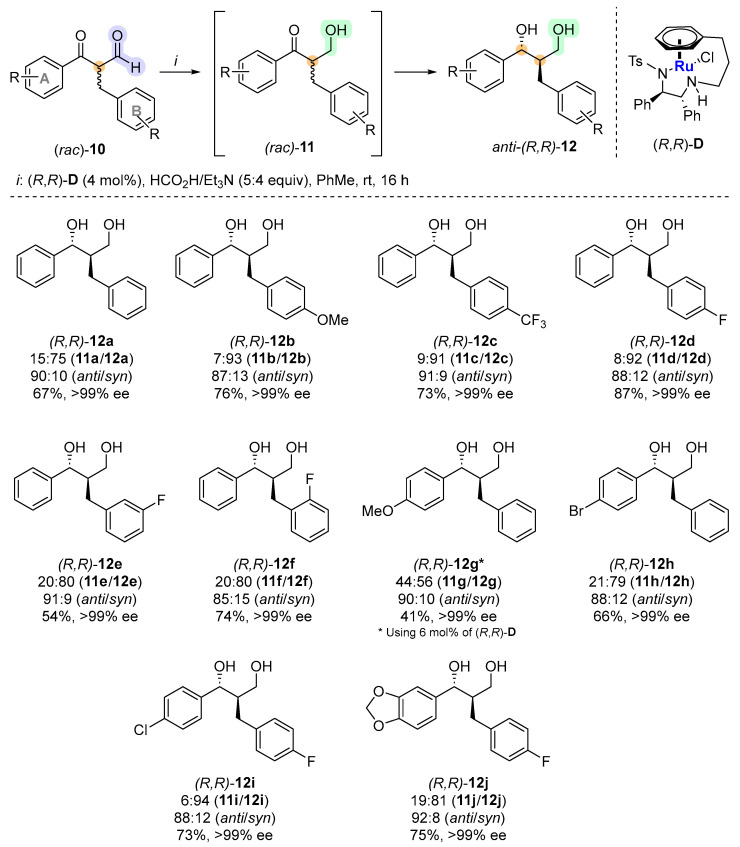
Scope of the one-pot double C=O reduction of α-benzyl-β-ketoaldehydes (*rac*)-**10** under ATH-DKR conditions catalyzed by (*R*,*R*)-**D**.

The scope of the one-pot double C=O reduction of α-benzyl-β-ketoaldehydes (*rac*)-**10**, promoted by the (*R*,*R*)-**D** catalyst, was evaluated using the optimal conditions (Scheme 2). Ten (1*R,*2*R*)-2-benzyl-1-phenylpropane-1,3-diols were obtained in yields ranging from 41% to 87%, demonstrating viability of the dynamic kinetic resolution (DKR) process even under mild reaction conditions for these challenging acyclic α-alkyl-β-ketoaldehydes substrates. Complete conversion of the starting materials **10** was observed in all cases. However, the ratio between the ketone intermediate **11** and the diol **12** was influenced by the substituents.

Generally, substrates with no substituents at the A-ring showed higher conversion of the intermediate ketone **11** compared to substrates substituted with bromine, methoxy, and methylenedioxy groups. The deactivating effect of the electron-donating methoxy group, conjugated at the para-position of the A-ring with the carbonyl group, was particularly remarkable. In this case, an increase in the catalyst loading was necessary to obtain the diol **12g** with good yield (69%). Additionally, fluorine groups at *meta*- and *ortho*-positions of the B-ring resulted in a slight decrease in the ketone **11** reactivity compared to fluorine and other groups at the para-position of the ring. On the other hand, the electron-withdrawing chlorine substituent resulted in the higher conversion of the intermediate ketone **11**. 

The *anti*-diastereoisomers were the major products in all examples, with ratios varying from 85:15 to 92:8 *anti*/*syn*, demonstrating good stereorecognition the α-alkyl substituents by the (*R*,*R*)-**D** catalyst. Notably, excellent enantioselectivities (>99% ee) were found for all examples, regardless of the stereoelectronic effect of the substituent. 

**Scheme 3:**
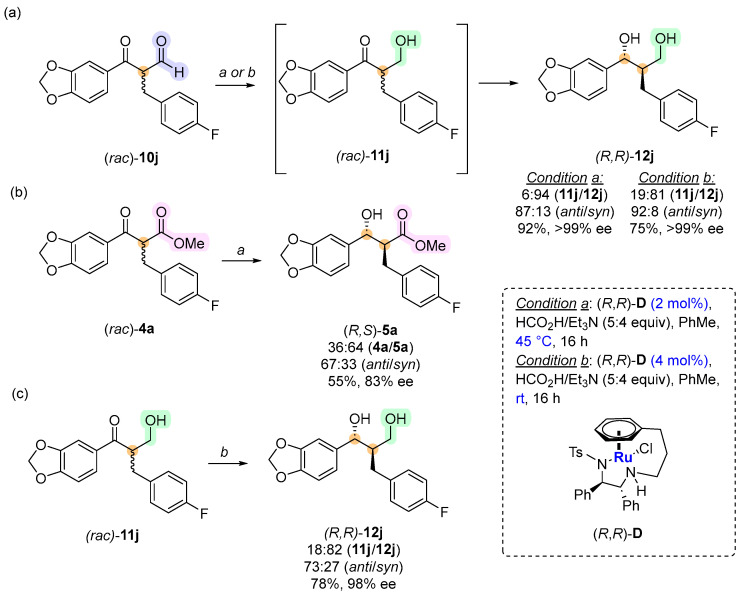
Control experiments using different substrates (**a**) (*rac*)-**10j**, (**b**) (*rac*)-**4a**, and (**c**) (*rac*)-**11j**.

The enhancement of the substrate reactivity and stereorecognition by the chiral complex through in situ formation of the intermediate’s (**11**) hydrogen bonding donor group was supported by control experiments (Scheme 3). 

These experiments were conducted under two conditions previously evaluated for the α-benzyl-β-ketoaldehyde (*rac*)-**10j**, using 2 mol% of (*R*,*R*)-**D** at 45 °C or 4 mol% of (*R*,*R*)-**D** at room temperature (Scheme 3a; Table 2, entries 8 and 16). When the α-benzyl-β-ketoester (*rac*)-**4a** underwent the Ru(II)-catalyzed ATH-DKR at 45 °C, only 64% conversion was observed (Scheme 3b), in sharp contrast to the 94% of conversion of the intermediate (*rac*)-**11j** under the same conditions (Scheme 3a). 

Furthermore, the role of a hydrogen bond donor in these acyclic α-alkyl substrates also proved to be crucial for the catalyst stereorecognition. The ATH-DKR of (*rac*)-**4a** resulted in significantly lower levels of diastereo- and enantioselectivity (67:33 *anti*/*syn*, 83% ee) compared to (*rac*)-**10j** (87:13 *anti*/*syn*, >99% ee). Notably, the in situ formation of the (*rac*)-**11j** played a key role in improving the reaction’s stereoselectivity (92:8 *anti*/*syn*, >99% ee) compared to using (*rac*)-**11j** as the starting material (73:27 *anti*/*syn*, 98% ee) (Scheme 3).

**Scheme 4:**
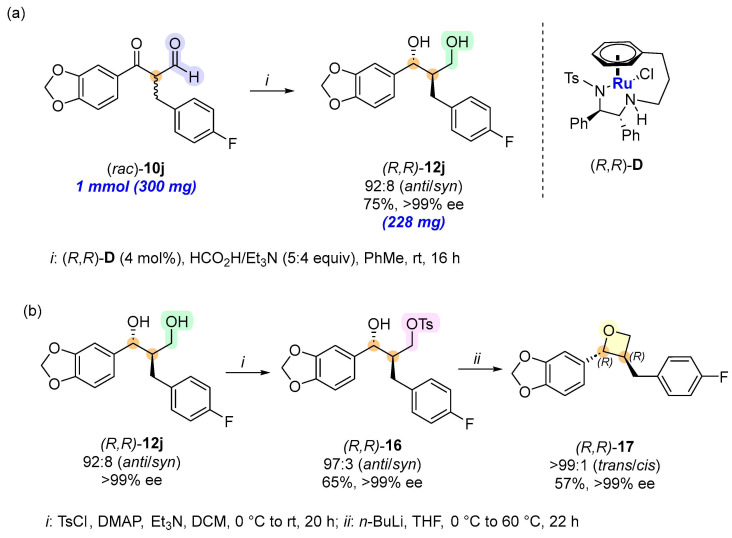
Scale-up (**a**) and synthetic application (**b**) experiments.

To demonstrate the feasibility of the developed Ru(II)-catalyzed ATH-DKR of α-benzyl-β-ketoaldehydes, the reaction was scaled up 10-fold, from 0.1 to 1 mmol (Scheme 4a). After 16 h at rt, the 2-benzyl-1-phenylpropane-1,3-diol **12j** was obtained with the same yield (75%, 228 mg) and stereoselectivity observed previously. 

Subsequently, the utility of the enantiomer-enriched 2-benzyl-1-phenylpropane-1,3-diol **12j** was demonstrated in the synthesis of the oxetane **17** (Scheme 4b). Oxetanes are strained cyclic ethers found in the structures of several natural products. They are considered stable motifs for medicinal chemistry and serve as key reactive intermediates in organic synthesis [43]. The regioselective tosylation of the primary hydroxyl group in **12j** yielded intermediate (*R*,*R*)-**16** in 65% yield, with a slight improvement in the diastereoisomeric ratio (97:3 *anti*/*syn*) and no erosion of the optical purity after purification (>99% ee). Finally, an intramolecular *O*-nucleophilic substitution triggered by *n*-BuLi exclusively afforded *trans*-(2*R*,3*R*)-3-benzyl-2-phenyloxetane **17** in good yield (57%) and >99% ee. 

## 3. Materials and Methods

### 3.1. General Information

All commercial reagents and solvents were purchased from Sigma-Aldrich (St. Louis, MO, USA), Ambeed (Arlington Heights, IL, USA), Biograde (Anápolis, GO, Brazil), Tedia (Fairfield, OH, USA), TCI (Portland, OR, USA), Oakwood Chemical (Estill, SC, USA), or Acros Organics (Geel, Belgium) and used without further purification. All reactions that required heating were performed using an oil bath. Analytical thin layer chromatography (TLC) was performed on 0.25 mm silica gel 60 F254 SiliCycle (Quebec, QC, Canada) plates and visualized under UV light (254 nm or 365 nm) or by staining with vanillin/H_2_SO_4_. Preparative TLC was performed on 20 × 20 cm glass backed plates bearing a 0.5 mm layer of silica gel 60 F254 (Macherey-Nagel; Düren, Germany). Flash column chromatography was performed on silica gel 60 (230–400 mesh) SiliCycle (Quebec, QC, Canada). High-performance liquid chromatography (HPLC) analyses were carried out on a Shimadzu (Kyoto, Japan) LC-20AT liquid chromatograph equipped with an SPD-M20A diode array detector, and retention times (tR) are expressed in minutes. NMR spectra were recorded on Varian Unity (Palo Alto, CA, USA) 400 or 500 MHz instruments at 25 °C. Chemical shifts are expressed in ppm relative to TMS (Me4Si) or deuterated solvent (CDCl_3_, DMSO-*d*_6_, (CD_3_)_2_CO) and the coupling constants are expressed in Hz. High-resolution mass spectra (HRMS) were obtained with a Bruker solariX XR mass spectrometer (Billerica, MA, USA) with Electrospray Ionization (ESI) source coupled to a Fourier Transform–Ion Cyclotron Resonance (FT-ICR) mass analyzer. 

### 3.2. General Procedure for the Synthesis of Compounds ***8a**–**j***

A mixture of the appropriate acetophenone (1 equiv., 6 mmol) and KOH (7.5 equiv., 45 mmol) in MeOH/H_2_O (2:1, 36 mL) was magnetically stirred at rt for 10 min. The resulting solution was treated with the corresponding benzaldehyde (2.0 equiv., 6 mmol) and then stirred 2–27 h at rt, until the total consumption of acetophenone, indicated by TLC analysis. After completing the reaction, it was acidified to pH = 7 under an ice bath. Saturated sodium bisulfite solution (200 mL) was added, and the mixture was stirred for 40 min. After, the mixture was diluted with EtOAc (50 mL), washed with H_2_O (3 × 50 mL) and brine (1 × 50 mL), dried over anhydrous Na_2_SO_4_, filtered, and concentrated under reduced pressure. The residue was purified by flash chromatography on a silica gel (EtOAc/hexane, 5:95), yielding the pure product (see Supplementary Materials for NMR spectra).

(*E*)-chalcone (**8a**): Synthesized in a 6 mmol scale and purified by recrystallization from chloroform/hexane. Pale yellow solid, 1067.6 mg, 85% yield. ^1^H NMR (500 MHz, CDCl_3_) δ 8.02 (d, *J* = 7.1 Hz, 2H), 7.81 (d, *J* = 15.8 Hz, 1H), 7.67–7.62 (m, 2H), 7.58 (t, *J* = 7.4 Hz, 1H), 7.55–7.48 (m, 3H), 7.41 (dd, *J* = 5.1, 1.9 Hz, 3H). ^13^C NMR (126 MHz, CDCl_3_) δ 190.6, 144.8, 138.2, 134.9, 132.8, 130.6, 129.0, 128.6, 128.5, 128.5, 122.1. Spectroscopic data were consistent with those reported in the literature [44].

(*E*)-3-(4-methoxyphenyl)-1-phenylprop-2-en-1-one (**8b**): Synthesized in a 5 mmol scale and purified by recrystallization from chloroform/hexane. Pale yellow solid, 796.4 mg, 67% yield. ^1^H NMR (500 MHz, CDCl_3_) δ 8.01 (d, *J* = 6.9 Hz, 2H), 7.79 (d, *J* = 15.6 Hz, 1H), 7.60 (d, *J* = 8.7 Hz, 2H), 7.56 (d, *J* = 7.5 Hz, 1H), 7.49 (t, *J* = 7.5 Hz, 2H), 7.41 (d, *J* = 15.7 Hz, 1H), 6.94 (d, *J* = 8.9 Hz, 2H), 3.85 (s, 3H). ^13^C NMR (126 MHz, CDCl_3_) δ 190.6, 161.7, 144.7, 138.5, 132.6, 130.3, 128.6, 128.4, 127.6, 119.8, 114.4, 55.4. Spectroscopic data were consistent with those reported in the literature [44].

(*E*)-1-phenyl-3-(4-(trifluoromethyl)phenyl)prop-2-en-1-one (**8c**): Synthesized in a 6 mmol scale and purified by recrystallization from H_2_O/MeOH. White solid, 1017.6 mg, 61% yield. ^1^H NMR (500 MHz, CDCl_3_) δ 8.03 (d, *J* = 7.0 Hz, 2H), 7.80 (d, *J* = 15.9 Hz, 1H), 7.73 (d, *J* = 8.2 Hz, 2H), 7.67 (d, *J* = 8.5 Hz, 2H), 7.60 (d, *J* = 15.9 Hz, 2H), 7.54–7.49 (m, 2H). ^13^C NMR (126 MHz, CDCl_3_) δ 190.0, 142.7, 138.3, 137.8, 131.9 (q, *J* = 32.8 Hz), 131.8, 128.7, 128.6, 128.5, 125.9 (q, *J* = 3.9 Hz), 124.3 (q, *J* = 272.2 Hz), 124.2. Spectroscopic data were consistent with those reported in the literature [44].

(*E*)-3-(4-fluorophenyl)-1-phenylprop-2-en-1-one (**8d**): Synthesized in a 5 mmol scale and purified by column chromatography on silica gel (EtOAc/hexane, 10:90). White solid, 940 mg, 83% yield. ^1^H NMR (500 MHz, CDCl_3_) δ 8.02 (dd, *J* = 8.4, 1.3 Hz, 2H), 7.78 (d, *J* = 15.8 Hz, 1H), 7.64 (dd, *J* = 8.4, 5.3 Hz, 2H), 7.59 (t, *J* = 7.4 Hz, 1H), 7.51 (t, *J* = 7.6 Hz, 2H), 7.46 (d, *J* = 15.8 Hz, 1H), 7.11 (t, *J* = 8.7 Hz, 2H). ^13^C NMR (126 MHz, CDCl_3_) δ 190.3, 164.1 (d, *J* = 251.8 Hz), 143.5, 138.2, 132.8, 131.2 (d, *J* = 3.3 Hz), 130.4 (d, *J* = 8.6 Hz), 128.7, 128.5, 121.8, 121.8, 116.2, 116.1 (d, *J* = 21.9 Hz). Spectroscopic data were consistent with those reported in the literature [45].

(*E*)-3-(3-fluorophenyl)-1-phenylprop-2-en-1-one (**8e**): Synthesized in a 6 mmol scale and the crude material was used in the next step without purification. White solid. ^1^H NMR (500 MHz, CDCl_3_) δ 8.02 (dd, *J* = 8.4, 1.3 Hz, 2H), 7.75 (d, *J* = 15.8 Hz, 1H), 7.59 (t, *J* = 7.4 Hz, 1H), 7.54–7.48 (m, 3H), 7.42–7.31 (m, 3H), 7.13–7.06 (m, 1H). ^13^C NMR (126 MHz, CDCl_3_) δ 190.1, 163.1 (d, *J* = 247.0 Hz), 143.3, 137.9, 137.2 (d, *J* = 7.6 Hz), 133.0, 130.5 (d, *J* = 8.1 Hz), 128.7, 128.5, 124.6, 124.5, 123.2, 117.4 (d, *J* = 21.5 Hz), 114.5 (d, *J* = 21.9 Hz). Spectroscopic data were consistent with those reported in the literature [44].

(*E*)-3-(2-fluorophenyl)-1-phenylprop-2-en-1-one (**8f**): Synthesized in a 6 mmol scale and the crude material was used in the next step without purification. Pale yellow solid. ^1^H NMR (500 MHz, CDCl_3_) δ 8.02 (d, *J* = 7.1 Hz, 2H), 7.90 (d, *J* = 15.9 Hz, 1H), 7.67–7.61 (m, 2H), 7.58 (t, *J* = 7.3 Hz, 1H), 7.50 (t, *J* = 7.6 Hz, 2H), 7.40–7.34 (m, 1H), 7.19 (t, *J* = 7.6 Hz, 1H), 7.15–7.09 (m, 1H). ^13^C NMR (126 MHz, CDCl_3_) δ 190.5, 161.7 (d, *J* = 254.2 Hz), 138.0, 137.5 (d, *J* = 2.4 Hz), 132.9, 131.9 (d, *J* = 8.6 Hz), 129.8 (d, *J* = 2.9 Hz), 128.7, 128.6, 124.6 (d, *J* = 7.6 Hz), 124.5 (d, *J* = 1.9 Hz), 123.0 (d, *J* = 11.4 Hz), 116.3 (d, *J* = 21.9 Hz). Spectroscopic data were consistent with those reported in the literature [46].

(*E*)-1-(4-methoxyphenyl)-3-phenylprop-2-en-1-one (**8g**): Synthesized in a 6 mmol scale and purified by recrystallization from chloroform/hexane. White solid, 1155.4 mg, 81% yield. ^1^H NMR (500 MHz, CDCl_3_) δ 8.04 (d, *J* = 9.0 Hz, 2H), 7.80 (d, *J* = 15.6 Hz, 1H), 7.64 (d, *J* = 9.6 Hz, 2H), 7.54 (d, *J* = 15.7 Hz, 1H), 7.44–7.39 (m, 3H), 6.98 (d, *J* = 9.0 Hz, 2H), 3.88 (s, 3H). ^13^C NMR (126 MHz, CDCl_3_) δ 188.7, 163.4, 144.0, 135.1, 131.1, 130.8, 130.3, 128.9, 128.4, 121.9, 113.9, 55.5. Spectroscopic data were consistent with those reported in the literature [44].

(*E*)-1-(4-bromophenyl)-3-phenylprop-2-en-1-one (**8h**): Synthesized in a 6 mmol scale. White solid, 1722.5 mg, quantitative yield. ^1^H NMR (400 MHz, CDCl_3_) δ 7.88 (d, *J* = 8.6 Hz, 2H), 7.81 (d, *J* = 15.7 Hz, 1H), 7.64 (d, *J* = 8.6 Hz, 4H), 7.47 (d, *J* = 15.7 Hz, 1H), 7.44–7.38 (m, 3H). ^13^C NMR (101 MHz, CDCl_3_) δ 189.4, 145.4, 136.9, 134.7, 131.9, 130.8, 130.0, 129.0, 128.5, 127.9, 121.5. Spectroscopic data were consistent with those reported in the literature [47].

(*E*)-1-(4-chlorophenyl)-3-(4-fluorophenyl)prop-2-en-1-one (**8i**): Synthesized in a 5 mmol scale and purified by recrystallization from chloroform/hexane. White solid, 841.1 mg, 65% yield. ^1^H NMR (400 MHz, CDCl_3_) δ 7.96 (d, *J* = 8.6 Hz, 2H), 7.78 (d, *J* = 15.7 Hz, 1H), 7.64 (dd, *J* = 8.9, 5.3 Hz, 2H), 7.48 (d, *J* = 8.6 Hz, 2H), 7.41 (d, *J* = 15.7 Hz, 1H), 7.12 (t, *J* = 8.6 Hz, 2H). ^13^C NMR (101 MHz, CDCl_3_) δ 189.0, 164.2 (d, *J* = 252.2 Hz), 144.0, 139.3, 136.4, 131.0 (d, *J* = 3.4 Hz), 130.4 (d, *J* = 8.8 Hz), 129.9, 129.0, 121.2, 116.2 (d, *J* = 21.7 Hz). Spectroscopic data were consistent with those reported in the literature [48].

(*E*)-1-(benzo[*d*][1,3]dioxol-5-yl)-3-(4-fluorophenyl)prop-2-en-1-one (**8j**): Synthesized in a 5 mmol scale and purified by recrystallization from chloroform/hexane. White crystalline solid, 1154.3 mg, 85% yield. ^1^H NMR (400 MHz, CDCl_3_) δ 7.75 (d, *J* = 15.7 Hz, 1H), 7.67–7.58 (m, 3H), 7.52 (d, *J* = 1.8 Hz, 1H), 7.41 (d, *J* = 15.6 Hz, 1H), 7.10 (t, *J* = 8.6 Hz, 2H), 6.89 (d, *J* = 8.1 Hz, 1H), 6.06 (s, 2H). ^13^C NMR (101 MHz, CDCl_3_) δ 188.0, 164.0 (d, J = 251.4 Hz), 151.7, 148.3, 142.9, 132.9, 131.2 (d, *J* = 3.4 Hz), 130.2 (d, *J* = 8.8 Hz), 124.6, 121.4, 116.1 (d, *J* = 21.7 Hz), 108.4, 107.9, 101.9. Spectroscopic data were consistent with those reported in the literature [49].

### 3.3. General Procedure for the Synthesis of Compounds ***13a**–**j***

To a solution of the corresponding chalcone **8a**–**j** (1.0 equiv.) in EtOAc (0.083 M) was added 10% Pd/C (2–8% m/m in Pd/chalcone ratio) and stirred under hydrogen atmosphere at rt for 1–4.5 h, until the total consumption of starting material, indicated by TLC analysis. Upon completion, the mixture was diluted with EtOAc (40 mL), vacuum-filtered through a pad of Celite/Silica, and the solvent was removed under reduced pressure. The obtained crude was purified by flash chromatography on a silica gel (EtOAc/hexane, 5:95), to afford the dihydrochalcones **13a**–**j**.

1,3-diphenylpropan-1-one (**13a**): Synthesized in a 4.1 mmol scale and purified by column chromatography on silica gel (EtOAc/hexane, 5:95). White solid, 927.1 mg, 87% yield. ^1^H NMR (400 MHz, CDCl_3_) δ 7.96 (d, *J* = 7.1 Hz, 2H), 7.56 (t, *J* = 7.4 Hz, 1H), 7.45 (t, *J* = 7.6 Hz, 2H), 7.34–7.23 (m, 4H), 7.21 (t, *J* = 7.0 Hz, 1H), 3.31 (t, *J* = 7.5 Hz, 2H), 3.07 (t, *J* = 8.2 Hz, 2H). ^13^C NMR (101 MHz, CDCl_3_) δ 199.2, 141.3, 136.8, 133.1, 128.6, 128.5, 128.4, 128.0, 126.1, 40.4, 30.1. Spectroscopic data were consistent with those reported in the literature [50].

3-(4-methoxyphenyl)-1-phenylpropan-1-one (**13b**): Synthesized in a 3.5 mmol scale and purified by column chromatography on silica gel (EtOAc/hexane, 10:90). Pale beige solid, 443.3 mg, 53% yield. ^1^H NMR (400 MHz, CDCl_3_) δ 7.96 (d, *J* = 7.0 Hz, 2H), 7.56 (t, *J* = 7.4 Hz, 1H), 7.46 (t, *J* = 7.9 Hz, 2H), 7.18 (d, *J* = 8.8 Hz, 2H), 6.85 (d, *J* = 8.8 Hz, 2H), 3.79 (s, 3H), 3.31–3.24 (m, 2H), 3.02 (t, *J* = 7.6 Hz, 2H). ^13^C NMR (101 MHz, CDCl_3_) δ 199.4, 158.0, 136.9, 133.3, 133.0, 129.4, 128.6, 128.0, 113.9, 55.3, 40.7, 29.3. Spectroscopic data were consistent with those reported in the literature [51].

1-phenyl-3-(4-(trifluoromethyl)phenyl)propan-1-one (**13c**): Synthesized in a 3.7 mmol scale and purified by column chromatography on silica gel (EtOAc/hexane, 7:93). White solid, 685.8 mg, 67% yield. ^1^H NMR (400 MHz, CDCl_3_) δ 7.98 (d, *J* = 7.1 Hz, 2H), 7.57 (d, *J* = 7.9 Hz, 3H), 7.49 (t, *J* = 7.5 Hz, 2H), 7.40 (d, *J* = 8.3 Hz, 2H), 3.35 (t, *J* = 7.3 Hz, 2H), 3.16 (t, *J* = 7.5 Hz, 2H). ^13^C NMR (101 MHz, CDCl_3_) δ 198.6, 145.5, 136.7, 133.3, 128.8, 128.7, 128.6 (q, *J* = 24.6 Hz), 128.0, 125.4 (q, *J* = 3.9 Hz), 124.3 (q, *J* = 271.8 Hz), 39.8, 29.8. Spectroscopic data were consistent with those reported in the literature [52].

3-(4-fluorophenyl)-1-phenylpropan-1-one (**13d**): Synthesized in a 4.16 mmol scale and purified by column chromatography on silica gel (EtOAc/hexane, 5:95). White solid, 615.3 mg, 65% yield. ^1^H NMR (400 MHz, CDCl_3_) δ 7.95 (d, *J* = 7.1 Hz, 2H), 7.55 (t, *J* = 7.4 Hz, 1H), 7.45 (t, *J* = 7.5 Hz, 2H), 7.20 (dd, *J* = 8.8, 5.4 Hz, 2H), 6.97 (t, *J* = 8.8 Hz, 2H), 3.27 (t, *J* = 7.4 Hz, 2H), 3.04 (t, *J* = 7.6 Hz, 2H). ^13^C NMR (101 MHz, CDCl_3_) δ 199.0, 161.4 (d, *J* = 243.8 Hz), 136.9 (d, *J* = 3.4 Hz), 136.8, 133.1, 129.8 (d, *J* = 7.6 Hz), 128.6, 128.0, 115.2 (d, *J* = 21.0 Hz), 40.4, 29.2. Spectroscopic data were consistent with those reported in the literature [51].

3-(3-fluorophenyl)-1-phenylpropan-1-one (**13e**): Synthesized in a 6 mmol scale from the crude material of the previous step. Purified by column chromatography on silica gel (EtOAc/hexane, 3:97). White solid, 1077.7 mg, 79% yield (two steps). ^1^H NMR (400 MHz, CDCl_3_) δ 7.95 (dd, *J* = 8.4, 1.3 Hz, 2H), 7.55 (t, *J* = 7.4 Hz, 1H), 7.45 (t, *J* = 7.5 Hz, 2H), 7.26–7.21 (m, 1H), 7.02 (d, *J* = 8.3 Hz, 1H), 6.95 (d, *J* = 9.9 Hz, 1H), 6.89 (t, *J* = 7.9 Hz, 1H), 3.29 (t, *J* = 7.8 Hz, 2H), 3.06 (t, *J* = 7.6 Hz, 2H). ^13^C NMR (101 MHz, CDCl_3_) δ 198.8, 162.9 (d, *J* = 245.8 Hz), 143.9 (d, *J* = 7.3 Hz), 136.8, 133.2, 130.0, 129.9 (d, *J* = 8.4 Hz), 128.7, 128.0, 124.1, 115.3 (d, *J* = 20.9 Hz), 113.0 (d, *J* = 20.9 Hz), 40.0, 29.7. Spectroscopic data were consistent with those reported in the literature [53].

3-(2-fluorophenyl)-1-phenylpropan-1-one (**13f**): Synthesized in a 6 mmol scale from the crude material of the previous step. Purified by column chromatography on silica gel (EtOAc/hexane, 3:97). White solid, 503.8 mg, 37% yield (two steps). ^1^H NMR (500 MHz, CDCl_3_) δ 7.95 (dd, *J* = 8.5, 1.3 Hz, 2H), 7.54 (t, *J* = 7.4 Hz, 1H), 7.47–7.40 (m, 2H), 7.29–7.24 (m, 1H), 7.21–7.15 (m, 1H), 7.06 (t, *J* = 8.1 Hz, 1H), 7.04–6.99 (m, 1H), 3.32–3.27 (m, 2H), 3.09 (t, *J* = 7.7 Hz, 2H). ^13^C NMR (126 MHz, CDCl_3_) δ 199.0, 161.3 (d, *J* = 244.6 Hz), 136.8, 130.9 (d, *J* = 5.2 Hz), 128.6, 128.1 (d, *J* = 15.7 Hz), 128.0 (d, *J* = 8.6 Hz), 124.1 (d, *J* = 3.8 Hz), 115.3 (d, *J* = 21.9 Hz), 38.9, 24.0. Spectroscopic data were consistent with those reported in the literature [51].

1-(4-methoxyphenyl)-3-phenylpropan-1-one (**13g**): Synthesized in a 4.3 mmol scale and purified by column chromatography on silica gel (EtOAc/hexane, 10:90). Pale yellow solid, 1063.1 mg, 92% yield. ^1^H NMR (500 MHz, CDCl_3_) δ 7.93 (d, *J* = 9.0 Hz, 2H), 7.32–7.27 (m, 2H), 7.25 (d, *J* = 6.6 Hz, 2H), 7.22–7.17 (m, 1H), 6.91 (d, *J* = 9.0 Hz, 2H), 3.85 (s, 3H), 3.24 (s, 2H), 3.05 (s, 2H). ^13^C NMR (126 MHz, CDCl_3_) δ 197.8, 163.5, 141.5, 130.3, 130.0, 128.5, 128.4, 126.1, 113.7, 55.5, 40.1, 30.3. Spectroscopic data were consistent with those reported in the literature [50].

1-(4-bromophenyl)-3-phenylpropan-1-one (**13h**): Synthesized in a 4.22 mmol scale and filtrated with vacuum pump. White solid, 1111.5 mg, 91% yield. ^1^H NMR (500 MHz, CDCl_3_) δ 7.81 (d, *J* = 8.7 Hz, 2H), 7.58 (d, *J* = 8.7 Hz, 2H), 7.33–7.17 (m, 5H), 3.26 (t, *J* = 7.2 Hz, 2H), 3.06 (t, *J* = 7.3 Hz, 2H). ^13^C NMR (126 MHz, CDCl_3_) δ 198.2, 141.0, 135.6, 132.0, 129.6, 128.6, 128.4, 128.2, 126.2, 40.4, 30.0. Spectroscopic data were consistent with those reported in the literature [51].

1-(4-chlorophenyl)-3-(4-fluorophenyl)propan-1-one (**13i**): Synthesized in a 3.17 mmol scale and purified by column chromatography on silica gel (EtOAc/hexane, 10:90). White solid, 640.1 mg, 77% yield. ^1^H NMR (400 MHz, CDCl_3_) δ 7.88 (d, *J* = 8.7 Hz, 2H), 7.41 (d, *J* = 8.7 Hz, 2H), 7.19 (dd, *J* = 8.7, 5.4 Hz, 2H), 6.97 (t, *J* = 8.7 Hz, 2H), 3.24 (t, *J* = 7.8 Hz, 2H), 3.03 (t, *J* = 7.5 Hz, 2H). ^13^C NMR (101 MHz, CDCl_3_) δ 197.75, 161.45 (d, *J* = 244.0 Hz), 139.6, 136.7 (d, *J* = 3.2 Hz), 135.1, 129.8 (d, *J* = 7.8 Hz), 129.4, 128.9, 115.3 (d, *J* = 21.2 Hz), 40.4, 29.2. Spectroscopic data were consistent with those reported in the literature [54].

1-(benzo[*d*][1,3]dioxol-5-yl)-3-(4-fluorophenyl)propan-1-one (**13j**): Synthesized in a 4.2 mmol scale and purified by column chromatography on silica gel (EtOAc/hexane, 10:90). White solid, 1145.6 mg, 88% yield. ^1^H NMR (400 MHz, CDCl_3_) δ 7.54 (d, *J* = 8.2 Hz, 1H), 7.43 (s, 1H), 7.23–7.14 (m, 2H), 6.96 (t, *J* = 8.8 Hz, 2H), 6.83 (d, *J* = 8.2 Hz, 1H), 6.03 (s, 2H), 3.19 (t, *J* = 7.3 Hz, 2H), 3.01 (t, *J* = 7.6 Hz, 2H). ^13^C NMR (101 MHz, CDCl_3_) δ 197.1, 161.4 (d, *J* = 243.8 Hz), 151.8, 148.2, 136.9 (d, *J* = 3.1 Hz), 131.7, 129.8 (d, *J* = 8.0 Hz), 124.2, 115.2 (d, *J* = 21.0 Hz), 107.9, 107.8, 101.8, 40.1, 29.5. Spectroscopic data were consistent with those reported in the literature [55].

### 3.4. Method for the Synthesis of Compound ***4a***

To a solution of methyl 3-(benzo[d][1,3]dioxol-5-yl)-3-oxopropanoate (1.0 equiv, 1110 mg, 5 mmol) in dimethylformamide (12.5 mL) was added K_2_CO_3_ (1.0 equiv, 570 mg, 5 mmol) and 1-(bromomethyl)-4-fluorobenzene (1.0 equiv, 0.62 mL, 5 mmol) and was stirred 24 h at 60 °C. Subsequently, the reaction was diluted with EtOAc (75 mL), washed with H_2_O (7 × 75 mL), dried over anhydrous Na_2_SO_4_, filtered, and concentrated under reduced pressure. The obtained crude was purified by flash chromatography on a silica gel (EtOAc/hexane/DCM, 1:6:1), yielding the pure product **4a**

methyl 3-(benzo[*d*][1,3]dioxol-5-yl)-2-(4-fluorobenzyl)-3-oxopropanoate (**4a**): White solid, 907.7 mg, 55% yield. ^1^H NMR (500 MHz, DMSO-*d*_6_) δ 7.62 (dd, *J* = 8.3, 1.8 Hz, 1H), 7.42 (d, *J* = 1.8 Hz, 1H), 7.27 (dd, *J* = 8.8, 5.5 Hz, 2H), 7.08–6.99 (m, 3H), 6.13 (s, 2H), 4.98 (t, *J* = 7.6 Hz, 1H), 3.54 (s, 3H), 3.14 (dd, *J* = 7.6, 2.8 Hz, 2H). ^13^C NMR (126 MHz, DMSO-*d_6_*) δ 193.3, 170.0, 161.4 (d, *J* = 242.2 Hz), 152.6, 148.5, 134.7, 131.4 (d, *J* = 8.1 Hz), 130.9 (d, *J* = 3.3 Hz), 125.9, 115.1 (d, *J* = 21.5 Hz), 108.6, 108.2, 102.7, 54.2, 52.6, 34.0. HRMS (ESI) [M + H]^+^ calc. For C_18_H_16_FO_5_^+^ = 331.0976; found = 331.0972.

### 3.5. General Procedure for the Synthesis of Compounds ***10**/**15a**–**j***

To a solution of the corresponding dihydrochalcones **13a**–**j** (1.0 equiv.) in DMSO (0.196 M) was added N,N-dimethylformamide dimethyl acetal (DMF-DMA, 5.0 equiv.) and stirred at rt for 10 min. Then, the reaction mixture was heated to 60 °C and stirred for 42–120 h, until the total consumption of starting material, indicated by TLC analysis. After this time, the mixture was cooled to rt and a solution acetic acid/H_2_O (1:1, 0.24 M) was added, allowed to stir at rt for 1.5 h. Subsequently, the reaction was diluted with EtOAc (75 mL), washed with H_2_O (7 × 75 mL), dried over anhydrous Na_2_SO_4_, filtered, and concentrated under reduced pressure. The obtained crude was purified by flash chromatography on a silica gel (EtOAc/hexane gradient, 10:90−15:85) to afford a mixture of tautomers keto-enol **10/15a**–**j**.

2-benzyl-3-hydroxy-1-phenylprop-2-en-1-one (**15a**): Synthesized in a 4.22 mmol scale and purified by column chromatography on silica gel (EtOAc/hexane, 10:90−15:85) to afford a mixture of tautomers keto-enol. White solid, 707 mg, 70% yield. ^1^H NMR (500 MHz, CDCl_3_) δ 8.67 (d, *J* = 4.0 Hz, 1H), 7.90–7.10 (m, 10H), 3.70 (s, 2H). ^13^C NMR (126 MHz, CDCl_3_) δ 188.5, 184.6, 140.5, 133.9, 130.9, 128.7, 128.4, 128.1, 127.7, 126.4, 110.2, 33.5. HRMS (ESI) [M + H]^+^ calc. For C_16_H_15_O_2_^+^ = 239.1067; found = 239.1064.

2-(4-methoxybenzyl)-3-oxo-3-phenylpropanal (**10b**): Synthesized in a 1.79 mmol scale and purified by column chromatography on silica gel (EtOAc/hexane, 10:90−15:85) to afford a mixture of tautomers keto-enol. Beige solid, 378.23 mg, 79% yield. ^1^H NMR (500 MHz, CDCl_3_) δ 9.69 (d, *J* = 2.8 Hz, 1H), 7.90–6.75 (m, 9H), 4.65 (td, *J* = 7.2, 2.8 Hz, 1H), 3.69 (s, 3H), 3.27 (d, *J* = 7.1 Hz, 2H). ^13^C NMR (126 MHz, CDCl_3_) δ 197.9, 195.8, 158.5, 136.4, 133.9, 130.0, 129.5, 128.9, 128.7, 114.1, 63.1, 55.2, 32.7. HRMS (ESI) [M + H]^+^ calc. For C_17_H_17_O_3_^+^ = 269.1172; found = 269.1170.

3-hydroxy-1-phenyl-2-(4-(trifluoromethyl)benzyl)prop-2-en-1-one (**15c**): Synthesized in a 2.31 mmol scale and purified by column chromatography on silica gel (EtOAc/hexane, 10:90−15:85) to afford a mixture of tautomers keto-enol. Pale beige solid, 506.18 mg, 71% yield. ^1^H NMR (500 MHz, CDCl_3_) δ 8.63 (s, 1H), 7.52 (d, *J* = 8.2 Hz, 2H), 7.48–7.41 (m, 3H), 7.38 (t, *J* = 7.3 Hz, 2H), 7.20 (d, *J* = 8.6 Hz, 2H), 3.75 (s, 2H). ^13^C NMR (126 MHz, CDCl_3_) δ 187.3, 185.8, 144.6, 135.5, 131.1, 128.9 (d, *J* = 32.9 Hz), 128.5, 128.3, 127.5, 125.1 (q, *J* = 3.8 Hz), 124.1 (q, *J* = 271.8 Hz), 109.6, 33.5. HRMS (ESI) [M + H]^+^ calc. For C_17_H_14_F_3_O_2_^+^ = 307.0940; found = 307.0934.

2-(4-fluorobenzyl)-3-oxo-3-phenylpropanal (**10d**): Synthesized in a 2.7 mmol scale and purified by column chromatography on silica gel (EtOAc/hexane, 10:90−15:85) to afford a mixture of tautomers keto-enol. Pale beige solid, 412.3 mg, 49% yield. ^1^H NMR (400 MHz, (CD_3_)_2_CO) δ 9.81 (d, *J* = 1.9 Hz, 1H), 8.03–6.97 (m, 9H), 5.09 (td, *J* = 7.1, 1.9 Hz, 1H), 3.43–3.24 (m, 2H). ^13^C NMR (101 MHz, (CD_3_)_2_CO) δ 197.6, 195.4, 161.5 (d, *J* = 243.0 Hz), 136.8, 134.5 (d, *J* = 3.4 Hz), 133.7, 130.9, 130.8, 128.8, 128.6, 114.9 (d, *J* = 21.4 Hz), 62.3, 31.5. HRMS (ESI) [M + H]^+^ calc. For C_16_H_14_FO_2_^+^ = 257.0972; found = 257.0970.

2-(3-fluorobenzyl)-3-oxo-3-phenylpropanal (**10e**): Synthesized in a 4.48 mmol scale and purified by column chromatography on silica gel (EtOAc/hexane, 10:90−15:85) to afford a mixture of tautomers keto-enol. White solid, 743 mg, 65% yield. ^1^H NMR (500 MHz, (CD_3_)_2_CO) δ 9.83 (d, *J* = 1.8 Hz, 1H), 8.05–6.85 (m, 9H), 5.15 (td, *J* = 7.1, 1.8 Hz, 1H), 3.40 (dd, *J* = 14.2, 6.8 Hz, 1H), 3.32 (dd, *J* = 14.2, 7.5 Hz, 1H). ^13^C NMR (126 MHz, (CD_3_)_2_CO) δ 197.4, 195.2, 162.8 (d, *J* = 242.7 Hz), 144.3 (d, *J* = 7.2 Hz), 136.7, 133.7, 130.4, 129.7 (d, *J* = 9.1 Hz), 128.7, 128.1, 124.4, 115.0 (d, *J* = 21.0 Hz), 112.2 (d, *J* = 21.0 Hz), 62.0, 31.9. HRMS (ESI) [M + H]^+^ calc. For C_16_H_14_FO_2_^+^ = 257.0972; found = 257.0969.

2-(2-fluorobenzyl)-3-oxo-3-phenylpropanal (**10f**): Synthesized in a 2.03 mmol scale and purified by column chromatography on silica gel (EtOAc/hexane, 10:90−15:85) to afford a mixture of tautomers keto-enol. Pale yellow solid, 296.7 mg, 57% yield. ^1^H NMR (400 MHz, CDCl_3_) δ 9.75 (d, *J* = 2.5 Hz, 1H), 7.99–6.94 (m, 9H), 4.80 (td, *J* = 7.1, 2.6 Hz, 1H), 3.44 (dd, *J* = 14.2, 7.5 Hz, 1H), 3.32 (dd, *J* = 14.2, 6.8 Hz, 1H). ^13^C NMR (101 MHz, CDCl_3_) δ 197.2, 195.4, 161.2 (d, *J* = 244.9 Hz), 136.2, 134.0, 131.7 (d, *J* = 4.6 Hz), 128.9, 128.8, 128.7, 124.6, 124.4, 124.3, 124.3, 115.4 (d, *J* = 21.7 Hz), 61.1, 27.3. HRMS (ESI) [M + H]^+^ calc. For C_16_H_14_FO_2_^+^ = 257.0972; found = 257.0970.

2-benzyl-3-(4-methoxyphenyl)-3-oxopropanal (**10g**): Synthesized in a 2.51 mmol scale and purified by column chromatography on silica gel (EtOAc/hexane, 10:90−15:85) to afford a mixture of tautomers keto-enol. Yellow solid, 392.2 mg, 58% yield. ^1^H NMR (500 MHz, CDCl_3_) δ 9.71 (d, *J* = 2.9 Hz, 1H), 7.94–6.82 (m, 9H), 4.62 (td, *J* = 7.0, 2.9 Hz, 1H), 3.84 (s, 3H), 3.34 (dd, *J* = 7.0, 1.8 Hz, 2H). ^13^C NMR (126 MHz, CDCl_3_) δ 197.9, 193.7, 164.2, 140.7, 131.2, 129.5, 128.9, 128.7, 126.8, 114.1, 62.7, 55.4, 33.5. HRMS (ESI) [M + H]^+^ calc. For C_17_H_17_O_3_^+^ = 269.1172; found = 269.1169.

2-benzyl-3-(4-bromophenyl)-3-oxopropanal (**10h**): Synthesized in a 3.80 mmol scale and purified by column chromatography on silica gel (EtOAc/hexane, 10:90−15:85) to afford a mixture of tautomers keto-enol. Pale beige solid, 818.5 mg, 68% yield. ^1^H NMR (500 MHz, CDCl_3_) δ 9.71 (s, 1H), 7.72–7.05 (m, 9H), 4.60 (td, *J* = 7.2, 2.7 Hz, 1H), 3.34 (d, *J* = 7.6 Hz, 2H). ^13^C NMR (126 MHz, CDCl_3_) δ 197.3, 194.6, 140.2, 137.3, 132.2, 130.1, 129.4, 128.8, 128.0, 127.0, 62.9, 33.6. HRMS (ESI) [M + H]^+^ calc. For C_16_H_14_BrO_2_^+^ = 317.0172; found = 317.0169.

3-(4-chlorophenyl)-2-(4-fluorobenzyl)-3-oxopropanal (**10i**): Synthesized in a 2.44 mmol scale and purified by column chromatography on silica gel (EtOAc/hexane, 10:90−15:85) to afford a mixture of tautomers keto-enol. Pale yellow solid, 412.5 mg, 58% yield. ^1^H NMR (400 MHz, CDCl_3_) δ 9.69 (d, *J* = 2.6 Hz, 1H), 7.80–6.85 (m, 8H), 4.61 (td, *J* = 7.1, 2.7 Hz, 1H), 3.31 (d, *J* = 7.2 Hz, 2H). ^13^C NMR (126 MHz, (CD_3_)_2_CO) δ 197.5, 194.6, 161.6 (d, *J* = 242.7 Hz), 139.5, 135.4, 134.4 (d, *J* = 2.9 Hz), 130.9, 130.4, 129.0, 115.0 (d, *J* = 21.5 Hz), 62.1, 31.5. HRMS (ESI) [M + H]^+^ calc. For C_16_H_13_ClFO_2_^+^ = 291.0583; found = 291.0580.

1-(benzo[*d*][1,3]dioxol-5-yl)-2-(4-fluorobenzyl)-3-hydroxyprop-2-en-1-one (**15j**): Synthesized in a 1.67 mmol scale and purified by column chromatography on silica gel (EtOAc/hexane, 10:90−15:85) to afford a mixture of tautomers keto-enol. White solid, 454.7 mg, 91% yield. White solid. ^1^H NMR (500 MHz, CDCl_3_) δ 8.56 (d, *J* = 4.7 Hz, 1H), 7.12–7.05 (m, 2H), 7.03 (dd, *J* = 8.1, 1.8 Hz, 1H), 7.01–6.95 (m, 3H), 6.77 (d, *J* = 8.0 Hz, 1H), 6.00 (s, 2H), 3.70 (s, 1H). ^13^C NMR (126 MHz, CDCl_3_) δ 187.0, 184.7, 161.6 (d, *J* = 245.1 Hz), 150.1, 147.8, 136.1 (d, *J* = 3.3 Hz), 129.5, 129.4, 129.4, 123.0, 115.5 (d, *J* = 21.0 Hz), 109.7, 108.2, 108.1, 101.6, 33.1. HRMS (ESI) [M + H]^+^ calc. For C_17_H_14_FO_4_^+^ = 301.0871; found = 301.0868.

### 3.6. General Procedure for the Synthesis of Compounds ***12a–j*** and ***5a***

A mixture of tautomers keto-enol **10/15a**–**j** (1.0 equiv., 0.06 mmol), (*R*,*R*)-D (0.0024 mmol, 4 mol%), formic acid/triethylamine mole ratio (5:4) in toluene (0.25 M) was stirred under argon at rt for 16 h. Following this, the reaction was diluted with EtOAc (20 mL), dried over anhydrous Na_2_SO_4_, filtered over a pad of silica gel, and concentrated under reduced pressure. The residue was purified by preparative thin-layer chromatography (PTLC), resulting in the isolation of products **12a**–**j**.

(1*R*,2*R*)-2-benzyl-1-phenylpropane-1,3-diol (**12a**): Purified by preparative TLC (DCM/petroleum ether/EtOAc, 2:5:2, 100 mL). White waxy solid, 9.74 mg, 0.06 mmol, 67% yield (containing 9% of the diastereoisomer, 61% corrected yield). Enantiomeric excess (>99% ee), was determined by HPLC analysis using a Phenomenex LC column 250 × 4.6 mm—Lux 5 µm Amylose-2, 90:10 hexane/iPrOH, flow rate 1 mL/min, at 208 nm (RT_maj_ = 10.399, RT_min_ = 16.160). Specific rotation: $\alpha_{D}^{25}=+1.17\;\left(c\;0.1,MeOH\right);+3.96\;\left(c\;0.2,CHCl3\right).$ ^1^H NMR (500 MHz, (CD_3_)_2_CO) δ 7.40 (d, *J* = 6.7 Hz, 2H), 7.35 (t, *J* = 7.6 Hz, 2H), 7.29–7.23 (m, 3H), 7.23–7.15 (m, 3H), 4.82 (t, *J* = 5.0 Hz, 1H), 4.70 (d, *J* = 4.5 Hz, 1H), 3.86 (t, *J* = 5.1 Hz, 1H), 3.69 (d, *J* = 10.8 Hz, 1H), 3.52–3.43 (m, 1H), 2.75–2.63 (m, 2H), 2.14–2.08 (m, 1H). ^13^C NMR (126 MHz, (CD_3_)_2_CO) δ 144.7, 141.2, 129.1, 128.2, 127.9, 126.8, 126.4, 125.7, 75.2, 61.2, 49.5, 34.1. HRMS (ESI) [M + Na]^+^ calc. For C_16_H_18_NaO_2_^+^ = 265.1199; found = 265.1197.

(1*R*,2*R*)-2-(4-methoxybenzyl)-1-phenylpropane-1,3-diol (**12b**): Purified by preparative TLC (DCM/petroleum ether/EtOAc, 2:5:2, 100 mL). Dark gray waxy solid, 12.5 mg, 0.06 mmol, 76% yield (containing 13% of the diastereoisomer, 66% corrected yield). Enantiomeric excess (>99% ee), was determined by HPLC analysis using a Phenomenex LC column 250 × 4.6 mm—Lux 5 µm Amylose-2, 85:15 hexane/iPrOH, flow rate 1 mL/min, at 278 nm (RT_maj_ = 10.325, RT_min_ = 18.241). Specific rotation: $\alpha_{D}^{25}=+1.58\;\left(c\;0.1,MeOH\right).$ ^1^H NMR (400 MHz, CDCl_3_) δ 7.41–7.27 (m, 5H), 7.05 (d, *J* = 8.7 Hz, 2H), 6.81 (d, *J* = 8.7 Hz, 2H), 4.76 (d, *J* = 6.2 Hz, 1H), 3.77 (s, 3H), 3.75–3.72 (m, 1H), 3.57 (dd, *J* = 11.1, 5.5 Hz, 1H), 2.66 (dd, *J* = 13.9, 5.8 Hz, 1H), 2.55 (dd, *J* = 13.9, 9.3 Hz, 1H), 2.48 (s, 1H), 2.12–2.01 (m, 1H). ^13^C NMR (126 MHz, (CD_3_)_2_CO) δ 158.0, 144.7, 132.8, 129.9, 127.9, 126.8, 126.4, 113.6, 75.2, 61.2, 54.5, 49.6, 33.2. HRMS (ESI) [M + Na]^+^ calc. For C_17_H_20_NaO_3_^+^ = 295.1305; found = 295.1303.

(1*R*,2*R*)-1-phenyl-2-(4-(trifluoromethyl)benzyl)propane-1,3-diol (**12c**): Purified by preparative TLC (DCM/petroleum ether/EtOAc, 1:3:1, 100 mL). Colorless oil, 13.7 mg, 0.06 mmol, 73% yield (containing 9% of the diastereoisomer, 66% corrected yield). Enantiomeric excess (>99% ee), was determined by HPLC analysis using a Phenomenex LC column 250 × 4.6 mm—Lux 5 µm Amylose-2, 95:5 hexane/iPrOH, flow rate 1 mL/min, at 208 nm (RT_maj_ = 12.846, RT_min_ = 20.204). Specific rotation: $\alpha_{D}^{25}=+1.39\;\left(c\;0.1,MeOH\right).$ ^1^H NMR (500 MHz, (CD_3_)_2_CO) δ 7.60 (d, *J* = 7.8 Hz, 2H), 7.43 (d, *J* = 7.8 Hz, 2H), 7.39 (d, *J* = 7.0 Hz, 2H), 7.33 (t, *J* = 7.6 Hz, 2H), 7.24 (t, *J* = 7.3 Hz, 1H), 4.81 (t, *J* = 5.1 Hz, 1H), 4.72 (s, 1H), 3.89 (s, 1H), 3.68 (d, *J* = 10.9 Hz, 1H), 3.48–3.40 (m, 1H), 2.80 (d, *J* = 7.5 Hz, 2H), 2.19–2.10 (m, 1H). ^13^C NMR (126 MHz, (CD_3_)_2_CO) δ 146.2, 144.4, 129.8, 128.0, 127.0 (q, *J* = 86.4 Hz), 126.9, 126.4, 125.0 (q, *J* = 3.8 Hz), 124.7 (q, *J* = 270 Hz), 74.7, 60.7, 49.3, 33.9. HRMS (ESI) [M + FA − H]^−^ calc. For C_18_H_18_F_3_O_4_^−^ = 355.1163; found = 355.1162.

(1*R*,2*R*)-2-(4-fluorobenzyl)-1-phenylpropane-1,3-diol (**12d**): Purified by preparative TLC (DCM/petroleum ether/EtOAc, 1:3:1, 100 mL). Colorless oil, 13.6 mg, 0.06 mmol, 87% yield (containing 12% of the diastereoisomer, 77% corrected yield). Enantiomeric excess (>99% ee), was determined by HPLC analysis using a Phenomenex LC column 250 × 4.6 mm—Lux 5 µm Amylose-2, 90:10 hexane/iPrOH, flow rate 1 mL/min, at 267 nm (RT_maj_ = 9.123, RT_min_ = 14.316). Specific rotation: $\alpha_{D}^{25}=+1.51\;\left(c\;0.1,MeOH\right).$ ^1^H NMR (400 MHz, CDCl_3_) δ 7.41–7.34 (m, 4H), 7.33–7.27 (m, 1H), 7.10 (dd, *J* = 8.8, 5.4 Hz, 2H), 6.95 (t, *J* = 8.8 Hz, 2H), 4.78 (d, *J* = 6.2 Hz, 1H), 3.77 (dd, *J* = 11.1, 2.7 Hz, 1H), 3.57 (dd, *J* = 11.0, 5.4 Hz, 1H), 2.70 (dd, *J* = 13.9, 6.0 Hz, 1H), 2.61 (dd, *J* = 13.8, 9.2 Hz, 1H), 2.13–2.02 (m, 1H). ^13^C NMR (126 MHz, (CD_3_)_2_CO) δ 161.2 (d, *J* = 241.3 Hz), 144.6, 137.1 (d, *J* = 3.3 Hz), 130.7 (d, *J* = 8.1 Hz), 127.9, 126.8, 126.4, 114.7 (d, *J* = 21.0 Hz), 74.9, 60.9, 49.5, 33.3. HRMS (ESI) [M + FA − H]^−^ calc. For C_17_H_18_FO_4_^−^ = 305.1195; found = 305.1194.

(1*R*,2*R*)-2-(3-fluorobenzyl)-1-phenylpropane-1,3-diol (**12e**): Purified by preparative TLC (DCM/petroleum ether/EtOAc, 1:3:1, 100 mL). Dark gray waxy solid, 8.5 mg, 0.06 mmol, 54% yield (containing 9% of the diastereoisomer, 49% corrected yield). Enantiomeric excess (>99% ee), was determined by HPLC analysis using a Phenomenex LC column 250 × 4.6 mm—Lux 5 µm Amylose-2, 90:10 hexane/iPrOH, flow rate 1 mL/min, at 263 nm (RT_maj_ = 10.485, RT_min_ = 14.573). Specific rotation: $\alpha_{D}^{25}=+1.48\;\left(c\;0.1,MeOH\right).$ ^1^H NMR (400 MHz, (CD_3_)_2_CO) δ 7.42–7.18 (m, 6H), 7.10–6.85 (m, 3H), 4.80 (t, *J* = 5.0 Hz, 1H), 4.70 (d, *J* = 4.6 Hz, 1H), 3.88 (t, *J* = 4.9 Hz, 1H), 3.73–3.62 (m, 1H), 3.45 (m, 1H), 2.72 (d, *J* = 7.8 Hz, 2H), 2.10 (m, 1H). ^13^C NMR (126 MHz, (CD_3_)_2_CO) δ 205.4, 162.8 (d, *J* = 243.2 Hz), 144.5, 144.2 (d, *J* = 7.2 Hz), 129.9 (d, *J* = 8.6 Hz), 128.0, 126.9, 126.4, 125.1, 115.7 (d, *J* = 21.0 Hz), 112.4 (d, *J* = 21.5 Hz)., 74.8, 60.9, 49.3, 33.9, 28.8. HRMS (ESI) [M + Na]^+^ calc. For C_16_H_17_FNaO_2_^+^ = 283.1105; found = 283.1102.

(1*R*,2*R*)-2-(2-fluorobenzyl)-1-phenylpropane-1,3-diol (**12f**): Purified by preparative TLC (DCM/petroleum ether/EtOAc, 1:3:1, 100 mL). Brown oil, 11.7 mg, 0.06 mmol, 74% yield (containing 14% of the diastereoisomer, 64% corrected yield). Enantiomeric excess (>99% ee), was determined by HPLC analysis using a Phenomenex LC column 250 × 4.6 mm—Lux 5 µm Amylose-2, 90:10 hexane/iPrOH, flow rate 1 mL/min, at 209 nm (RT_maj_ = 10.603, RT_min_ = 15.706). Specific rotation: $\alpha_{D}^{25}=+1.21\;\left(c\;0.1,MeOH\right).$ ^1^H NMR (400 MHz, (CD_3_)_2_CO) δ 7.43–7.18 (m, 7H), 7.15–6.99 (m, 2H), 4.82 (t, *J* = 5.0 Hz, 1H), 4.72 (d, *J* = 4.5 Hz, 1H), 3.88 (t, *J* = 4.9 Hz, 1H), 3.75–3.62 (m, 1H), 3.53–3.40 (m, 1H), 2.72 (d, *J* = 6.2 Hz, 2H), 2.21–2.09 (m, 1H). ^13^C NMR (101 MHz, (CD_3_)_2_CO) δ 161.3 (d, *J* = 243.6 Hz), 144.4, 131.7 (d, *J* = 5.0 Hz), 127.9, 127.8 (d, *J* = 4.8 Hz), 126.9, 126.4, 126.1, 124.0 (d, *J* = 3.5 Hz), 115.0 (d, *J* = 22.4 Hz), 75.2, 61.2, 47.9, 27.4. HRMS (ESI) [M + Na]^+^ calc. For C_16_H_17_FNaO_2_^+^ = 283.1105; found = 283.1103.

(1*R*,2*R*)-2-benzyl-1-(4-methoxyphenyl)propane-1,3-diol (**12g**): Purified by preparative TLC (DCM/petroleum ether/EtOAc, 2:5:2, 100 mL). Colorless oil, 17.6 mg, 0,094 mmol, 69% yield (containing 20% of the diastereoisomer, 55% corrected yield). Enantiomeric excess (>99% ee), was determined by HPLC analysis using a Phenomenex LC column 250 × 4.6 mm—Lux 5 µm Amylose-2, 85:15 hexane/iPrOH, flow rate 1 mL/min, at 276 nm (RT_maj_ = 11.265, RT_min_ = 17.587). Specific rotation: $\alpha_{D}^{25}=+1.21\;\left(c\;0.1,MeOH\right).$ ^1^H NMR (500 MHz, (CD_3_)_2_CO) δ 7.31 (d, *J* = 8.6 Hz, 2H), 7.25 (m, 2H), 7.19–7.14 (m, 3H), 6.90 (d, *J* = 8.7 Hz, 2H), 4.75 (d, *J* = 6.4 Hz, 1H), 4.64 (s, 1H), 3.88 (s, 1H), 3.78 (s, 3H), 3.68 (dd, *J* = 10.7, 3.9 Hz, 1H), 3.46 (dd, *J* = 10.7, 5.4 Hz, 1H), 2.65 (dd, *J* = 13.5, 5.6 Hz, 1H), 2.56 (dd, *J* = 13.6, 9.2 Hz, 1H), 2.04 (s, 1H). ^13^C NMR (126 MHz, (CD_3_)_2_CO) δ 158.8, 141.2, 136.5, 129.0, 128.1, 127.6, 125.7, 113.3, 75.1, 61.4, 54.6, 49.5, 34.1. HRMS (ESI) [M + Na]^+^ calc. For C_17_H_20_NaO_3_^+^ = 295.1305; found = 295.1302.

(1*R*,2*R*)-2-benzyl-1-(4-bromophenyl)propane-1,3-diol (**12h**): Purified by preparative TLC (DCM/petroleum ether/EtOAc, 1:3:1, 100 mL). Colorless oil, 12.7 mg, 0.06 mmol, 66% yield (containing 12% of the diastereoisomer, 58% corrected yield). Enantiomeric excess (>99% ee), was determined by HPLC analysis using a Phenomenex LC column 250 × 4.6 mm—Lux 5 µm Amylose-2, 95:5 hexane/iPrOH, flow rate 1 mL/min, at 222 nm (RT_maj_ = 19.504, RT_min_ = 26.438). Specific rotation: $\alpha_{D}^{25}=+1.14\;\left(c\;0.1,MeOH\right).$ ^1^H NMR (400 MHz, CDCl_3**,**_) δ 7.48 (d, *J* = 8.4 Hz, 2H), 7.33–7.19 (m, 5H), 7.14 (d, *J* = 8.8 Hz, 2H), 4.74 (d, *J* = 5.9 Hz, 1H), 3.73 (d, *J* = 10.6 Hz, 1H), 3.57 (dd, *J* = 11.1, 5.3 Hz, 1H), 2.73 (dd, *J* = 13.7, 5.9 Hz, 1H), 2.62 (dd, *J* = 14.0, 9.3 Hz, 1H), 2.10–1.95 (m, 1H). ^13^C NMR (101 MHz, (CD_3_)_2_CO) δ 144.0, 140.9, 130.9, 129.2, 128.6, 128.4, 125.7, 120.1, 74.2, 61.0, 49.3, 34.0. HRMS (ESI) [M + H]^+^ calc. For C_16_H_18_BrO_2_^+^ = 321.0485; found = 321.0306.

(1*R*,2*R*)-1-(4-chlorophenyl)-2-(4-fluorobenzyl)propane-1,3-diol (**12i**): Synthesized in a 0.06 mmol scale and purified by preparative TLC (DCM/petroleum ether/EtOAc, 1:3:1, 100 mL). Colorless oil, 13 mg, 73% yield (containing 11% of the diastereoisomer, 65% corrected yield). Enantiomeric excess (>99% ee), was determined by HPLC analysis using a Phenomenex LC column 250 × 4.6 mm—Lux 5 µm Amylose-2, 95:5 hexane/iPrOH, flow rate 1 mL/min, at 222 nm (RT_maj_ = 16.478, RT_min_ = 21.835). Specific rotation: $\alpha_{D}^{25}=+1.09\;\left(c\;0.1,MeOH\right).$ ^1^H NMR (400 MHz, CDCl_3_) δ 7.37–7.24 (m, 4H), 7.09 (dd, *J* = 8.8, 5.4 Hz, 2H), 6.95 (t, *J* = 8.8 Hz, 2H), 4.74 (d, *J* = 5.9 Hz, 1H), 3.73 (dd, *J* = 10.9, 2.8 Hz, 1H), 3.55 (dd, *J* = 10.9, 5.3 Hz, 1H), 2.70 (dd, *J* = 13.9, 6.2 Hz, 1H), 2.61 (dd, *J* = 13.9, 9.2 Hz, 1H), 2.05–1.94 (m, 1H). ^13^C NMR (126 MHz, (CD_3_)_2_CO) δ 161.1 (d, *J* = 241.8 Hz), 143.5, 136.9, 132.0, 130.7 (d, *J* = 7.6 Hz), 128.1, 127.9, 114.7 (d, *J* = 21.5 Hz), 74.1, 60.7, 49.4, 33.1. HRMS (ESI) [M + Na]^+^ calc. For C_16_H_16_ClFNaO_2_^+^ = 317.0715; found = 317.0713.

(1*R*,2*R*)-1-(benzo[*d*][1,3]dioxol-5-yl)-2-(4-fluorobenzyl)propane-1,3-diol (**12j**): Purified by preparative TLC (DCM/petroleum ether/EtOAc, 1:2:1, 100 mL). Colorless oil, 15.4 mg, 0.067 mmol, 75% yield (containing 8% of the diastereoisomer, 69% corrected yield). Enantiomeric excess (>99% ee), was determined by HPLC analysis using a Phenomenex LC column 250 × 4.6 mm—Lux 5 µm Amylose-2, 85:15 hexane/iPrOH, flow rate 1 mL/min, at 286 nm (RT_maj_ = 12.147, RT_min_ = 20.759). Specific rotation: $\alpha_{D}^{25}=+0.69\;\left(c\;0.1,MeOH\right).$ ^1^H NMR (500 MHz, (CD_3_)_2_CO) δ 7.21 (dd, *J* = 8.7, 5.5 Hz, 2H), 7.01 (t, *J* = 8.9 Hz, 2H), 6.91 (d, *J* = 1.8 Hz, 1H), 6.85–6.78 (m, 2H), 5.97 (s, 2H), 4.71 (t, *J* = 4.3 Hz, 1H), 4.64 (d, *J* = 4.4 Hz, 1H), 3.68 (dt, *J* = 10.6, 4.5 Hz, 1H), 3.45 (dt, *J* = 10.5, 5.1 Hz, 1H), 2.69–2.57 (m, 2H), 2.04–1.97 (m, 1H). ^13^C NMR (126 MHz, (CD_3_)_2_CO) δ 161.2 (d, *J* = 241.3 Hz), 147.6, 146.5, 138.7, 137.1, 130.7 (d, *J* = 8.1 Hz), 119.7, 114.7 (d, *J* = 21.0 Hz), 107.5, 106.7, 100.9, 75.0, 61.0, 49.5, 33.2. HRMS (ESI) [M + Na]^+^ calc. For C_17_H_17_FNaO_4_^+^ = 327.1003; found = 327.1001.

methyl (2*S*,3*R*)-3-(benzo[*d*][1,3]dioxol-5-yl)-2-(4-fluorobenzyl)-3-hydroxypropanoate (**5a**): Purified by preparative TLC (DCM/petroleum ether/EtOAc, 1:5:1, 100 mL). White solid, 18.3 mg, 0.1 mmol, 55% yield. Enantiomeric excess (82% ee), was determined by HPLC analysis using a Phenomenex LC column 250 × 4.6 mm—Lux 5 µm Amylose-2, 60:40 hexane/iPrOH, flow rate 1 mL/min, at 283 nm (RT_maj_ = 6.408, RT_min_ = 8.837). Specific rotation: $\alpha_{D}^{25}=+0.75\;\left(c\;0.1,MeOH\right).$ ^1^H NMR (500 MHz, (CD_3_)_2_CO) δ 7.12–7.06 (m, 2H), 7.00–6.94 (m, 3H), 6.90 (d, *J* = 6.4 Hz, 1H), 6.82 (d, *J* = 7.9 Hz, 1H), 6.00 (s, 2H), 4.76 (dd, *J* = 8.7, 4.6 Hz, 1H), 4.55 (d, *J* = 4.6 Hz, 1H), 3.49 (s, 3H), 3.01–2.93 (m, 1H), 2.75–2.66 (m, 1H), 2.48 (dd, *J* = 13.6, 4.3 Hz, 1H). ^13^C NMR (126 MHz, (CD_3_)_2_CO) δ 205.3, 173.6, 161.4 (d, *J* = 242.2 Hz), 147.9, 147.2, 137.0, 135.3, 130.4 (d, *J* = 8.1 Hz), 120.5, 114.8 (d, *J* = 21.0 Hz), 107.7, 106.8, 101.1, 75.1, 56.3, 50.5, 34.4. HRMS (ESI) [M + Na]^+^ calc. For C_18_H_17_FNaO_5_^+^ = 355.0952; found = 355.0949.

### 3.7. Method for the Synthesis of Compound ***16***

To a solution of 2-benzyl-1-phenylpropane-1,3-diol (**12j**) (1.0 equiv, 55.72 mg, 0.183 mmol) in dichloromethane (3 mL, 0.061 M) was added tosyl chloride (3.0 equiv, 104.67 mg, 0.549 mmol), triethylamine (3.4 equiv, 86.7 µL, 0.622 mmol), and 4-dimethylaminopyridine (15 mol%, 3.35 mg, 0.027 mmol) at 0 °C. The mixture was stirred for 20 h at rt, followed by evaporation of the solvent under reduced pressure. The crude was diluted with EtOAc (50 mL), extracted with NH_4_Cl saturated solution (2 × 50 mL) and H_2_O (3 × 50 mL). The combined organic phases were dried with anhydrous Na_2_SO_4_, filtered and evaporated. The product was purified by PTLC (dichloromethane/petroleum ether/EtOAc, 1:4:1, 100 mL) to 65% yield the desired product **16**.

(2*R*,3*R*)-3-(benzo[*d*][1,3]dioxol-5-yl)-2-(4-fluorobenzyl)-3-hydroxypropyl 4-methylbenzenesulfonate (**16**): Colorless oil, 54.5 mg, 0.183 mmol, 65% yield (containing 3% of the diastereoisomer, 63% corrected yield). Enantiomeric excess (>99% ee), was determined by HPLC analysis using a Phenomenex LC column 250 × 4.6 mm—Lux 5 µm Amylose-2, 20:80 hexane/iPrOH, flow rate 1 mL/min, at 207 nm (RT_maj_ = 7.822, RT_min_ = 17.078). Specific rotation: $\alpha_{D}^{25}=-0.44\;\left(c\;0.1,MeOH\right).$ ^1^H NMR (500 MHz, CDCl_3_) δ 7.76 (d, *J* = 8.4 Hz, 2H), 7.33 (d, *J* = 7.9 Hz, 2H), 6.93–6.83 (m, 4H), 6.80–6.70 (m, 3H), 5.96 (s, 2H), 4.57 (d, *J* = 8.1 Hz, 1H), 4.32 (dd, *J* = 9.5, 3.8 Hz, 1H), 3.81 (dd, *J* = 9.7, 3.2 Hz, 1H), 2.50–2.44 (m, 4H), 2.41 (dd, *J* = 13.6, 5.3 Hz, 1H), 2.06 (s, 1H). ^13^C NMR (126 MHz, CDCl_3_) δ 161.4 (d, *J* = 244.6 Hz), 148.1, 147.4, 144.9, 136.1, 134.6 (d, *J* = 3.3 Hz), 132.6, 130.3 (d, *J* = 8.1 Hz), 129.8, 128.0, 120.2, 115.2 (d, *J* = 21.0 Hz), 108.2, 106.6, 101.2, 73.0, 68.1, 47.7, 32.3, 21.6. HRMS (ESI) [M + FA − H]^−^ calc. For C_25_H_24_FSO_8_^−^ = 503.1176; found = 503.1181.

### 3.8. Method for the Synthesis of Compound ***17***

To a solution of intermediate **16** (1.0 equiv, 37.42 mg, 0.082 mmol) in anhydrous tetrahydrofuran (0.6 mL, 0.137 M) was added 2.5 M n-BuLi (30 µL, 0.082 mmol) at 0 °C under Ar atmosphere for 10 min. The reaction was heated to 60 °C and stirred for 22 h. Then, the phase aqueous was extracted with EtOAc (5 × 25 mL). The combined organic phases were dried with anhydrous Na_2_SO_4_, filtered, and evaporated. The product was purified by PTLC (dichloromethane/petroleum ether/EtOAc, 1:7.5:1, 100 mL) to 57% yield the desired product **17**.

5-((2*R*,3*R*)-3-(4-fluorobenzyl)oxetan-2-yl)benzo[*d*][1,3]dioxole (**17**): Pale yellow oil, 25.9 mg, 0.082 mmol, 57% yield. Enantiomeric excess (>99% ee), was determined by HPLC analysis using a Phenomenex LC column 250 × 4.6 mm—Lux 5 µm Amylose-2, 90:10 hexane/iPrOH, flow rate 1 mL/min, at 288 nm (RT_maj_ =15.557, RT_min_ = 14.480). Specific rotation: $\alpha_{D}^{25}=+2.22\;\left(c\;0.1,MeOH\right).$ ^1^H NMR (500 MHz, (CD_3_)_2_CO) δ 7.30 (dd, *J* = 8.5, 5.8 Hz, 2H), 7.06 (t, *J* = 8.9 Hz, 2H), 6.85 (d, *J* = 1.6 Hz, 1H), 6.80–6.73 (m, 2H), 6.00 (s, 2H), 5.40 (d, *J* = 6.0 Hz, 1H), 4.68–4.63 (m, 1H), 4.47 (t, *J* = 6.3 Hz, 1H), 3.18–3.12 (m, 1H), 3.12–3.09 (m, 2H). ^13^C NMR (126 MHz, (CD_3_)_2_CO) δ 161.5 (d, *J* = 242.2 Hz), 147.8, 147.2, 137.3, 135.4 (d, *J* = 3.3 Hz), 130.3 (d, *J* = 8.1 Hz), 118.8, 115.0 (d, *J* = 21.5 Hz), 107.7, 105.8, 101.1, 87.6, 72.3, 45.9, 37.8. HRMS (ESI) [M + Na]^+^ calc. For C_17_H_15_FNaO_3_^+^ = 309.0897; found = 309.0894.

## 4. Conclusions

In our pursuit of developing methods to obtain 2-alkyl-1-phenylpropane-1,3-diols, we present in this study the teth-TsDPEN-Ru(II)-catalyzed [(*R*,*R*)-**D**] one-pot double C=O reduction of α-benzyl-β-ketoaldehydes (**10**) through ATH-DKR under mild conditions. Initially, ten α-benzyl-β-ketoaldehydes (**10**) were synthesized as tautomeric keto-enol mixtures in a four-step sequence using commercially available acetophenones and benzaldehydes. These compounds were then subjected to ATH-DKR reactions.

The optimal conditions of the ATH-DKR were determined through careful optimization, considering various factors such as solvents, hydrogen sources, catalysts, additives, temperatures, and reaction times. In this process, ten *anti*-2-benzyl-1-phenylpropane-1,3-diols (**12**) (85:15 to 92:8 dr) were obtained with good yields (41–87%) and excellent enantioselectivities (>99% ee for all compounds). The reaction employed 4 mol% of (*R*,*R*)-**D** and formic acid/triethylamine mixture (5:4) as the hydrogen source in toluene at room temperature. Notably, the preferential reduction of the aldehyde moiety led to the in situ formation of 2-benzyl-3-hydroxy-1-phenylpropan-1-one intermediates (**11**). Control experiments confirmed that these intermediates (**11**) played a crucial role in enhancing both reactivity and stereoselectivity through hydrogen bonding. Finally, scale-up and functionalization experiments on the enantioenriched (1*R*,2*R*)-**12j** demonstrated the feasibility of the developed method.

## Figures and Tables

**Table 1:**
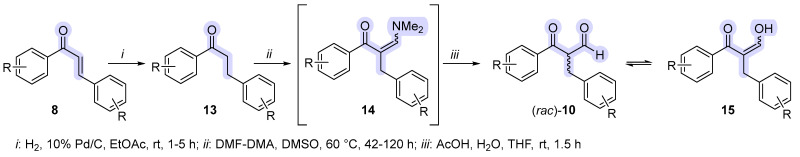
Synthesis of the α-benzyl-β-ketoaldehydes (*rac*)-**10** from chalcones (**8**).

Entry	Structure	8 ^a^	R^1^	R^2^	R^3^	13 ^b^	Yield (%) ^c^	10 ^d^	10:15 ^e^	Yield (%) ^f^
1	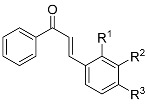	**8a**	H	H	H	**13a**	87	**10a**	4:96	70
2		**8b**	H	H	OMe	**13b**	53	**10b**	48:52	79
3		**8c**	H	H	CF_3_	**13c**	67	**10c**	0:100	71
4		**8d**	H	H	F	**13d**	65	**10d**	15:85	49
5		**8e**	H	F	H	**13e**	79	**10e**	12:88	65
6		**8f**	F	H	H	**13f**	37	**10f**	24:76	57
7	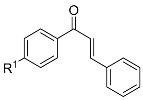	**8g**	OMe	-	-	**13g**	92	**10g**	33:67	58
8		**8h**	Br	-	-	**13h**	91	**10h**	37:63	68
9	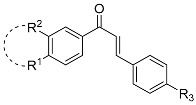	**8i**	Cl	H	F	**13i**	77	**10i**	53:47	58
10		**8j**	OCH_2_O		F	**13j**	88	**10j**	50:50	91

^a^ Obtained in a 6 mmol scale from the aldol condensation between the corresponding commercial acetophenones and aldehydes using KOH (7.5 equiv) in MeOH/H_2_O (1:1), rt, 2–27 h (65–96% yield). ^b^ Obtained in a 3–6 mmol scale. ^c^ Isolated yield after purification by column chromatography. ^d^ Obtained as a tautomeric keto-enol mixture (**10**/**15**) in a 1.7–4.5 mmol scale. ^e^ Determined by analysis of the ^1^H NMR spectrum of the isolated products. ^f^ Isolated yield of tautomeric keto-enol mixture (**10**/**15**) after purification by column chromatography.

**Table 2:**
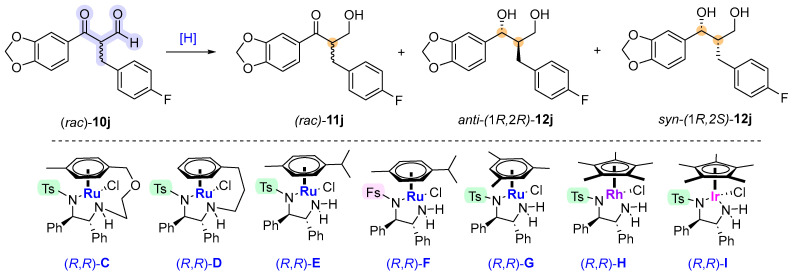
Optimization of the conditions for the ATH-DKR of (*rac*)-**10j** ^a^.

Entry	[H] Source (Ratio) ^b^	(*R*,*R*)-C-I (mol%)	Solvent ^c^	Add. ^d^	T (°C)	t (h)	11j/12j ^e^	12j anti/syn ^e^	Yield (%) ^f^	ee (%) ^g^
1	HCO_2_Na	**D** (2)	DCE/H_2_O	CTAB	rt	16	>99:1	nd	nd	nd
2	HCO_2_H/Et_3_N (5:4)	**D** (2)	DCE	-	rt	16	>99:1	nd	nd	nd
3	HCO_2_H/Et_3_N (5:4)	**D** (2)	MeCN	-	rt	16	68:32	84:16	nd	nd
4	HCO_2_H/Et_3_N (5:4)	**D** (2)	THF	-	rt	16	62:38	93:7	29	>99
5	HCO_2_H/Et_3_N (5:4)	**D** (2)	EtOAc	-	rt	16	54:46	92:8	36	98
6	HCO_2_H/Et_3_N (5:4)	**D** (2)	PhMe	-	rt	16	44:56	92:8	47	>99
7	HCO_2_H/Et_3_N (5:4)	**D** (2)	PhMe	-	45	16	7:93	87:13	92	>99
8	HCO_2_H/Et_3_N (5:4)	**C** (2)	PhMe	-	45	16	85:15	59:41	nd	nd
9	HCO_2_H/Et_3_N (5:4)	**E** (2)	PhMe	-	45	16	95:5	nd	nd	nd
10	HCO_2_H/Et_3_N (5:4)	**F** (2)	PhMe	-	45	16	93:7	nd	nd	nd
11	HCO_2_H/Et_3_N (5:4)	**G** (2)	PhMe	-	45	16	>99:1	nd	nd	nd
12	HCO_2_H/Et_3_N (5:4)	**H** (2)	PhMe	-	45	16	>99:1	nd	nd	nd
13	HCO_2_H/Et_3_N (5:4)	**I** (2)	PhMe	-	45	16	>99:1	nd	nd	nd
14	HCO_2_H/Et_3_N (5:4)	**D** (2)	PhMe	Cu(OTf)_2_	45	16	6:94	88:12	92	>99
15	HCO_2_H/Et_3_N (5:4)	**D** (2)	PhMe	TfOH	45	16	4:96	85:15	90	>99
16	HCO_2_H/Et_3_N (5:4)	**D** (4)	PhMe	-	rt	16	19:81	92:8	75	>99
17	HCO_2_H/Et_3_N (5:4)	**D** (4)	PhMe	-	rt	24	20:80	90:10	nd	nd

^a^ All reactions were carried out on a 0.085 mmol scale using (*R*,*R*)-**C**-**I**, HCO_2_Na or HCO_2_H/Et_3_N as [H] source in the quantities indicated in each case. ^b^ HCO_2_Na (6 equiv.) and to F/T 7.6 and 6.1 equivalents of each were used, respectively. ^c^ At 0.25 M, using the pure solvent or in a 1:1 mixture with water. ^d^ Using the additives: 20 mol% of CTAB (cetyltrimethylammonium bromide), 4 mol% of Cu(OTf)_2_ (copper(II) triflate), or 4 mol% of TfOH (triflic acid). ^e^ Ratios determined by analysis of the ^1^H NMR spectrum of the crude mixture; full conversion of (*rac*)-**10j** was observed in all tests. ^f^ Isolated yield for the anti/syn mixture of **12j** after preparative TLC. ^g^ Determined by HPLC analysis using chiral stationary phase column, for the major diastereoisomer. nd: not determined.

## Data Availability

Data are contained within the article and Supplementary Materials.

## Supplementary Materials

The following supporting information can be downloaded at: https://www.mdpi.com/article/10.3390/molecules29143420/s1, Table S1. Optimization of the reaction conditions for the synthesis of the enaminone **14j**; Table S2. Optimization of the conditions for the hydrolysis of enaminone **14j**; Figure S1. Control experiment 1. Figure S2. Control experiment 2. Figure S3. Absolute configuration assignment. NMR spectra of the synthetized compounds **8a**–**j**, **13a**–**j**, **12a**–**j**, **10/15a**–**j**, **4a**, **5a**, **16**, **17**. Chromatograms of compounds **12a**–**j**, **5a**, **16**, **17**.
