# Supplementary material for: Ru(II)-Catalyzed Asymmetric Transfer Hydrogenation of α-Alkyl-β-Ketoaldehydes via Dynamic Kinetic Resolution
Source: Molecules. 2024 Jul 21;29(14):3420. doi: 10.3390/molecules29143420 (PMC11279712; doi:10.3390/molecules29143420)
Supplement: Supplementary file 1 [file molecules-29-03420-s001.zip › molecules-3095265-supplementary.pdf]

SUPPORTING INFORMATION

Ru(II)-Catalyzed Asymmetric Transfer Hydrogenation of α-Alkyl-β-Ketoaldehydes via Dynamic Kinetic Resolution

Daiene P. Lapa [1], Leticia H. S. Araújo [1], Sávio R. Melo [1], Paulo R. R. Costa [1,*], and Guilherme S. Caleffi [1,*]

[1] *Laboratório de Química Bioorgânica, Instituto de Pesquisas de Produtos Naturais Walter Mors, Universidade Federal do Rio de Janeiro, 21941-902 Rio de Janeiro, Brazil*

* *Correspondence: prrcosta2011@gmail.com (PRRC); guilherme.caleffi@ippn.ufrj.br (GSC)*

Table of contentes

Table S1. Optimization of the reaction conditions for the synthesis of the enaminone **14j** 2

Table S2. Optimization of the conditions for the hydrolysis of enaminone **14j**. 2

Figure S1. Control experiment 1 3

Figure S2. Control experiment 2. 3

Figure S3. Absolute configuration assignment 4

NMR spectra of the synthetized compounds 8a-j, 13a-j, 12a-j, 10/15a-j, 4a, 5a, 16, 17 5

Chromatograms of compounds 12a-j, 5a, 16, 17 50

Table S1. Optimization of the reaction conditions for the synthesis of the enaminone **14j**

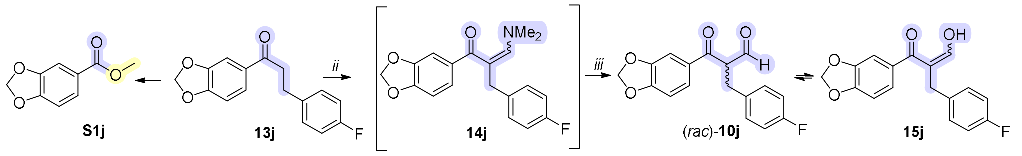

Entry	Solvent	DMF-DMA (equiv)	T (ºC)	t (h)	Conversion (%)	14:10:15:S1[a]
**1**	Toluene	1.2	70	22	9	100:0:0:0
**2**	Toluene	4	150	2	42	68:22:0:10
**3**	Toluene	4	120	19	65	96:0:0:4
**4**	DMF	2.5	80	45	86	94:3:0:3
**5**	DMF	4	150	2	100	23:0:0:77
**6**	DMSO	5	60	96	100	96:3:1:0

[a] Ratios determined by analysis of the ^{1}H NMR spectrum of the crude mixture.

Table S2. Optimization of the conditions for the hydrolysis of enaminone **14j**

Entry	13j (mmol)	Conv. (%)[a]	Add.[b]	t (h)	13:14:15:10[c]	Yield 10/15j (%)
1	0.114	100	HCl 5% (4 drops)	0.25	100:0:0:0	0
2	0.70	100	NaOH 25% (0.03 mL) H_2O (0.22 mL)	5	0:0:51:49	96
3	4.12	100	NaOH 25% (1.72 mL) H_2O (13 mL)	22	38:4:48:11	47
4	1.67	100	MeCOOH:H_2O (1:1) (6.8 mL)	1.5	5:0:70:25	91

[a] Conversion of the reaction with DMF-DMA to products **14**, **10** or **15**. [b] All hydrolysis reactions occurred at room temperature. [c] Ratios determined by analysis of the ^{1}H NMR spectrum of the crude mixture.

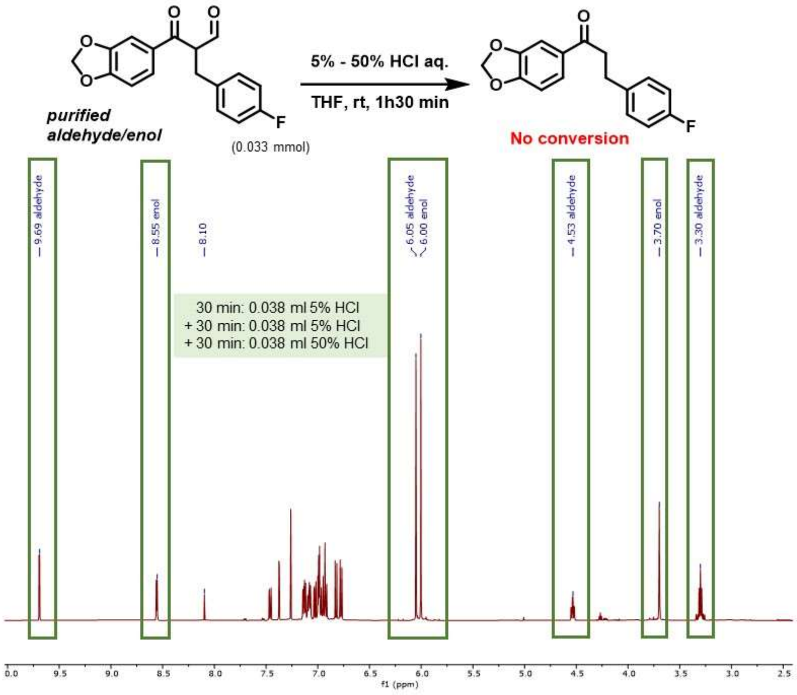

Figure S1. Control experiment 1

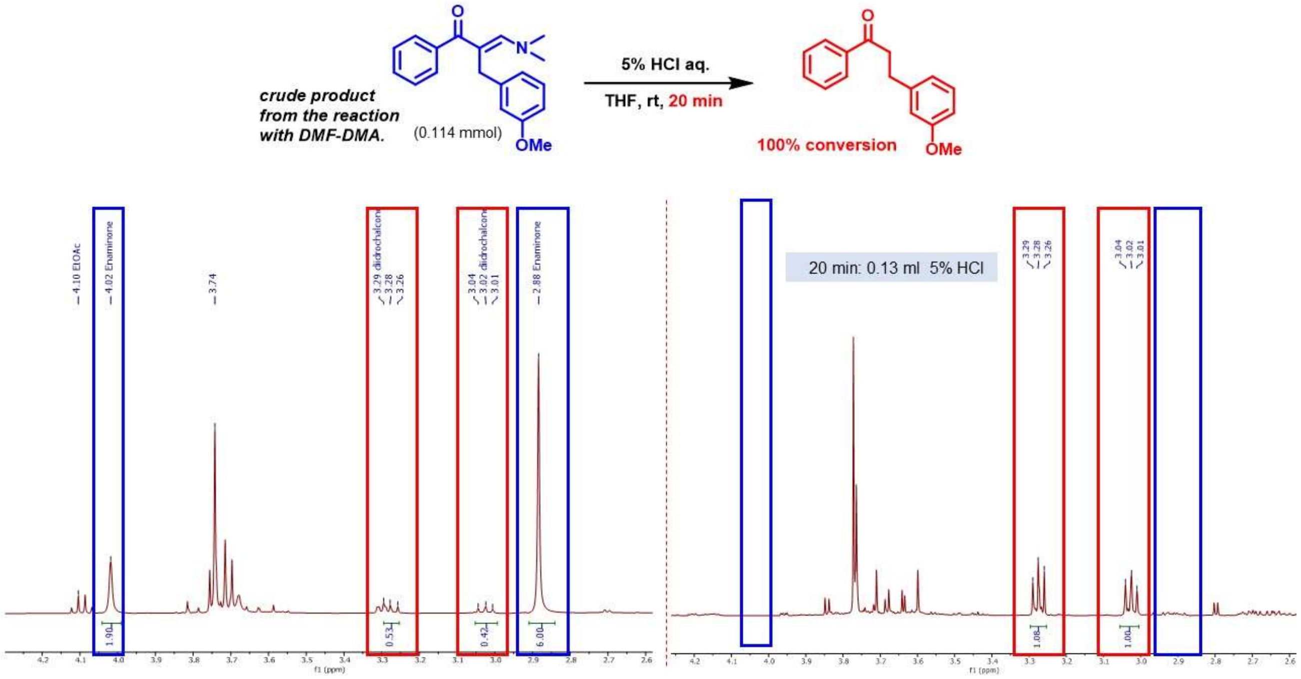

Figure S2. Control experiment 2

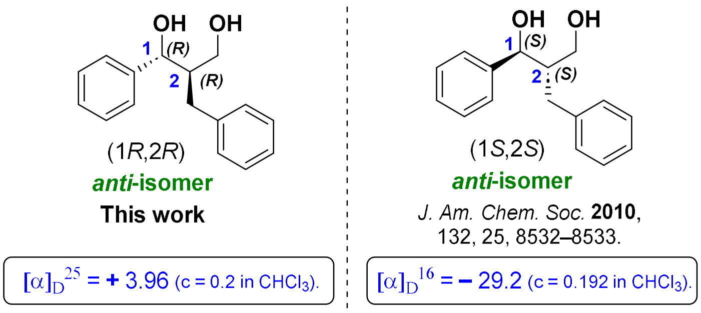

Figure S3. Absolute configuration assignment

NMR spectra of the synthetized compounds 8a-j, 13a-j, 12a-j, 10/15a-j, 4a, 5a, 16, 17

^{1}H NMR (500 MHz, CDCl$_3$) spectrum of 8a

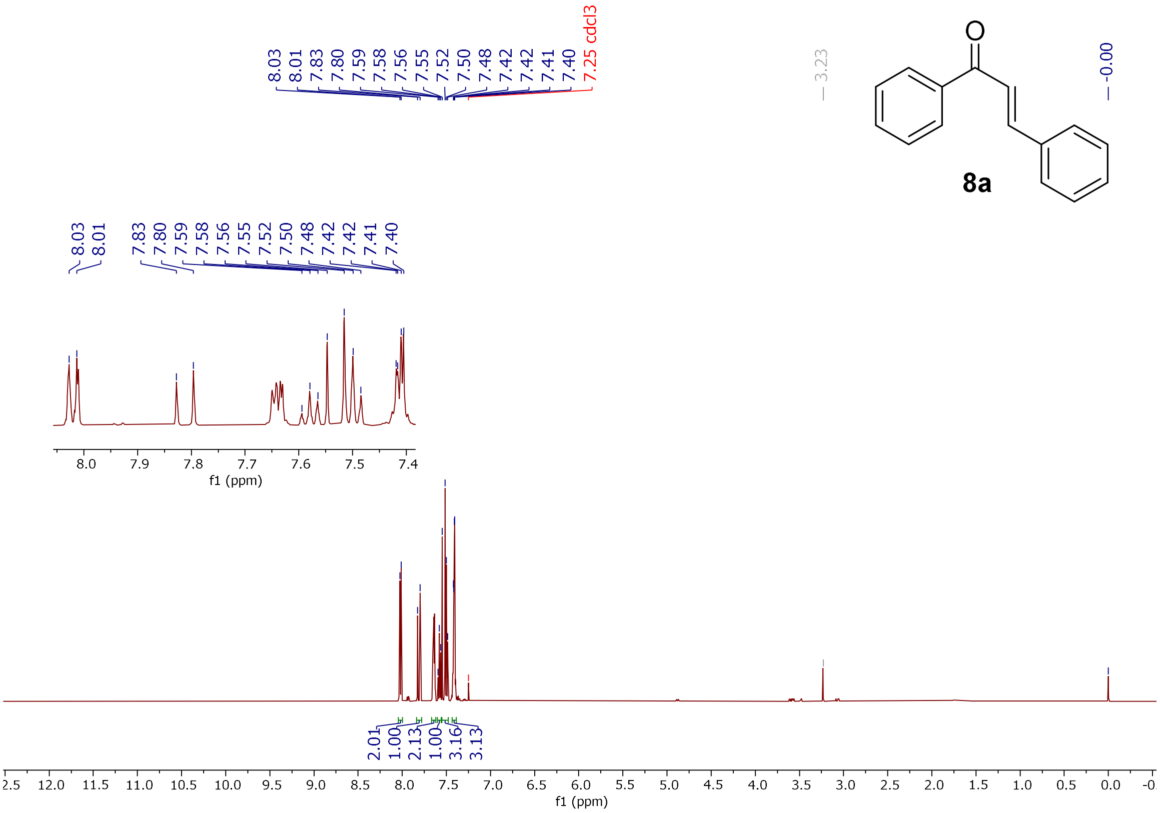

^{13}C{H} NMR (126 MHz, CDCl$_3$) spectrum of 8a

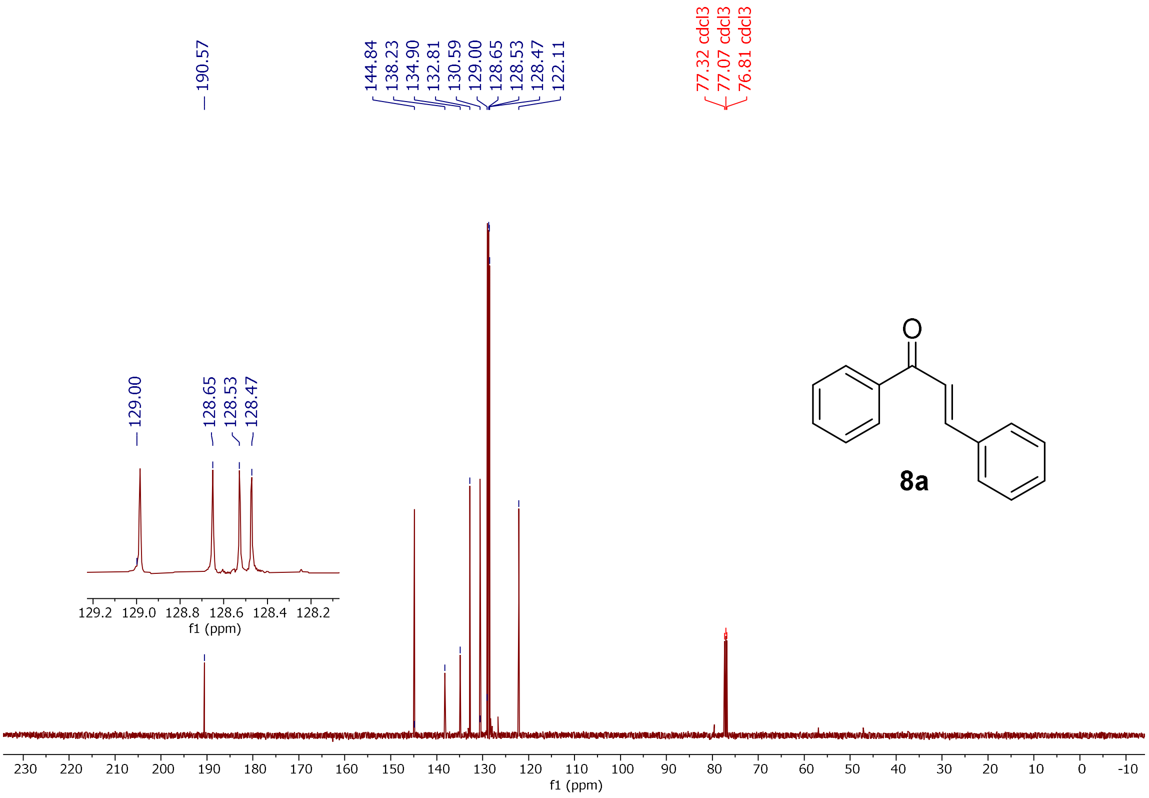

.

^{1}H NMR (500 MHz, $CDCl_3$) spectrum of 8b

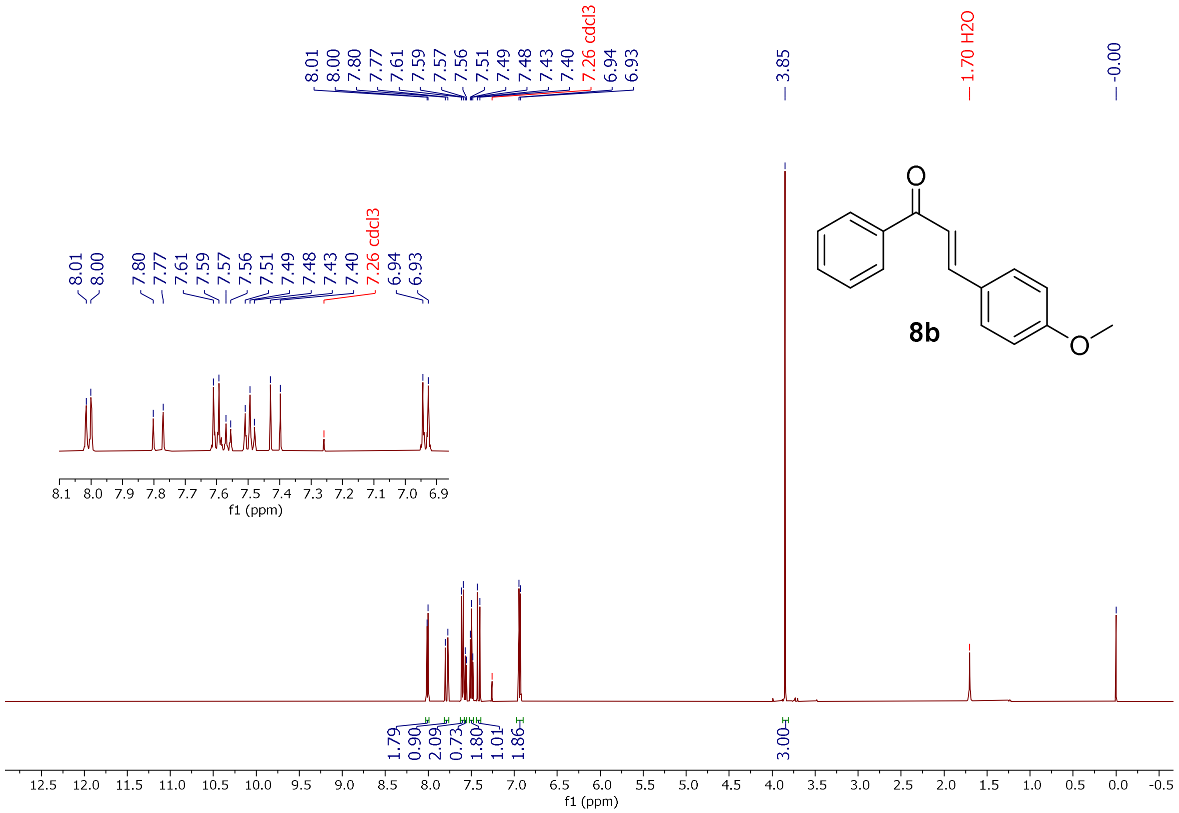

^{13}C{H} NMR (126 MHz, $CDCl_3$) spectrum of 8b

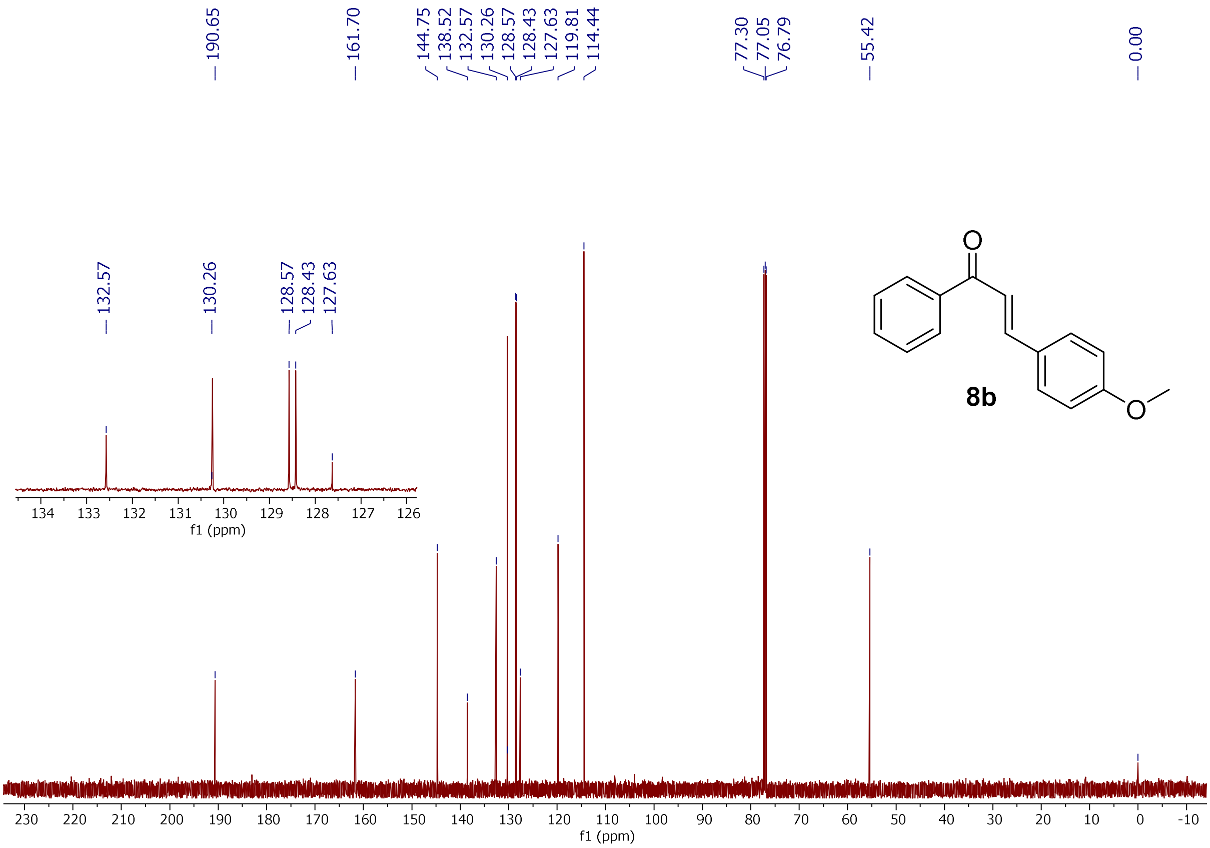

^{1}H NMR (500 MHz, $CDCl_3$) spectrum of 8c

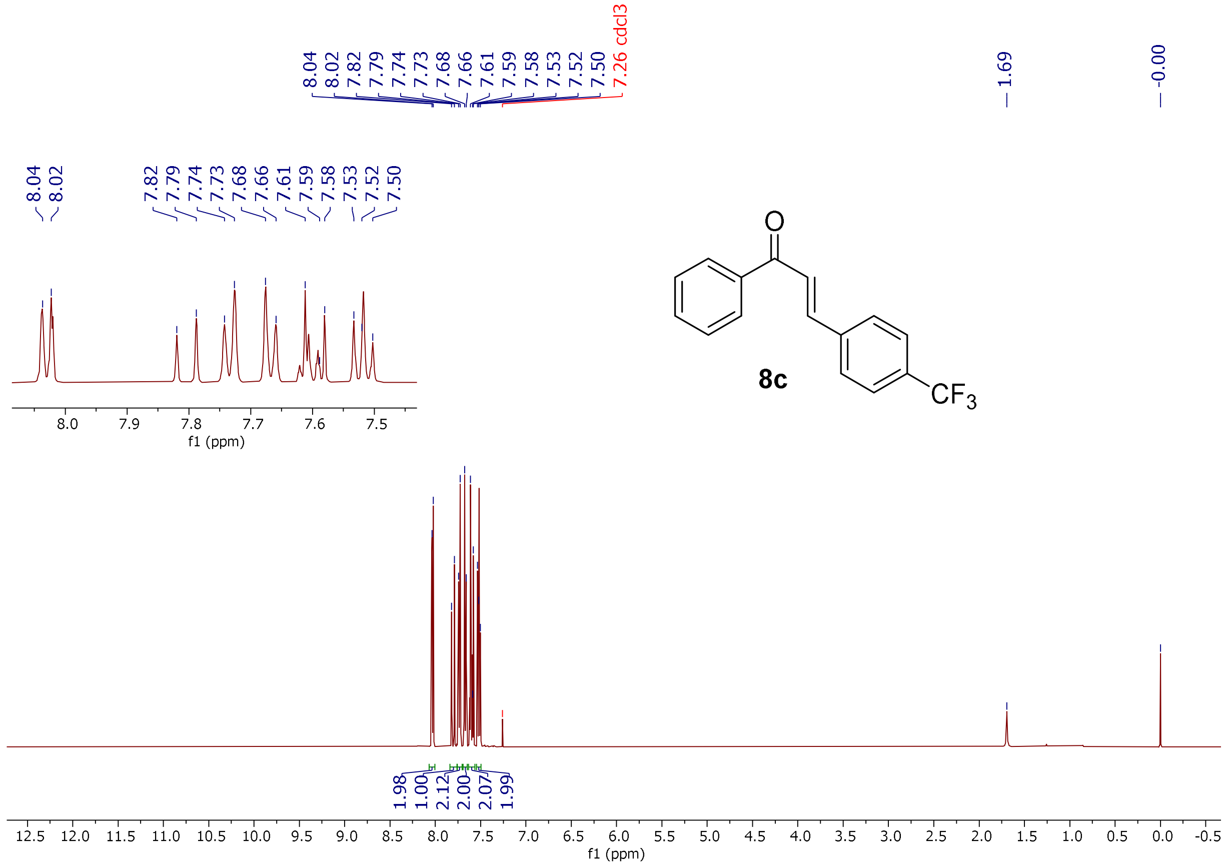

$^{13}C\{H\}$ NMR (126 MHz, $CDCl_3$) spectrum of 8c

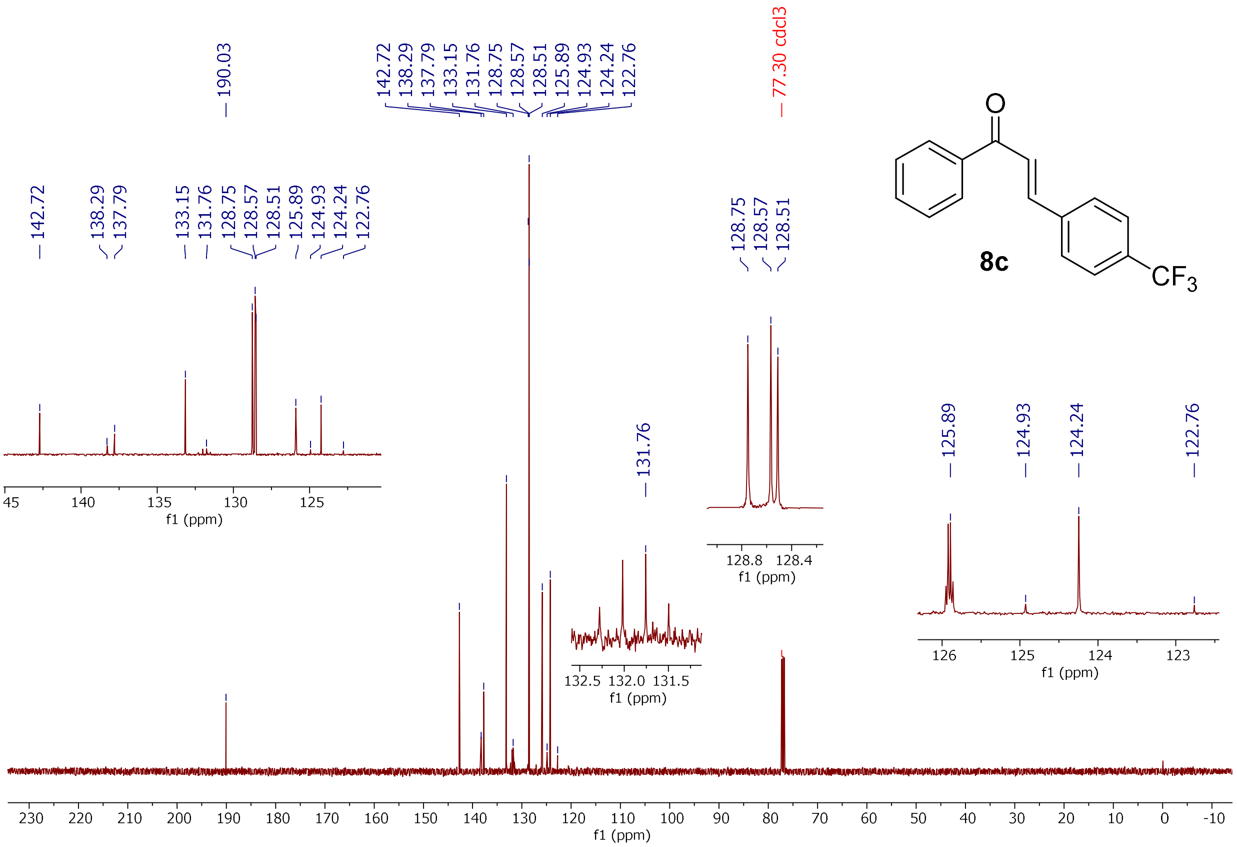

^{1}H NMR (500 MHz, $CDCl_3$) spectrum of 8d

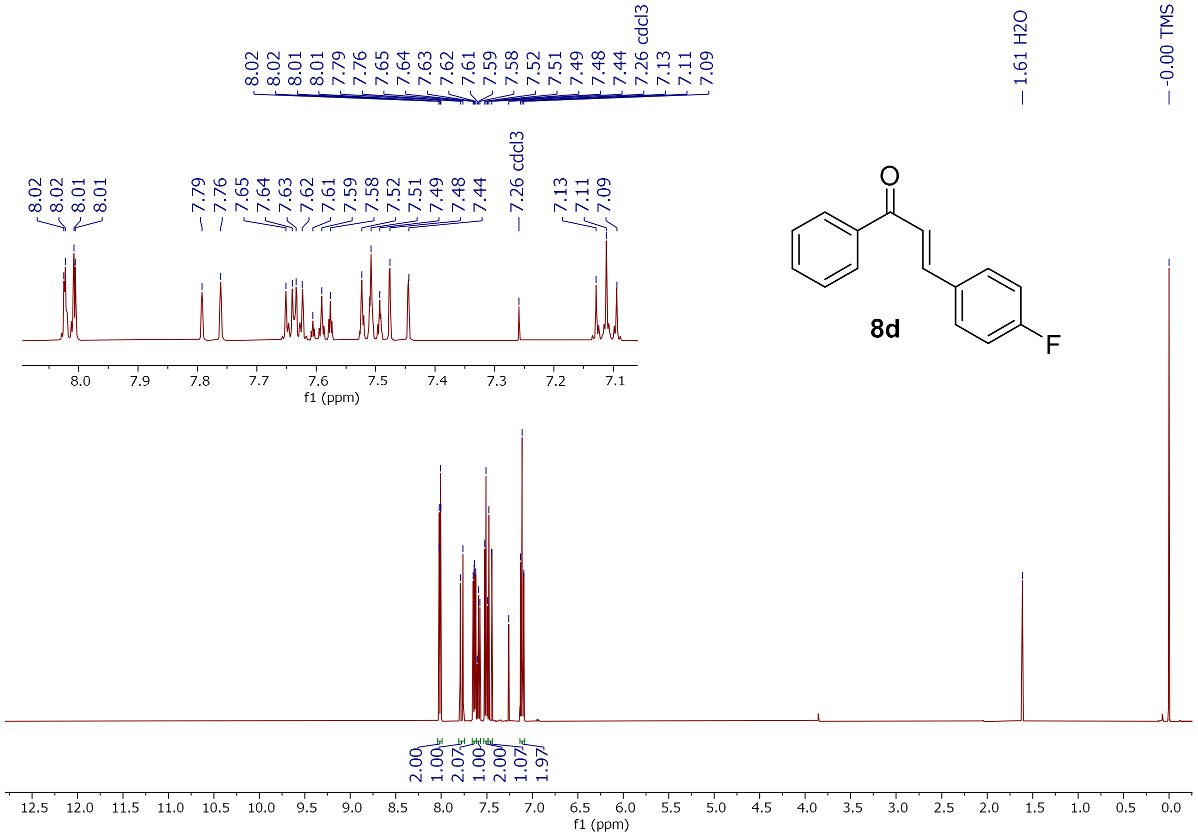

^{13}C{H} NMR (126 MHz, $CDCl_3$) spectrum of 8d

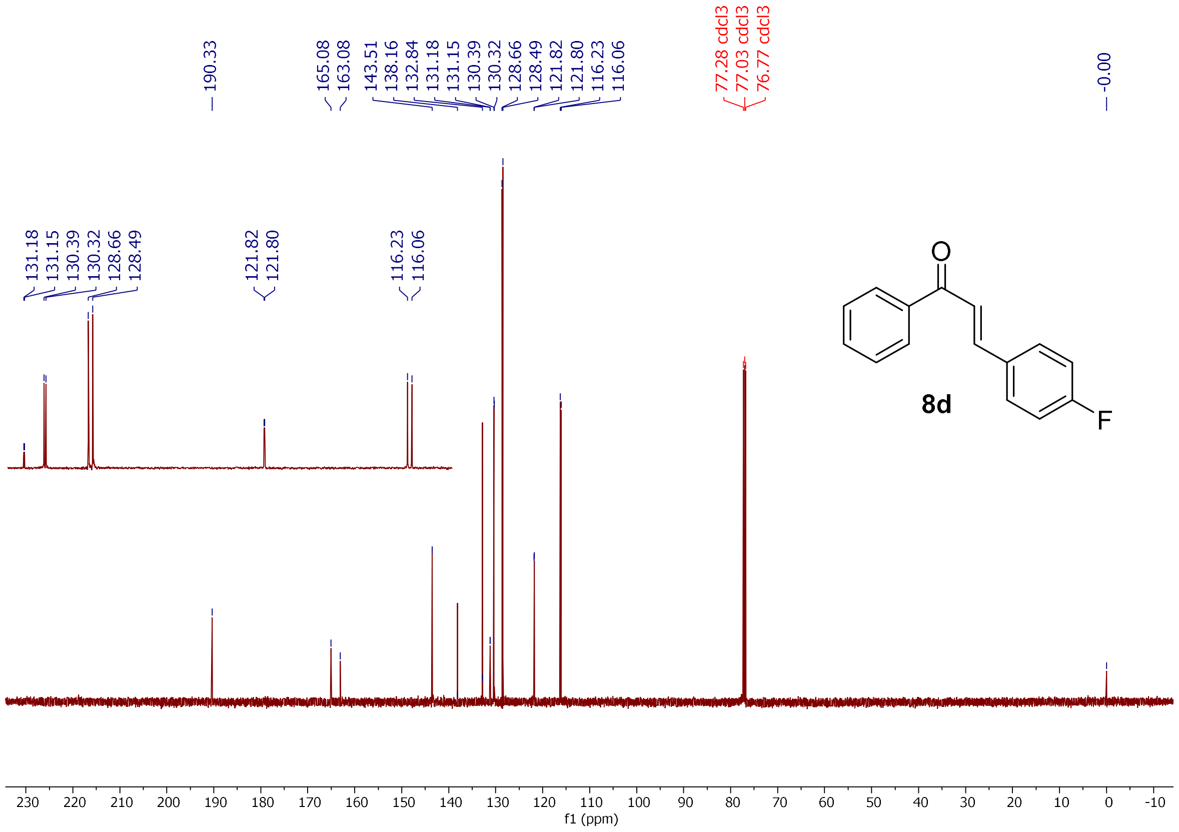

^{1}H NMR (500 MHz, $CDCl_3$) spectrum of 8e

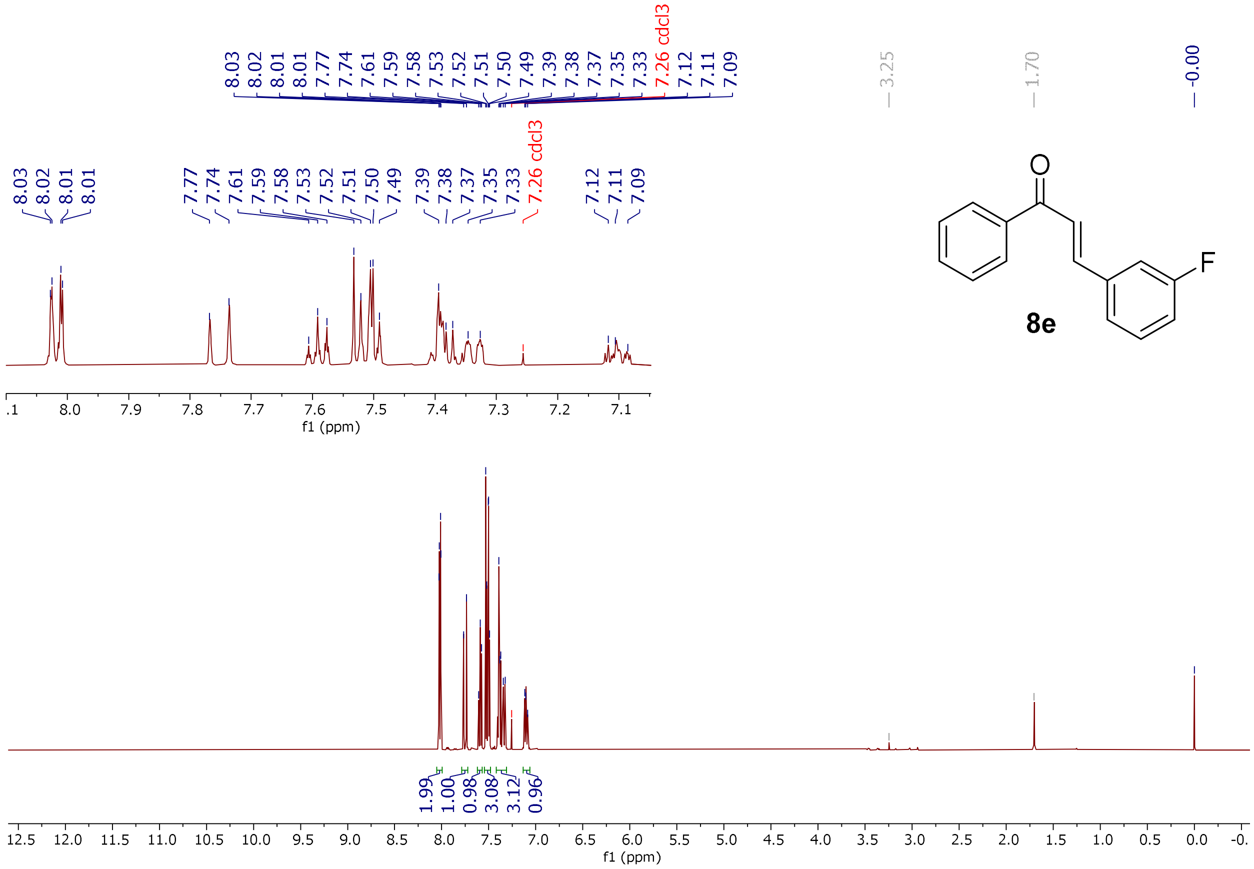

$^{13}C\{H\}$ NMR (126 MHz, $CDCl_3$) spectrum of 8e

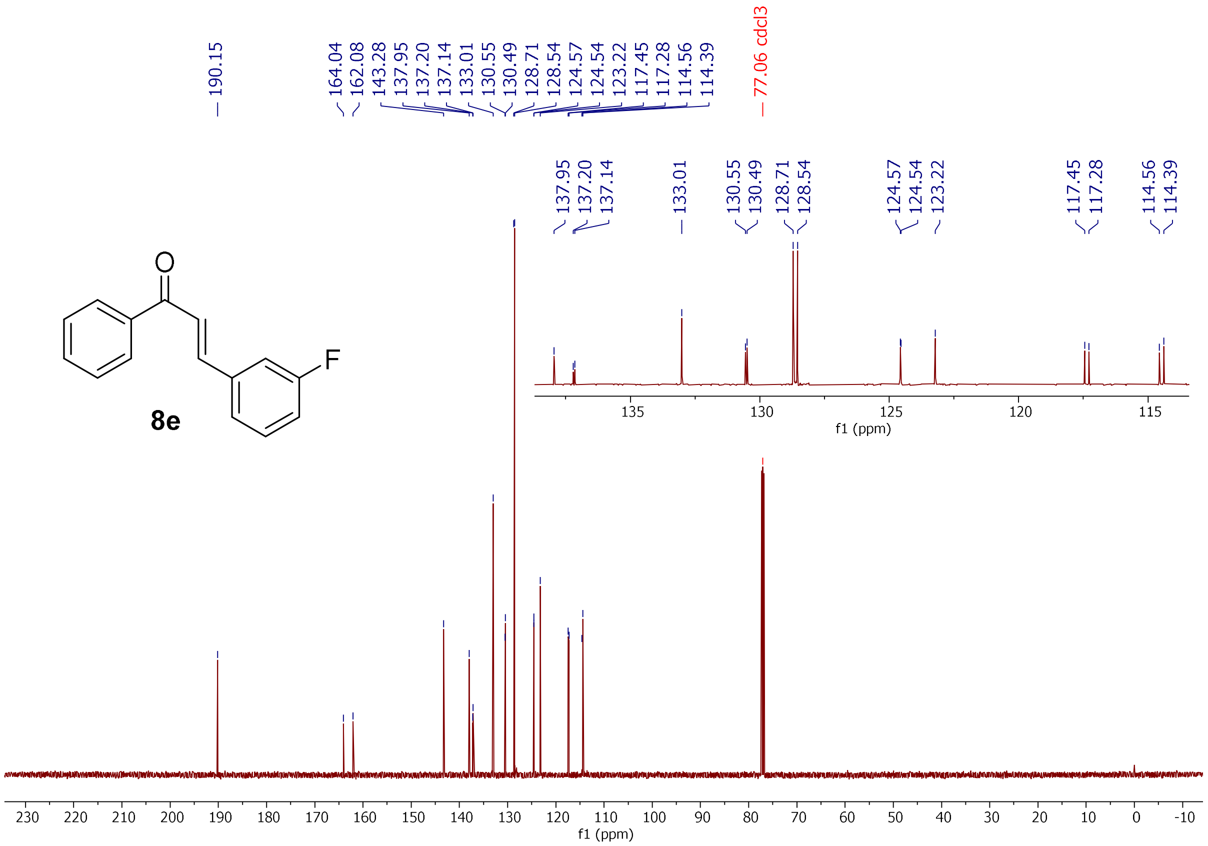

^{1}H NMR (500 MHz, $CDCl_3$) spectrum of 8f

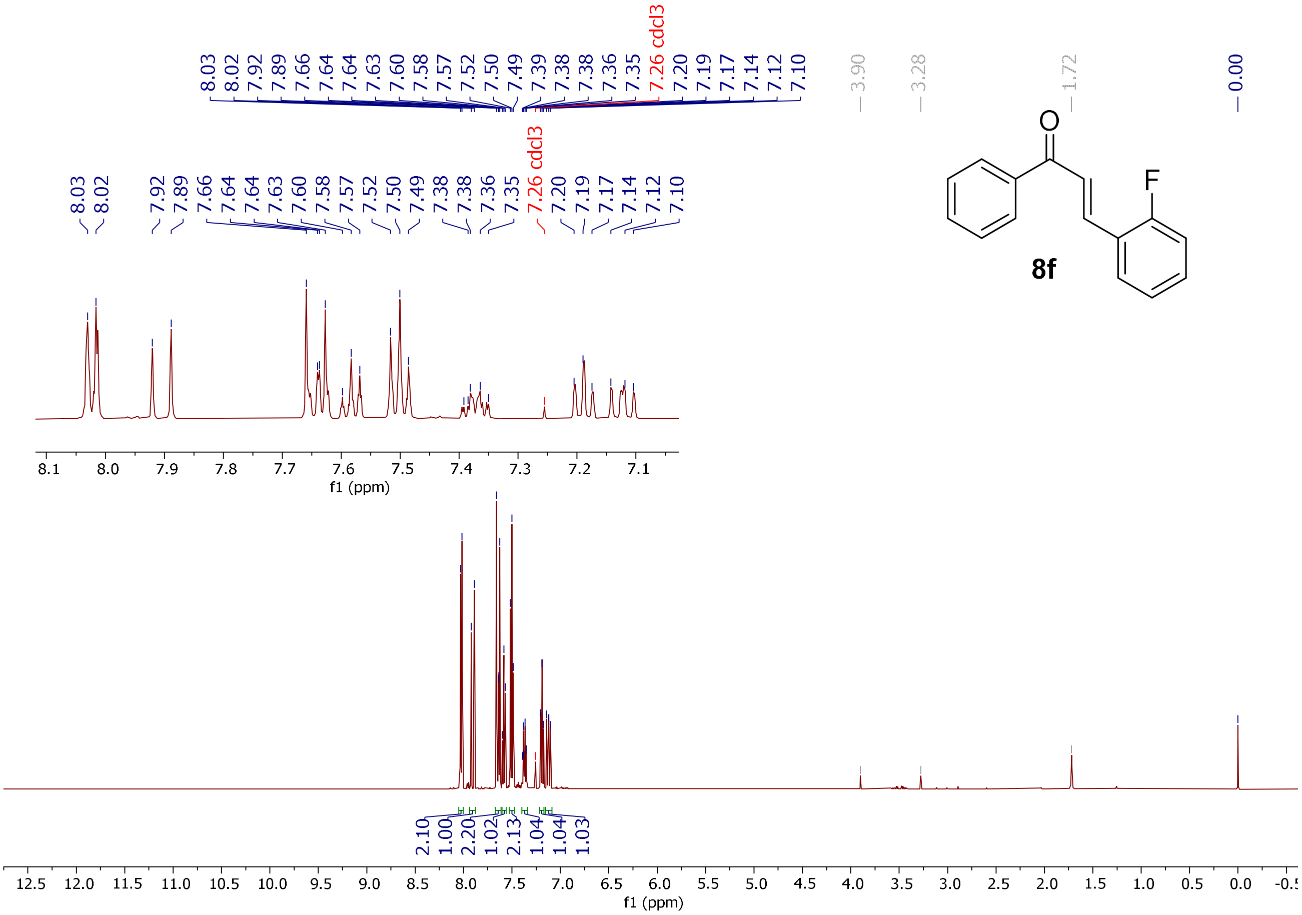

^{13}C{H} NMR (126 MHz, $CDCl_3$) spectrum of 8f

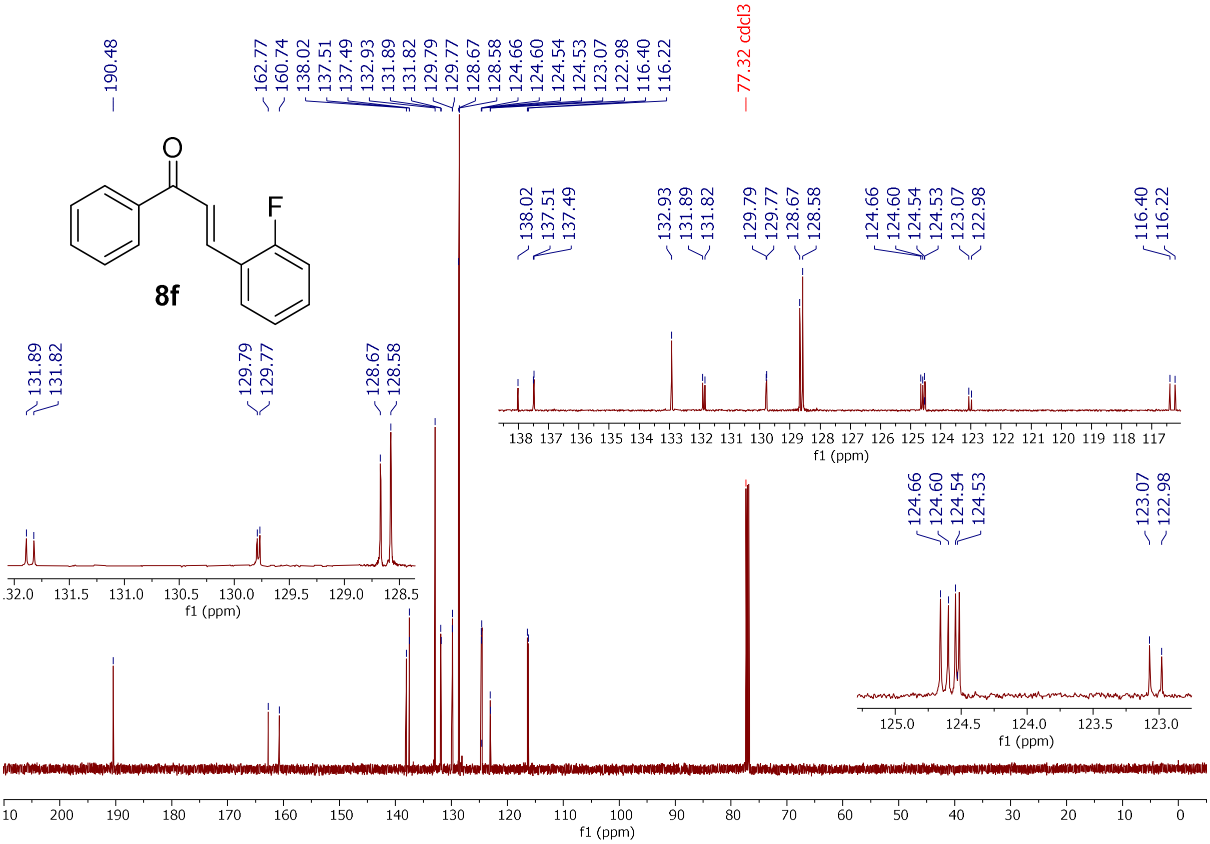

^{1}H NMR (500 MHz, $CDCl_3$) spectrum of 8g

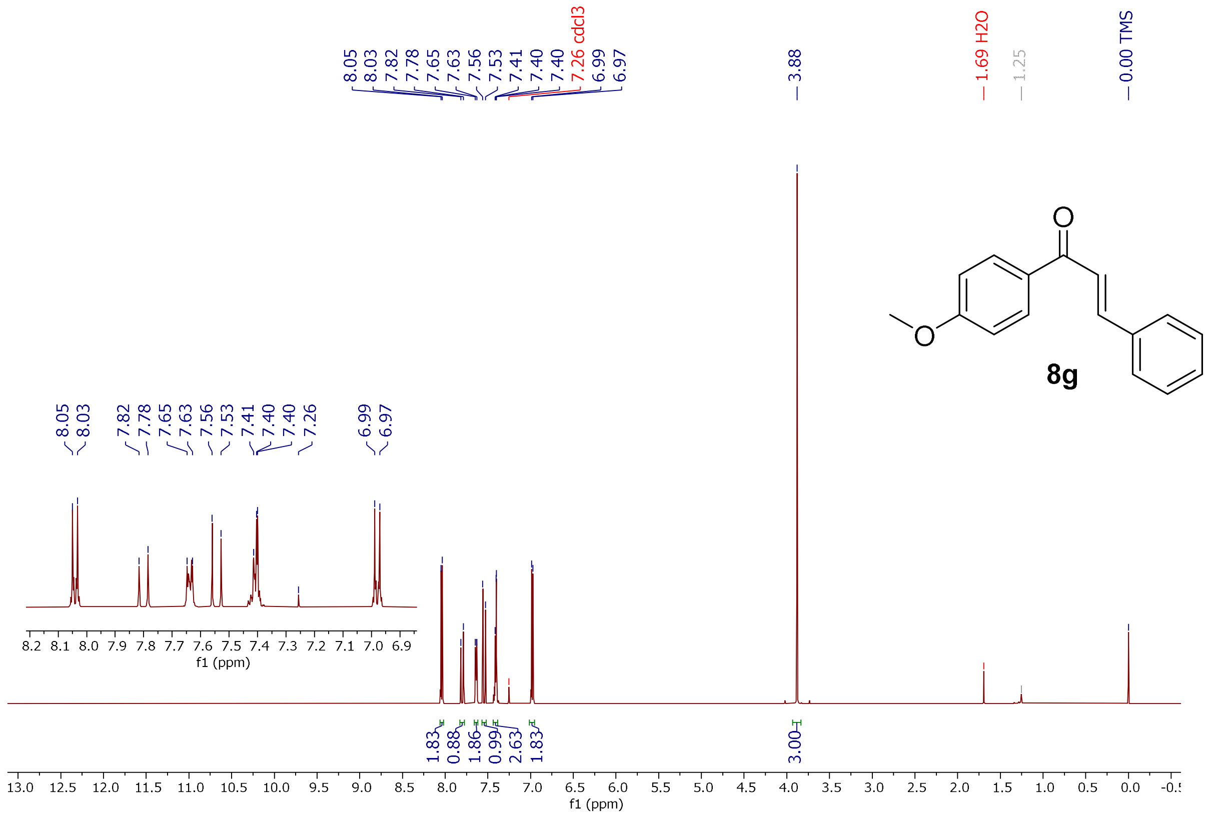

^{13}C{H} NMR (126 MHz, $CDCl_3$) spectrum of 8g

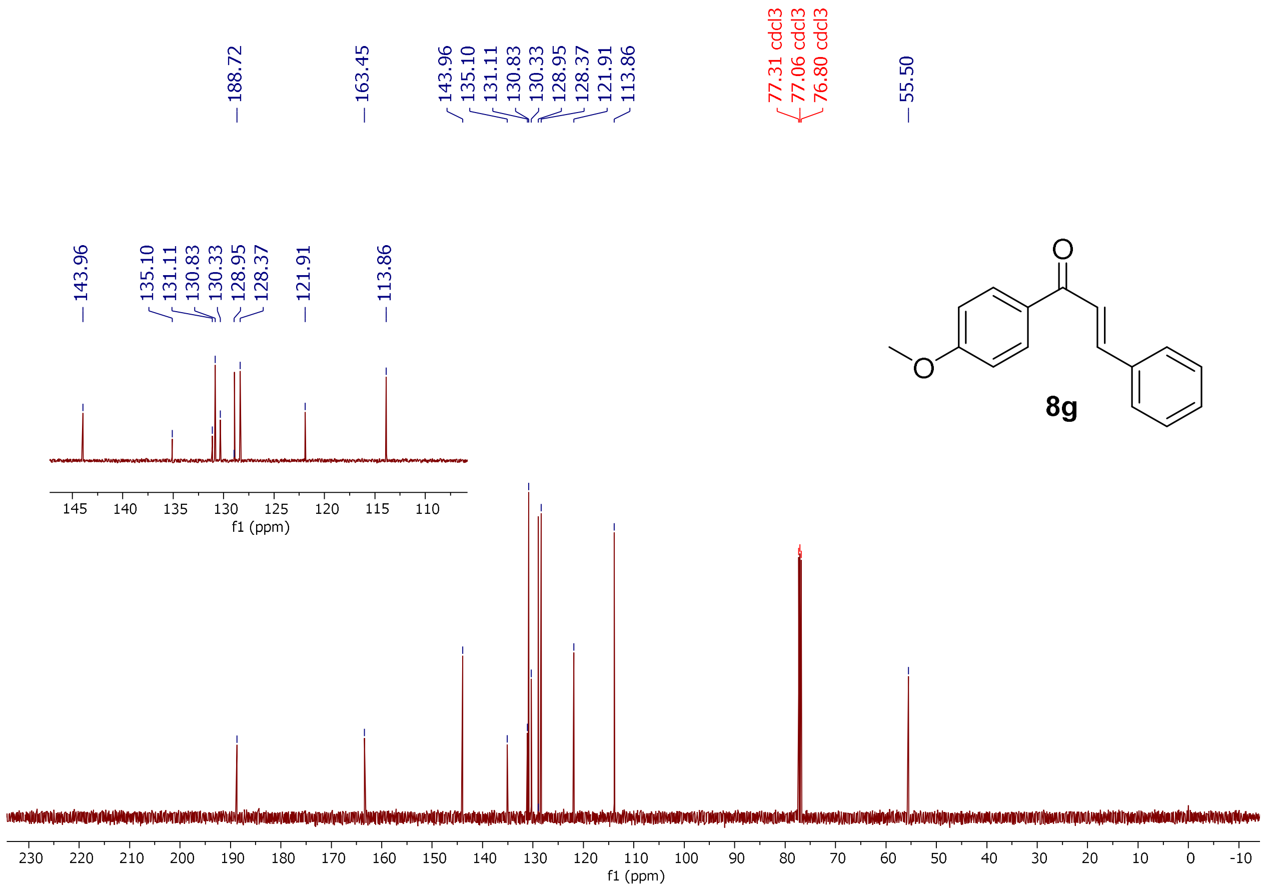

^{1}H NMR (400 MHz, $CDCl_3$) spectrum of 8h

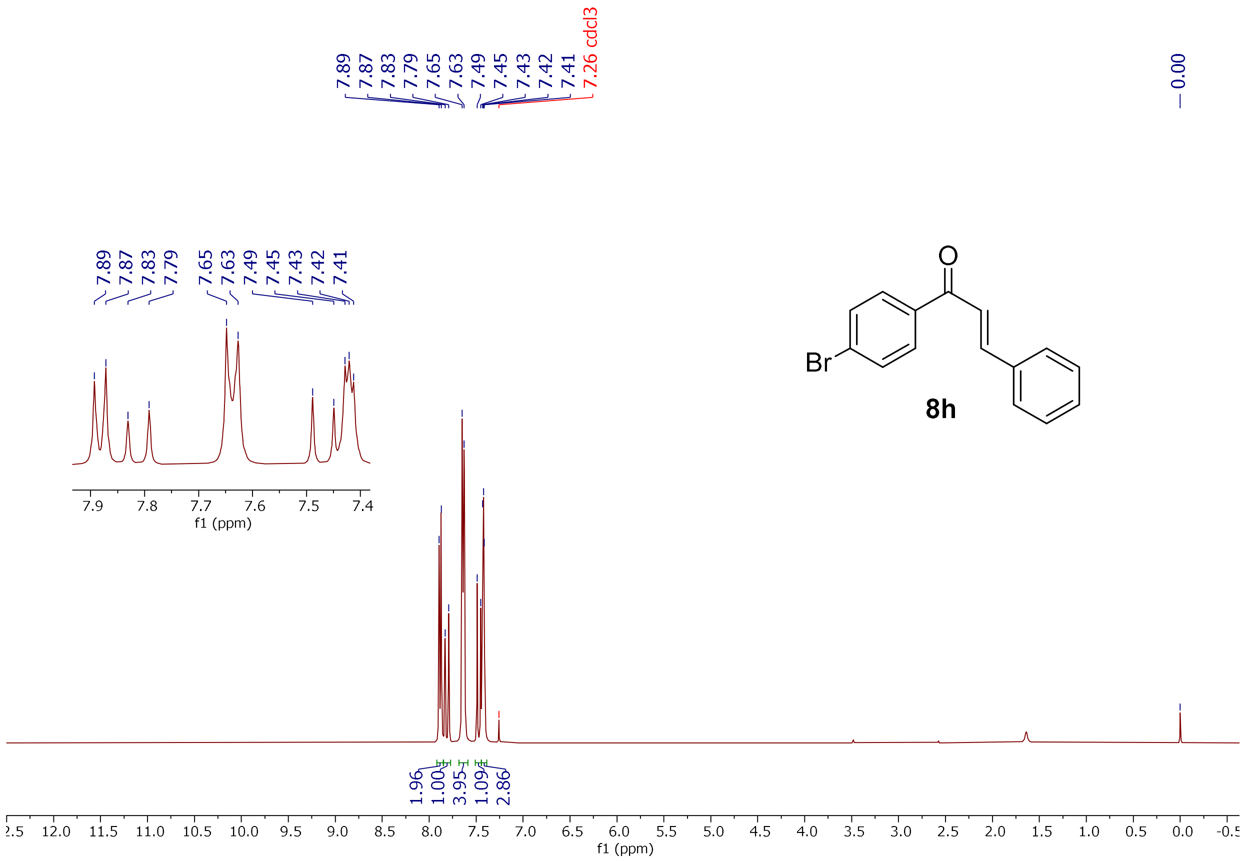

^{13}C{H} NMR (101 MHz, $CDCl_3$) spectrum of 8h

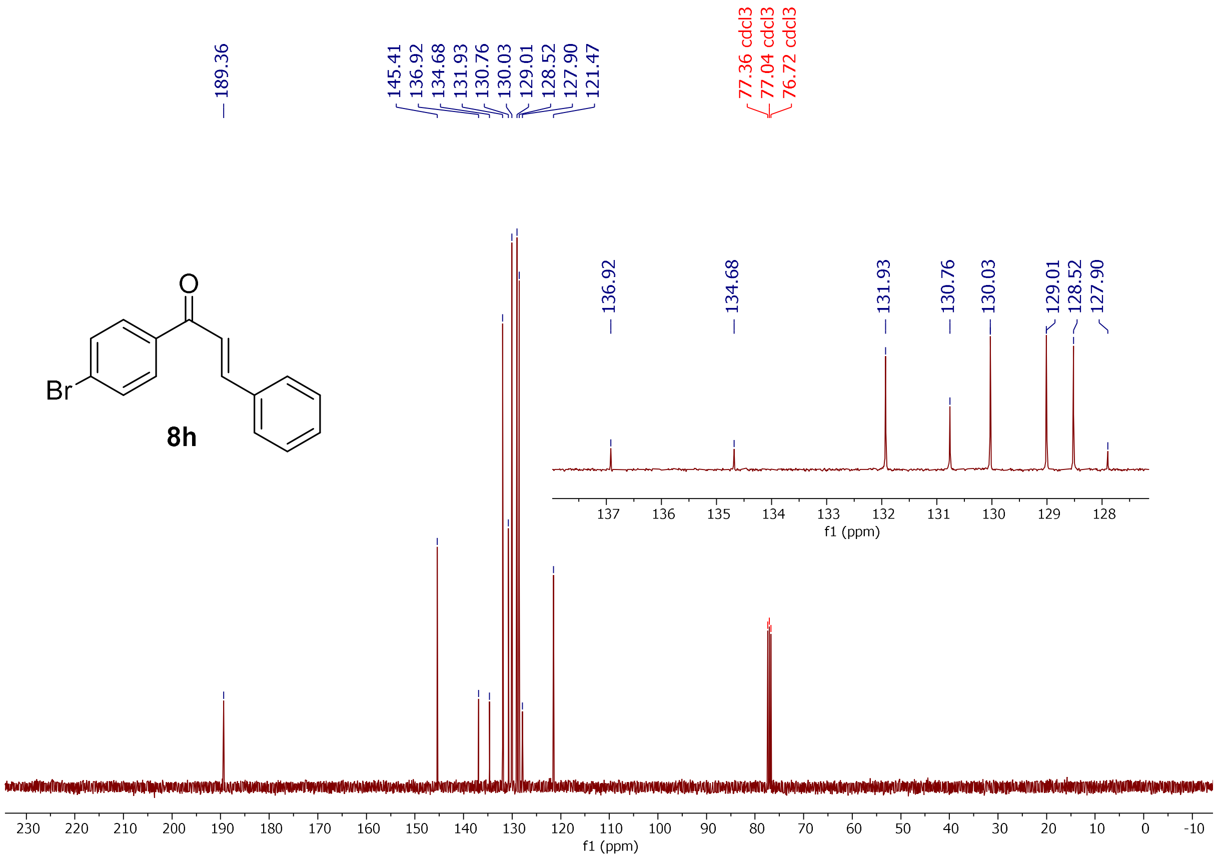

^{1}H NMR (400 MHz, $CDCl_3$) spectrum of 8i

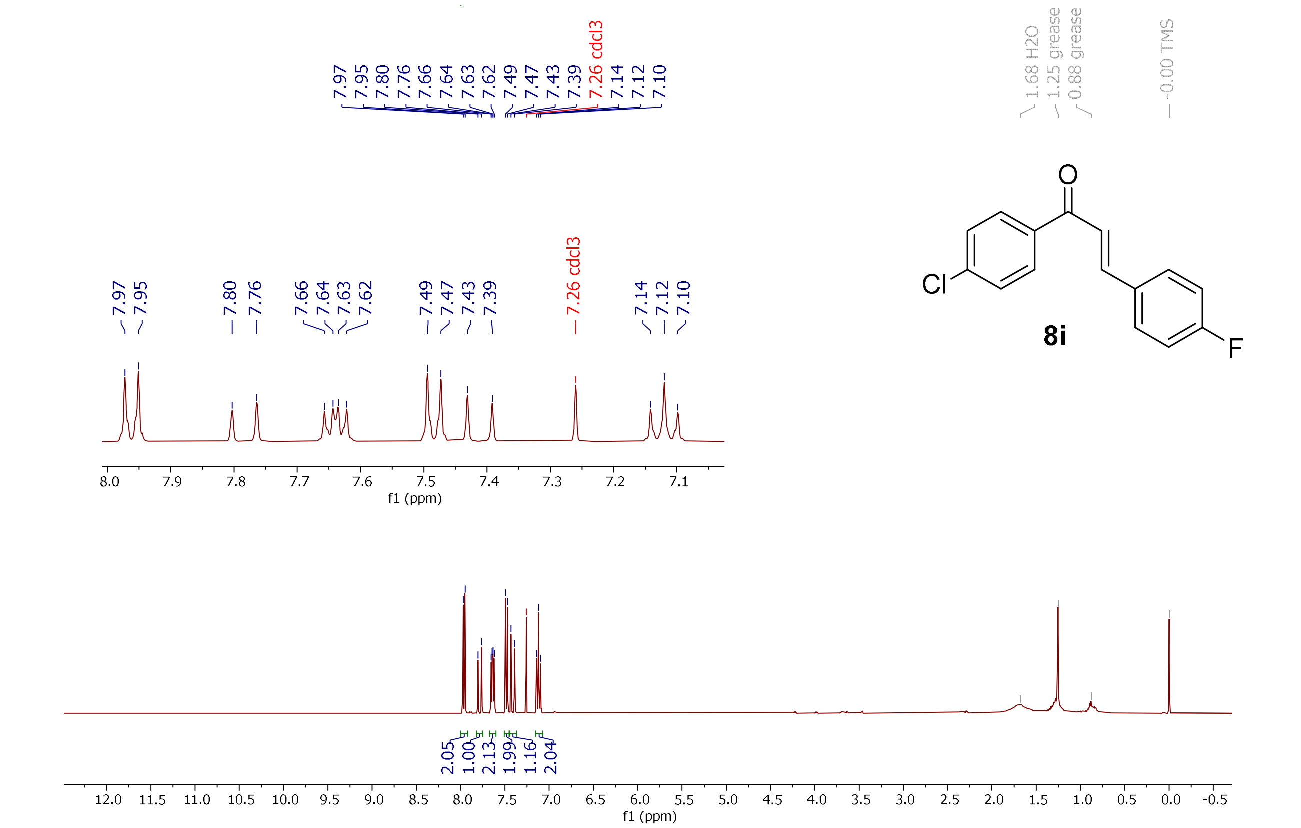

^{13}C{H} NMR (101 MHz, $CDCl_3$) spectrum of 8i

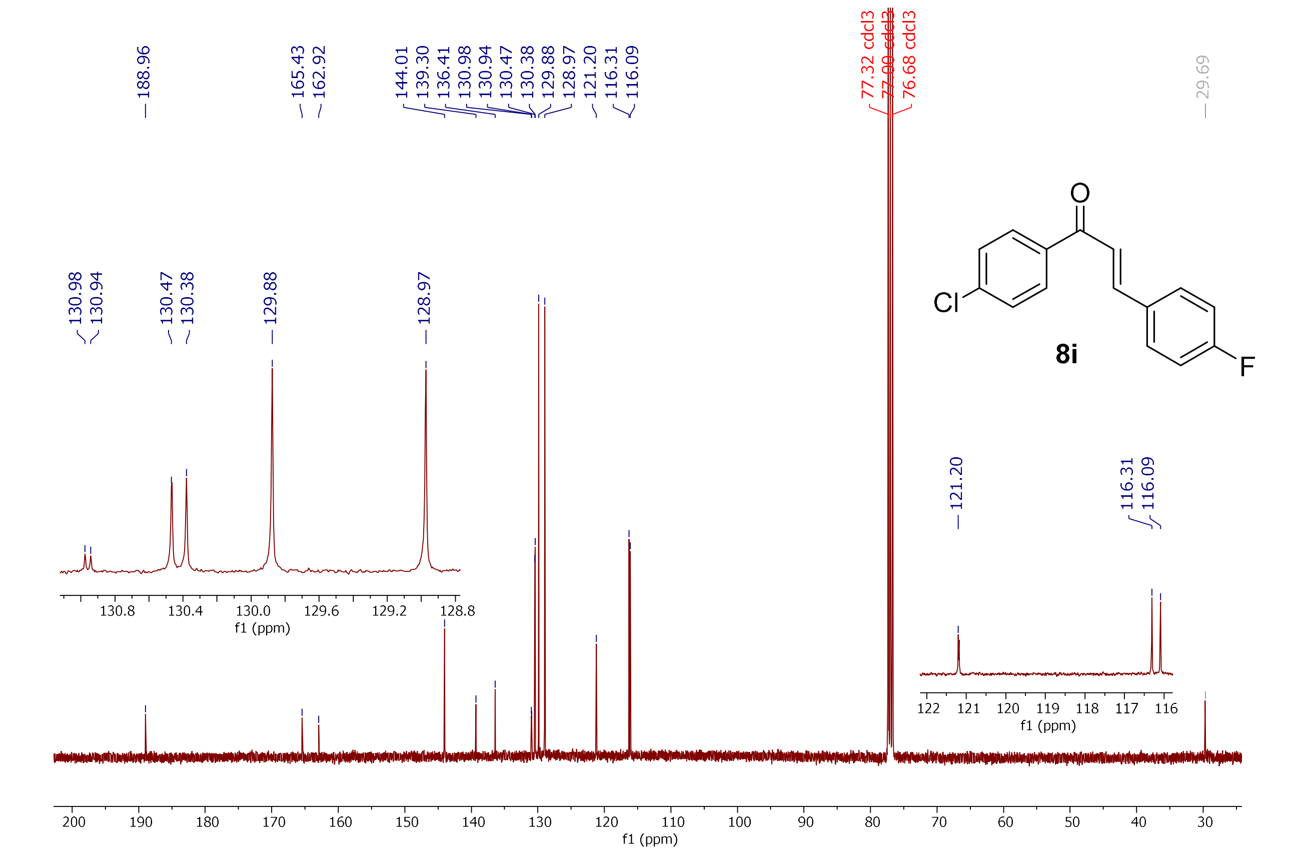

^{1}H NMR (400 MHz, $CDCl_3$) spectrum of 8j

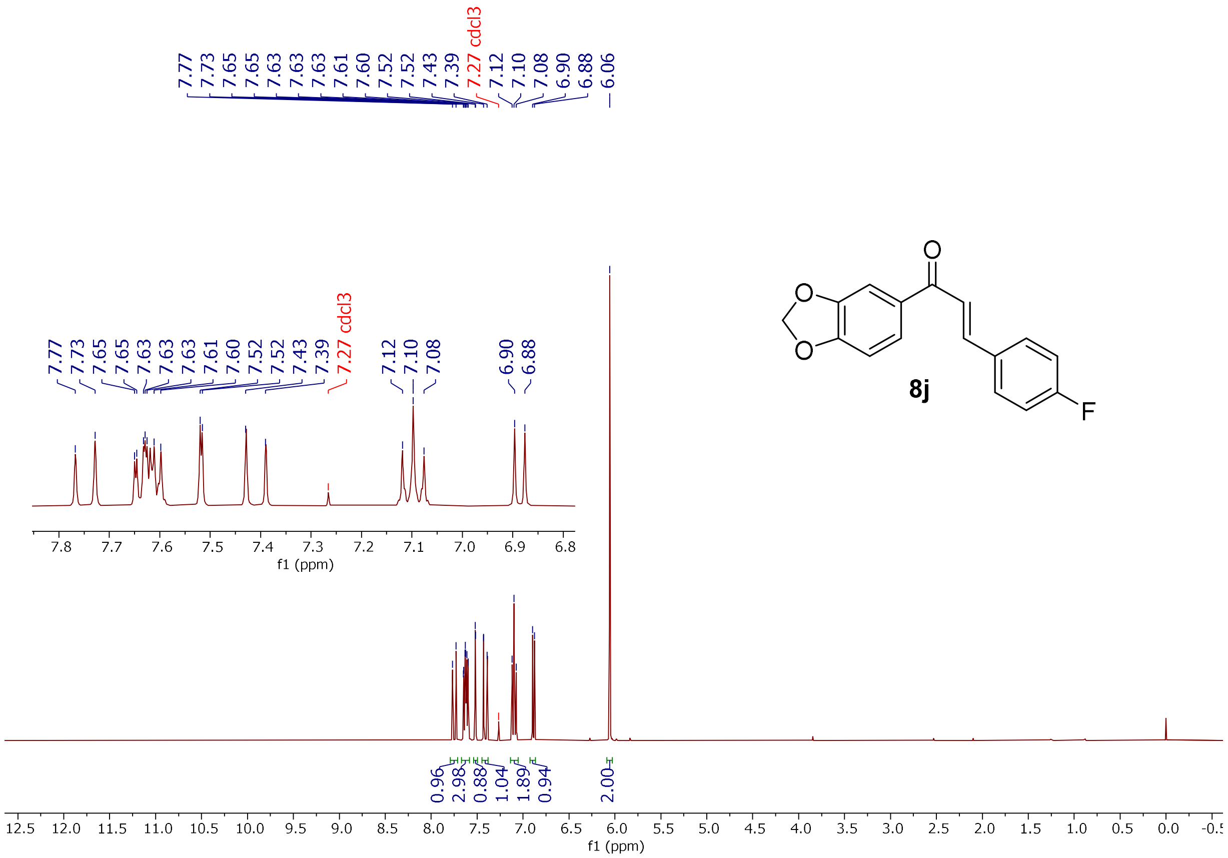

^{13}C{H} NMR (101 MHz, $CDCl_3$) spectrum of 8j

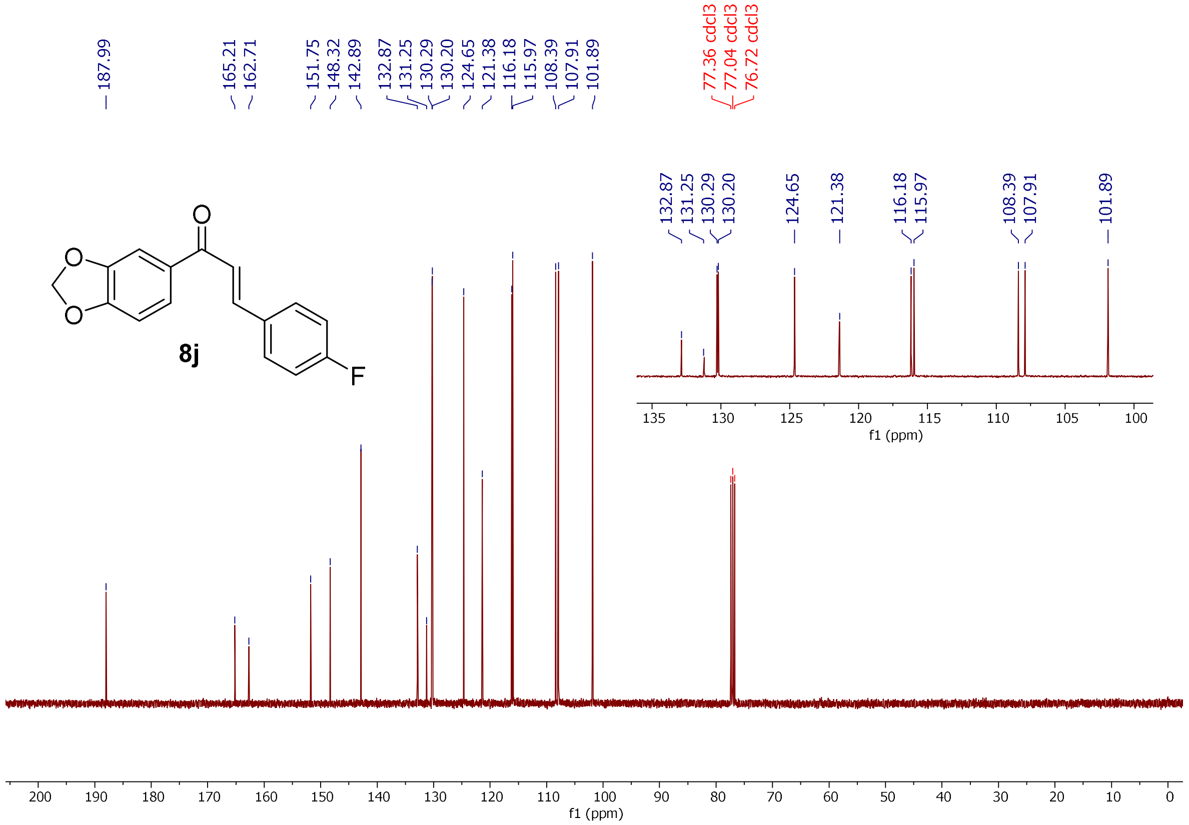

^{1}H NMR (400 MHz, CDCl$_3$) spectrum of 13a

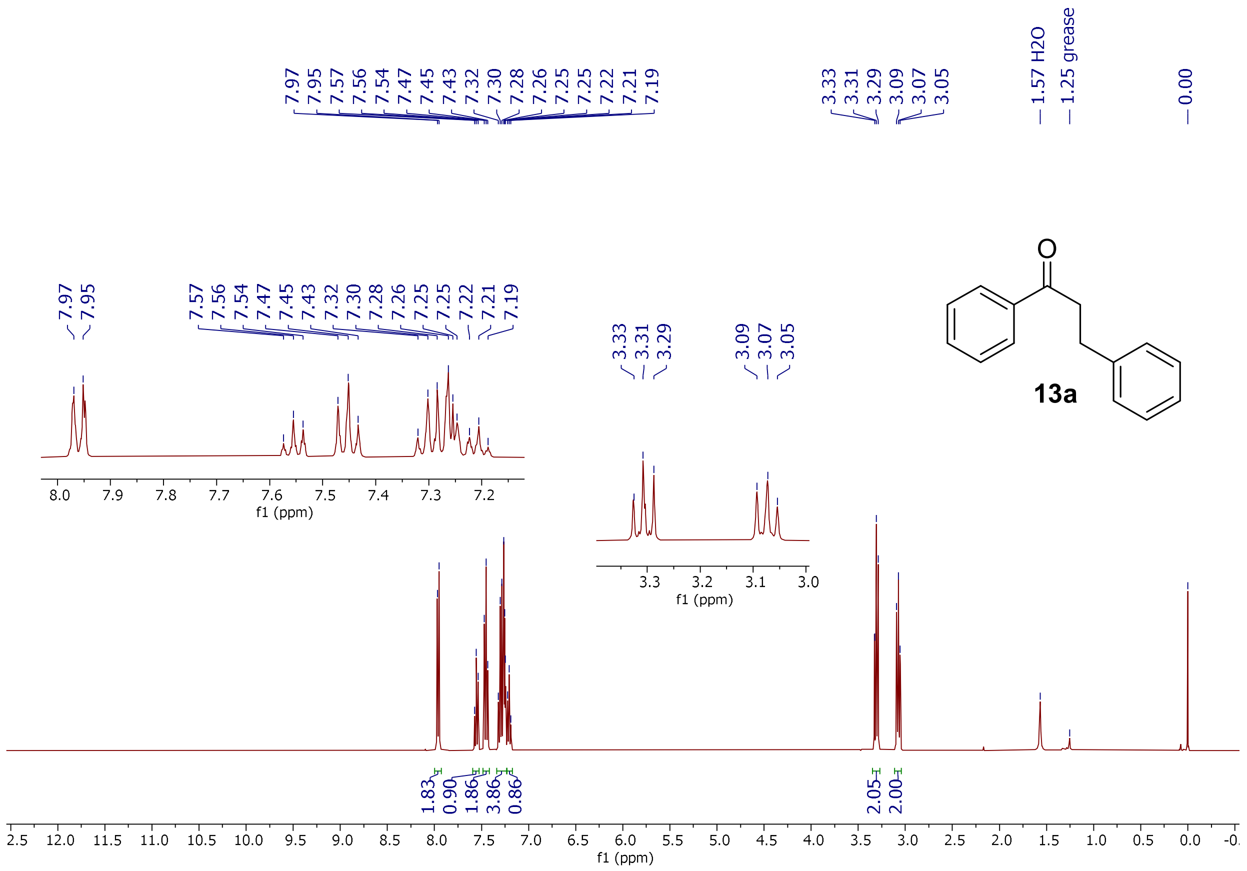

^{13}C{H} NMR (101 MHz, CDCl$_3$) spectrum of 13a

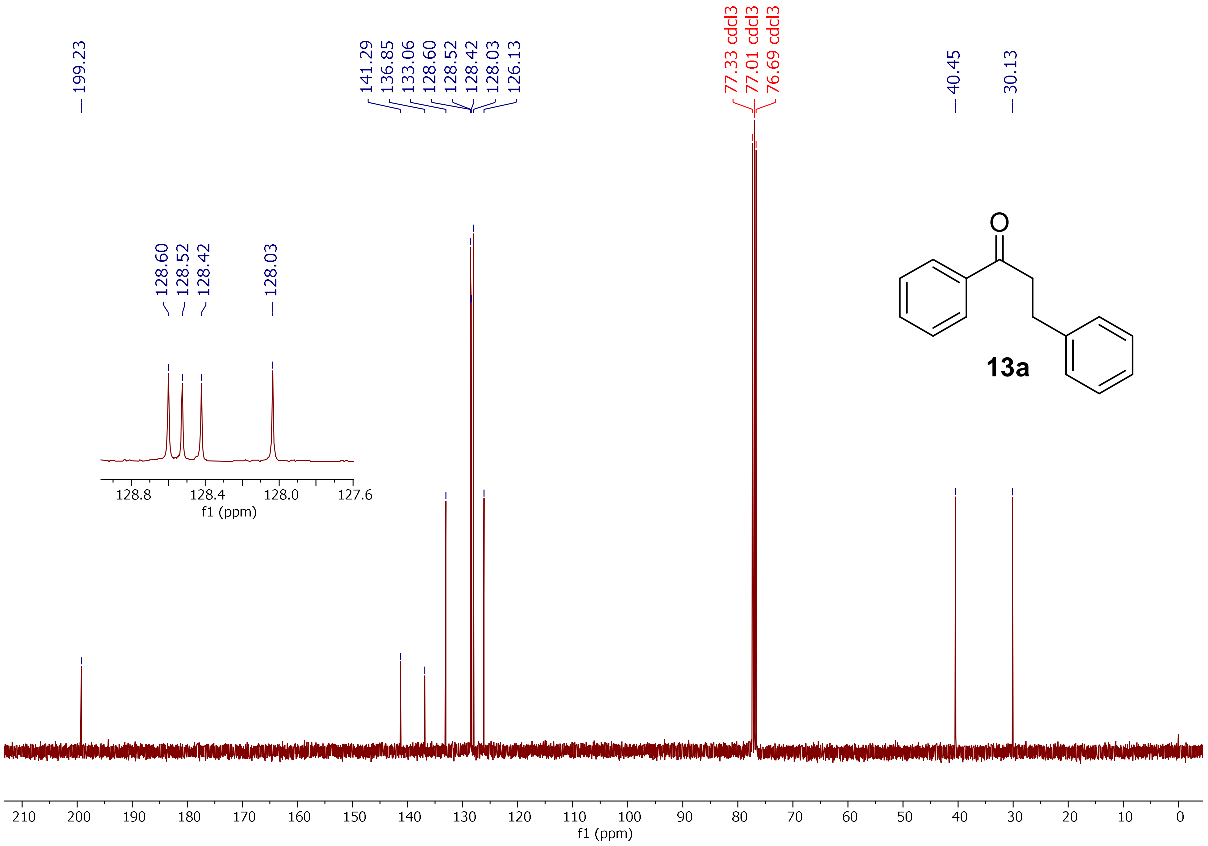

^{1}H NMR (400 MHz, CDCl3) spectrum of 13b

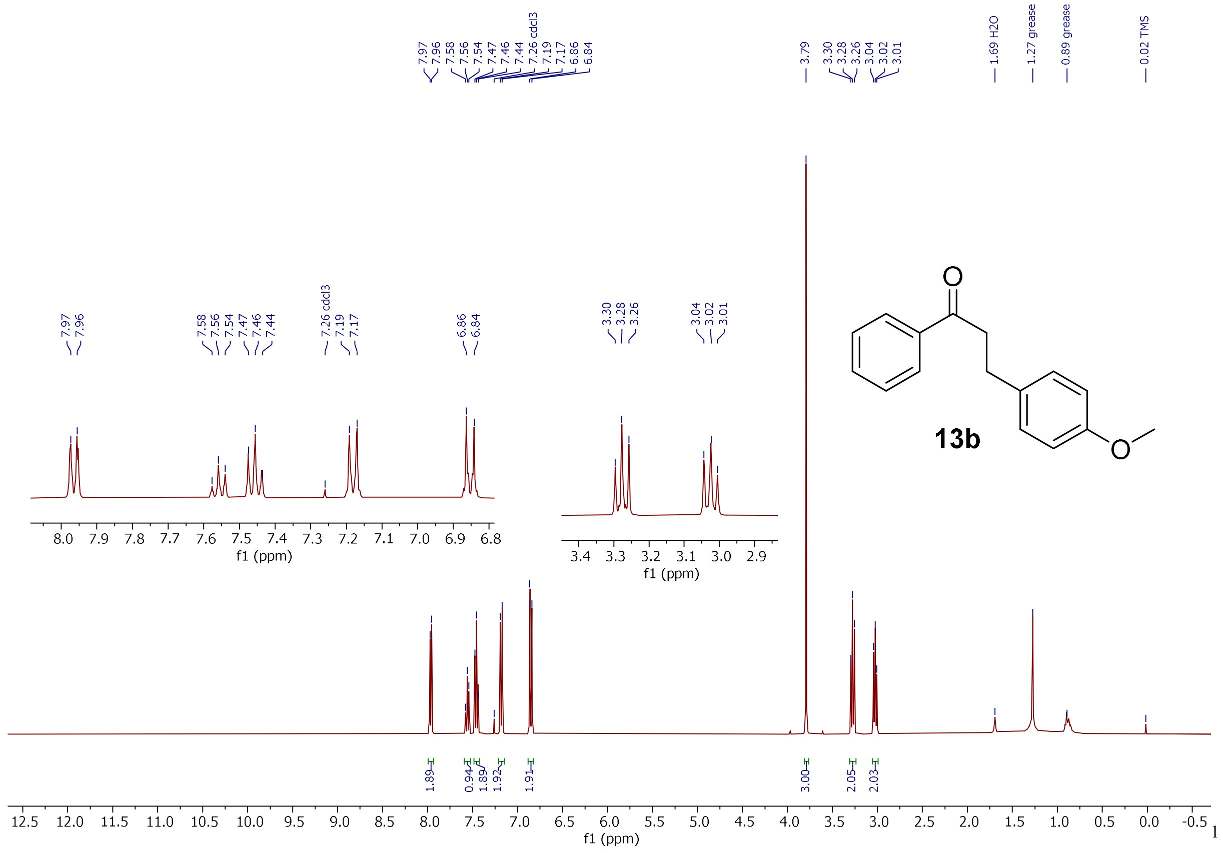

^{13}C{H} NMR (101 MHz, CDCl3) spectrum of 13b

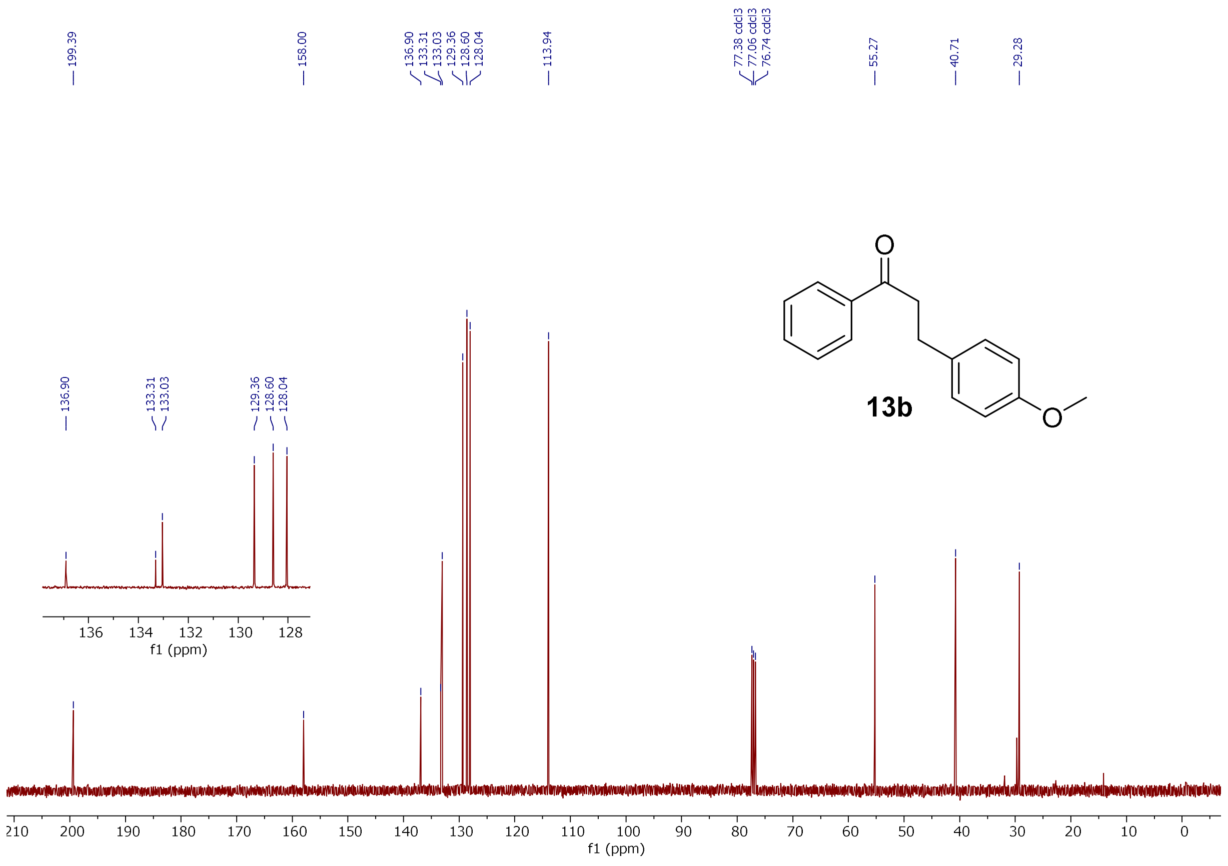

^{1}H NMR (400 MHz, CDCl$_3$) spectrum of 13c

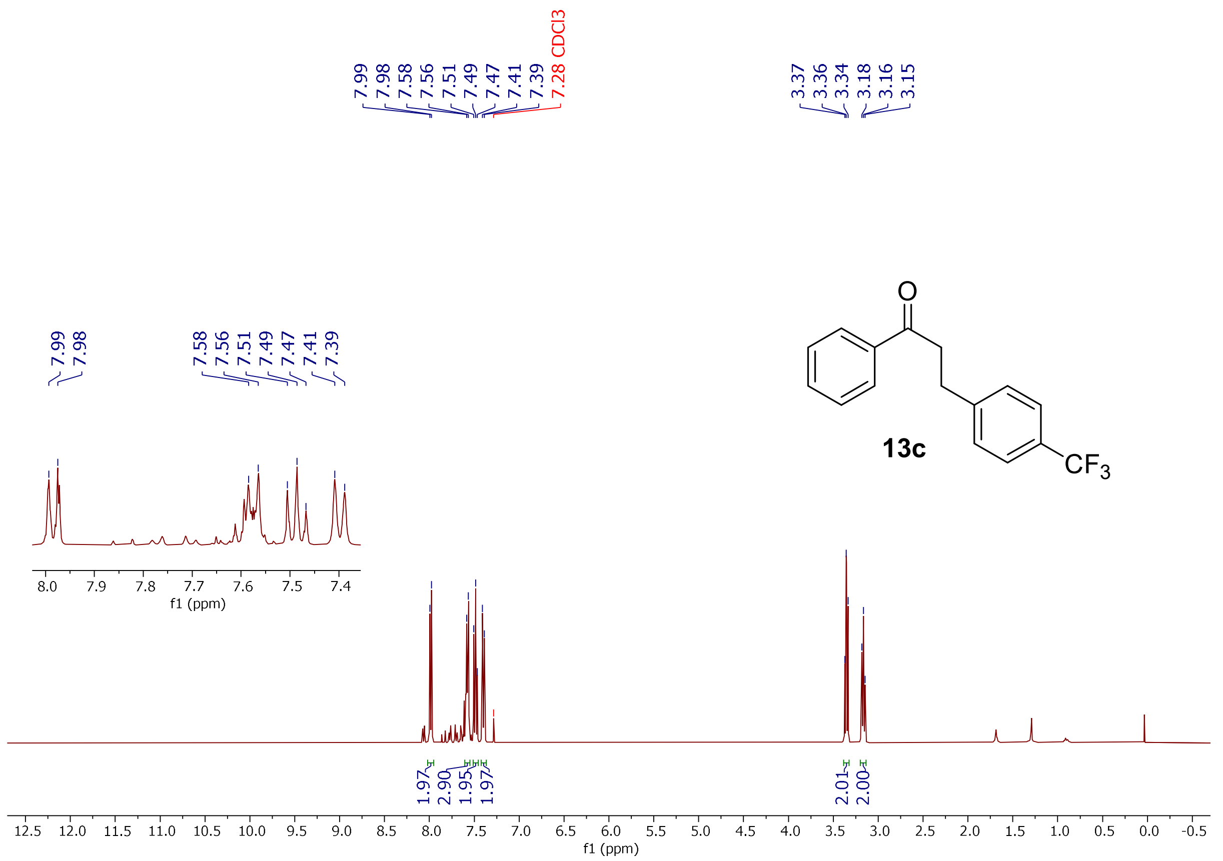

^{13}C{H} NMR (101 MHz, CDCl$_3$) spectrum of 13c

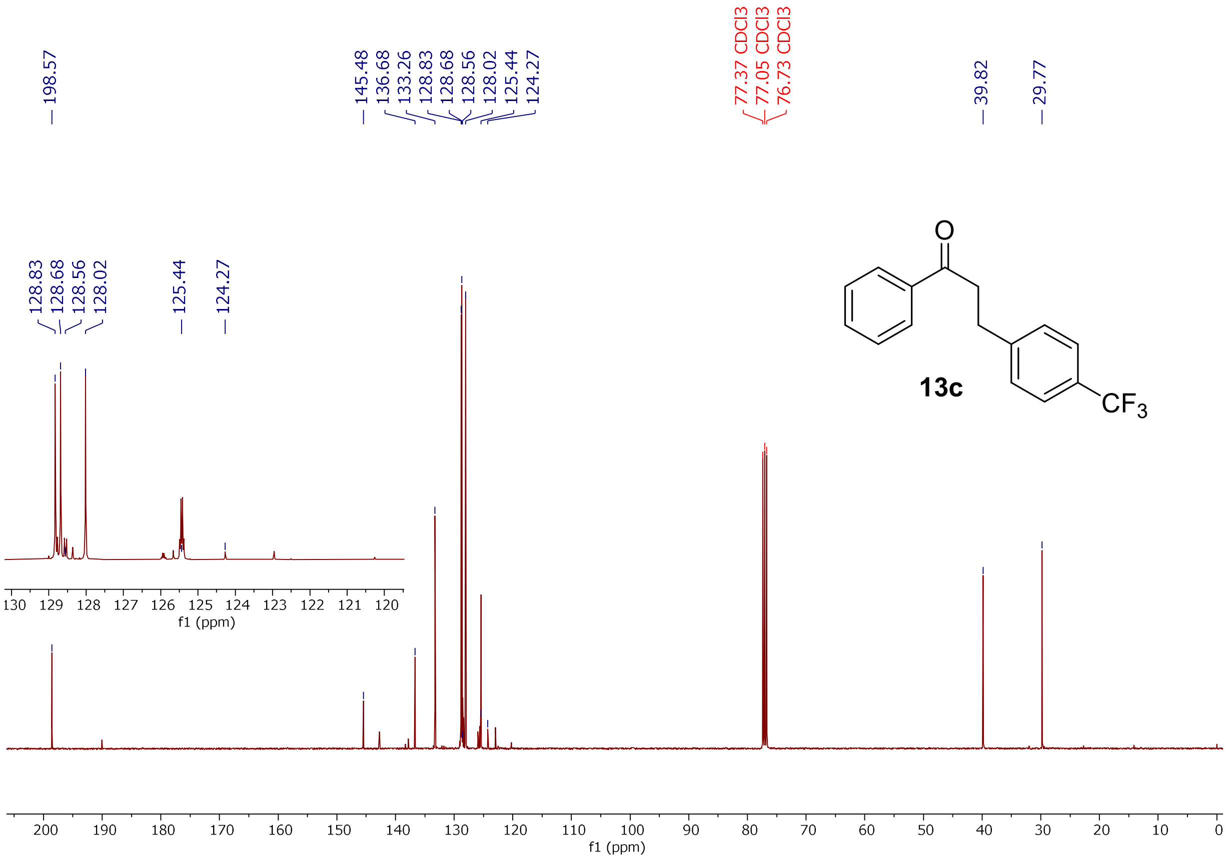

^{1}H NMR (400 MHz, $CDCl_3$) spectrum of 13d

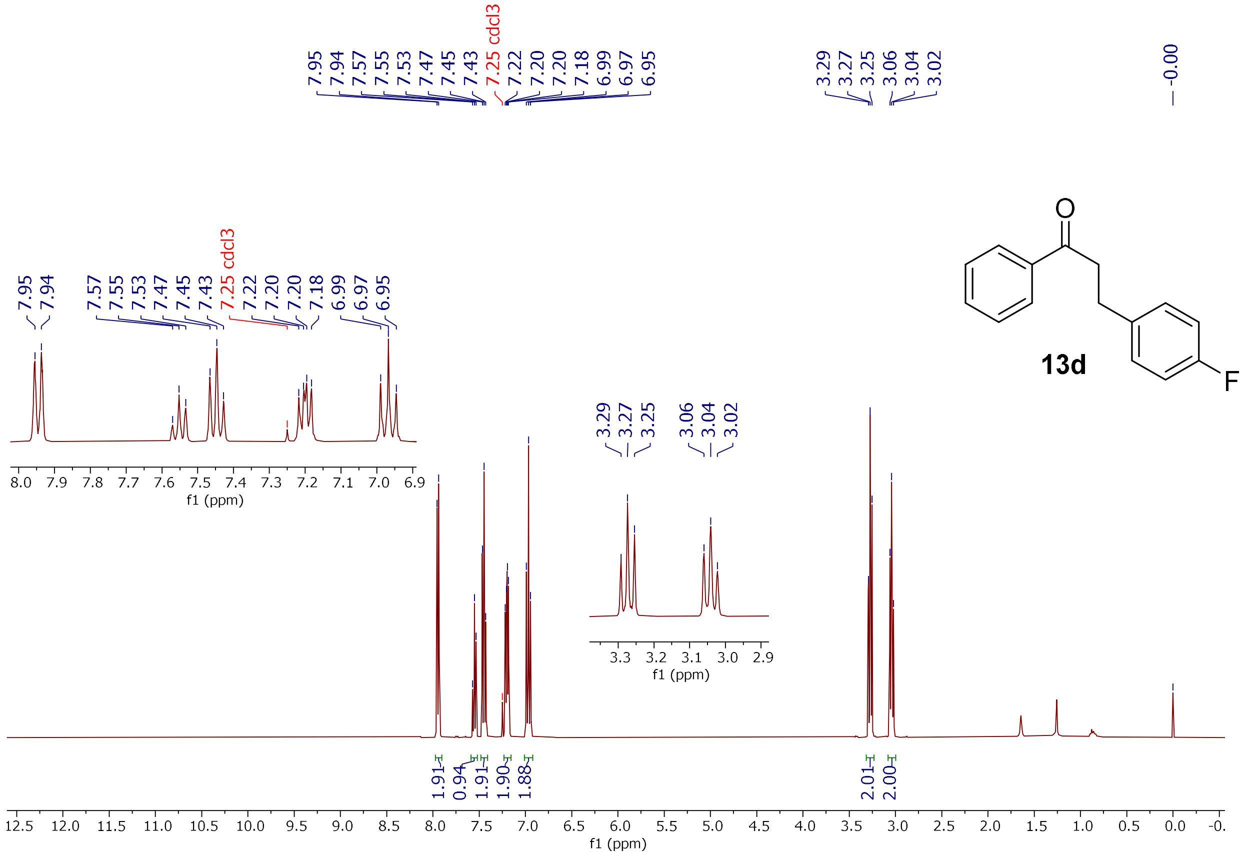

^{13}C{H} NMR (101 MHz, $CDCl_3$) spectrum of 13d

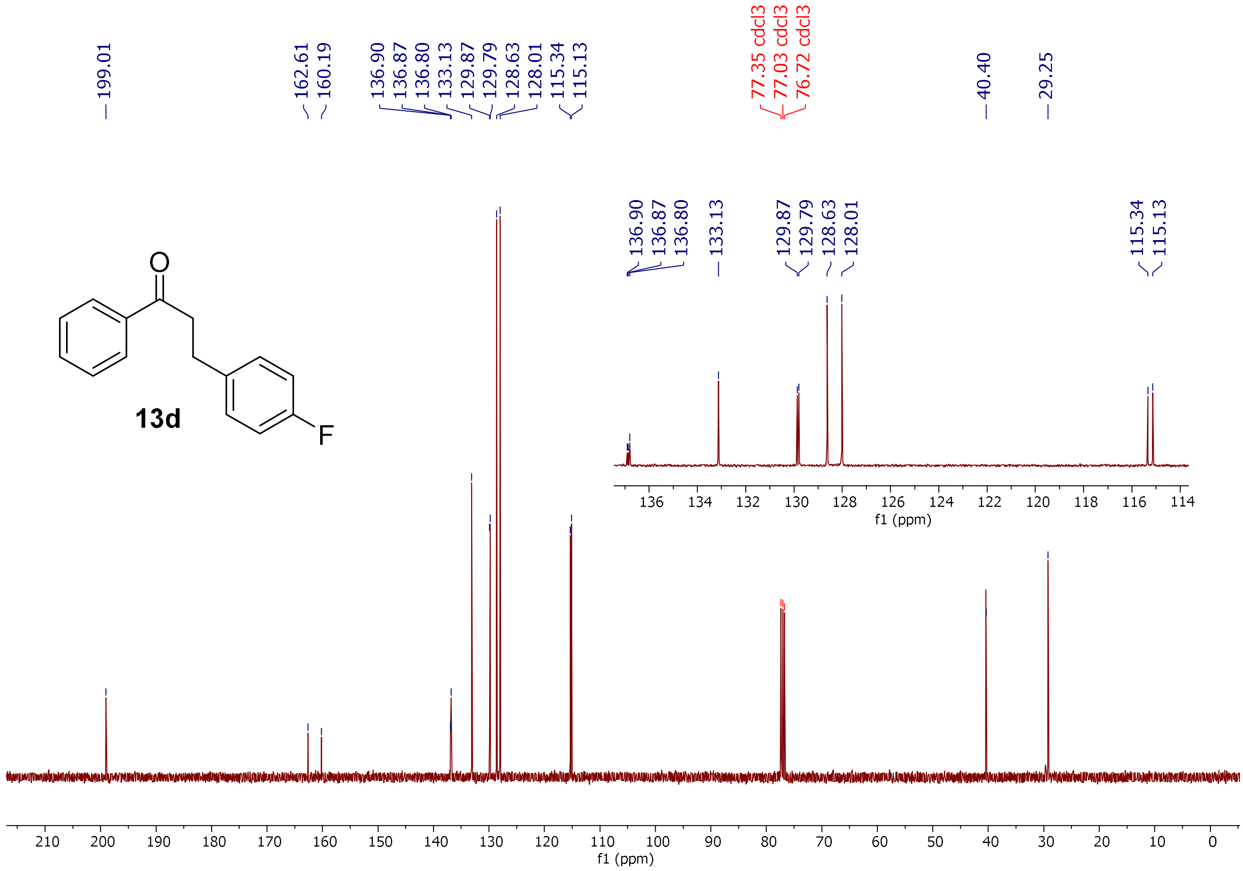

^{1}H NMR (400 MHz, $CDCl_3$) spectrum of 13e

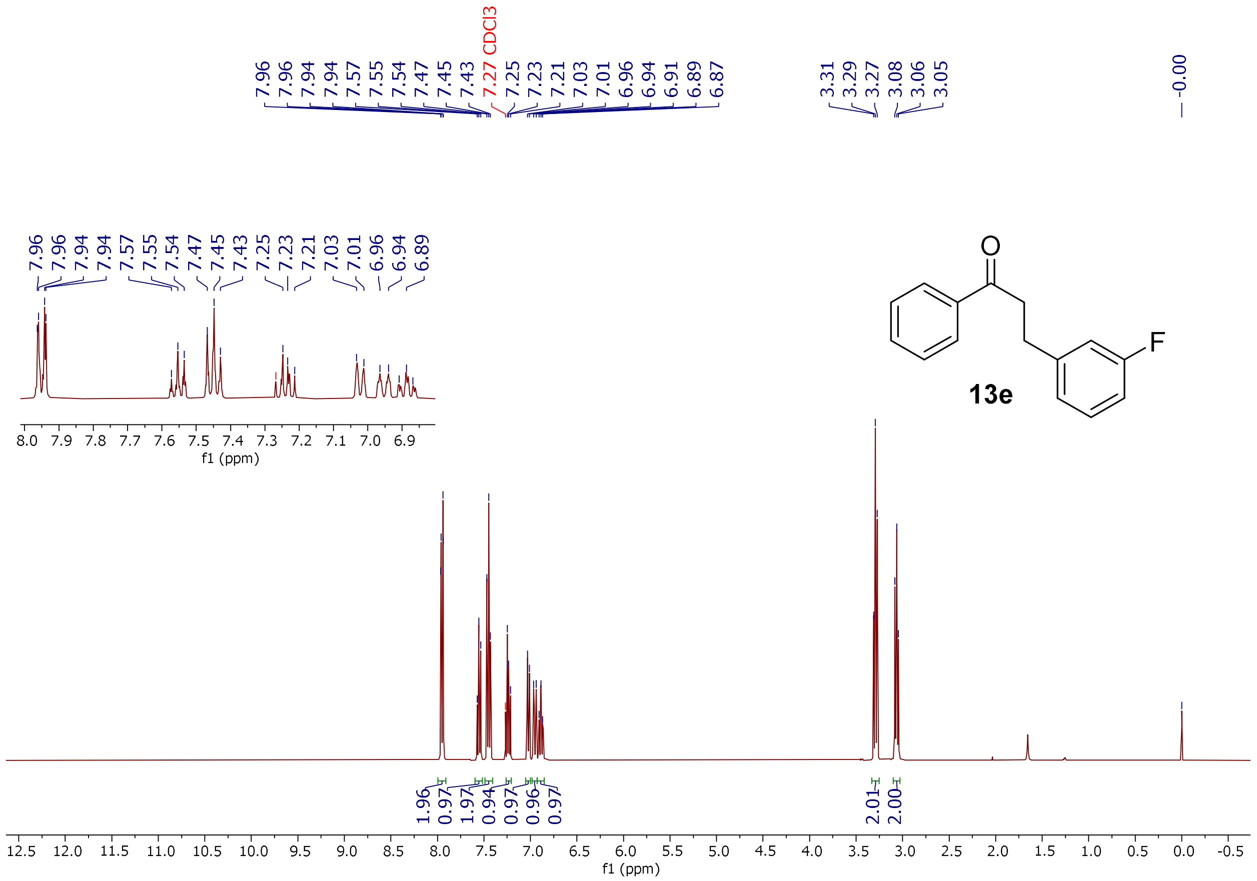

^{13}C{H} NMR (101 MHz, $CDCl_3$) spectrum of 13e

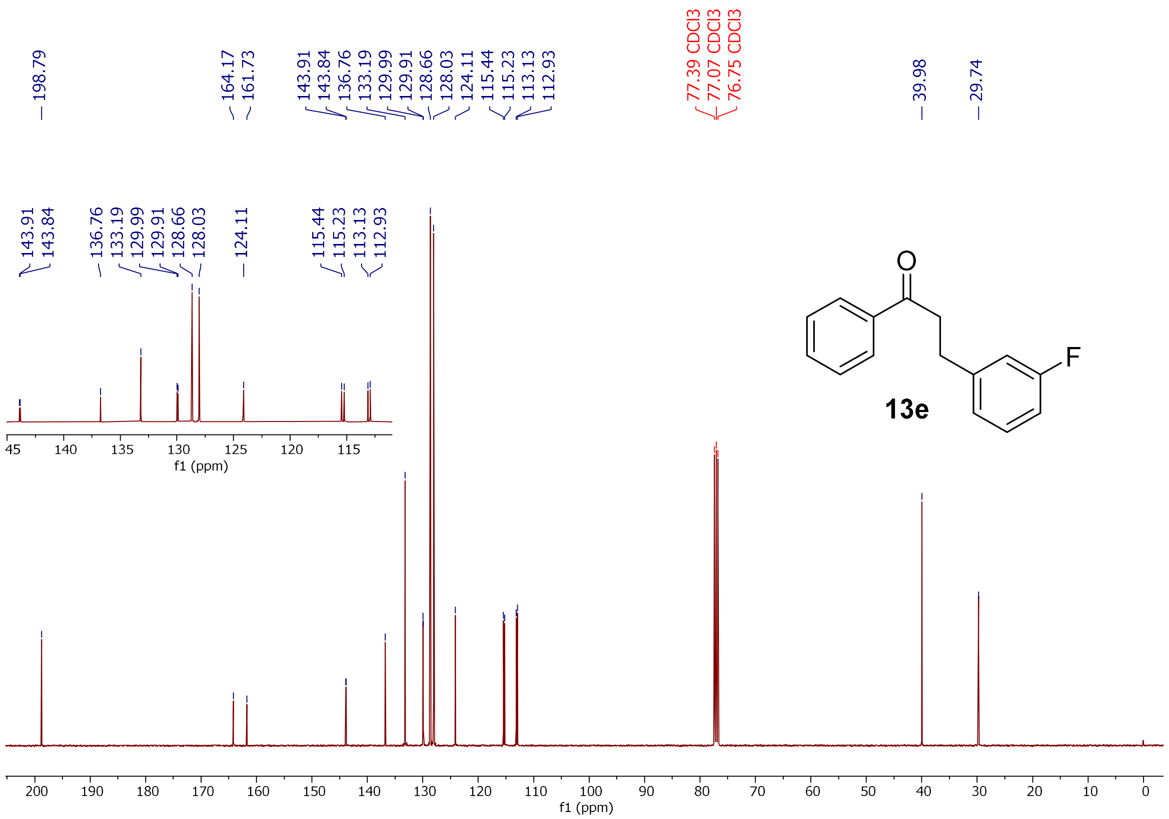

^{1}H NMR (500 MHz, $CDCl_3$) spectrum of 13f

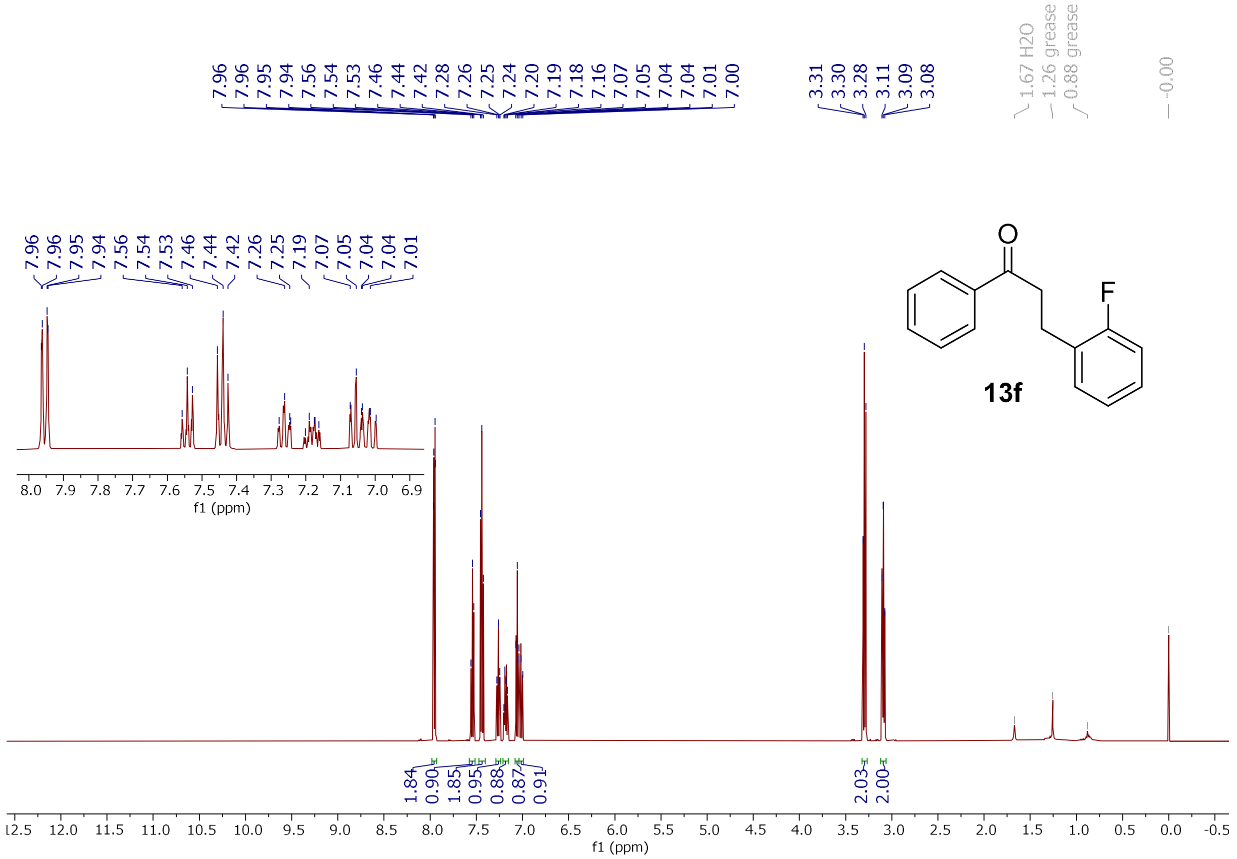

$^{13}C\{H\}$ NMR (126 MHz, $CDCl_3$) spectrum of 13f

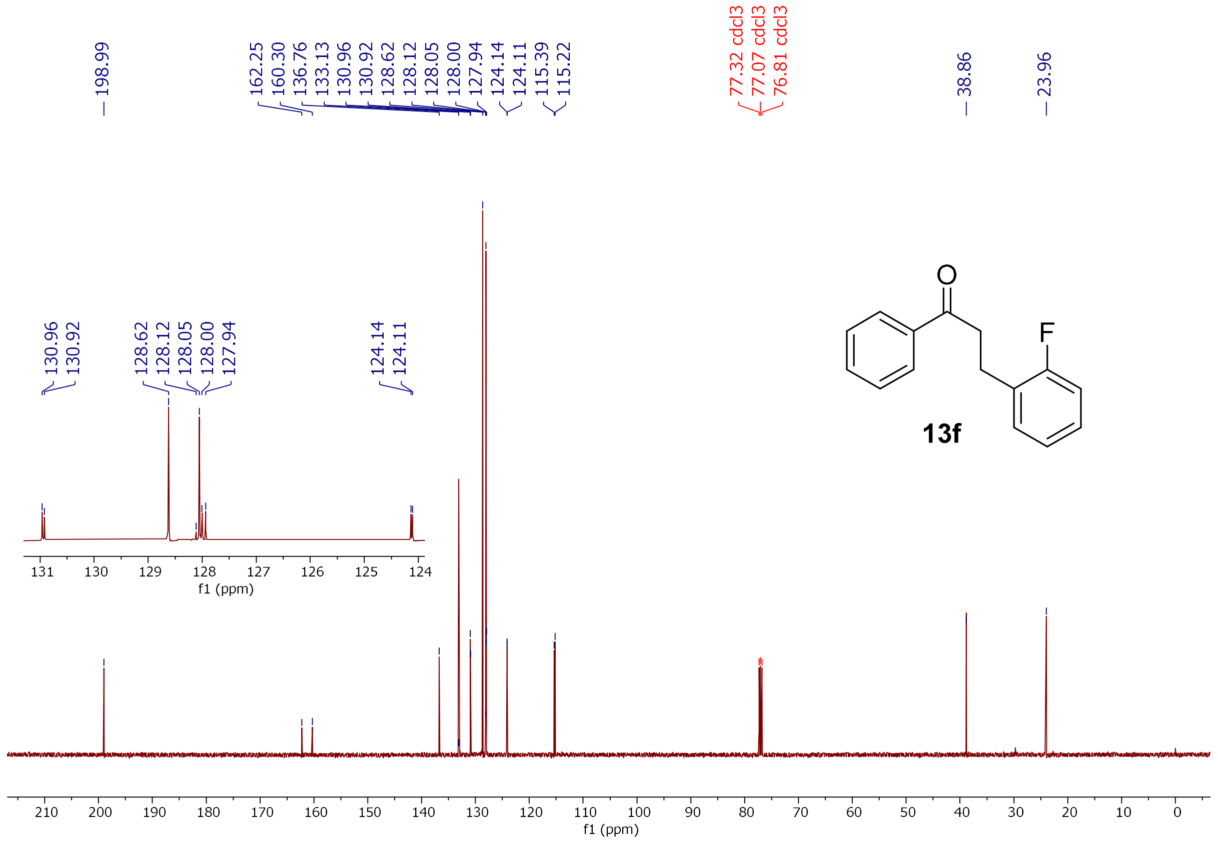

^{1}H NMR (500 MHz, $CDCl_3$) spectrum of 13g

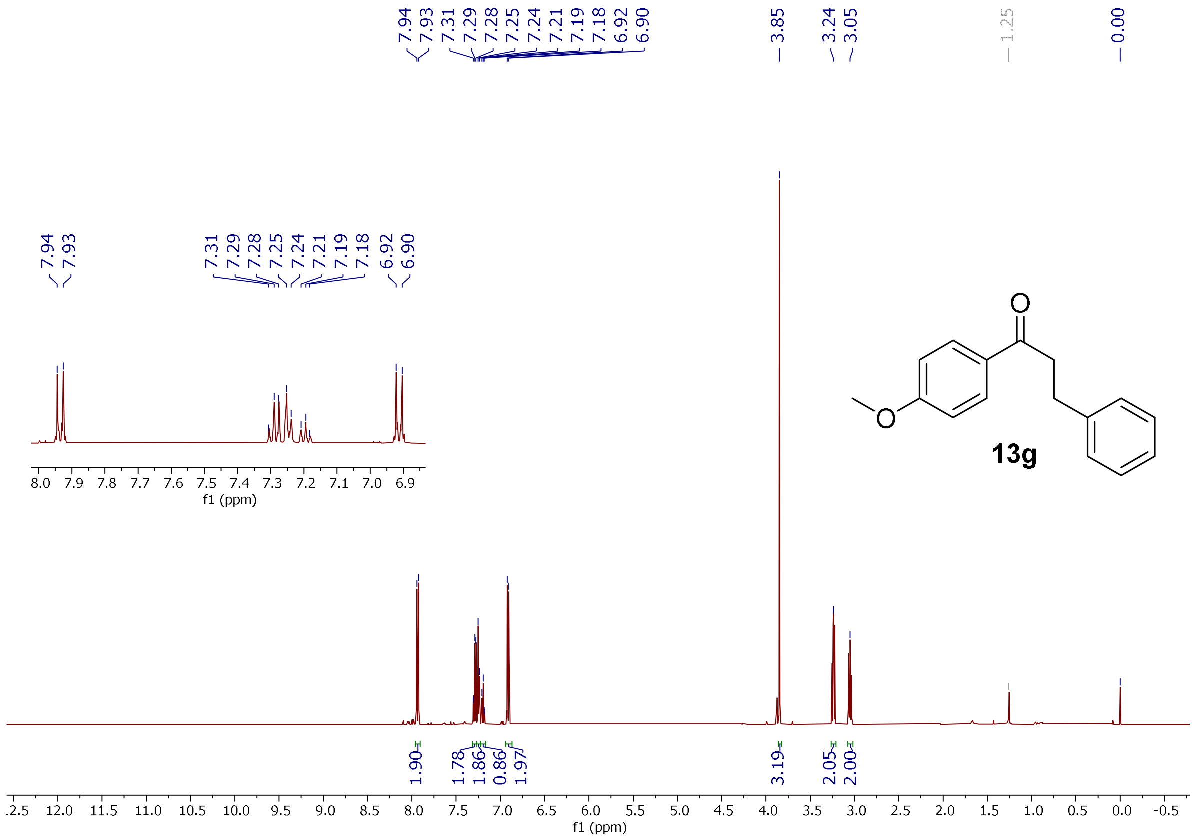

^{13}C{H} NMR (126 MHz, $CDCl_3$) spectrum of 13g

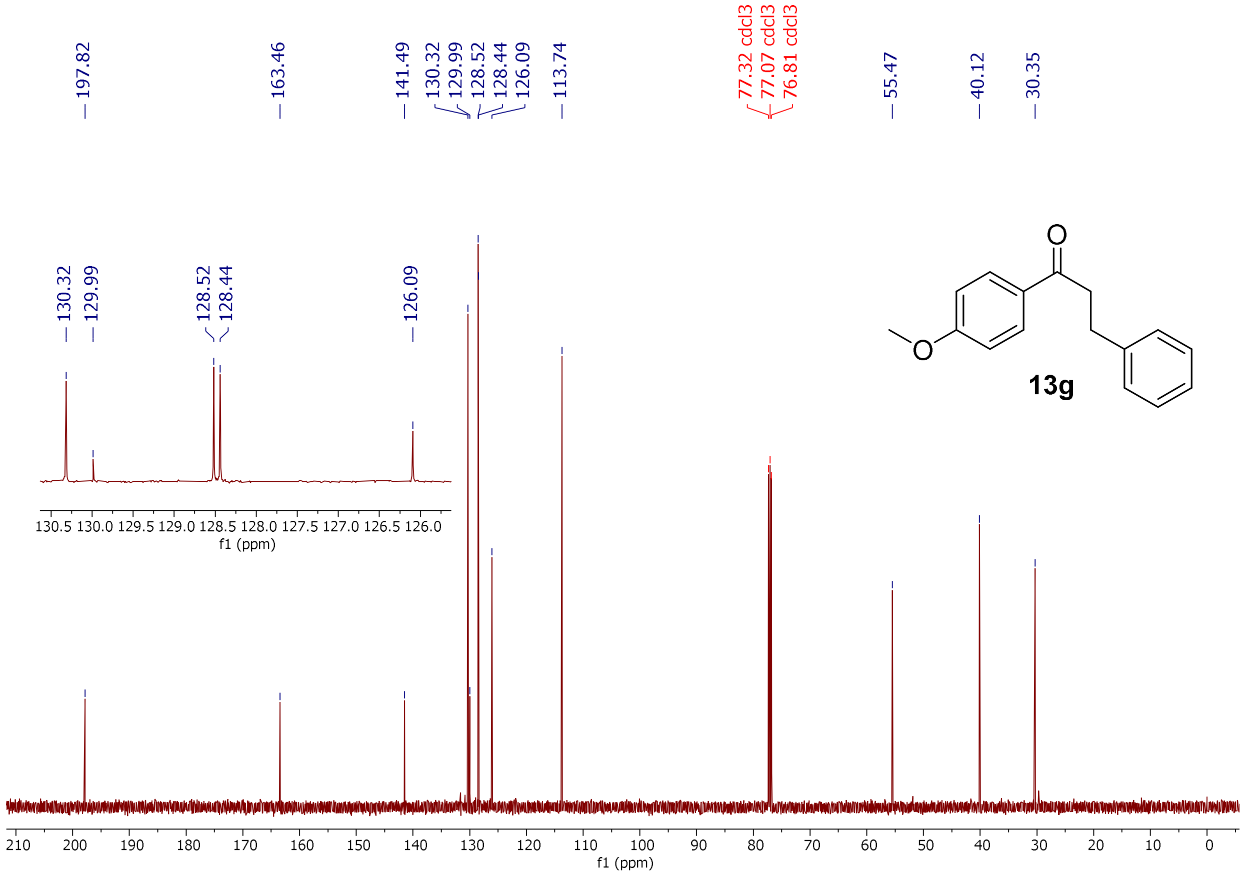

^{1}H NMR (500 MHz, $CDCl_3$) spectrum of 13h

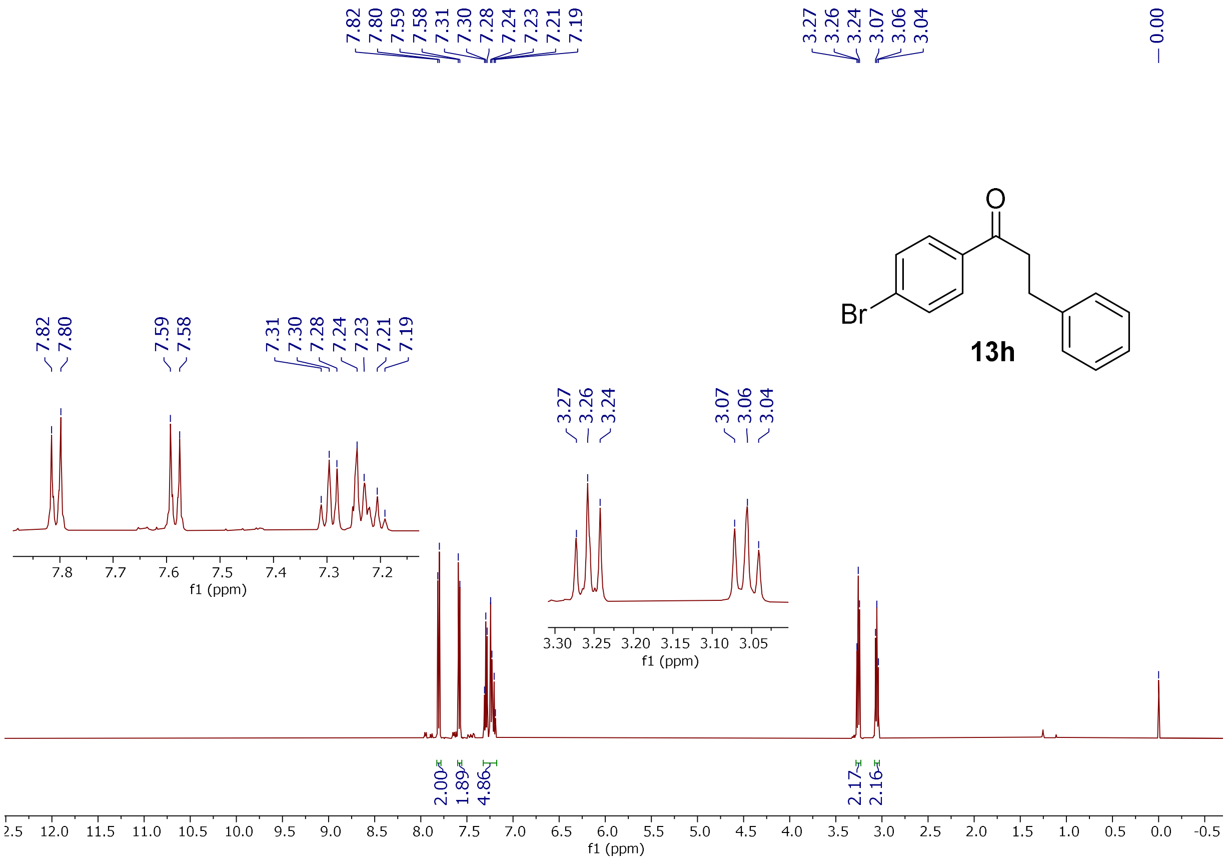

^{13}C{H} NMR (126 MHz, $CDCl_3$) spectrum of 13h

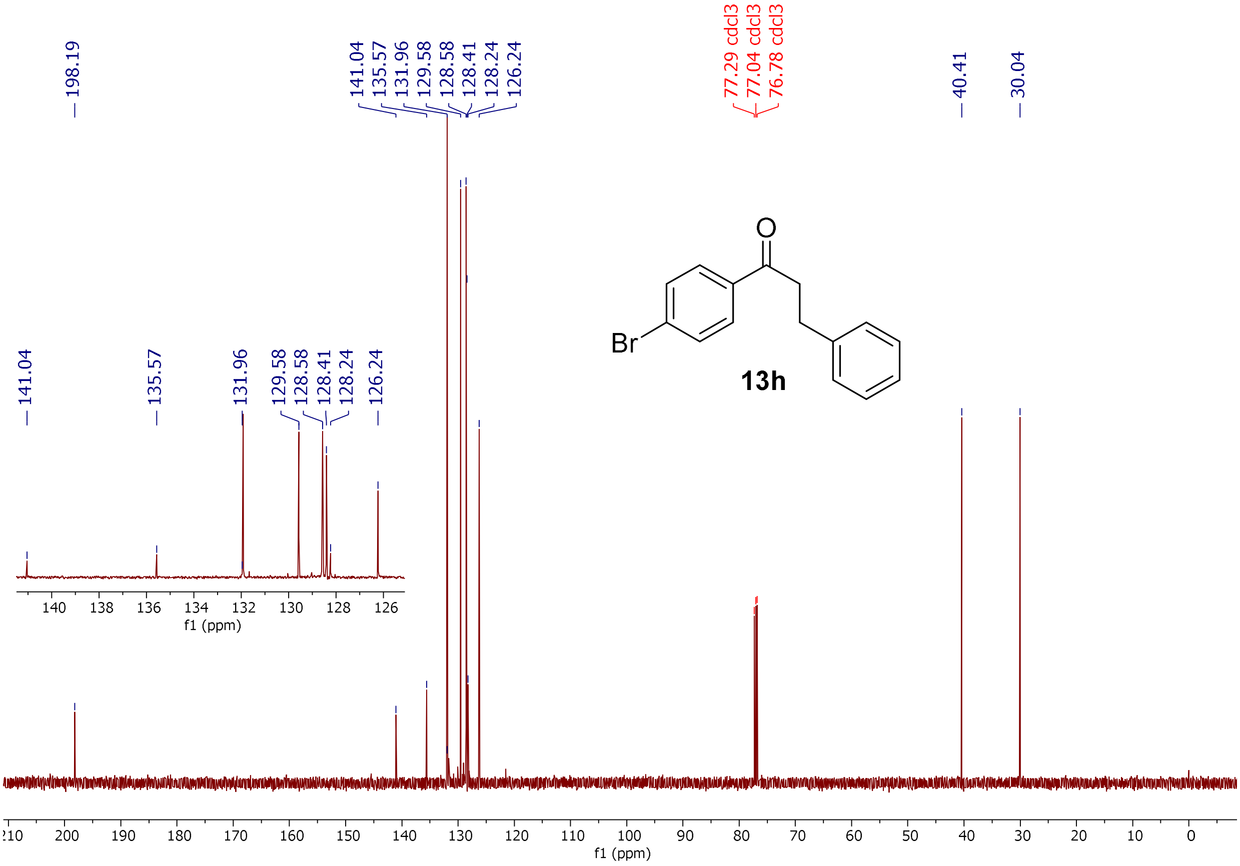

^{1}H NMR (400 MHz, CDCl3) spectrum of 13i

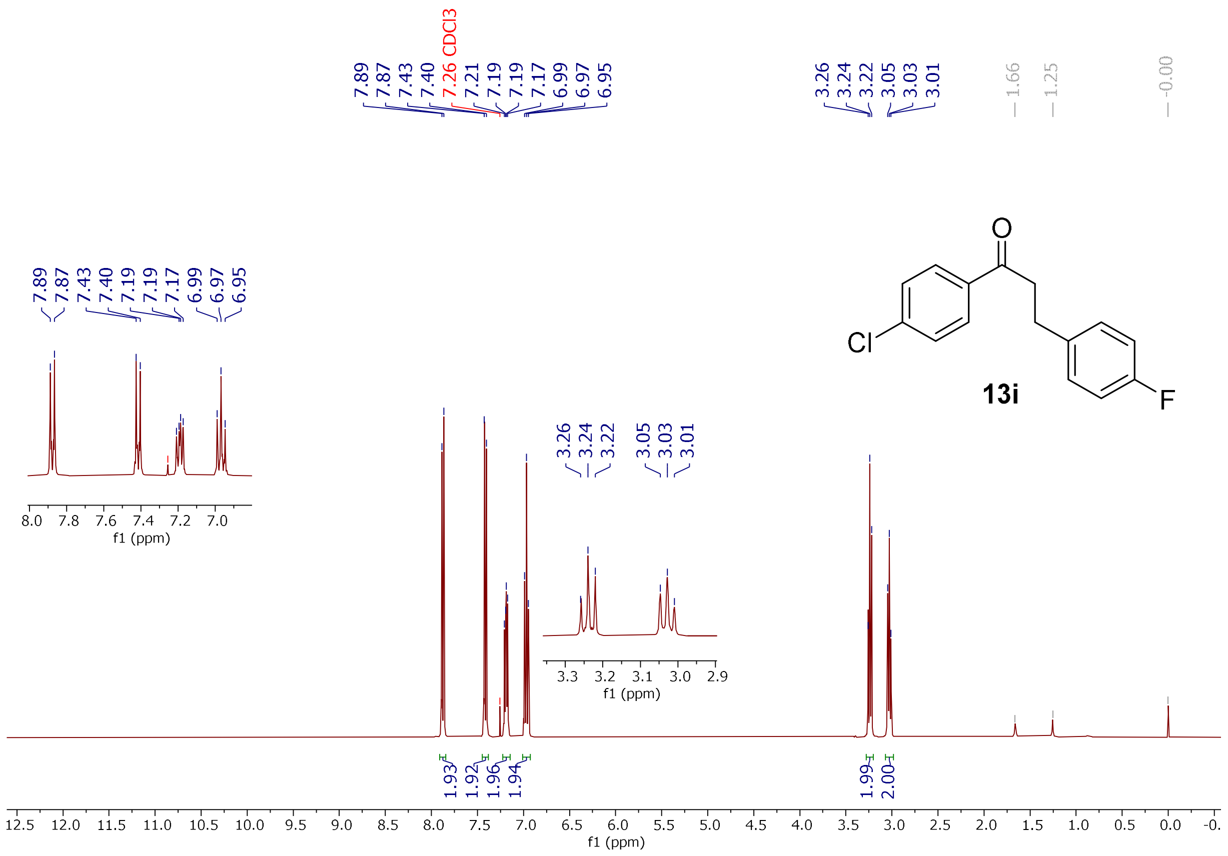

^{13}C{H} NMR (101 MHz, CDCl3) spectrum of 13i

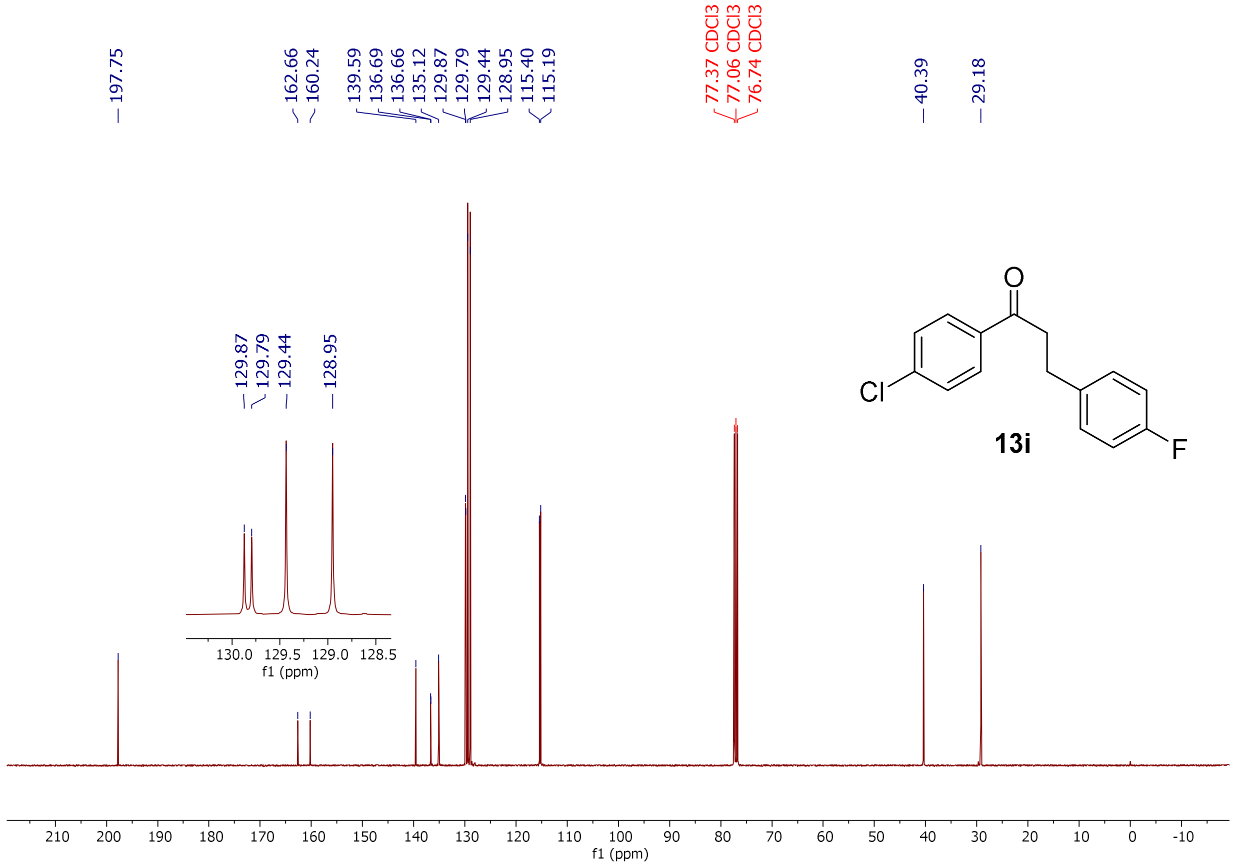

^{1}H NMR (400 MHz, $CDCl_3$) spectrum of 13j

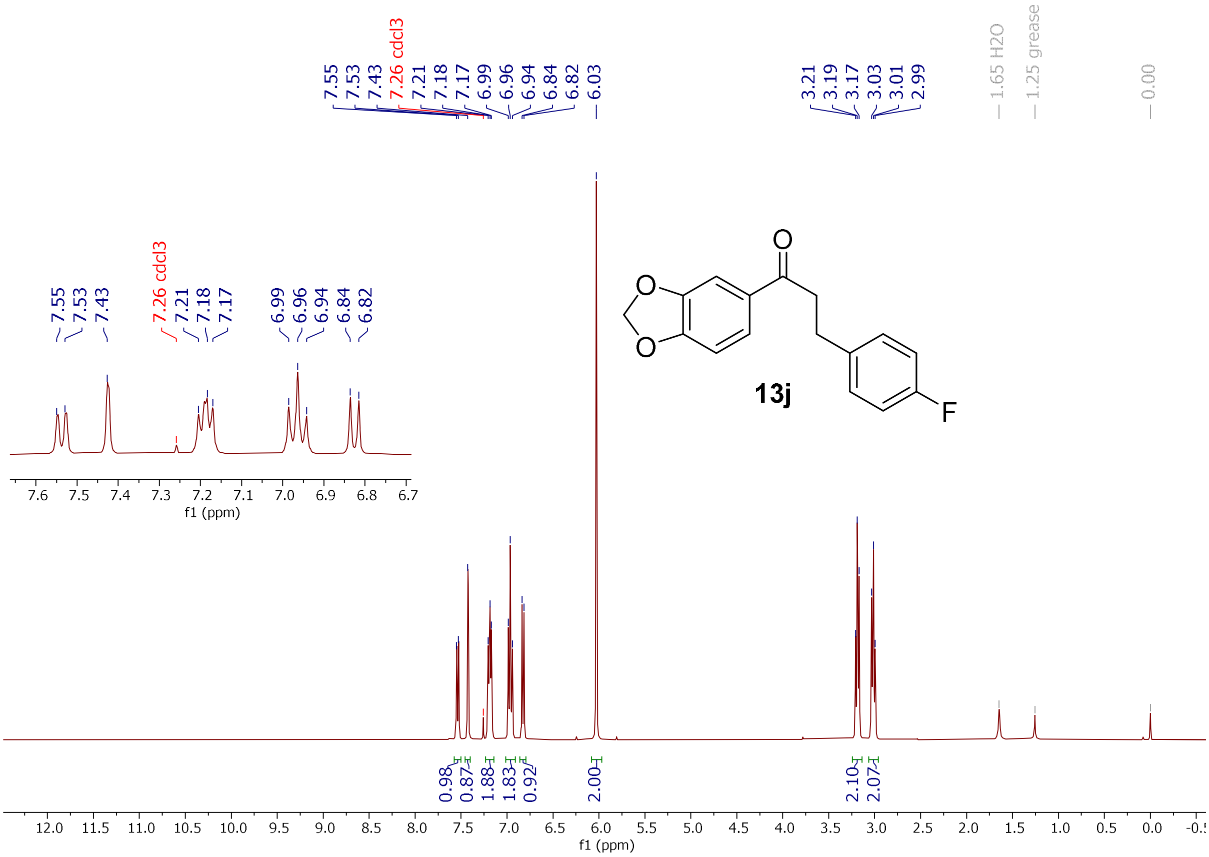

^{13}C{H} NMR (101 MHz, $CDCl_3$) spectrum of 13j

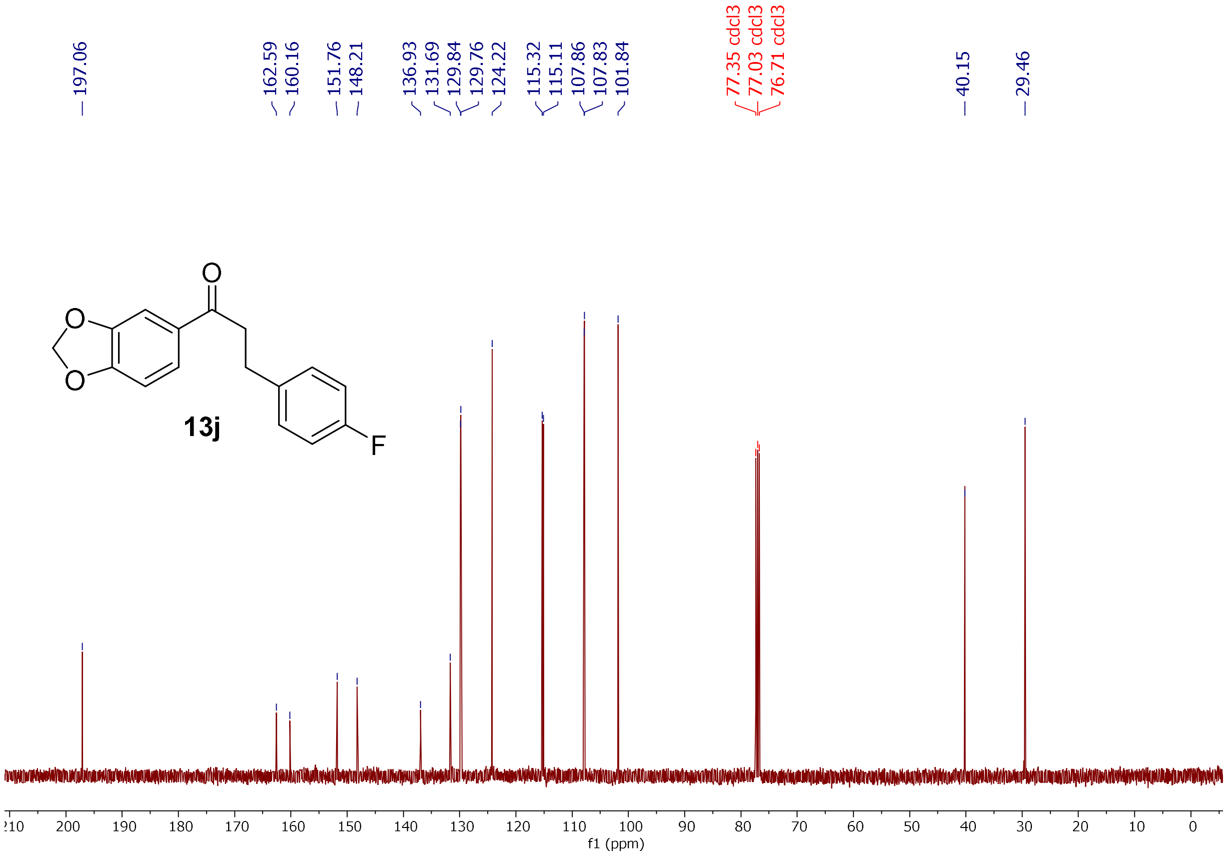

^{1}H NMR (500 MHz, CDCl$_3$) spectrum of 10a/15a

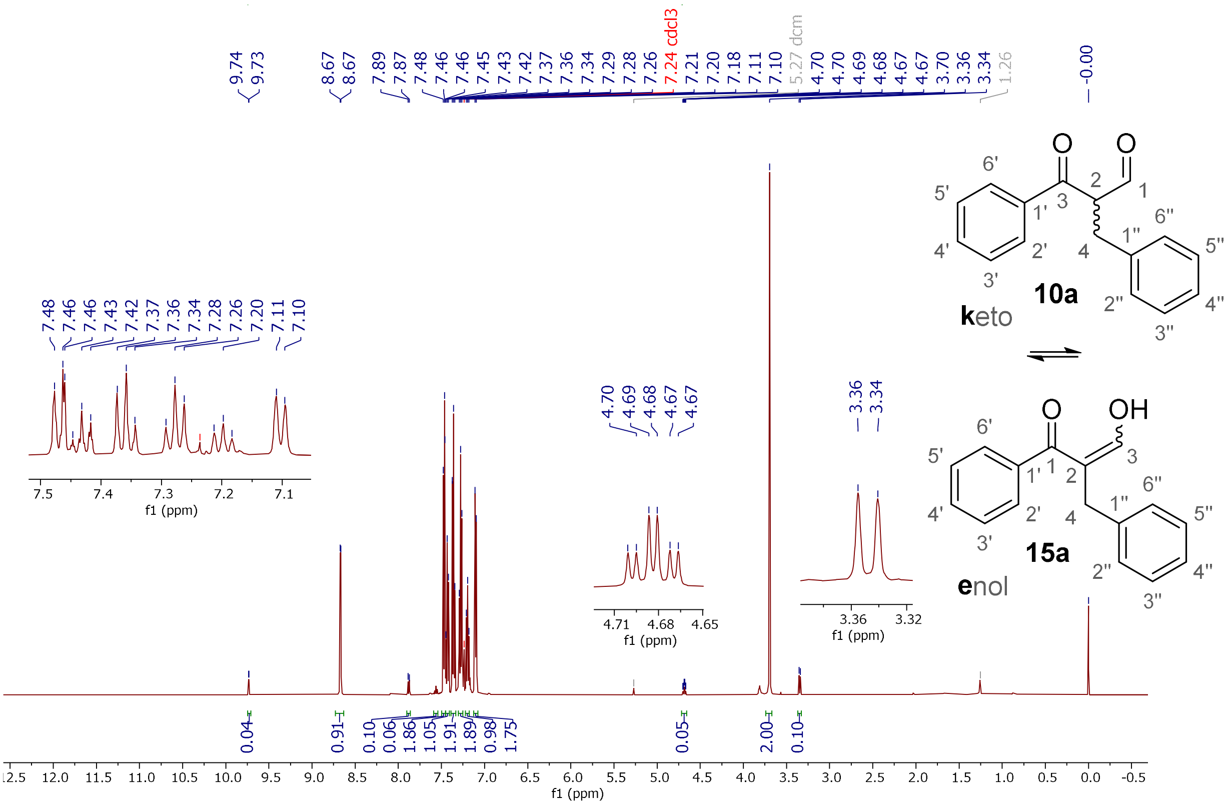

^{1}H NMR (500 MHz, CDCl$_3$, **mixture of tautomers**) δ 9.73 (d, *J* = 2.8 Hz, 1H, H1-K), 8.67 (d, *J* = 4.0 Hz, 1H, H3-E), 7.90 – 7.10 (m, 20H, H6'-K, H5'-K, H4'-K, H3'-K, H2'-K, H6''-K, H5''-K, H4''-K, H3''-K, H2''-K, H6'-E, H5'-E, H4'-E, H3'-E, H2'-E, H6''-E, H5''-E, H4''-E, H3''-E, H2''-E), 4.68 (td, *J* = 7.2, 2.9 Hz, 1H, H2-K), 3.70 (s, 2H, H4-E), 3.35 (d, *J* = 7.2 Hz, 2H, H4-K). Tautomeric ratio: 96:4 (enol/aldehyde).

^{13}C{H} NMR (126 MHz, CDCl$_3$) spectrum of 15a

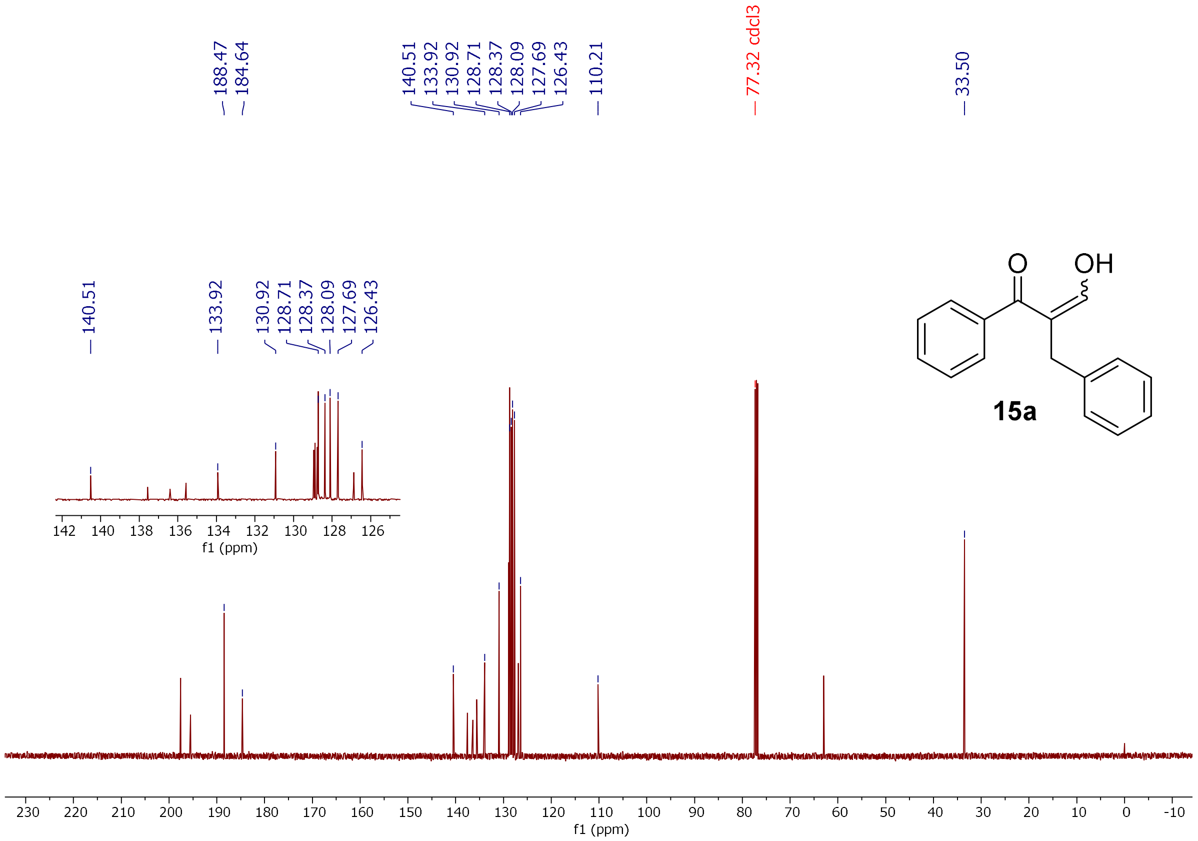

^{13}C NMR (126 MHz, CDCl$_3$) δ 188.5, 184.6, 140.5, 133.9, 130.9, 128.7, 128.4, 128.1, 127.7, 126.4, 110.2, 33.5 ***(only the enol was identified)***.

^{1}H NMR (500 MHz, $CDCl_3$) spectrum of 10b/15b

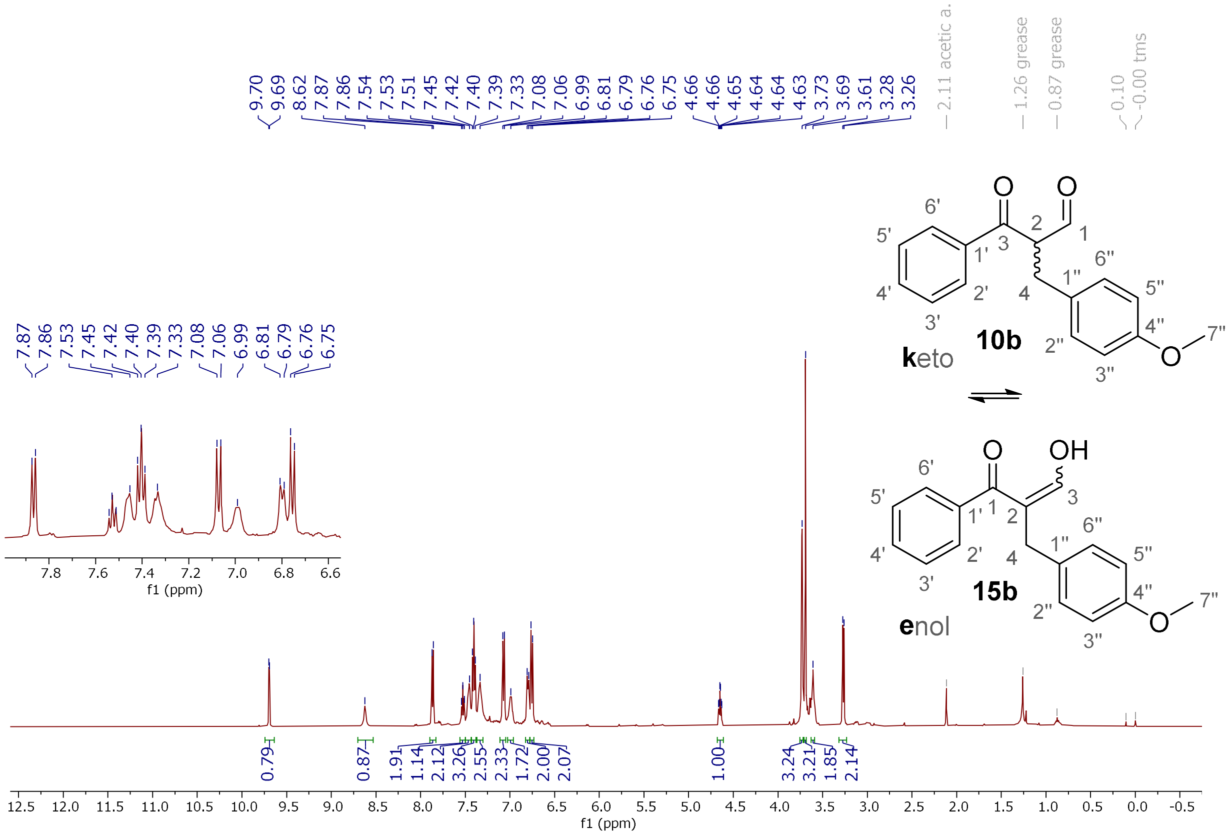

^{1}H NMR (500 MHz, $CDCl_3$, **mixture of tautomers**) δ 9.69 (d, J = 2.8 Hz, 1H, H1-K), 8.62 (s, 1H, H3-E), 7.90 – 6.75 (m, 18H, H6'-K, H5'-K, H4'-K, H3'-K, H2'-K, H6''-K, H5''-K, H3''-K, H2''-K, H6'-E, H5'-E, H4'-E, H3'-E, H2'-E, H6''-E, H5''-E, H3''-E, H2''-E), 4.65 (td, J = 7.2, 2.8 Hz, 1H, H2-K), 3.73 (s, 3H, H7"-E), 3.69 (s, 3H, H7"-K), 3.61 (s, 2H, H4-E), 3.27 (d, J = 7.1 Hz, 2H, H4-K). Tautomeric ratio: 52:48 (enol/aldehyde).

$^{13}C\{H\}$ NMR (126 MHz, $CDCl_3$) spectrum of 10b

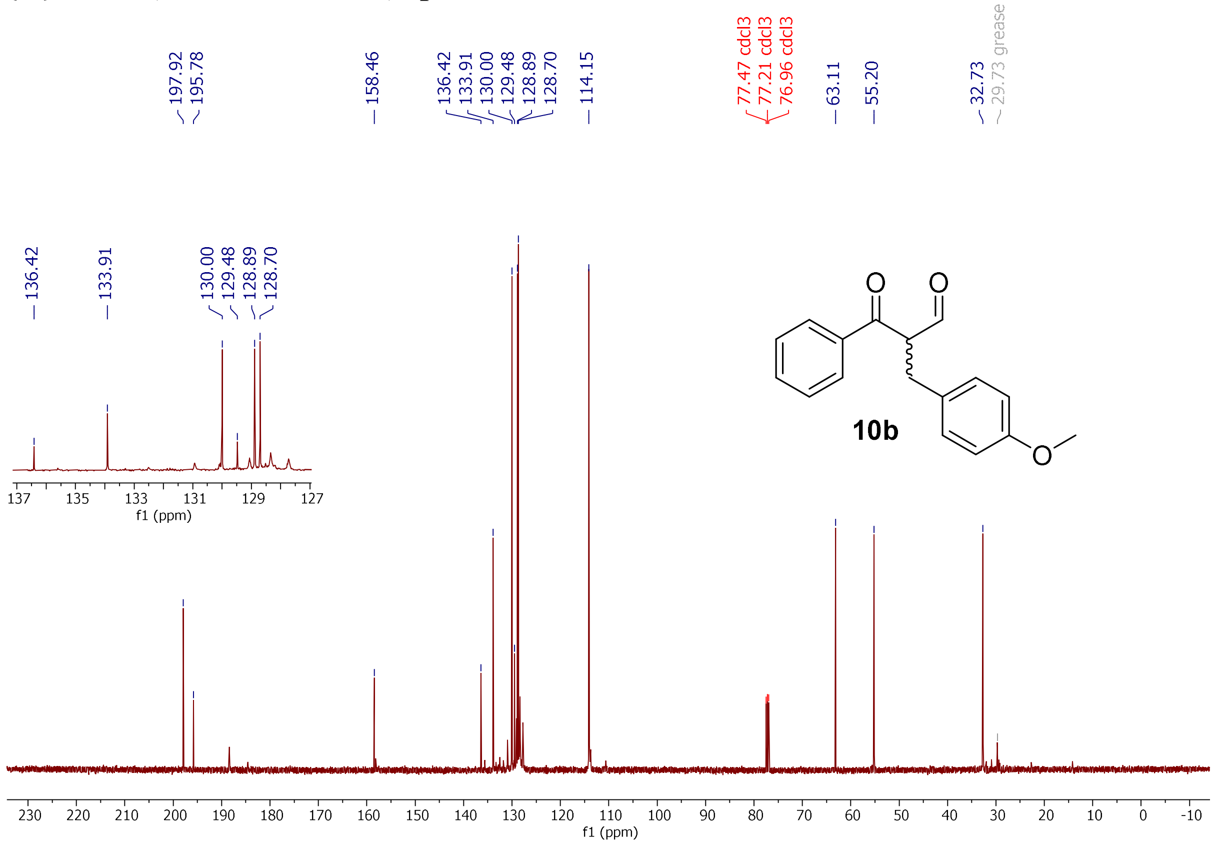

^{13}C NMR (126 MHz, $CDCl_3$) δ 197.9, 195.8, 158.5, 136.4, 133.9, 130.0, 129.5, 128.9, 128.7, 114.1, 63.1, 55.2, 32.7 ***(only the aldehyde was identified)***.

^{1}H NMR (500 MHz, $CDCl_3$) spectrum of 15c

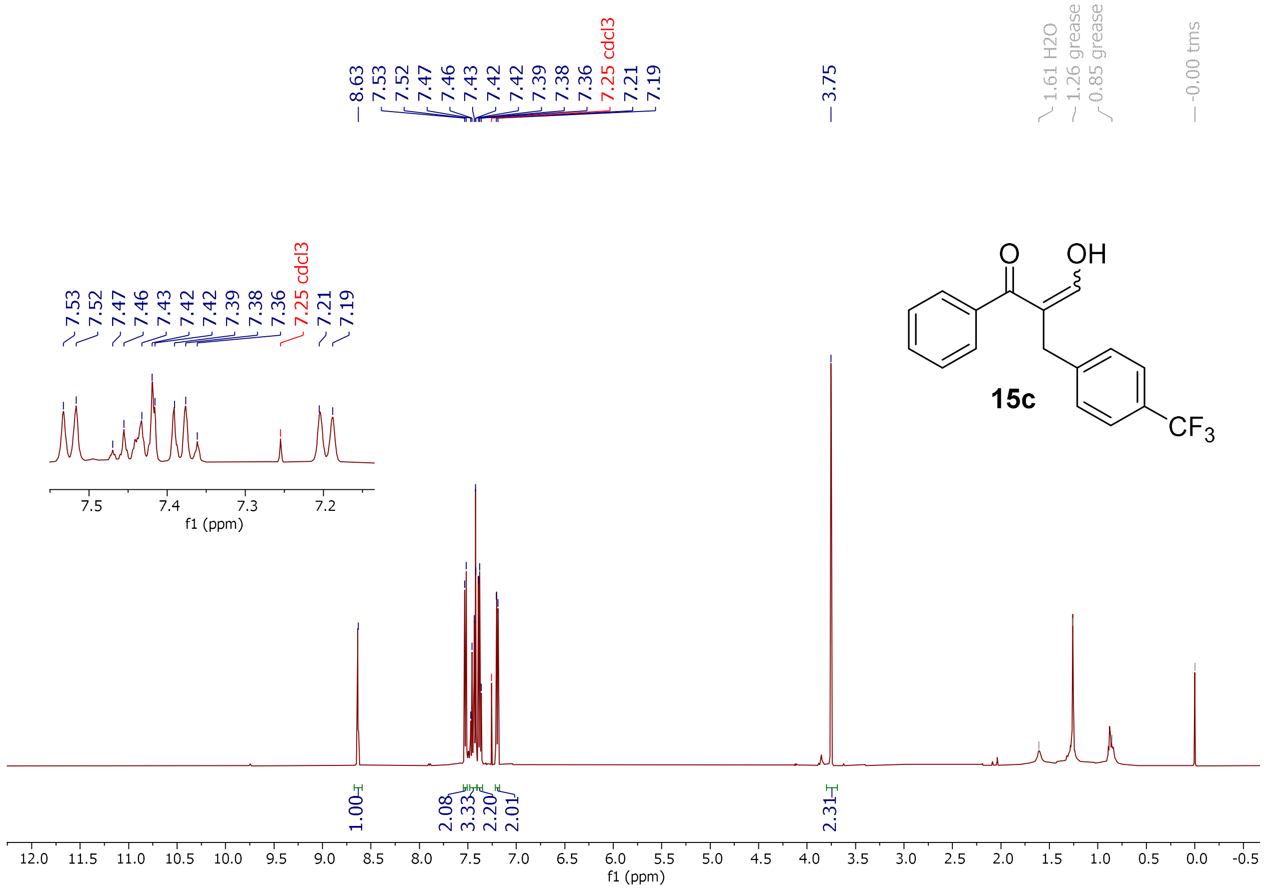

^{1}H NMR (500 MHz, $CDCl_3$) δ 8.63 (s, 1H), 7.52 (d, *J* = 8.2 Hz, 2H), 7.48 – 7.41 (m, 3H), 7.38 (t, *J* = 7.3 Hz, 2H), 7.20 (d, *J* = 8.6 Hz, 2H), 3.75 (s, 2H). Tautomeric ratio: 100:0 (enol/aldehyde).

$^{13}C\{H\}$ NMR (126 MHz, $CDCl_3$) spectrum of 15c

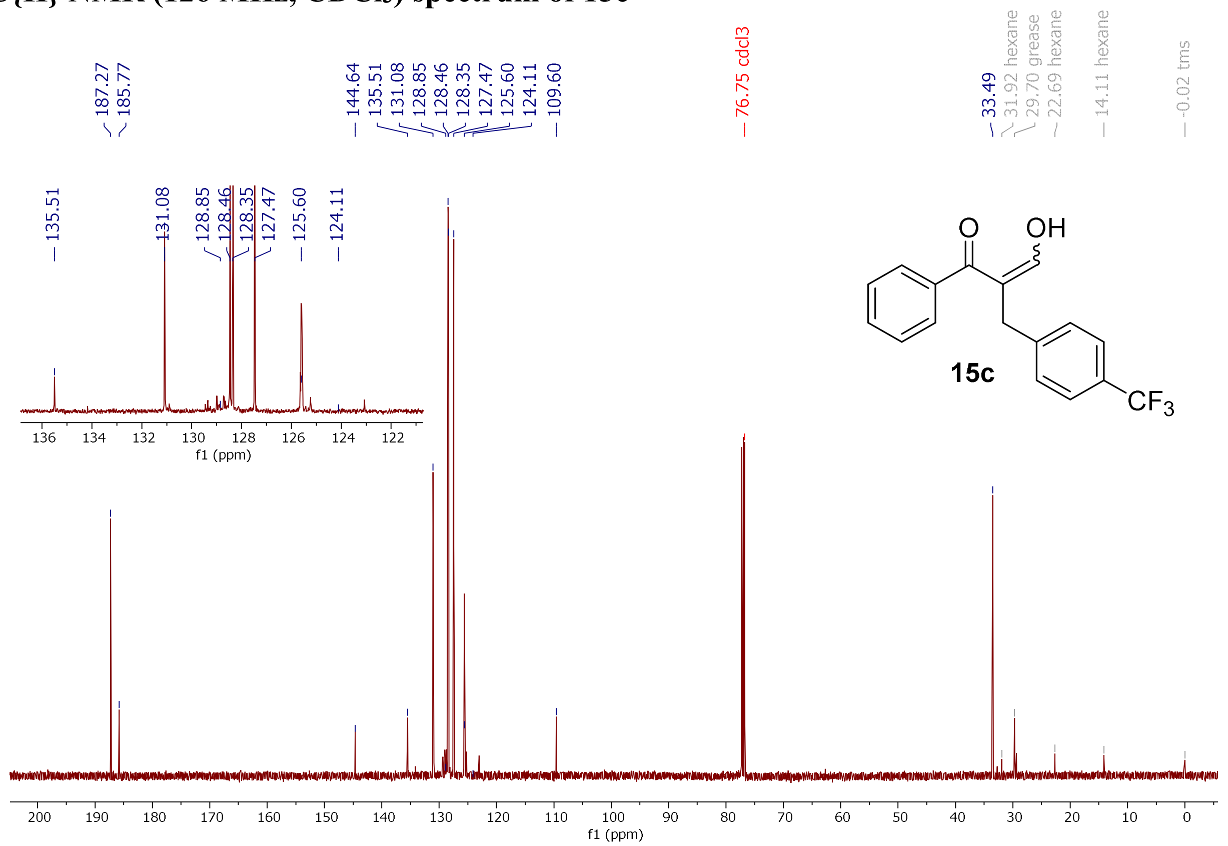

^{13}C NMR (126 MHz, $CDCl_3$) δ 187.3, 185.8, 144.6, 135.5, 131.1, 128.9 (d, *J* = 32.9 Hz), 128.5, 128.3, 127.5, 125.1 (q, *J* = 3.8 Hz), 124.1 (q, *J* = 271.8 Hz), 109.6, 33.5.

^{1}H NMR (400 MHz, $(CD_3)_2CO$) spectrum of 10d/15d

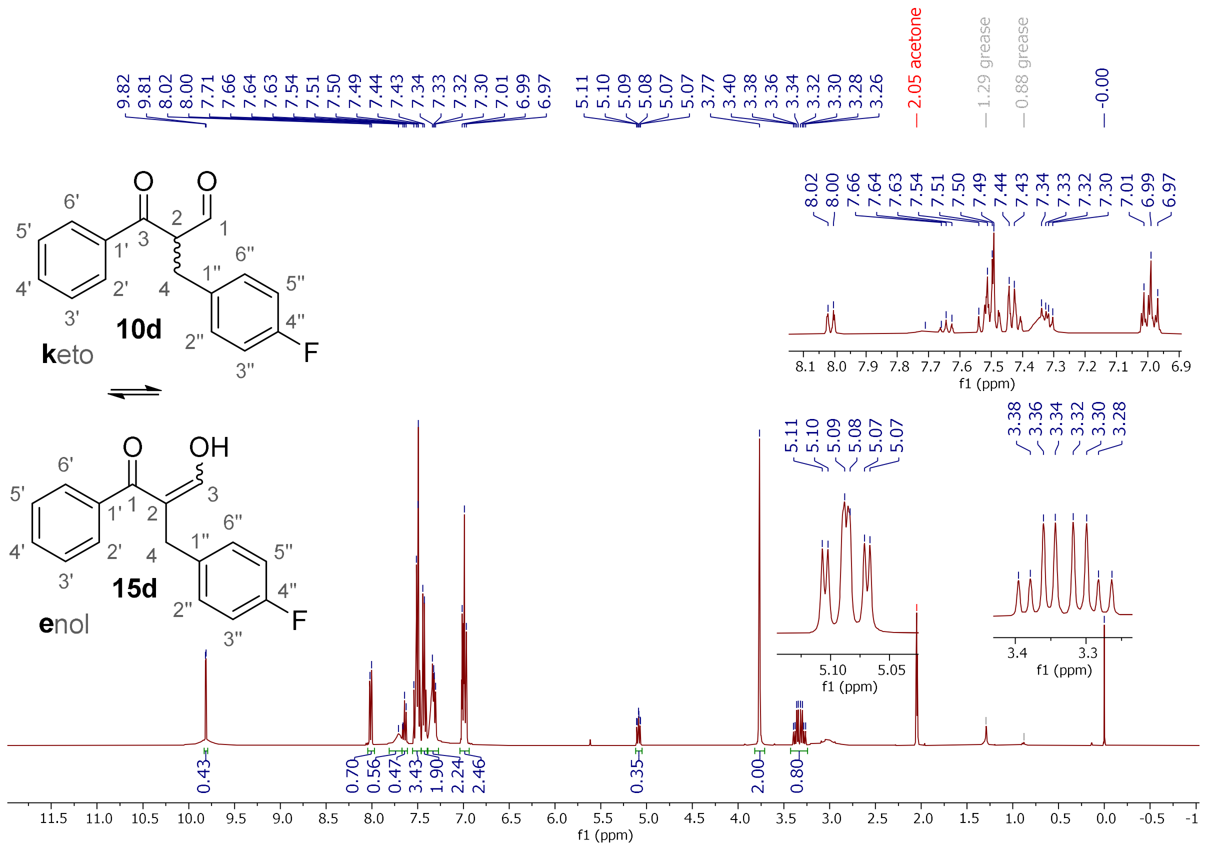

^{1}H NMR (400 MHz, $(CD_3)_2CO$, **mixture of tautomers**) δ 9.81 (d, J = 1.9 Hz, 1H, H1-K), 8.03 – 6.97 (m, 19H, H3-E, H6'-K, H5'-K, H4'-K, H3'-K, H2'-K, H6''-K, H5''-K, H3''-K, H2''-K, H6'-E, H5'-E, H4'-E, H3'-E, H2'-E, H6''-E, H5''-E, H3''-E, H2''-E), 5.09 (td, J = 7.1, 1.9 Hz, 1H, H2-K), 3.77 (s, 2H, H4-E), 3.43 – 3.24 (m, 2H, H4-K). Tautomeric ratio: 85:15 (enol/aldehyde).

^{13}C{H} NMR (101 MHz, $(CD_3)_2CO$) spectrum of 10d

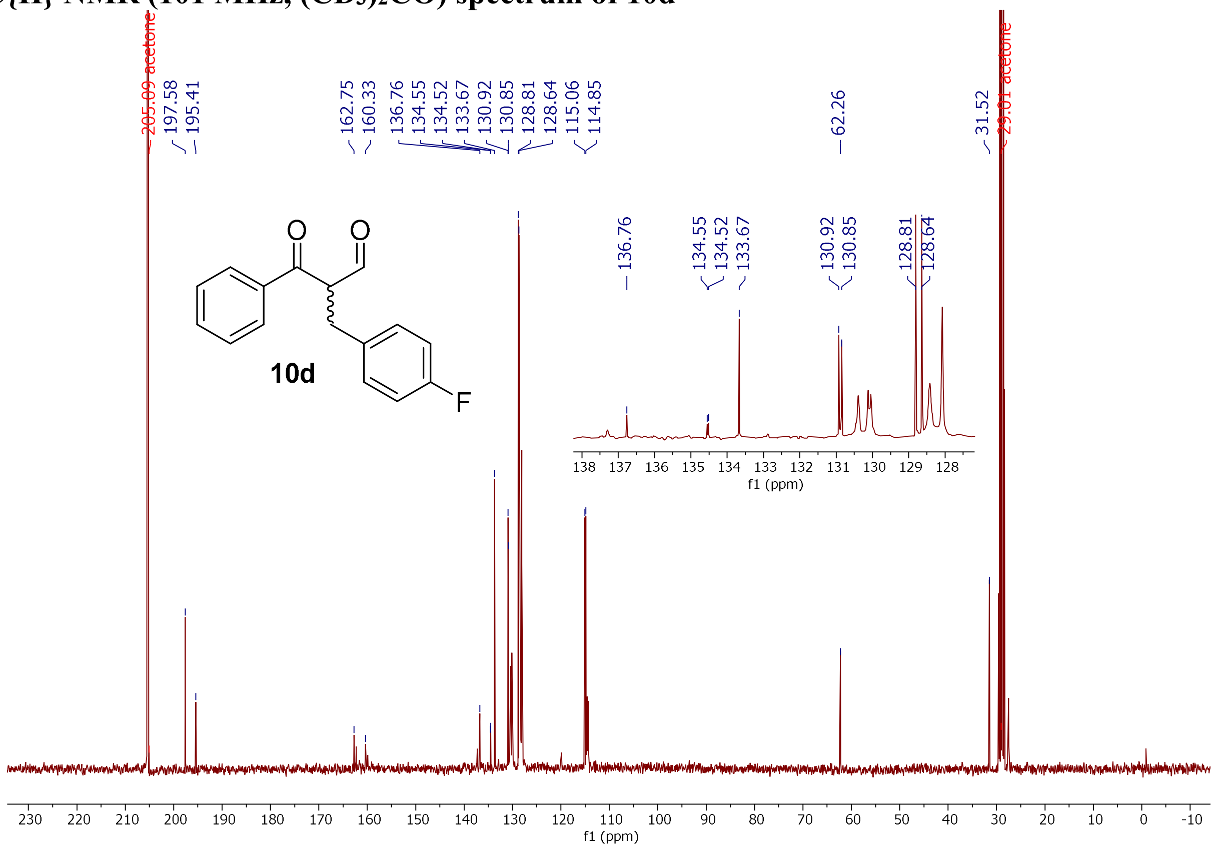

^{13}C NMR (101 MHz, $(CD_3)_2CO$) δ 197.6, 195.4, 161.5 (d, J = 243.0 Hz), 136.8, 134.5 (d, J = 3.4 Hz), 133.7, 130.9, 130.85, 128.8, 128.6, 114.9 (d, J = 21.4 Hz), 62.3, 31.5 ***(only the aldehyde was identified)***.

^{1}H NMR (500 MHz, $(CD_3)_2CO$) spectrum of 10e/15e

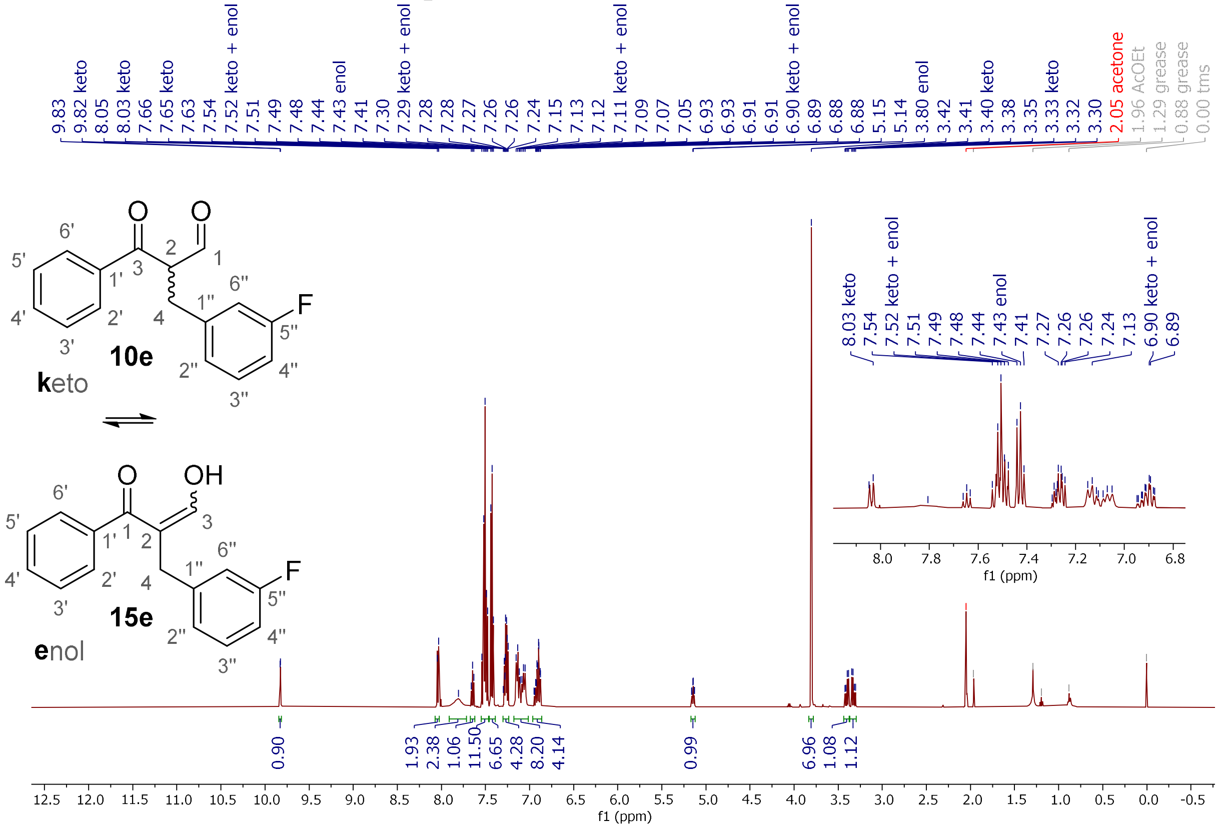

^{1}H NMR (500 MHz, $(CD_3)_2CO$, **mixture of tautomers**) δ 9.83 (d, J = 1.8 Hz, 1H, H1-K), 8.05 – 6.85 (m, 19H, H3-E, H6'-K, H5'-K, H4'-K, H3'-K, H2'-K, H6''-K, H4''-K, H3''-K, H2''-K, H6'-E, H5'-E, H4'-E, H3'-E, H2'-E, H6''-E, H4''-E, H3''-E, H2''-E), 5.15 (td, J = 7.1, 1.8 Hz, 1H, H2-K), 3.80 (s, 2H, H4-E), 3.40 (dd, J = 14.2, 6.8 Hz, 1H, H4-K), 3.32 (dd, J = 14.2, 7.5 Hz, 1H, H4-K). Tautomeric ratio: 88:12 (enol/aldehyde).

^{13}C{H} NMR (126 MHz, $(CD_3)_2CO$) spectrum of 10e/15e

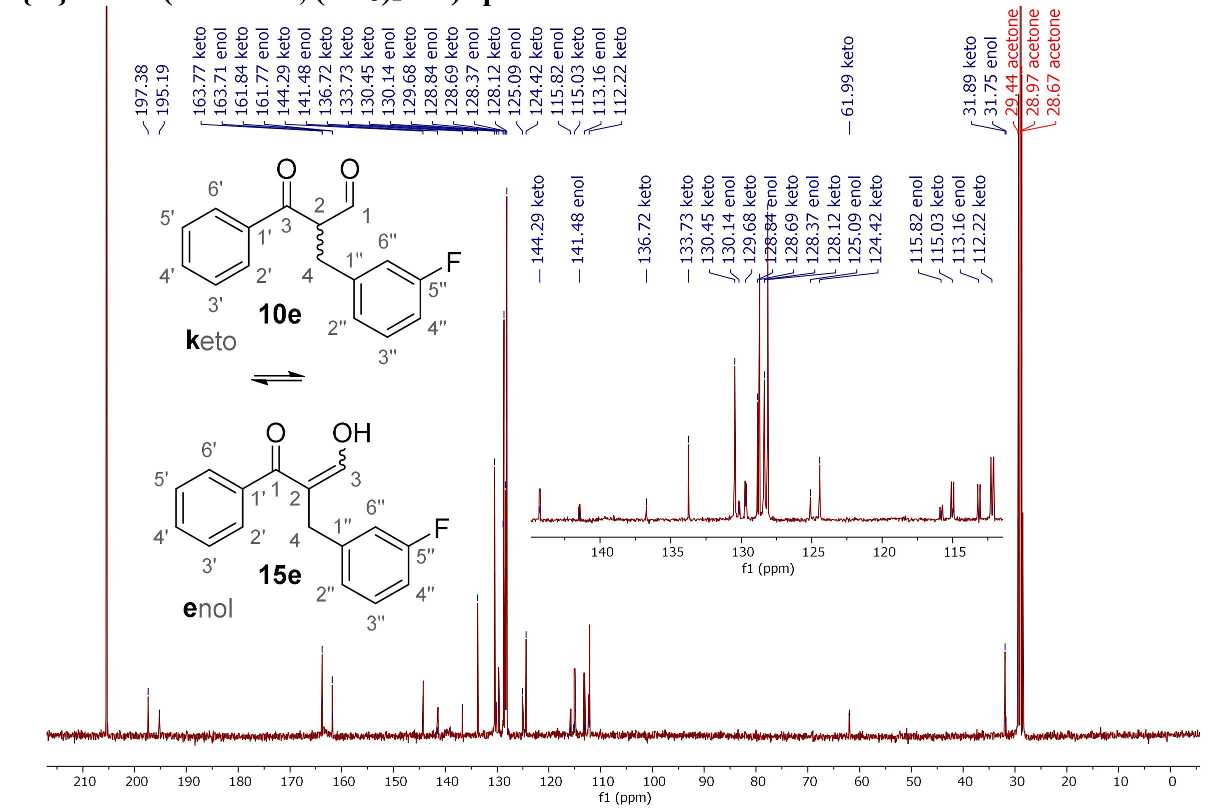

^{13}C NMR (126 MHz, $(CD_3)_2CO$, **mixture of tautomers**) δ 197.4, 195.2, 162.8 (d, J = 242.7 Hz), 162.7 (d, J = 243.7 Hz), 144.3 (d, J = 7.2 Hz), 141.5 (d, J = 7.6 Hz), 136.7, 133.7, 130.4, 130.1 (d, J = 8.6 Hz), 129.7 (d, J = 9.1 Hz), 128.8, 128.7, 128.4, 128.1, 125.1, 124.4, 115.8 (d, J = 19.1 Hz), 115.0 (d, J = 21.0 Hz), 113.1 (d, J = 21.0 Hz), 112.2 (d, J = 21.0 Hz), 62.0, 31.9, 31.7.

^{1}H NMR (400 MHz, CDCl$_3$) spectrum of 10f/15f

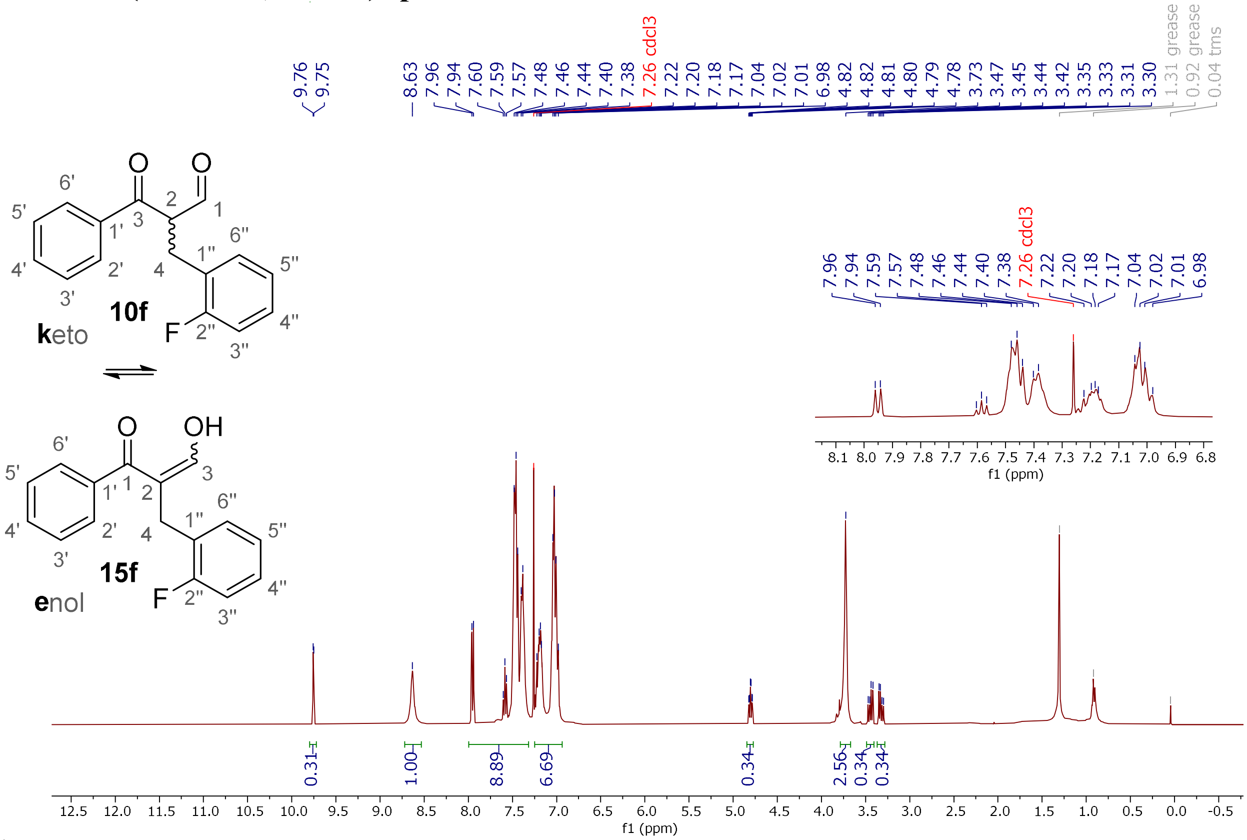

^{1}H NMR (400 MHz, CDCl$_3$, **mixture of tautomers**) δ 9.75 (d, J = 2.5 Hz, 1H, H1-K), 8.63 (s, 1H, H3-E), 7.99 – 6.94 (m, 18H, H6'-K, H5'-K, H4'-K, H3'-K, H2'-K, H6''-K, H5''-K, H4''-K, H3''-K, H6'-E, H5'-E, H4'-E, H3'-E, H2'-E, H6''-E, H5''-E, H4''-E, H3''-E), 4.80 (td, J = 7.1, 2.6 Hz, 1H, H2-K), 3.73 (s, 2H, H4-E), 3.44 (dd, J = 14.2, 7.5 Hz, 1H, H4-K), 3.32 (dd, J = 14.2, 6.8 Hz, 1H, H4-K). Tautomeric ratio: 76:24 (enol/aldehyde).

^{13}C{H} NMR (126 MHz, CDCl$_3$) spectrum of 10f

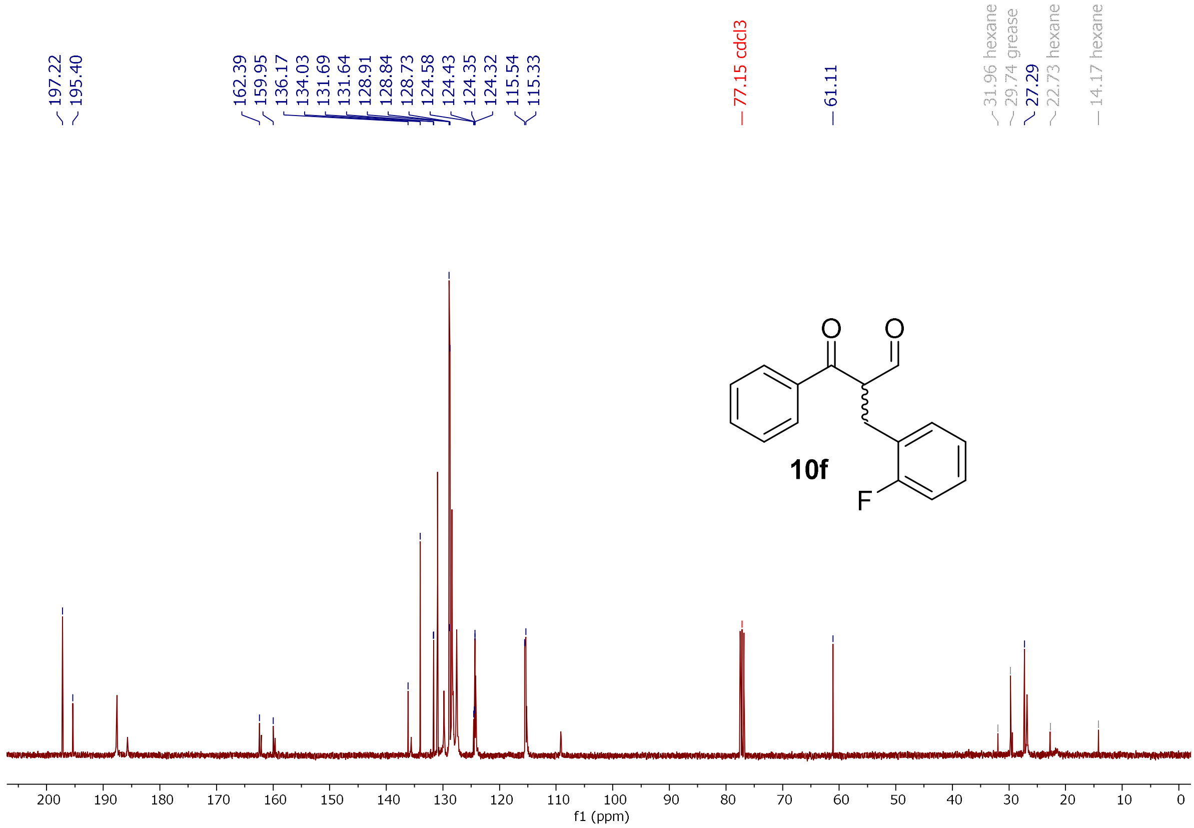

^{13}C NMR (101 MHz, CDCl$_3$) δ 197.2, 195.4, 161.2 (d, J = 244.9 Hz), 136.2, 134.0, 131.7 (d, J = 4.6 Hz), 128.9, 128.8, 128.7, 124.6, 124.4, 124.3, 124.3, 115.4 (d, J = 21.7 Hz), 61.1, 27.3 ***(only the aldehyde was identified).***

^{1}H NMR (500 MHz, CDCl$_3$) spectrum of 10g/15g

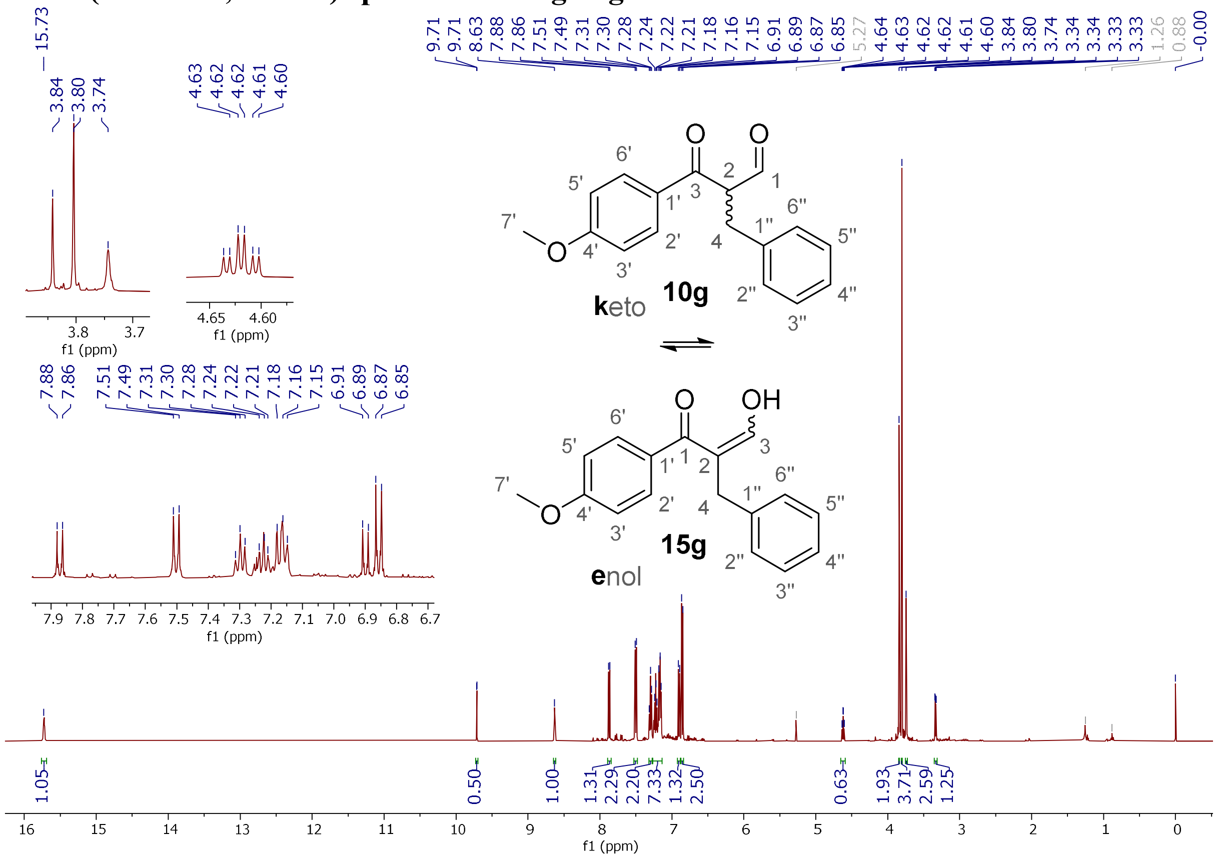

^{1}H NMR (500 MHz, CDCl$_3$, **mixture of tautomers**) δ 15.73 (s, 1H, OH), 9.71 (d, J = 2.9 Hz, 1H, H1-K), 8.63 (s, 1H, H3-E), 7.94 – 6.82 (m, 18H, H6'-K, H5'-K, H3'-K, H2'-K, H6''-K, H5''-K, H4''-K, H3''-K, H2''-K, H6'-E, H5'-E, H3'-E, H2'-E, H6''-E, H5''-E, H4''-E, H3''-E, H2''-E), 4.62 (td, J = 7.0, 2.9 Hz, 1H, H2-K), 3.84 (s, 3H, H7'-K), 3.80 (s, 3H, H7'-E), 3.74 (s, 2H, H4-E), 3.34 (dd, J = 7.0, 1.8 Hz, 2H, H4-K). Tautomeric ratio: 67:33 (enol/aldehyde).

^{13}C{H} NMR (126 MHz, CDCl$_3$) spectrum of 10f

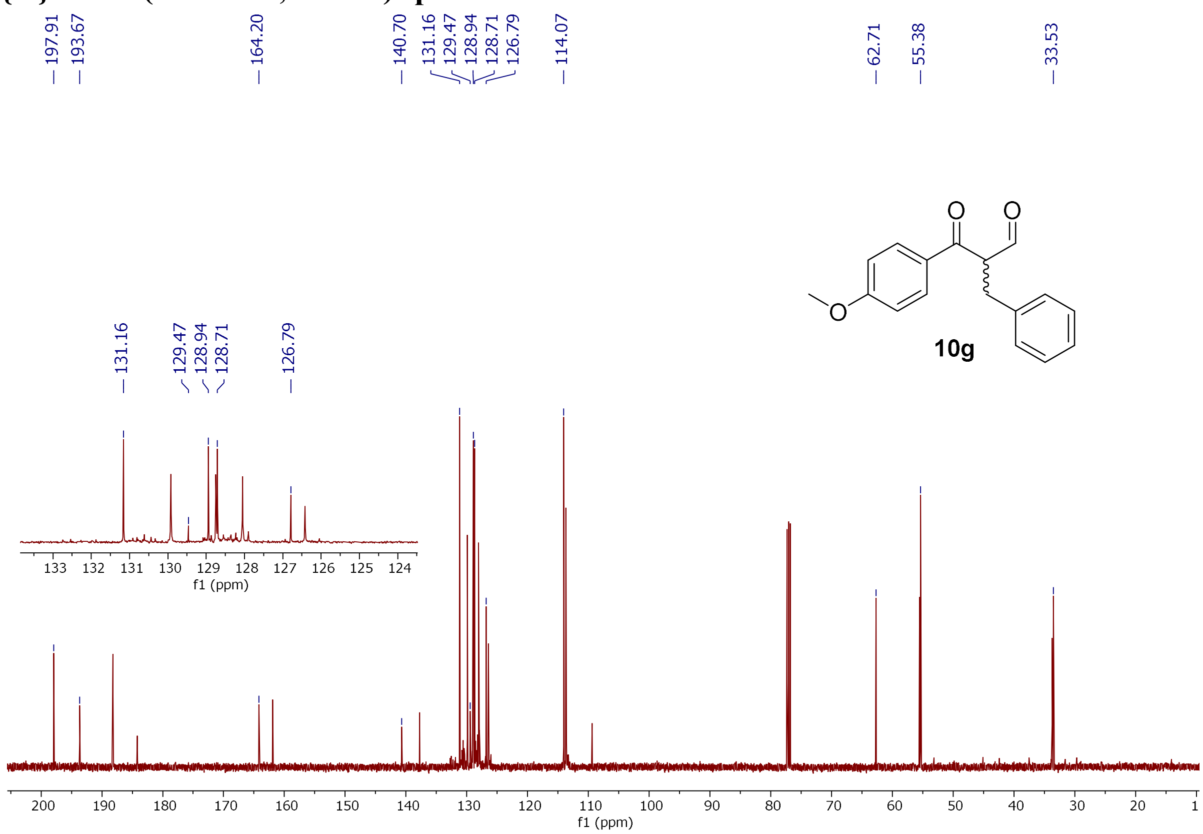

^{13}C NMR (126 MHz, CDCl$_3$) δ 197.9, 193.7, 164.2, 140.7, 131.2, 129.5, 128.9, 128.7, 126.8, 114.1, 62.7, 55.4, 33.5 ***(only the aldehyde was identified)***.

^{1}H NMR (500 MHz, CDCl$_3$) spectrum of 10h/15h

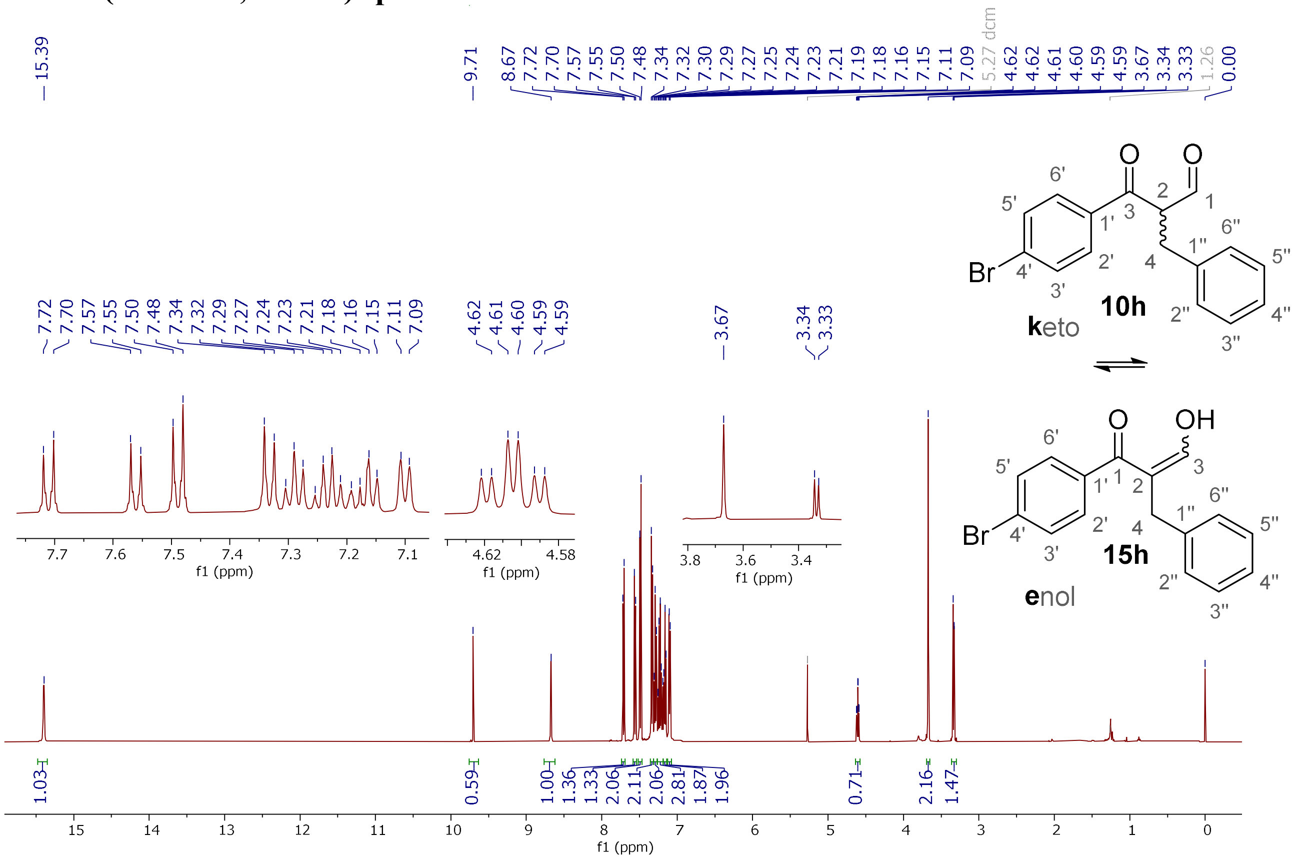

^{1}H NMR (500 MHz, CDCl$_3$, **mixture of tautomers**) δ 15.39 (s, 1H, OH), 9.71 (s, 1H, H1-K), 8.67 (s, 1H, H3-E), 7.72 – 7.05 (m, 18H, H6’-K, H5’-K, H3’-K, H2’-K, H6”-K, H5”-K, H4”-K, H3”-K, H2”-K, H6’-E, H5’-E, H3’-E, H2’-E, H6”-E, H5”-E, H4”-E, H3”-E, H2”-E), 4.60 (td, J = 7.2, 2.7 Hz, 1H, H2-K), 3.67 (s, 2H, H4-E), 3.34 (d, J = 7.6 Hz, 2H, H4-K). Tautomeric ratio: 63:37 (enol/aldehyde).

^{13}C{H} NMR (126 MHz, CDCl$_3$) spectrum of 10h

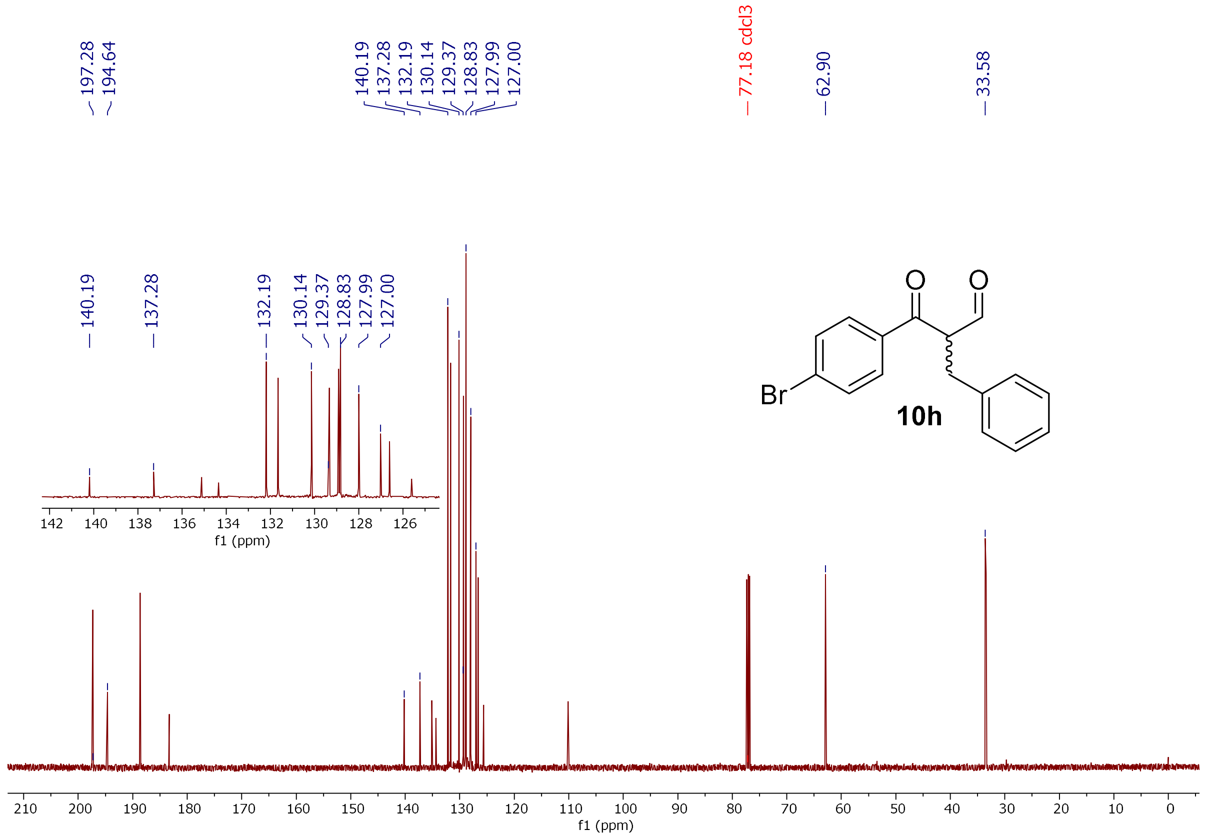

^{13}C NMR (126 MHz, CDCl$_3$) δ 197.3, 194.6, 140.2, 137.3, 132.2, 130.1, 129.4, 128.8, 128.0, 127.0, 62.9, 33.6 ***(only the aldehyde was identified)***.

^{1}H NMR (400 MHz, CDCl$_3$) spectrum of 10i/15i

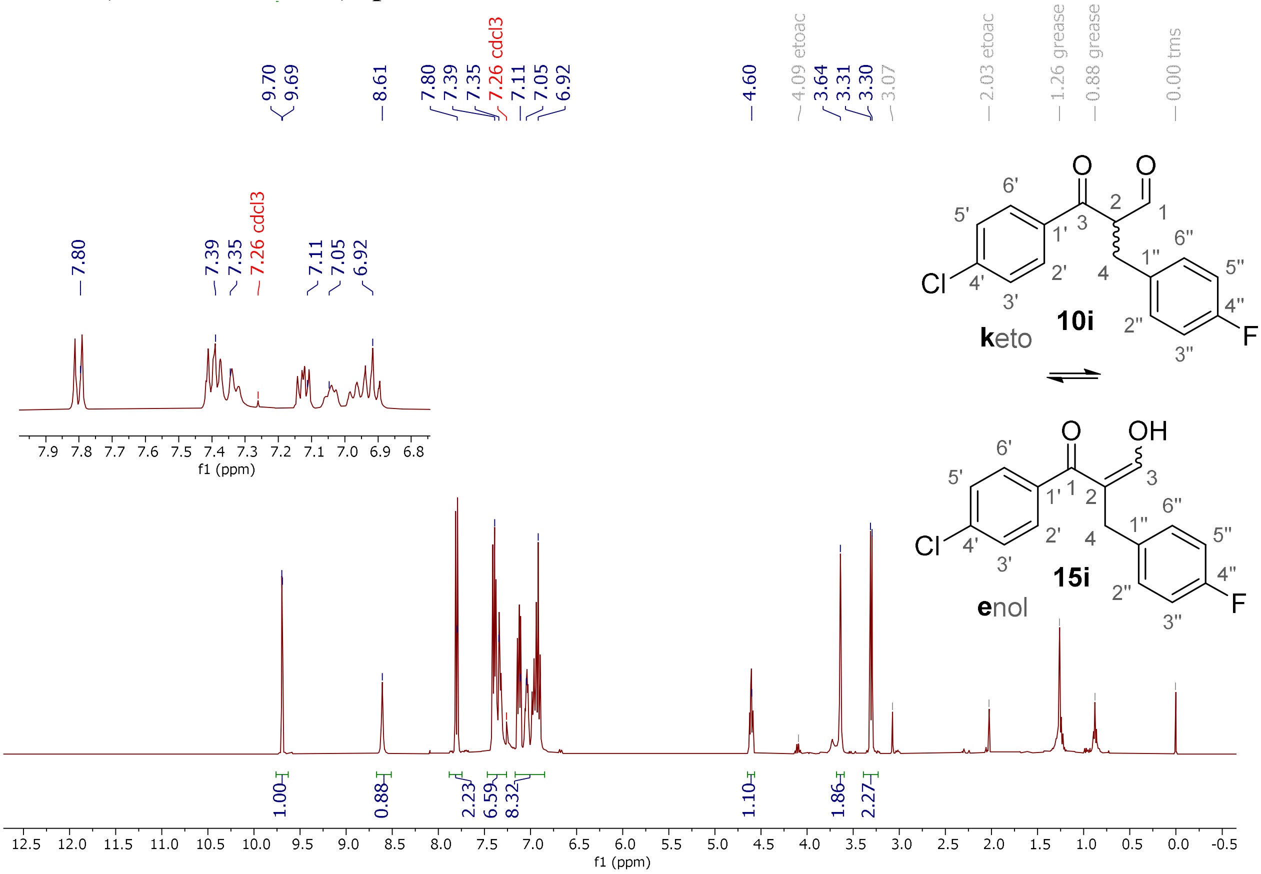

^{1}H NMR (400 MHz, CDCl$_3$, **mixture of tautomers**) δ 9.69 (d, J = 2.6 Hz, 1H, H1-K), 8.61 (s, 1H, H3-E), 7.80 – 6.85 (m, 16H), 4.61 (td, J = 7.1, 2.7 Hz, 1H, H2-K), 3.64 (s, 2H, H4-E), 3.31 (d, J = 7.2 Hz, 2H, H4-K). Tautomeric ratio: 47:53 (enol/aldehyde).

^{13}C{H} NMR (126 MHz, (CD$_3$)$_2$CO) spectrum of 10i

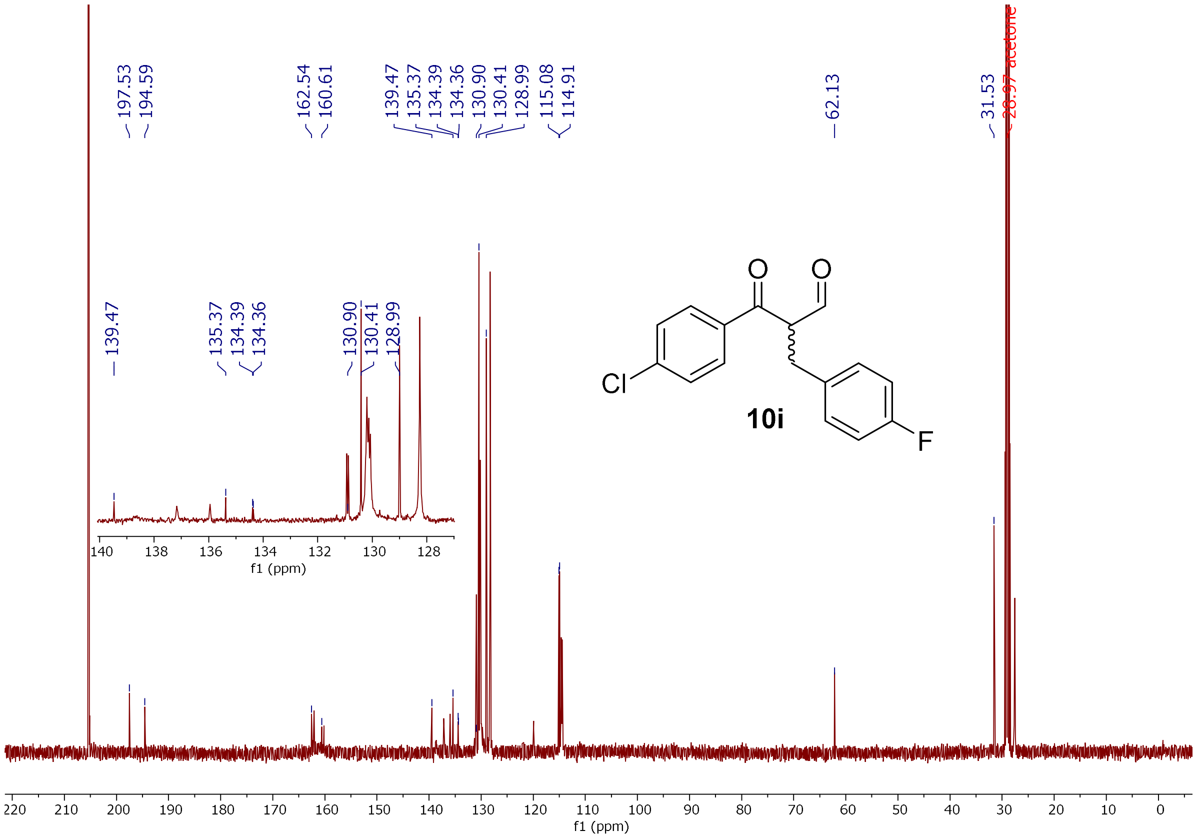

^{13}C NMR (126 MHz, (CD$_3$)$_2$CO) δ 197.5, 194.6, 161.6 (d, J = 242.7 Hz), 139.5, 135.4, 134.4 (d, J = 2.9 Hz), 130.9, 130.4, 129.0, 115.0 (d, J = 21.5 Hz), 62.1, 31.5 ***(only the aldehyde was identified)***.

^{1}H NMR (400 MHz, $CDCl_3$) spectrum of 10j/15j

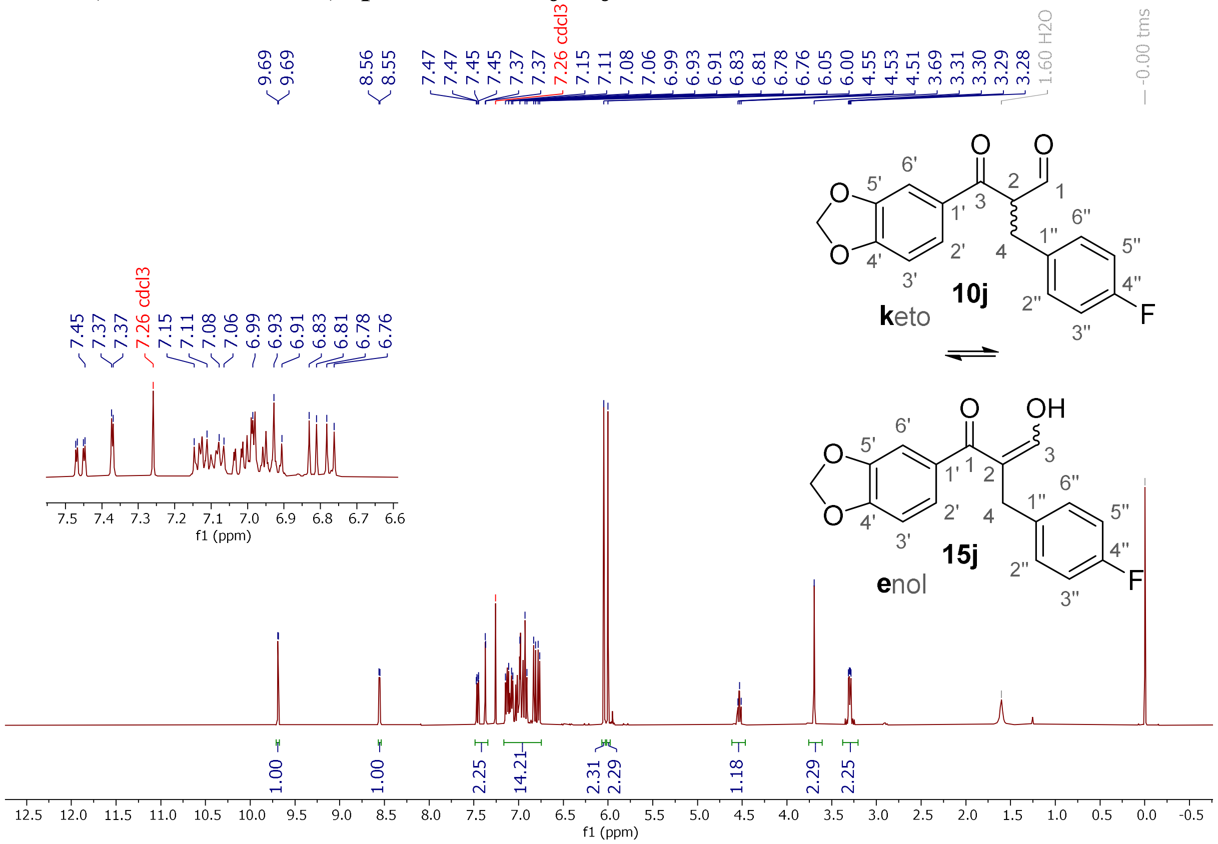

^{1}H NMR (400 MHz, $CDCl_3$, **mixture of tautomers**) δ 9.69 (d, J = 2.8 Hz, 1H, H1-K), 8.56 (d, J = 4.4 Hz, 1H, H3-E), 7.49 – 6.75 (m, 14H, H6'-K, H3'-K, H2'-K, H6''-K, H5''-K, H3''-K, H2''-K, H6'-E, H3'-E, H2'-E, H6''-E, H5''-E, H3''-E, H2''-E), 6.05 (s, 2H, H5-K), 6.00 (s, 2H, H5-E), 4.53 (t, J = 7.1 Hz, 1H, H2-K), 3.69 (s, 2H, H4-E), 3.30 (dd, J = 7.1, 3.9 Hz, 2H, H4-K). Tautomeric ratio: 50:50 (enol/aldehyde).

^{13}C{H} NMR (126 MHz, $CDCl_3$) spectrum of 15j

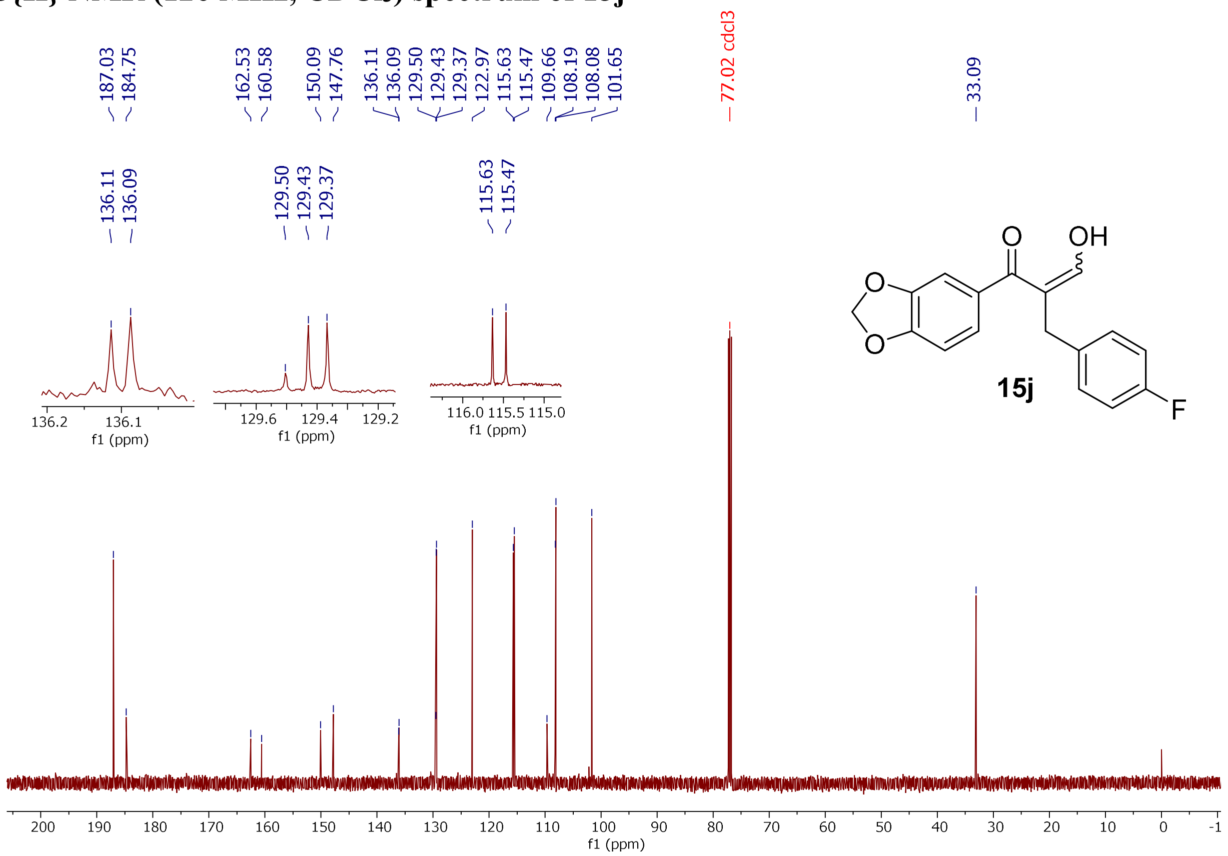

^{13}C NMR (126 MHz, $CDCl_3$) δ 187.0, 184.7, 161.6 (d, J = 245.1 Hz), 150.1, 147.8, 136.1 (d, J = 3.3 Hz), 129.5, 129.4, 129.4, 123.0, 115.5 (d, J = 21.0 Hz), 109.7, 108.2, 108.1, 101.6, 33.1.

^{1}H NMR (500 MHz, DMSO-d_6) spectrum of 4a

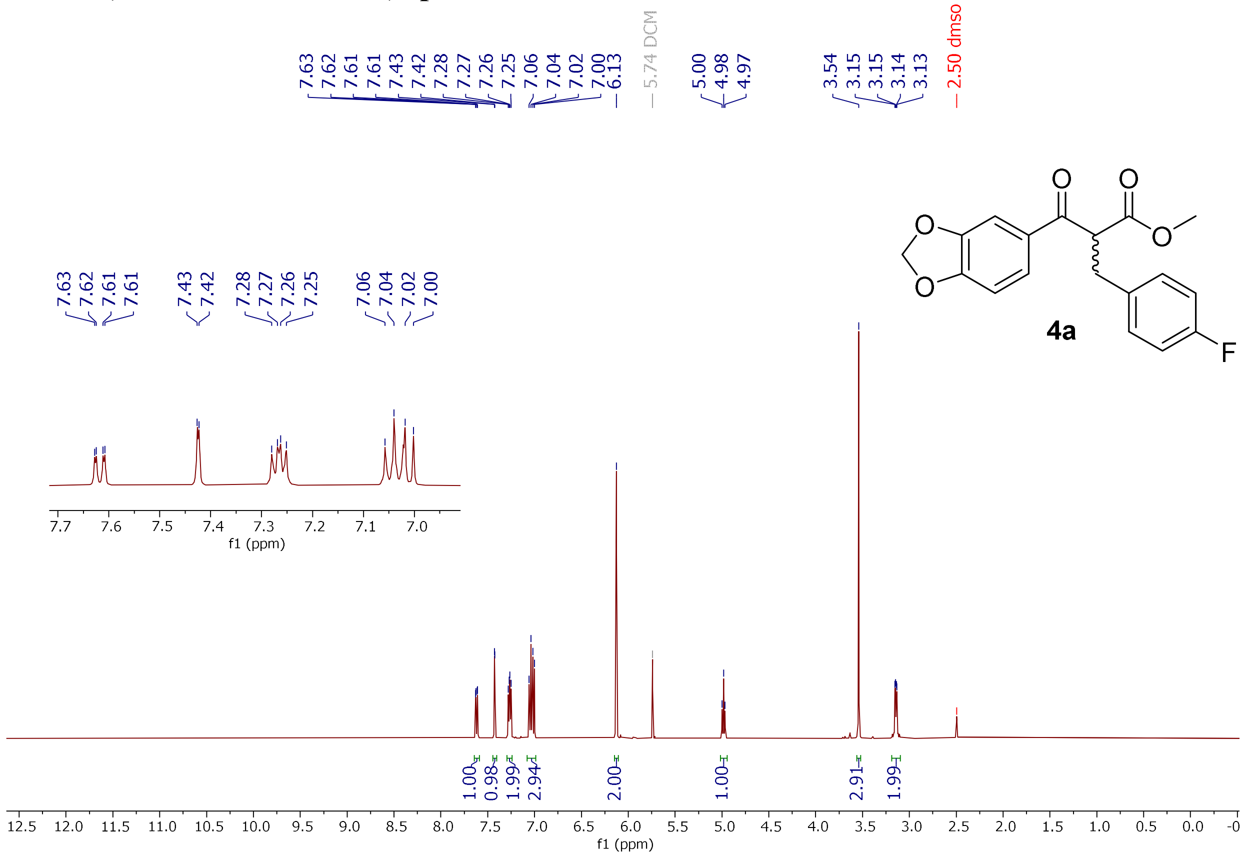

^{1}H NMR (500 MHz, DMSO-d_6) δ 7.62 (dd, J = 8.3, 1.8 Hz, 1H), 7.42 (d, J = 1.8 Hz, 1H), 7.27 (dd, J = 8.8, 5.5 Hz, 2H), 7.08 – 6.99 (m, 3H), 6.13 (s, 2H), 4.98 (t, J = 7.6 Hz, 1H), 3.54 (s, 3H), 3.14 (dd, J = 7.6, 2.8 Hz, 2H).

^{13}C{H} NMR (126 MHz, DMSO-d_6) spectrum of 4a

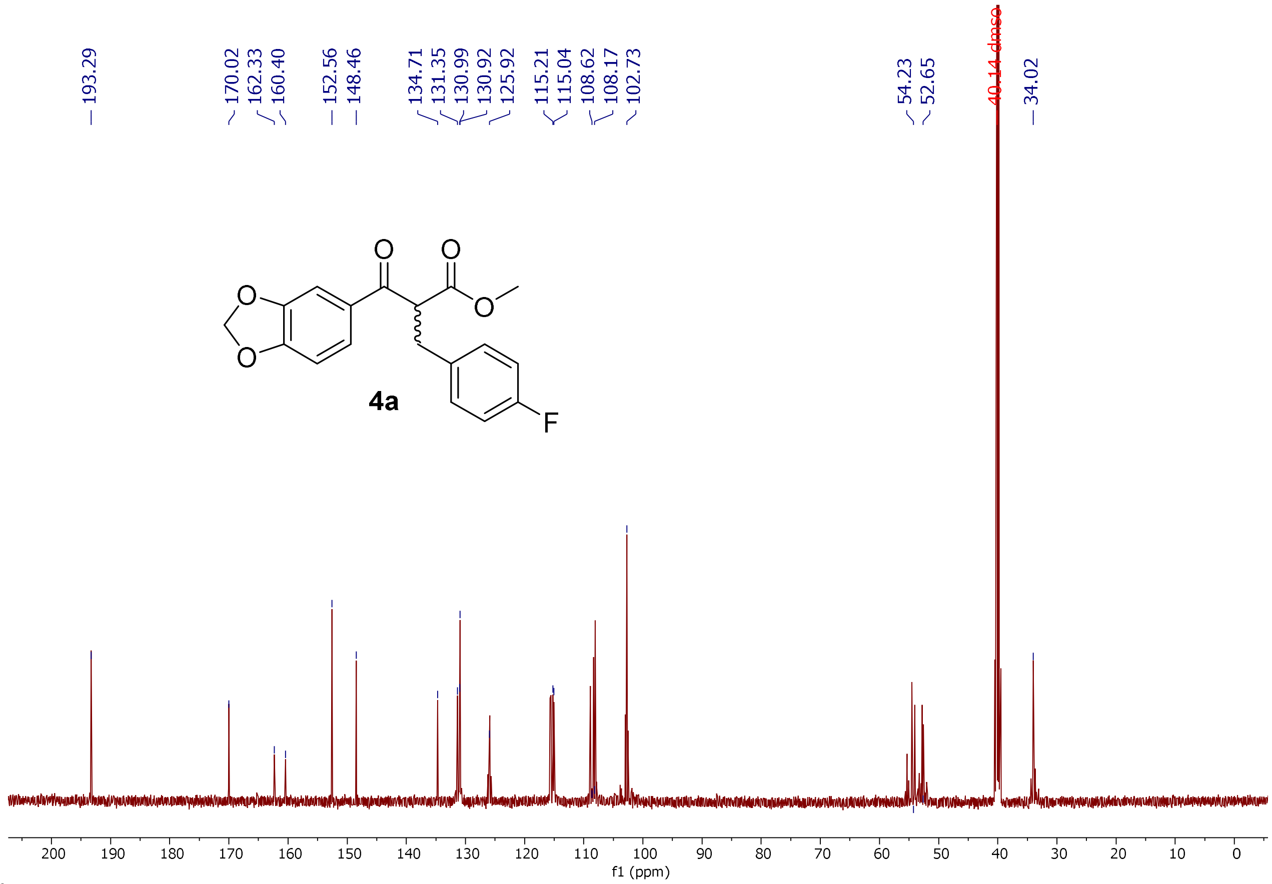

^{13}C NMR (126 MHz, DMSO-d_6) δ 193.3, 170.0, 161.4 (d, J = 242.2 Hz), 152.6, 148.5, 134.7, 131.3, 131.0, 130.9, 125.9, 115.1 (d, J = 21.5 Hz), 108.6, 108.2, 102.7, 54.2, 52.6, 34.0.

^{1}H NMR (500 MHz, $(CD_3)_2CO$) spectrum of 12a

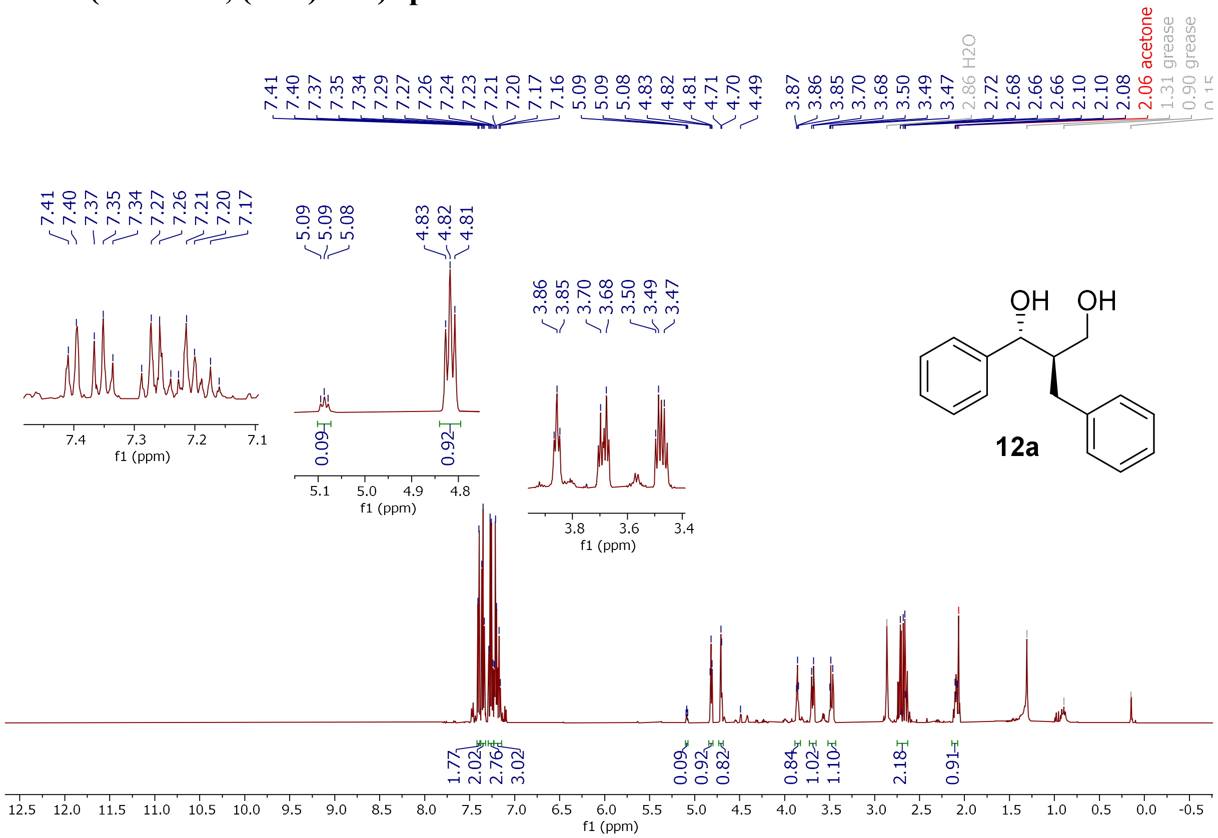

^{1}H NMR (500 MHz, $(CD_3)_2CO$, **only majority diastereoisomer was identified**) δ 7.40 (d, J = 6.7 Hz, 2H), 7.35 (t, J = 7.6 Hz, 2H), 7.29 – 7.23 (m, 3H), 7.23 – 7.15 (m, 3H), 5.09 (t, J = 4.1 Hz, 0H), 4.82 (t, J = 5.0 Hz, 1H), 4.70 (d, J = 4.5 Hz, 1H), 3.86 (t, J = 5.1 Hz, 1H), 3.69 (d, J = 10.8 Hz, 1H), 3.52 – 3.43 (m, 1H), 2.75 – 2.63 (m, 2H), 2.14 – 2.08 (m, 1H). Diastereomeric ratio 91:9 (anti/syn).

^{13}C{H} NMR (126 MHz, $(CD_3)_2CO$) spectrum of 12a

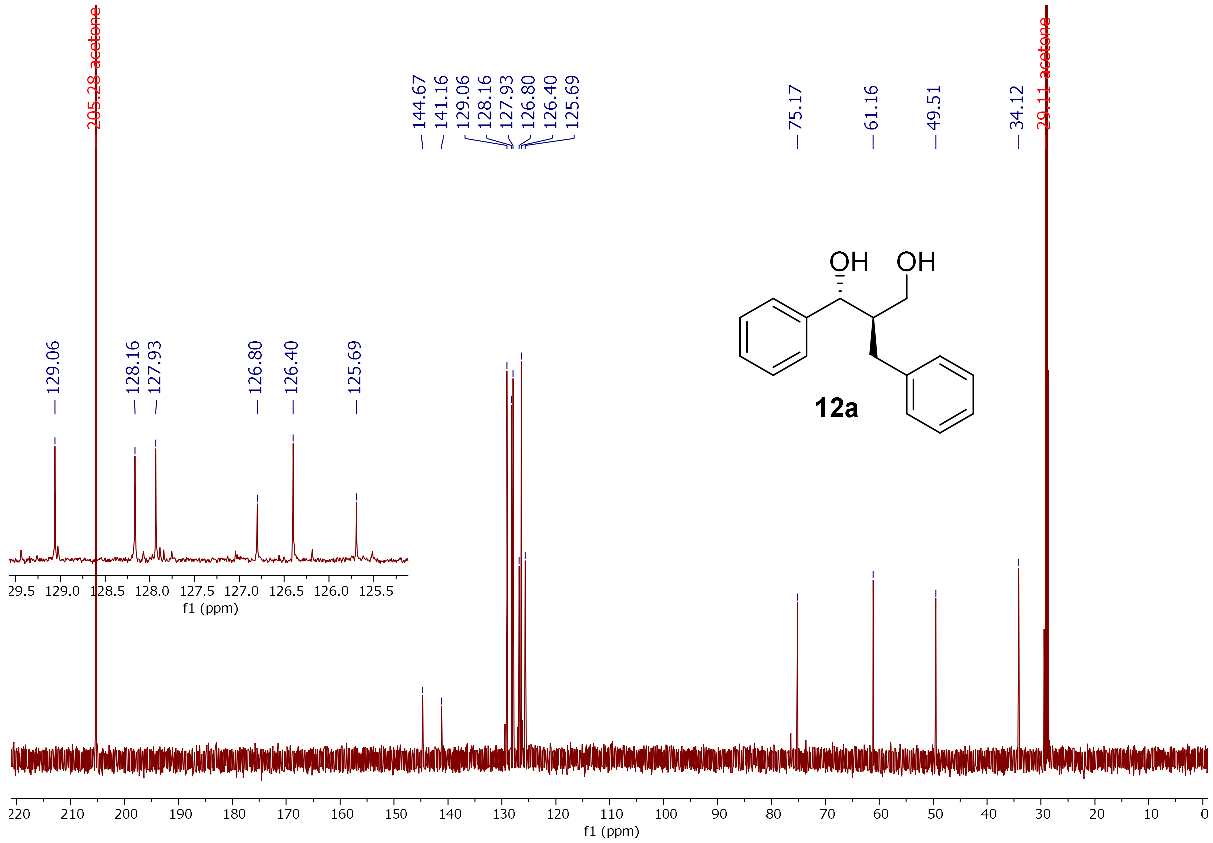

^{13}C NMR (126 MHz, $(CD_3)_2CO$) δ 144.7, 141.2, 129.1, 128.2, 127.9, 126.8, 126.4, 125.7, 75.2, 61.2, 49.5, 34.1.

^{1}H NMR (400 MHz, $CDCl_3$) spectrum of 12b

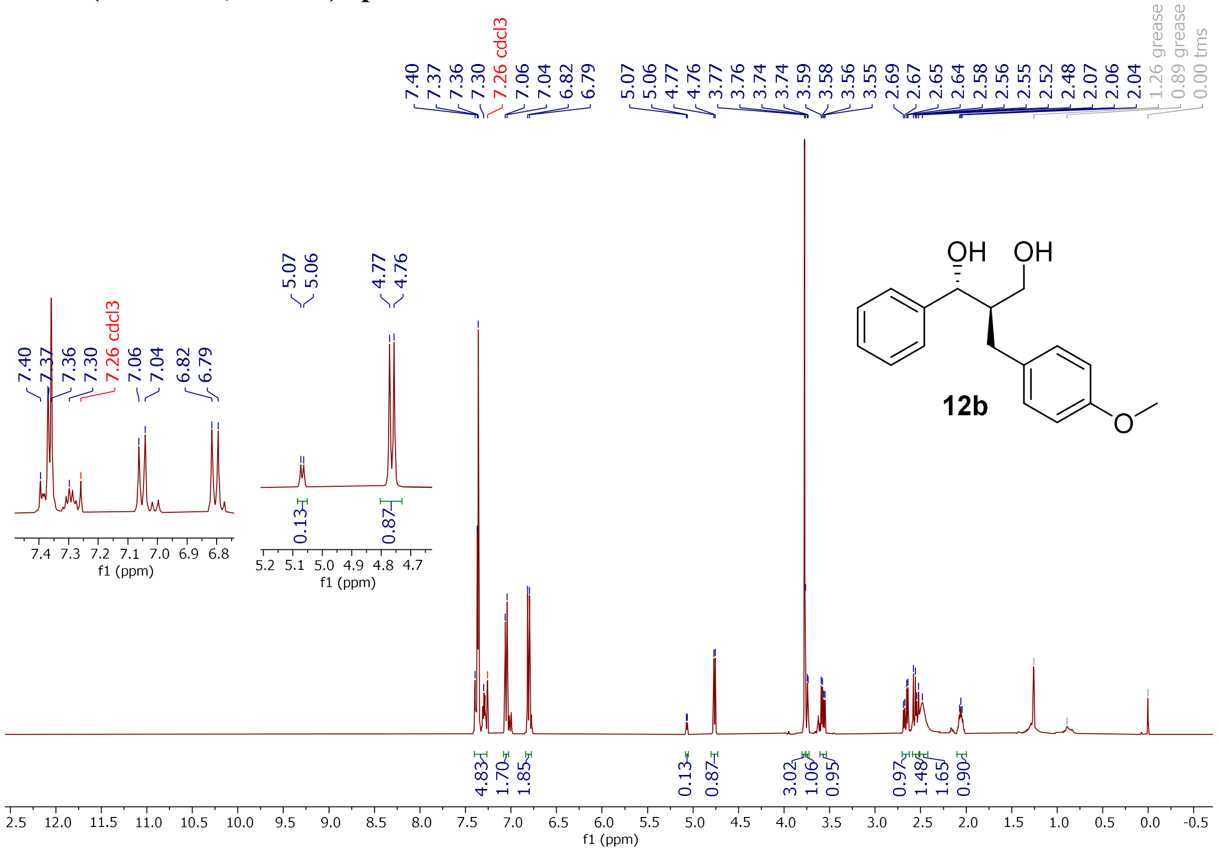

^{1}H NMR (400 MHz, $CDCl_3$, **only majority diastereoisomer was identified**) δ 7.41 – 7.27 (m, 5H), 7.05 (d, *J* = 8.7 Hz, 2H), 6.81 (d, *J* = 8.7 Hz, 2H), 5.07 (d, *J* = 4.1 Hz, 0H), 4.76 (d, *J* = 6.2 Hz, 1H), 3.77 (s, 3H), 3.75 – 3.72 (m, 1H), 3.57 (dd, J = 11.1, 5.5 Hz, 1H), 2.66 (dd, J = 13.9, 5.8 Hz, 1H), 2.55 (dd, J = 13.9, 9.3 Hz, 1H), 2.48 (s, 1H), 2.12 – 2.01 (m, 1H). Diastereomeric ratio 87:13 (anti/syn).

^{13}C{H} NMR (126 MHz, $(CD_3)_2CO$) spectrum of 12b

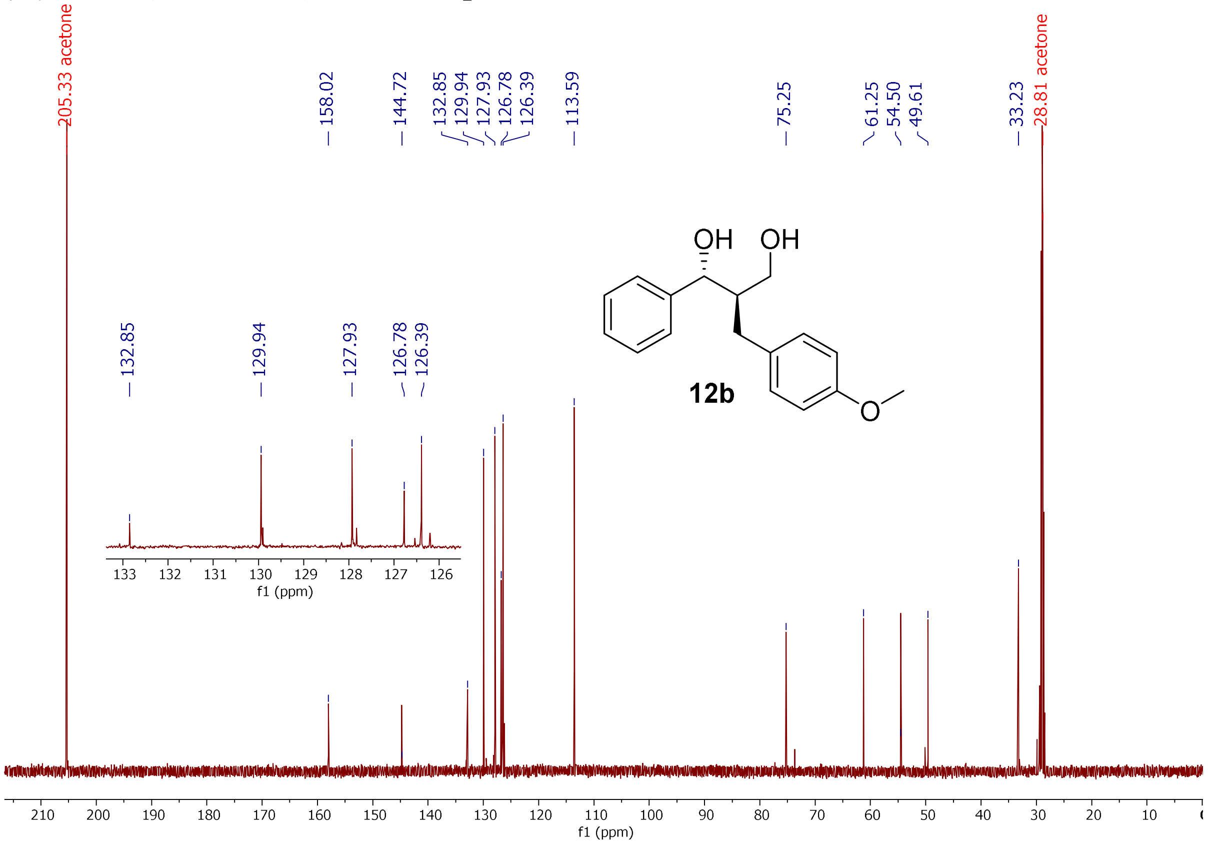

^{13}C NMR (126 MHz, $(CD_3)_2CO$) δ 158.0, 144.7, 132.8, 129.9, 127.9, 126.8, 126.4, 113.6, 75.2, 61.2, 54.5, 49.6, 33.2.

^{1}H NMR (500 MHz, $(CD_3)_2CO$) spectrum of 12c

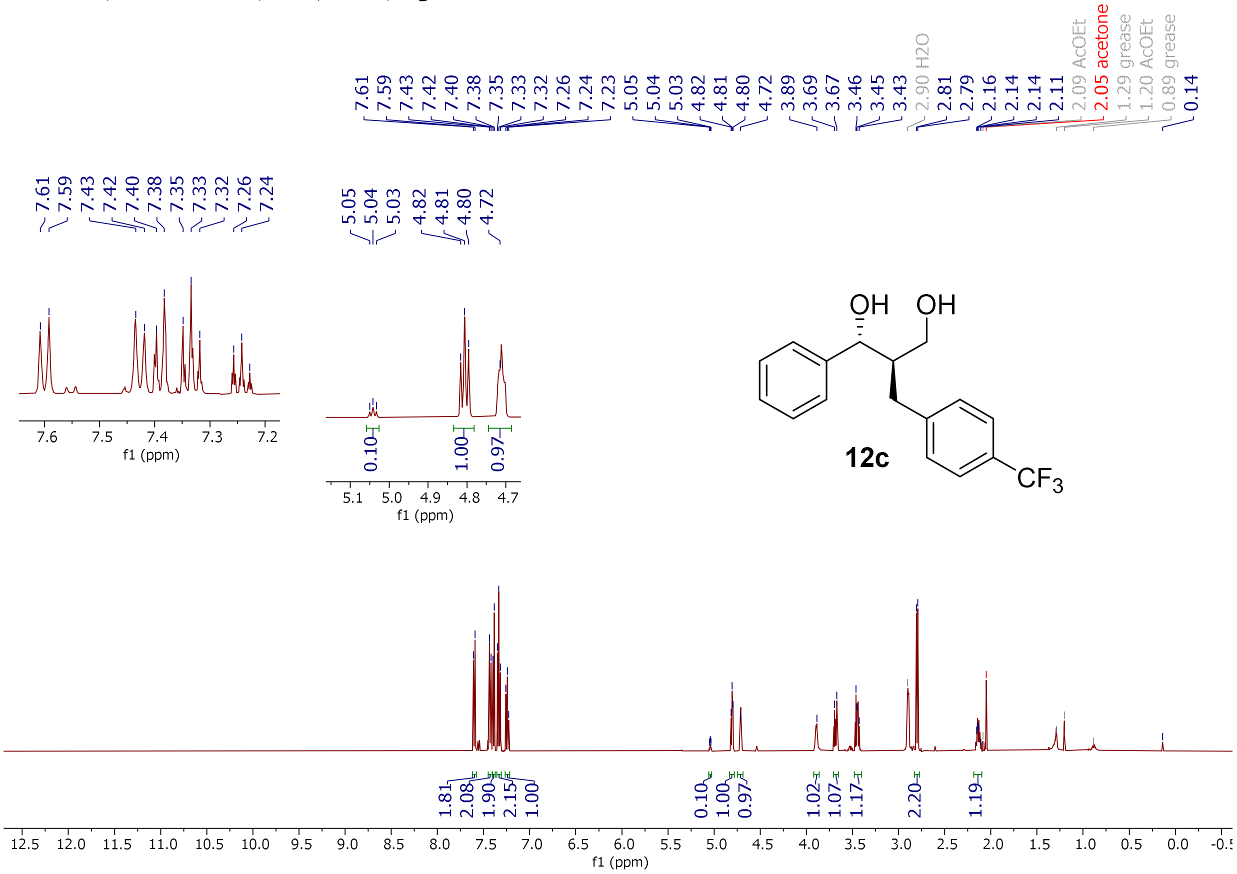

^{1}H NMR (500 MHz, $(CD_3)_2CO$, **only majority diastereoisomer was identified**) δ 7.60 (d, J = 7.8 Hz, 2H), 7.43 (d, J = 7.8 Hz, 2H), 7.39 (d, J = 7.0 Hz, 2H), 7.33 (t, J = 7.6 Hz, 2H), 7.24 (t, J = 7.3 Hz, 1H), 5.04 (t, J = 4.4 Hz, 0H), 4.81 (t, J = 5.1 Hz, 1H), 4.72 (s, 1H), 3.89 (s, 1H), 3.68 (d, J = 10.9 Hz, 1H), 3.48 – 3.40 (m, 1H), 2.80 (d, J = 7.5 Hz, 2H), 2.19 – 2.10 (m, 1H). Diastereomeric ratio: 91:9 (anti/syn).

^{13}C{H} NMR (126 MHz, $(CD_3)_2CO$) spectrum of 12c

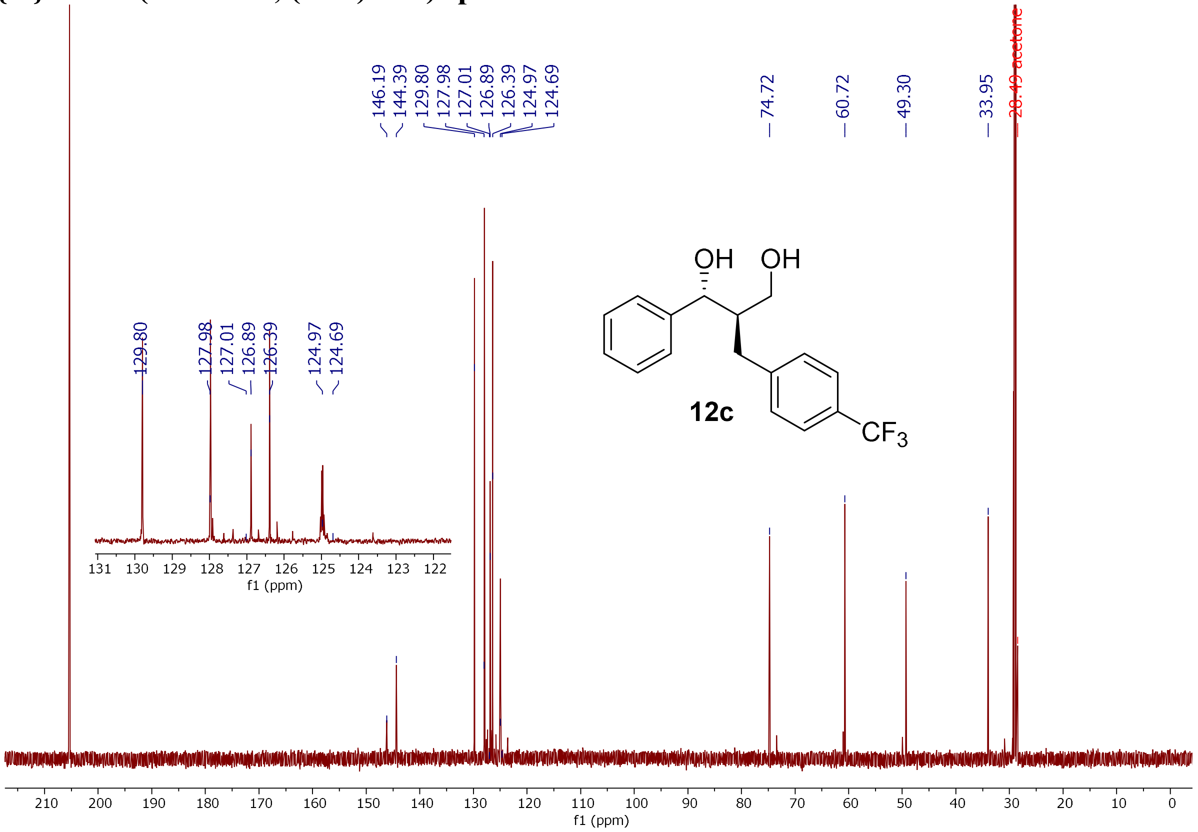

^{13}C NMR (126 MHz, $(CD_3)_2CO$) δ 146.2, 144.4, 129.8, 128.0, 127.0 (q, J = 86.4 Hz), 126.9, 126.4, 125.0 (q, J = 3.8 Hz), 124.7 (q, J = 270 Hz), 74.7, 60.7, 49.3, 33.9.

^{1}H NMR (400 MHz, $CDCl_3$) spectrum of 12d

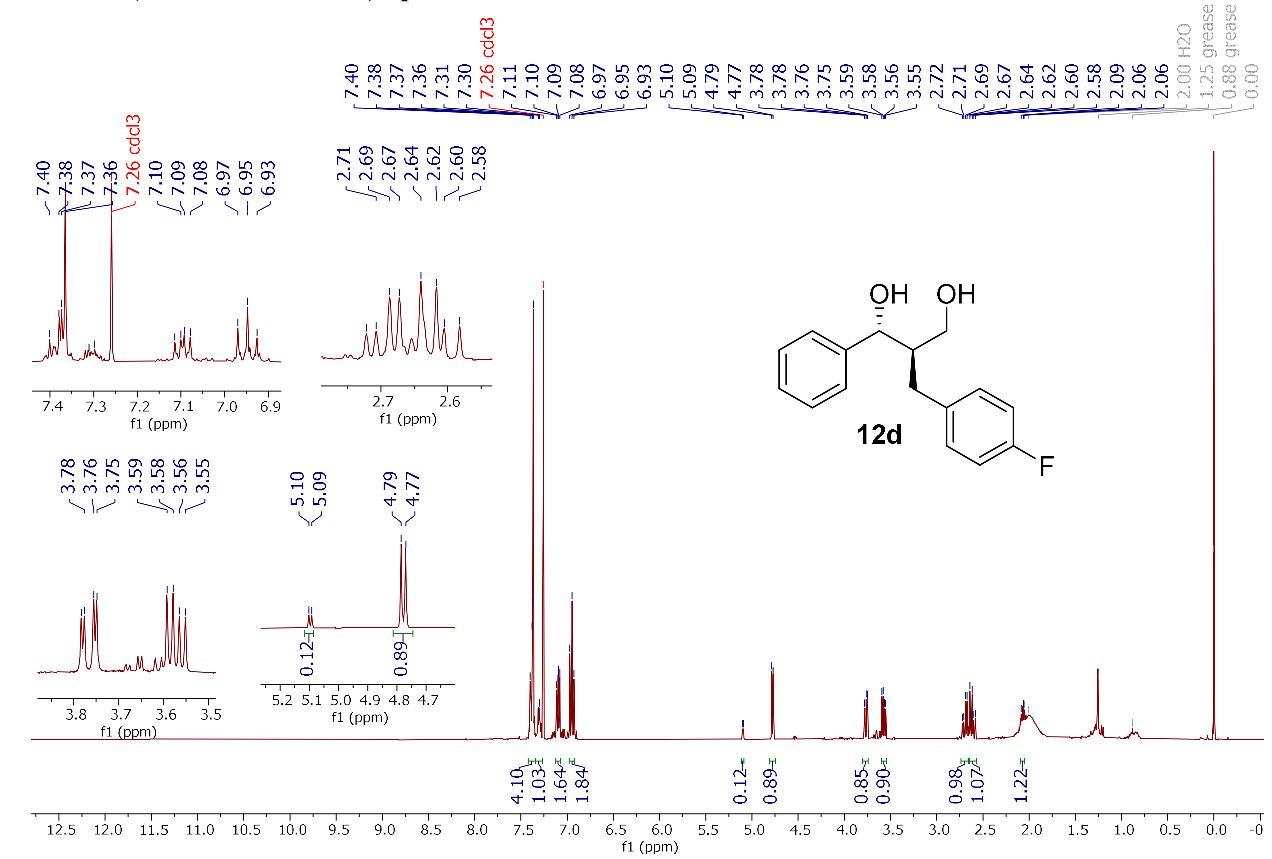

^{1}H NMR (400 MHz, $CDCl_3$, **only majority diastereoisomer was identified**) δ 7.41 – 7.34 (m, 4H), 7.33 – 7.27 (m, 1H), 7.10 (dd, J = 8.8, 5.4 Hz, 2H), 6.95 (t, J = 8.8 Hz, 2H), 5.10 (d, J = 4.2 Hz, 0H), 4.78 (d, J = 6.2 Hz, 1H), 3.77 (dd, J = 11.1, 2.7 Hz, 1H), 3.57 (dd, J = 11.0, 5.4 Hz, 1H), 2.70 (dd, J = 13.9, 6.0 Hz, 1H), 2.61 (dd, J = 13.8, 9.2 Hz, 1H), 2.13 – 2.02 (m, 1H). Diastereomeric ratio: 88:12 (anti/syn).

^{13}C{H} NMR (126 MHz, $(CD_3)_2CO$) spectrum of 12d

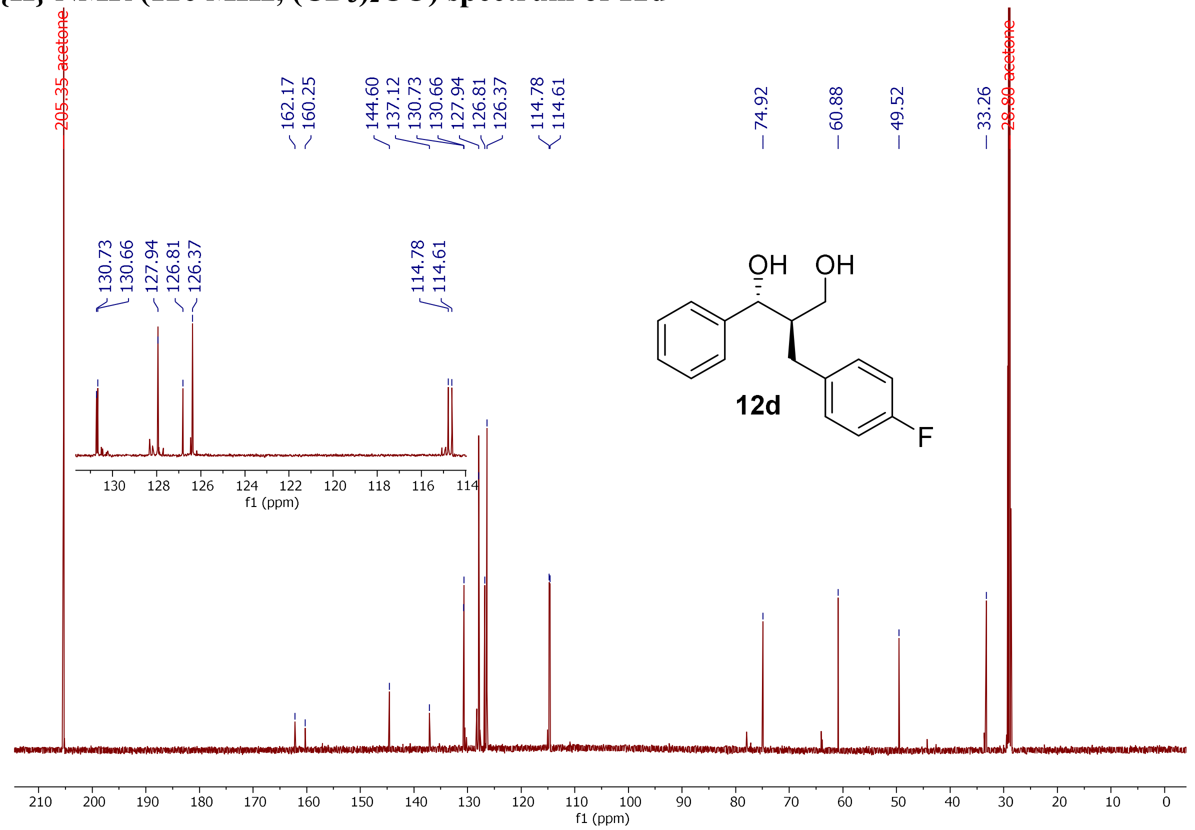

^{13}C NMR (126 MHz, $(CD_3)_2CO$) δ 161.2 (d, J = 241.3 Hz), 144.6, 137.1 (d, J = 3.3 Hz), 130.7 (d, J = 8.1 Hz), 127.9, 126.8, 126.4, 114.7 (d, J = 21.0 Hz), 74.9, 60.9, 49.5, 33.3.

^{1}H NMR (400 MHz, $(CD_3)_2CO$) spectrum of 12e

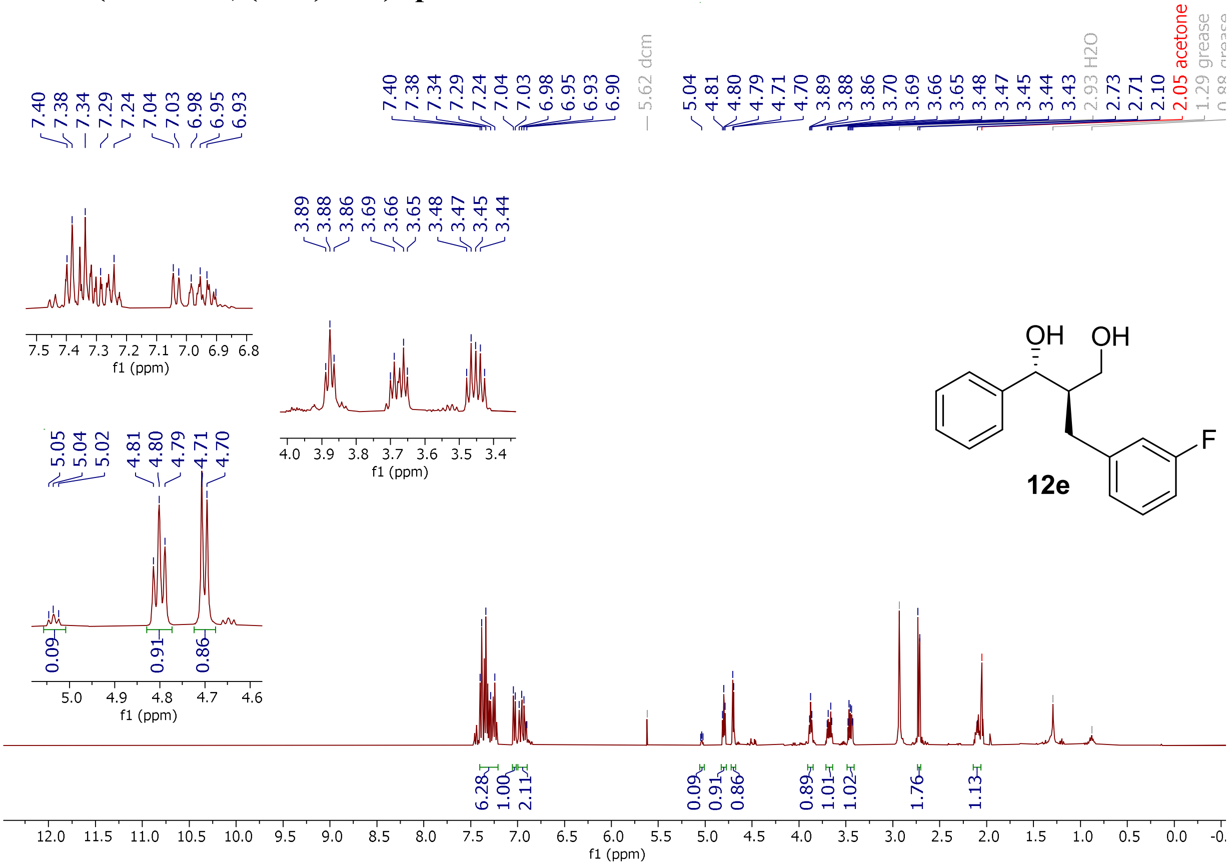

^{1}H NMR (400 MHz, $(CD_3)_2CO$, **only majority diastereoisomer was identified**) δ 7.42 – 7.18 (m, 6H), 7.10 – 6.85 (m, 3H), 5.04 (t, J = 4.5 Hz, 0H), 4.80 (t, J = 5.0 Hz, 1H), 4.70 (d, J = 4.6 Hz, 1H), 3.88 (t, J = 4.9 Hz, 1H), 3.73 – 3.62 (m, 1H), 3.45 (m, 1H), 2.72 (d, J = 7.8 Hz, 2H), 2.10 (m, 1H). Diastereomeric ratio: 91:9 (anti/syn).

^{13}C{H} NMR (126 MHz, $(CD_3)_2CO$) spectrum of 12e

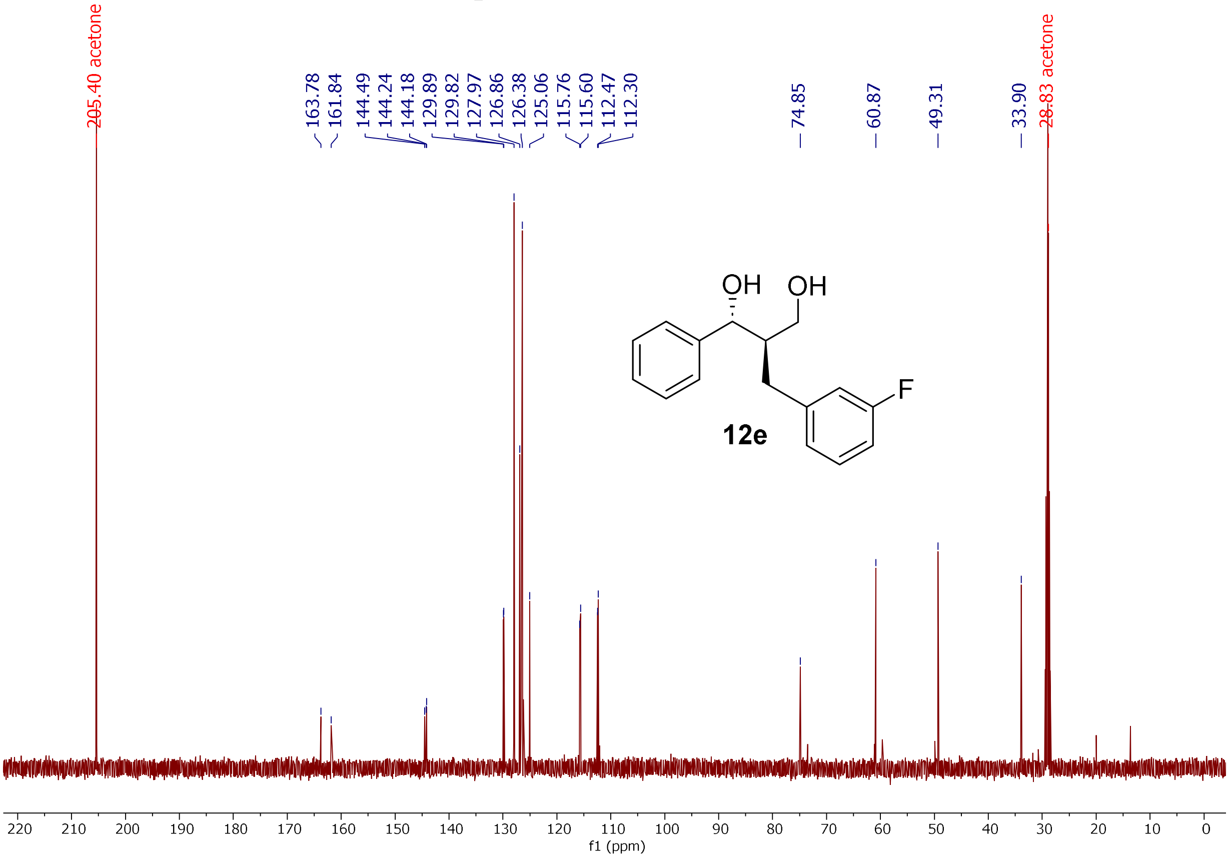

^{13}C NMR (126 MHz, $(CD_3)_2CO$) δ 205.4, 162.8 (d, J = 243.2 Hz), 144.5, 144.2 (d, J = 7.2 Hz), 129.9 (d, J = 8.6 Hz), 128.0, 126.9, 126.4, 125.1, 115.7 (d, J = 21.0 Hz), 112.4 (d, J = 21.5 Hz)., 74.8, 60.9, 49.3, 33.9, 28.8.

^{1}H NMR (400 MHz, $(CD_3)_2CO$) spectrum of 12f

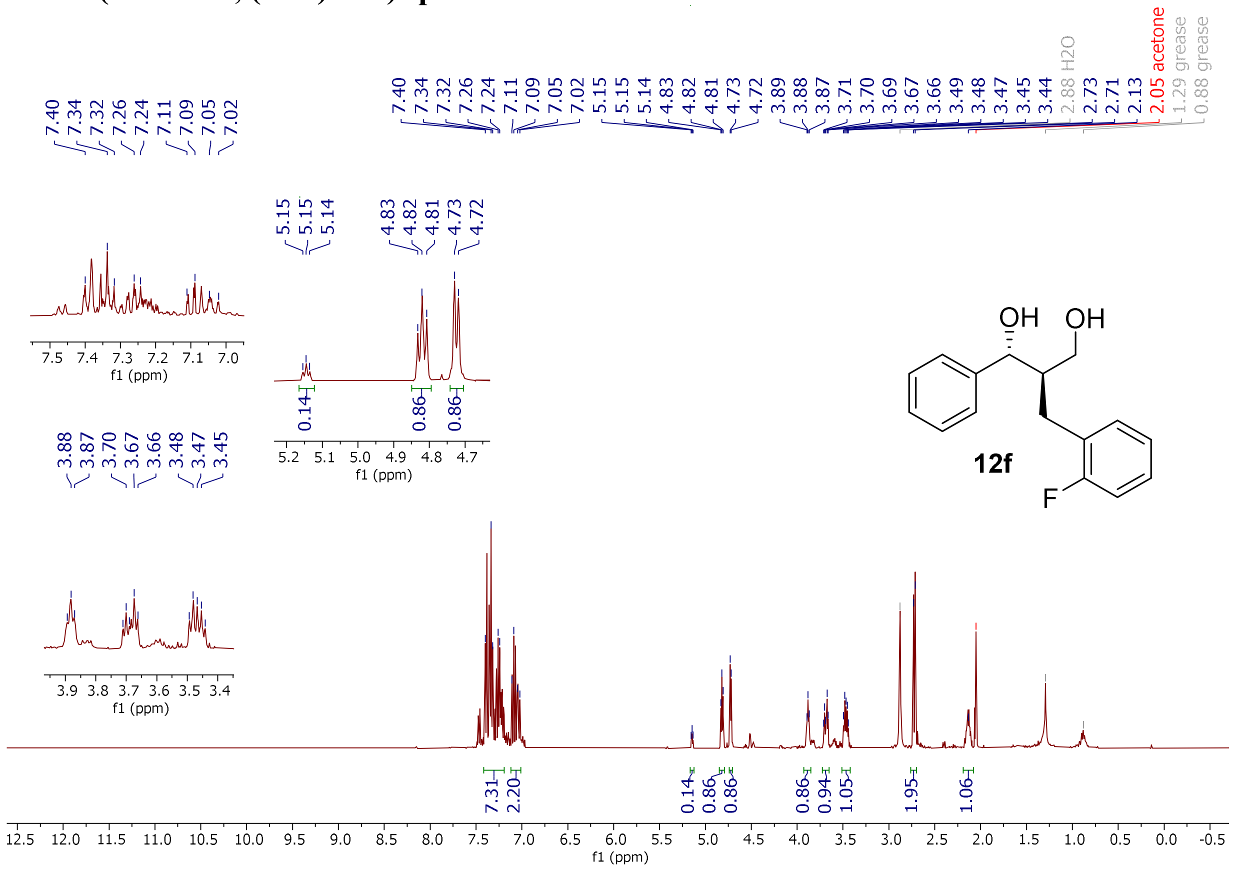

^{1}H NMR (400 MHz, $(CD_3)_2CO$, **only majority diastereoisomer was identified**) δ 7.43 – 7.18 (m, 7H), 7.15 – 6.99 (m, 2H), 5.15 (t, J = 4.2 Hz), 4.82 (t, J = 5.0 Hz, 1H), 4.72 (d, J = 4.5 Hz, 1H), 3.88 (t, J = 4.9 Hz, 1H), 3.75 – 3.62 (m, 1H), 3.53 – 3.40 (m, 1H), 2.72 (d, J = 6.2 Hz, 2H), 2.21 – 2.09 (m, 1H). Diastereomeric ratio: 86:14 (anti/syn).

^{13}C{H} NMR (101 MHz, $(CD_3)_2CO$) spectrum of 12f

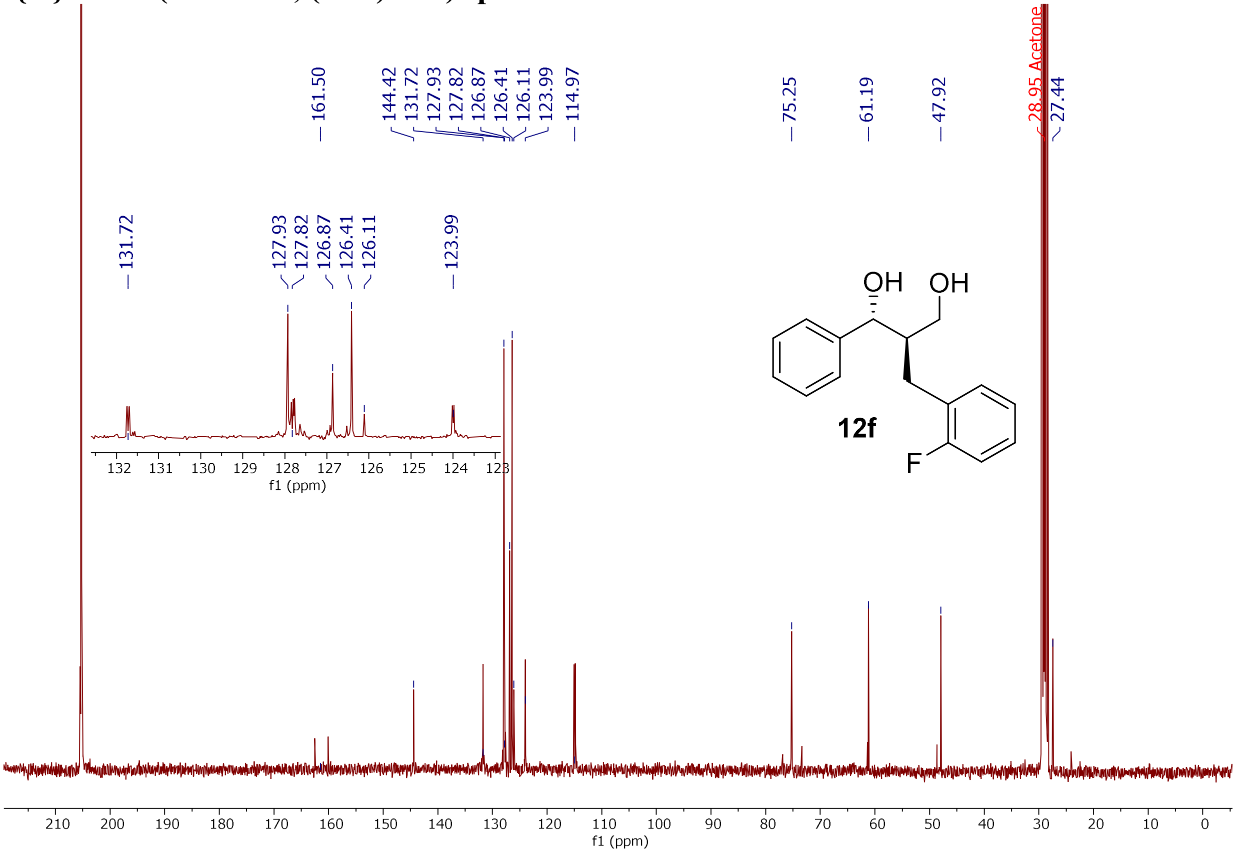

^{13}C NMR (101 MHz, $(CD_3)_2CO$) δ 161.3 (d, J = 243.6 Hz), 144.4, 131.7 (d, J = 5.0 Hz), 127.9, 127.8 (d, J = 4.8 Hz), 126.9, 126.4, 126.1, 124.0 (d, J = 3.5 Hz), 115.0 (d, J = 22.4 Hz), 75.2, 61.2, 47.9, 27.4.

^{1}H NMR (500 MHz, $(CD_3)_2CO$) spectrum of 12g

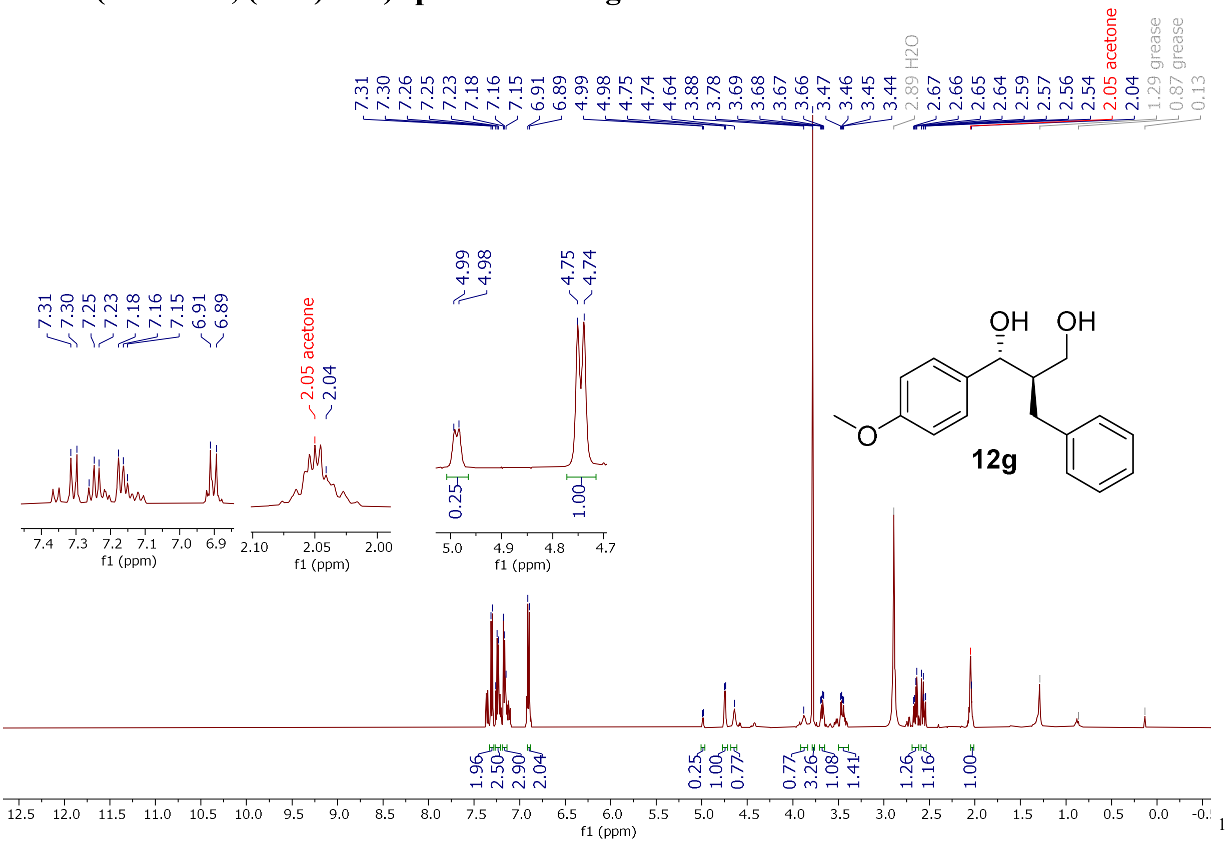

^{1}H NMR (500 MHz, $(CD_3)_2CO$, **only majority diastereoisomer was identified**) δ 7.31 (d, J = 8.6 Hz, 2H), 7.25 (m, 2H), 7.19 – 7.14 (m, 3H), 6.90 (d, J = 8.7 Hz, 2H), 4.99 (d, J = 5.0 Hz, 0H), 4.75 (d, J = 6.4 Hz, 1H), 4.64 (s, 1H), 3.88 (s, 1H), 3.78 (s, 3H), 3.68 (dd, J = 10.7, 3.9 Hz, 1H), 3.46 (dd, J = 10.7, 5.4 Hz, 1H), 2.65 (dd, J = 13.5, 5.6 Hz, 1H), 2.56 (dd, J = 13.6, 9.2 Hz, 1H), 2.04 (s, 1H). Diastereomeric ratio 80:20 (anti/syn).

^{13}C{H} NMR (126 MHz, $(CD_3)_2CO$) spectrum of 12g

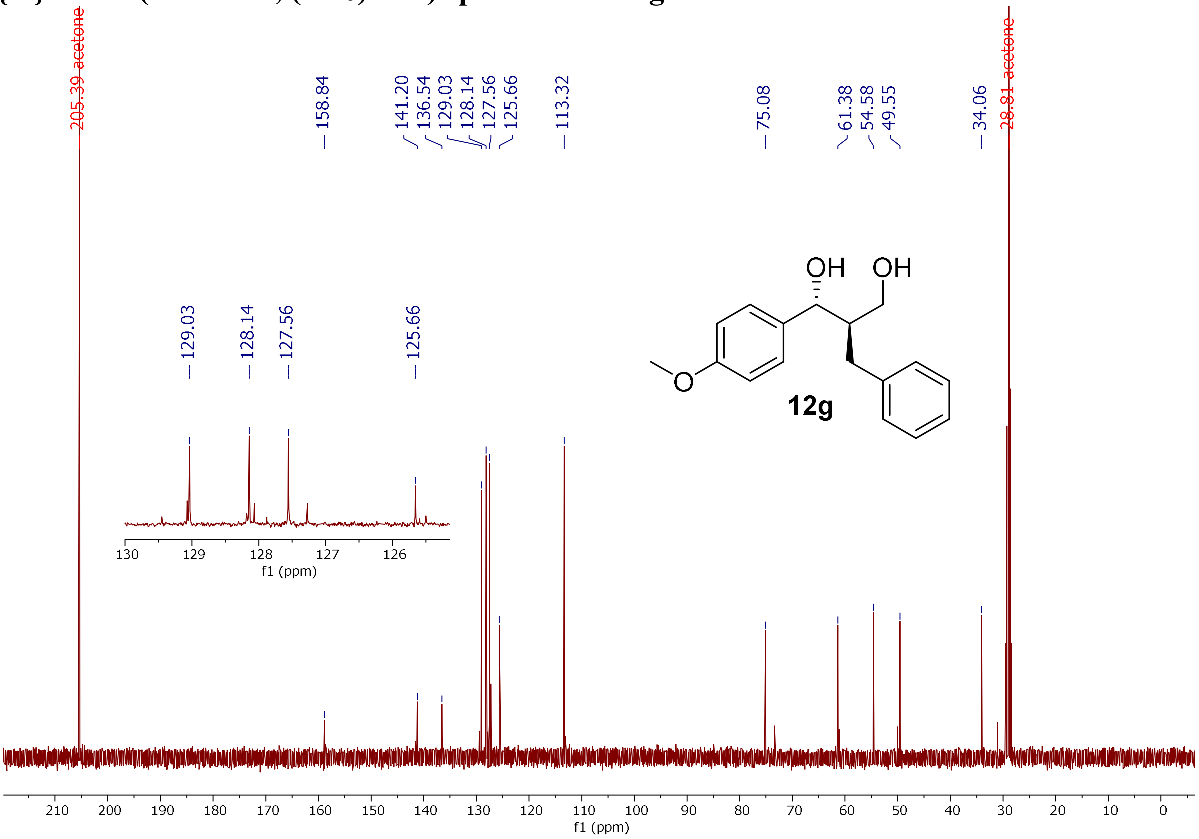

^{13}C NMR (126 MHz, $(CD_3)_2CO$) δ 158.8, 141.2, 136.5, 129.0, 128.1, 127.6, 125.7, 113.3, 75.1, 61.4, 54.6, 49.5, 34.1.

^{1}H NMR (400 MHz, $CDCl_3$) spectrum of 12h

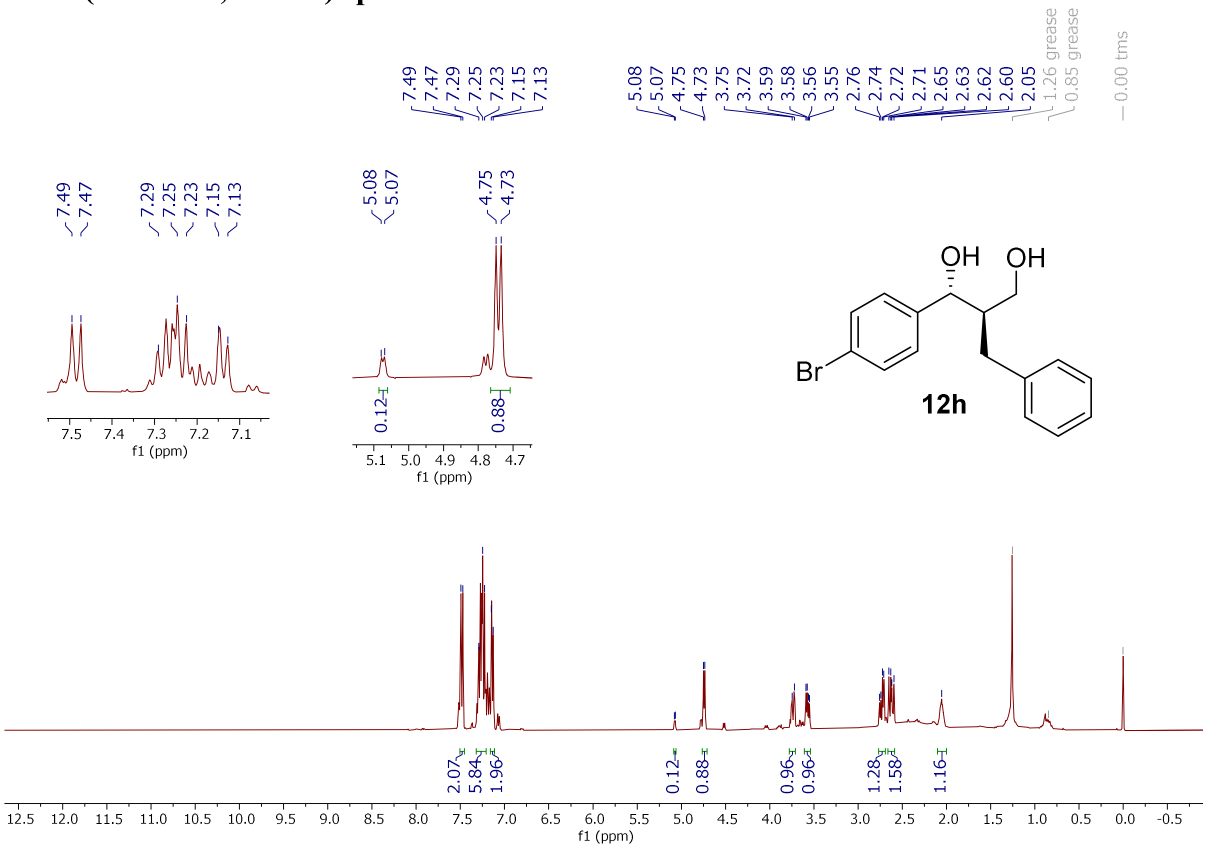

^{1}H NMR (400 MHz, $CDCl_3$, **only majority diastereoisomer was identified**) δ 7.48 (d, J = 8.4 Hz, 2H), 7.33 – 7.19 (m, 5H), 7.14 (d, J = 8.8 Hz, 2H), 5.07 (d, J = 3.9 Hz, 0H), 4.74 (d, J = 5.9 Hz, 1H), 3.73 (d, J = 10.6 Hz, 1H), 3.57 (dd, J = 11.1, 5.3 Hz, 1H), 2.73 (dd, J = 13.7, 5.9 Hz, 1H), 2.62 (dd, J = 14.0, 9.3 Hz, 1H), 2.10 – 1.95 (m, 1H). Diastereomeric ratio: 88:12 (anti/syn).

^{13}C{H} NMR (101 MHz, $(CD_3)_2CO$) spectrum of 12h

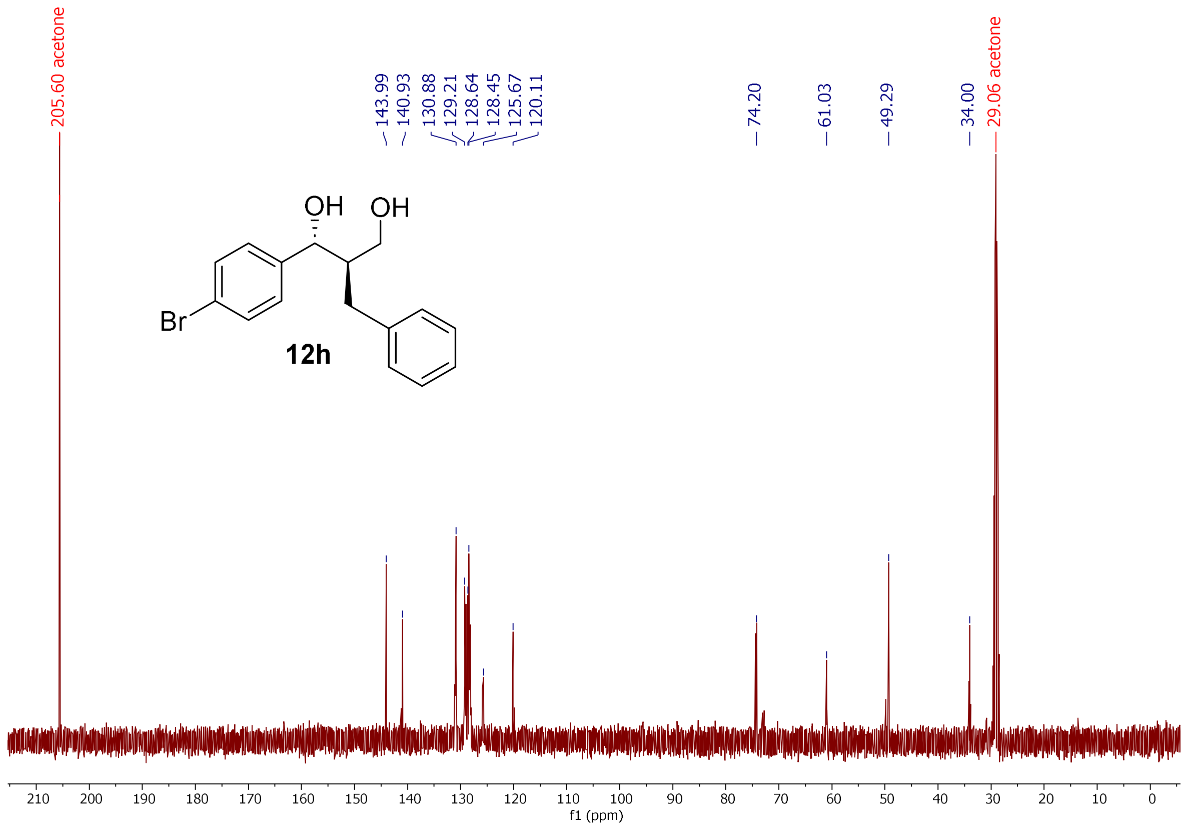

^{13}C NMR (101 MHz, $(CD_3)_2CO$) δ 144.0, 140.9, 130.9, 129.2, 128.6, 128.4, 125.7, 120.1, 74.2, 61.0, 49.3, 34.0.

^{1}H NMR (400 MHz, $CDCl_3$) spectrum of 12i

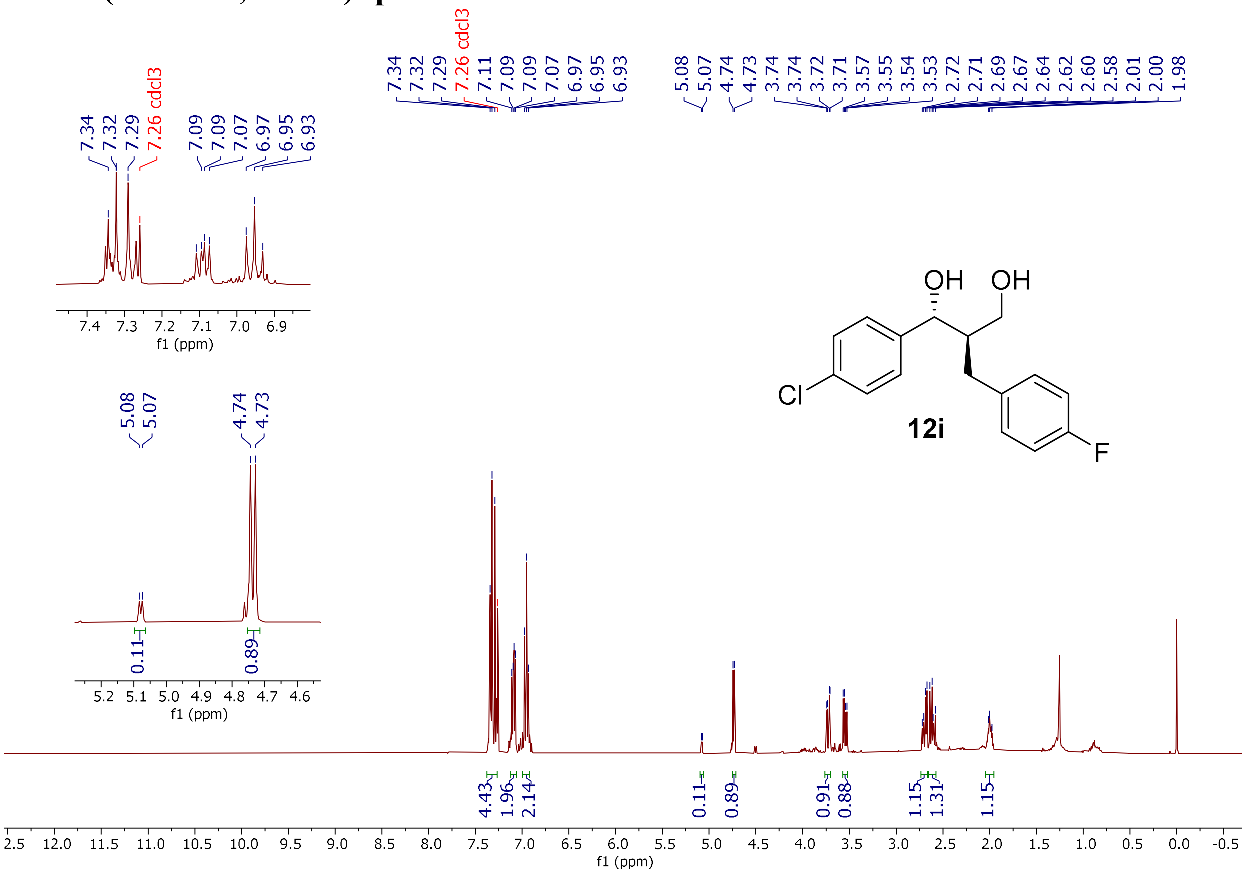

^{1}H NMR (400 MHz, $CDCl_3$,, **only majority diastereoisomer was identified**) δ 7.37 – 7.24 (m, 4H), 7.09 (dd, *J* = 8.8, 5.4 Hz, 2H), 6.95 (t, *J* = 8.8 Hz, 2H), 5.08 (d, *J* = 3.8 Hz, 0H), 4.74 (d, *J* = 5.9 Hz, 1H), 3.73 (dd, *J* = 10.9, 2.8 Hz, 1H), 3.55 (dd, *J* = 10.9, 5.3 Hz, 1H), 2.70 (dd, *J* = 13.9, 6.2 Hz, 1H), 2.61 (dd, *J* = 13.9, 9.2 Hz, 1H), 2.05 – 1.94 (m, 1H). Diastereomeric ratio: 89:11 (anti/syn).

$^{13}C\{H\}$ NMR (126 MHz, $(CD_3)_2CO$) spectrum of 12i

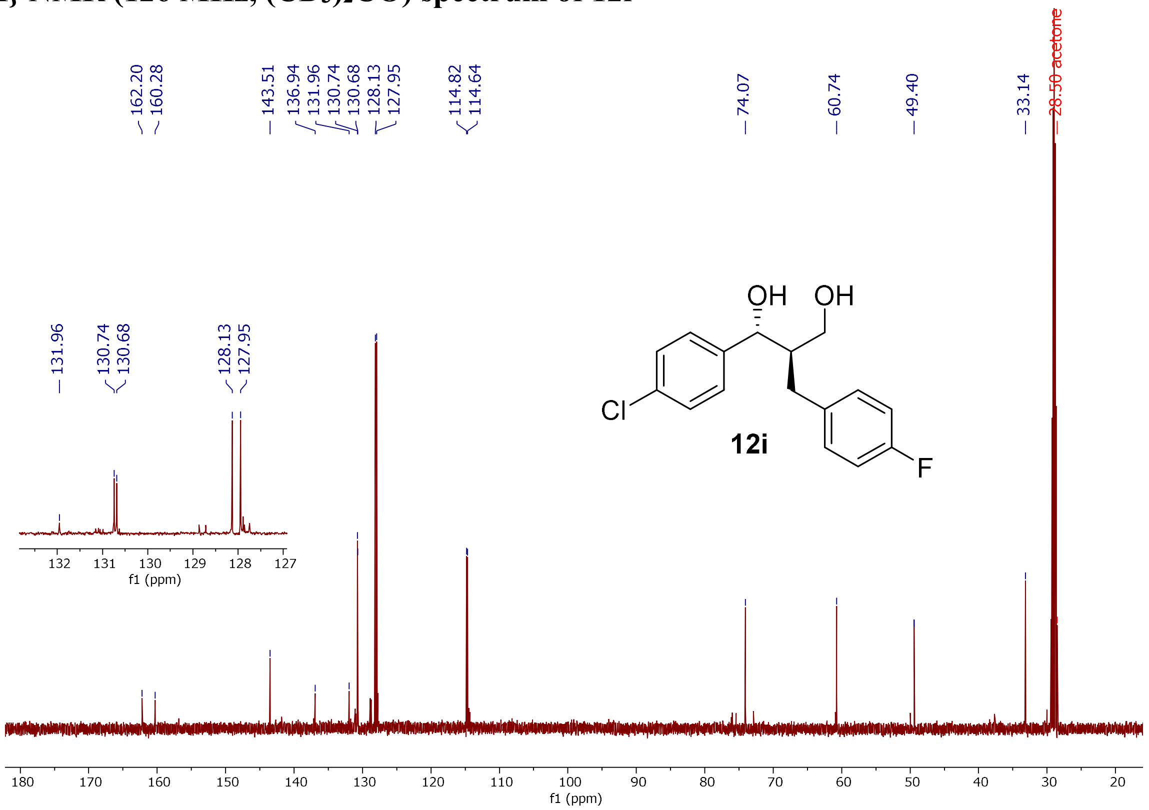

^{13}C NMR (126 MHz, $(CD_3)_2CO$) δ 161.1 (d, *J* = 241.8 Hz), 143.5, 136.9, 132.0, 130.7 (d, *J* = 7.6 Hz), 128.1, 127.9, 114.7 (d, *J* = 21.5 Hz), 74.1, 60.7, 49.4, 33.1.

^{1}H NMR (500 MHz, $(CD_3)_2CO$) spectrum of 12j

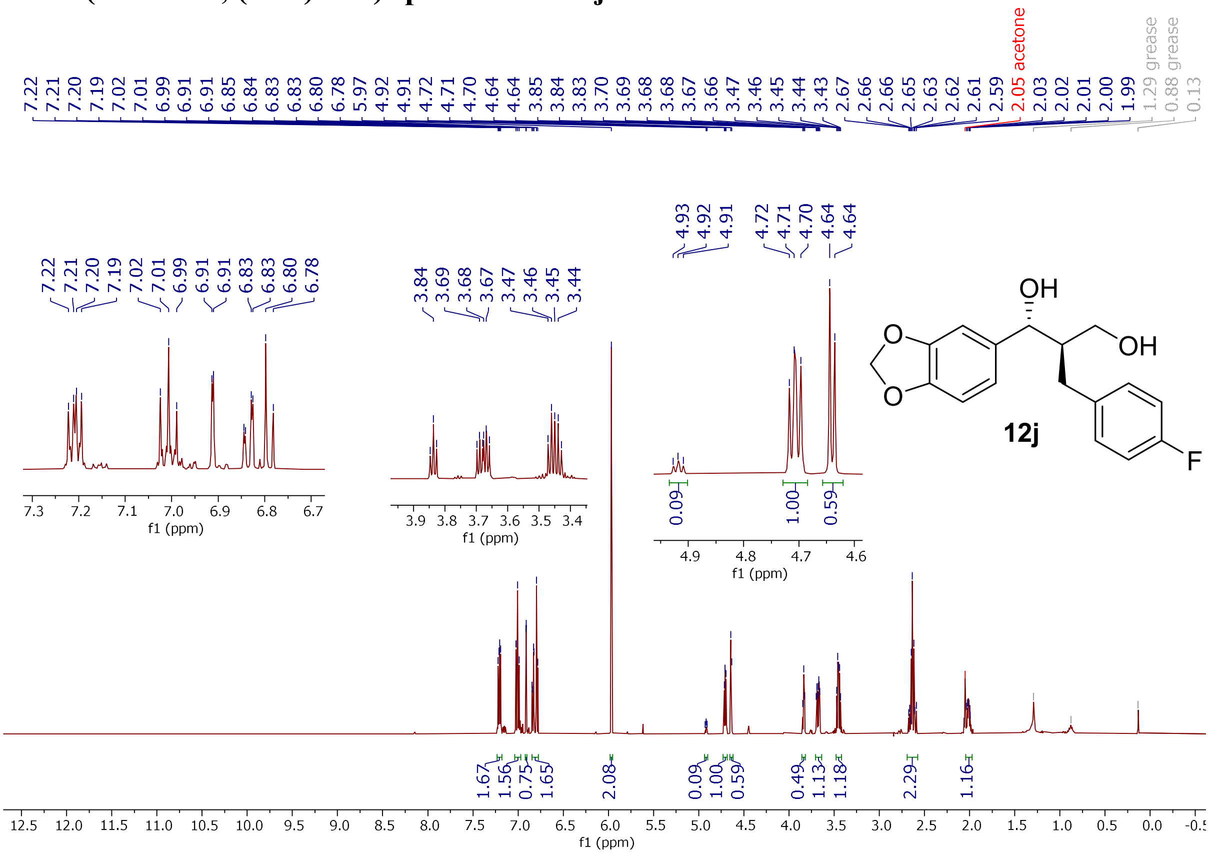

^{1}H NMR (500 MHz, $(CD_3)_2CO$, **only majority diastereoisomer was identified**) δ 7.21 (dd, J = 8.7, 5.5 Hz, 2H), 7.01 (t, J = 8.9 Hz, 2H), 6.91 (d, J = 1.8 Hz, 1H), 6.85 – 6.78 (m, 2H), 5.97 (s, 2H), 4.92 (t, J = 4.5 Hz, 0H), 4.71 (t, J = 4.3 Hz, 1H), 4.64 (d, J = 4.4 Hz, 1H), 3.68 (dt, J = 10.6, 4.5 Hz, 1H), 3.45 (dt, J = 10.5, 5.1 Hz, 1H), 2.69 – 2.57 (m, 2H), 2.04 – 1.97 (m, 1H). Diastereomeric ratio: 92:8 (anti/syn).

^{13}C{H} NMR (126 MHz, $(CD_3)_2CO$) spectrum of 12j

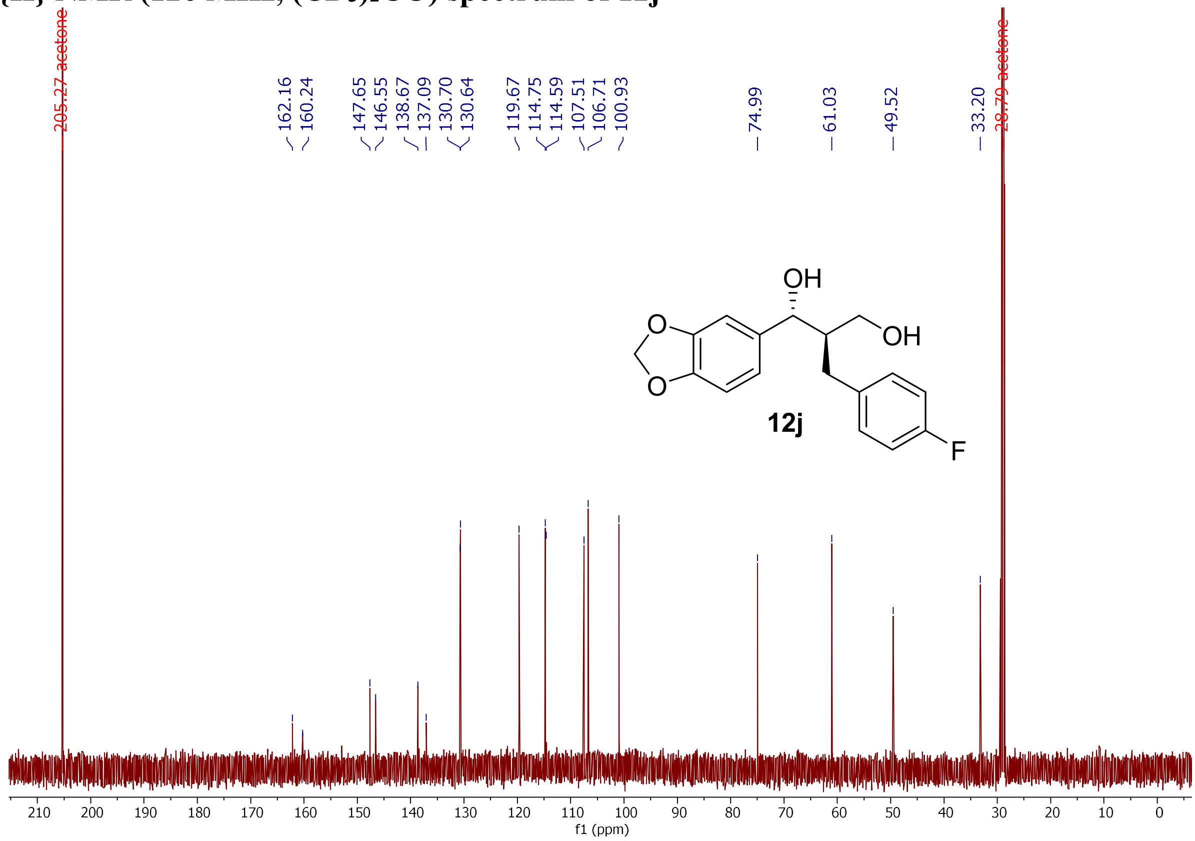

^{13}C NMR (126 MHz, $(CD_3)_2CO$) δ 161.2 (d, J = 241.3 Hz), 147.6, 146.5, 138.7, 137.1, 130.7 (d, J = 8.1 Hz), 119.7, 114.7 (d, J = 21.0 Hz), 107.5, 106.7, 100.9, 75.0, 61.0, 49.5, 33.2.

^{1}H NMR (500 MHz, $(CD_3)_2CO$) spectrum of 5a

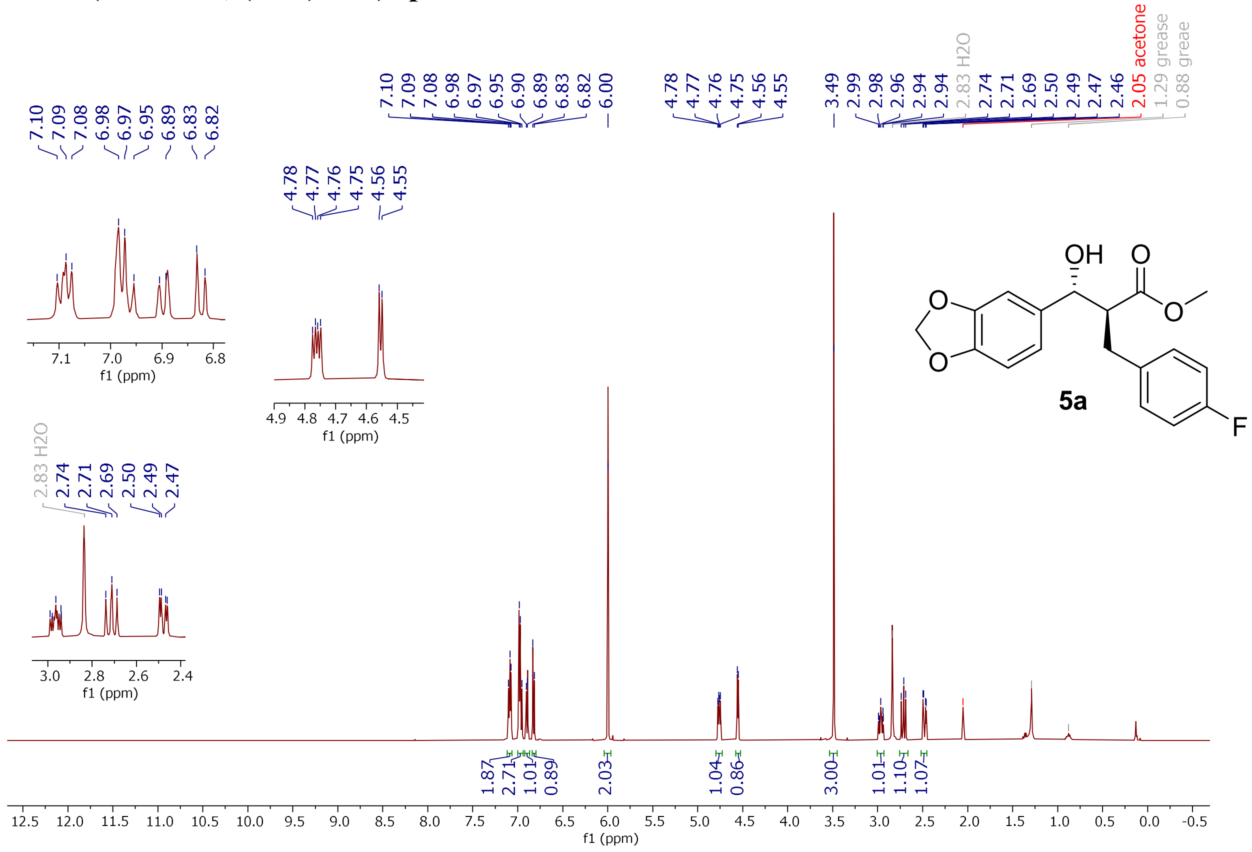

^{13}C{H} NMR (126 MHz, $(CD_3)_2CO$) spectrum of 5a

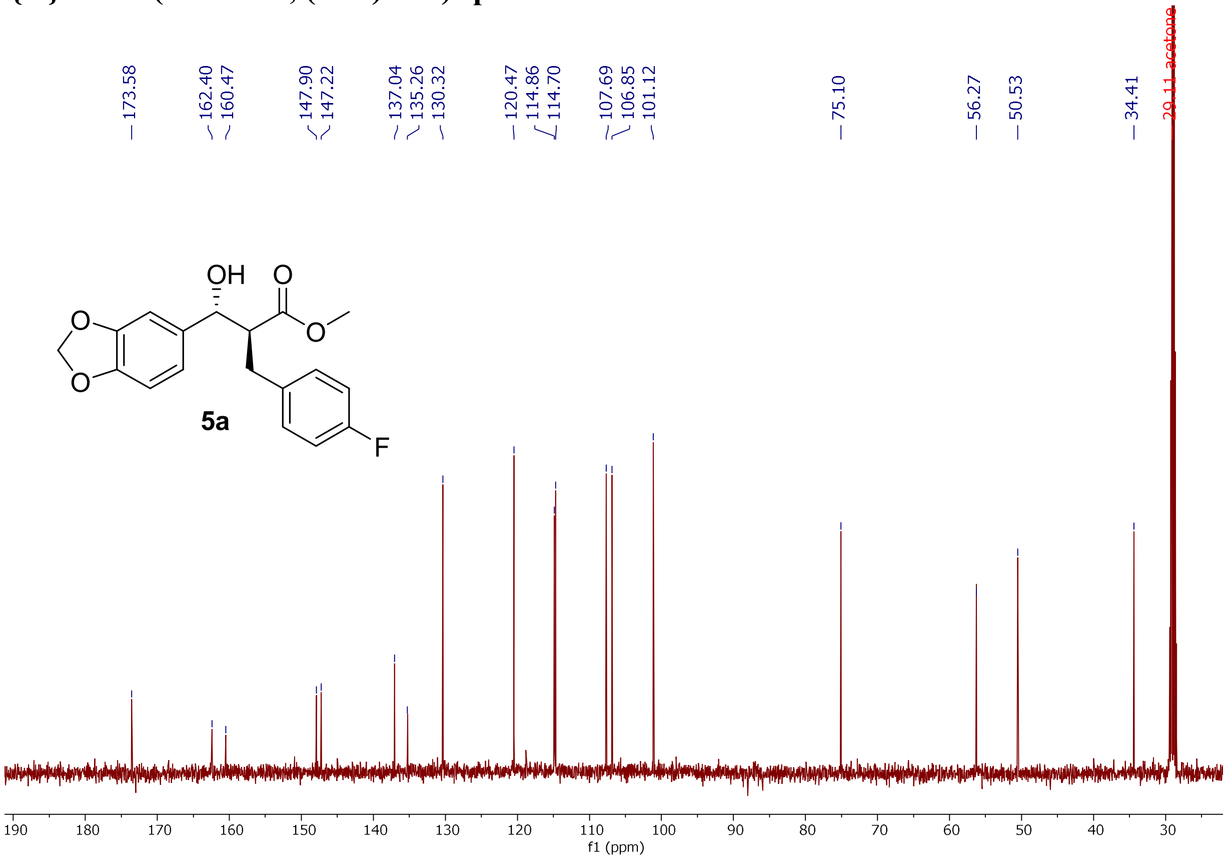

.

^{1}H NMR (500 MHz, $CDCl_3$) spectrum of 16

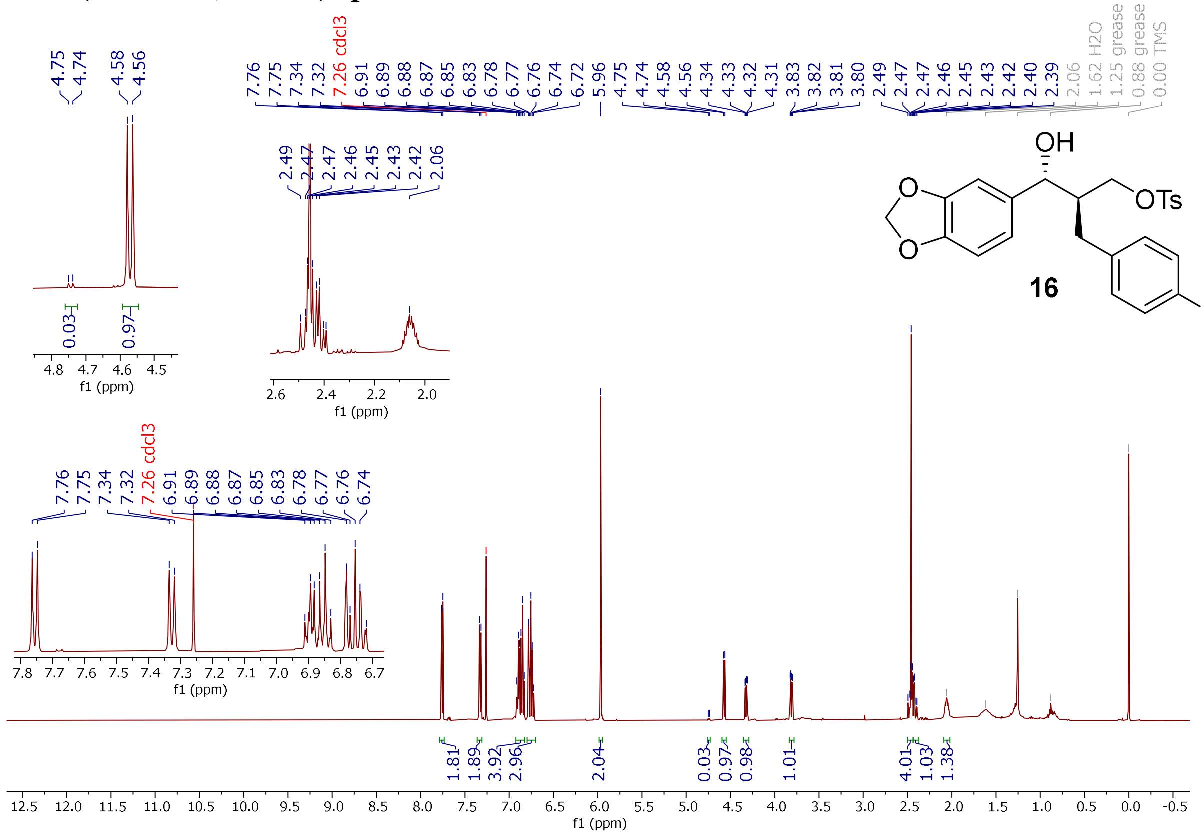

^{1}H NMR (500 MHz, $CDCl_3$) δ 7.76 (d, J = 8.4 Hz, 2H), 7.33 (d, J = 7.9 Hz, 2H), 6.93 – 6.83 (m, 4H), 6.80 – 6.70 (m, 3H), 5.96 (s, 2H), 4.74 (d, J = 6.5 Hz, 0H), 4.57 (d, J = 8.1 Hz, 1H), 4.32 (dd, J = 9.5, 3.8 Hz, 1H), 3.81 (dd, J = 9.7, 3.2 Hz, 1H), 2.50 – 2.44 (m, 4H), 2.41 (dd, J = 13.6, 5.3 Hz, 1H), 2.06 (s, 1H). Diastereomeric ratio: 97:3 (anti/syn)

$^{13}C\{H\}$ NMR (126 MHz, $CDCl_3$) spectrum of 16

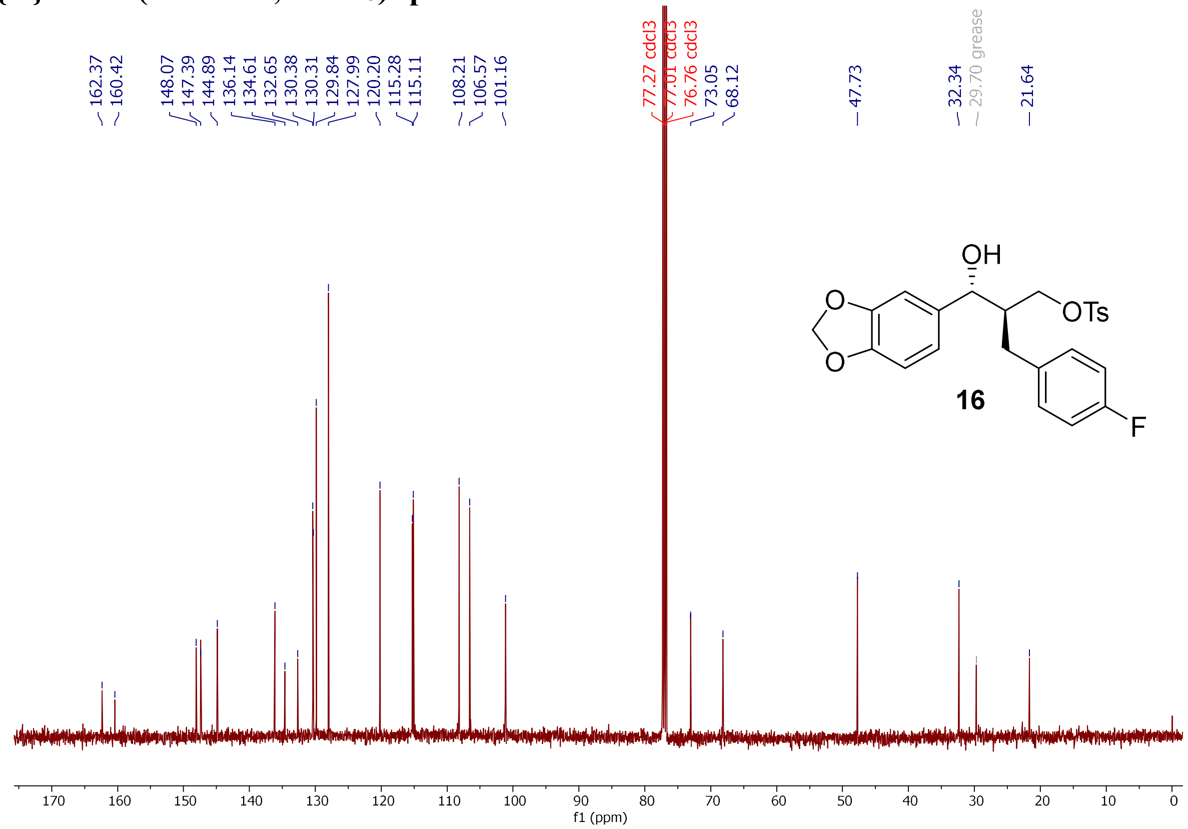

^{13}C NMR (126 MHz, $CDCl_3$) δ 161.4 (d, J = 244.6 Hz), 148.1, 147.4, 144.9, 136.1, 134.6 (d, J = 3.3 Hz), 132.6, 130.3 (d, J = 8.1 Hz), 129.8, 128.0, 120.2, 115.2 (d, J = 21.0 Hz), 108.2, 106.6, 101.2, 73.0, 68.1, 47.7, 32.3, 21.6.

^{1}H NMR (500 MHz, $(CD_3)_2CO$) spectrum of 17

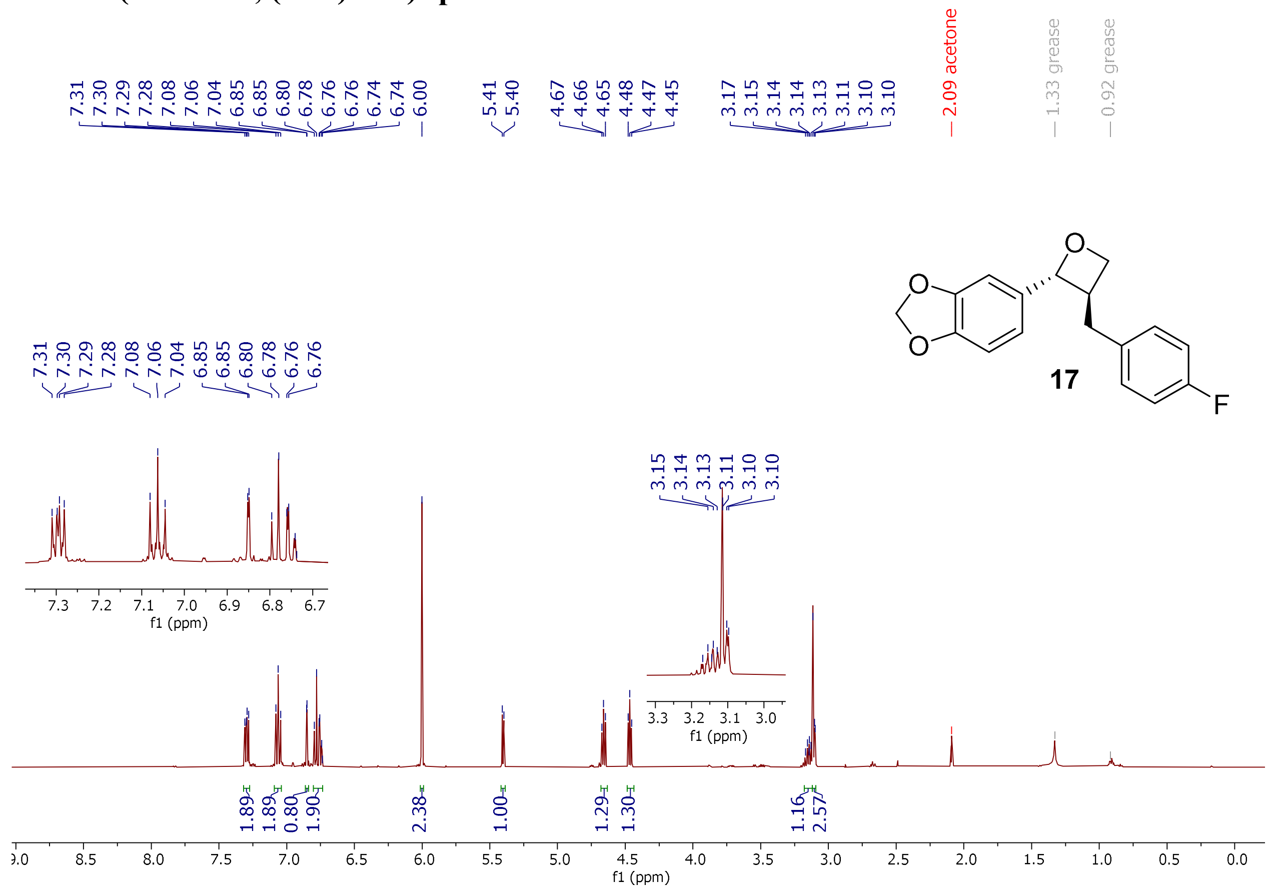

^{1}H NMR (500 MHz, $(CD_3)_2CO$) δ 7.30 (dd, J = 8.5, 5.8 Hz, 2H), 7.06 (t, J = 8.9 Hz, 2H), 6.85 (d, J = 1.6 Hz, 1H), 6.80 – 6.73 (m, 2H), 6.00 (s, 2H), 5.40 (d, J = 6.0 Hz, 1H), 4.68 – 4.63 (m, 1H), 4.47 (t, J = 6.3 Hz, 1H), 3.18 – 3.12 (m, 1H), 3.12 – 3.09 (m, 2H).

$^{13}C\{H\}$ NMR (126 MHz, $(CD_3)_2CO$) spectrum of 17

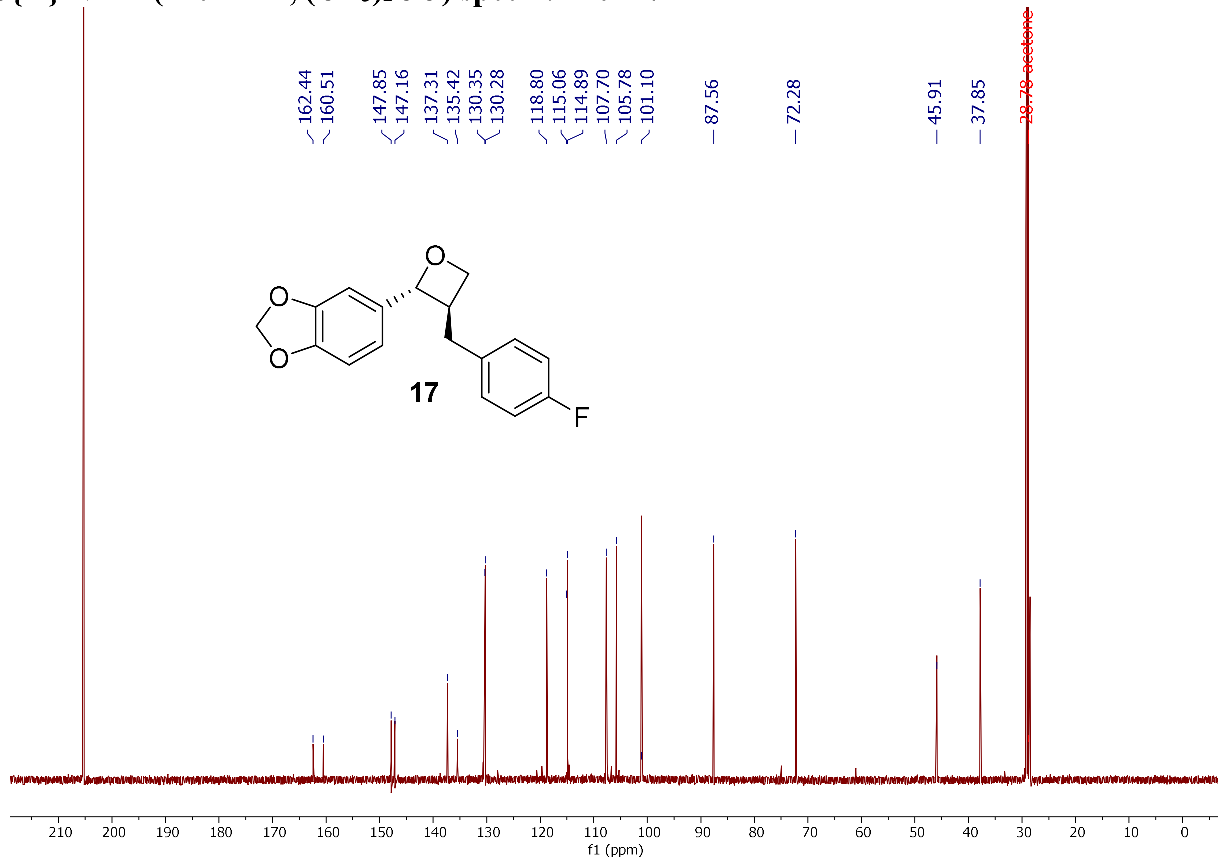

^{13}C NMR (126 MHz, $(CD_3)_2CO$) δ 161.47 (d, J = 242.2 Hz), 147.85, 147.16, 137.31, 135.43 (d, J = 3.3 Hz), 130.31 (d, J = 8.1 Hz), 118.80, 114.98 (d, J = 21.5 Hz), 107.70, 105.78, 101.10, 87.56, 72.28, 45.91, 37.85.

NOESY (500 MHz, $(CD_3)_2CO$)) spectrum of 17

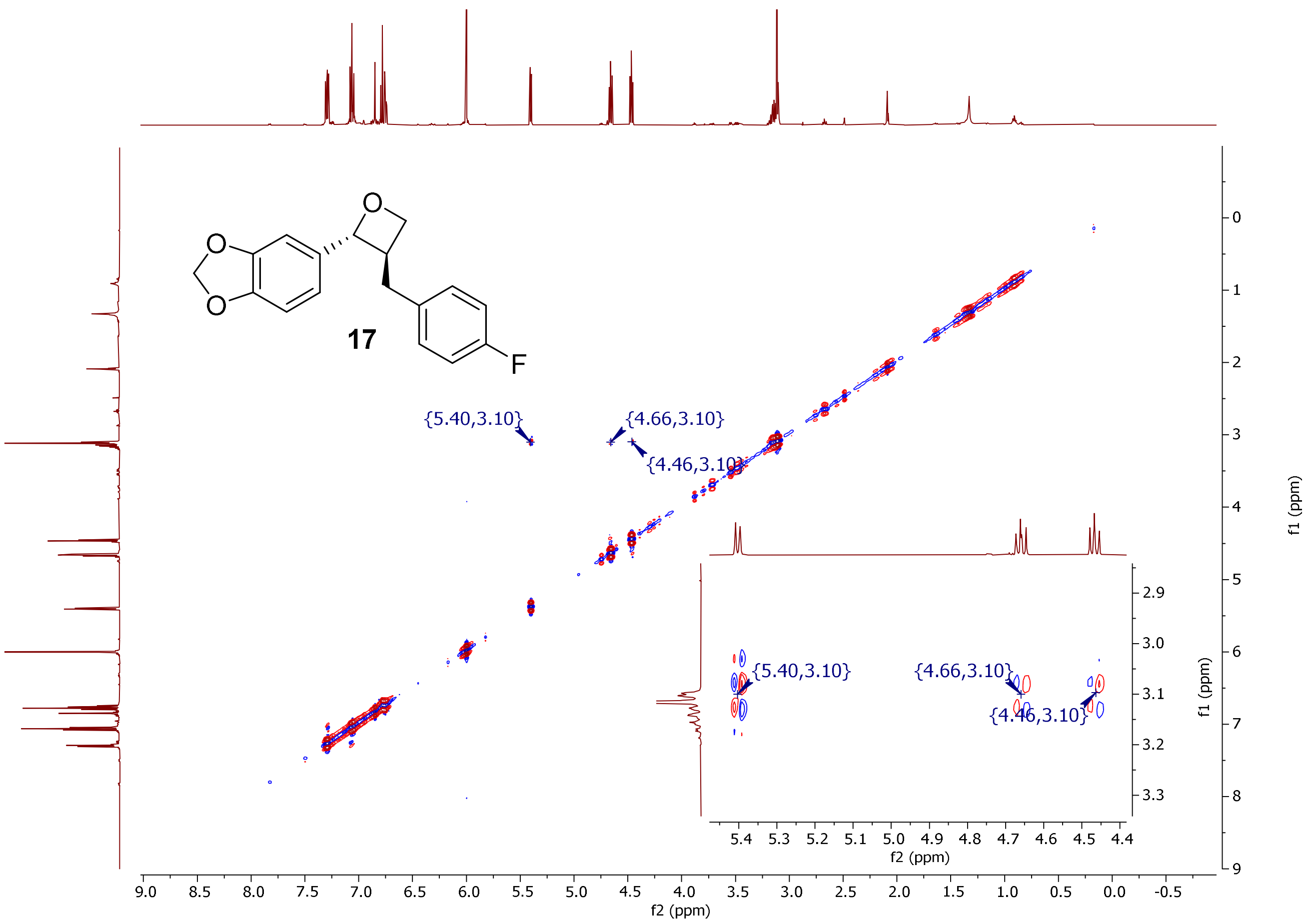

Chromatograms of compounds 12a-j, 5a, 16, 17

methyl (2S,3R)-3-(benzo[d][1,3]dioxol-5-yl)-2-(4-fluorobenzyl)-3-hydroxypropanoate:

<Chromatogram>

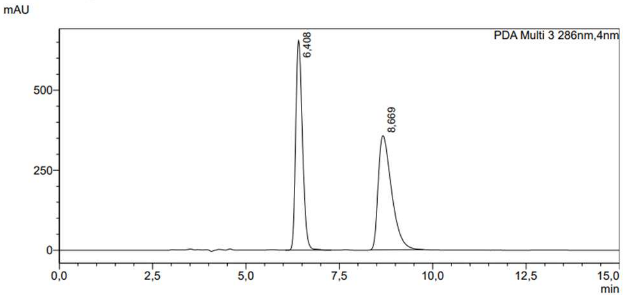

<Peak Table>

PDA Ch3 286nm

Peak#	Ret. Time	Area	Height	Area%	Height%
1	6,408	8239034	654686	48,095	64,743
2	8,669	8891798	356515	51,905	35,257
Total		17130832	1011201	100,000	100,000

<Chromatogram>

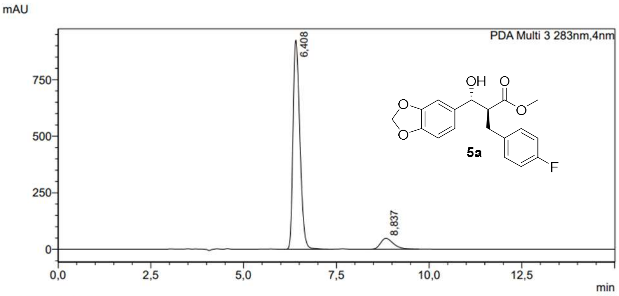

<Peak Table>

PDA Ch3 283nm

Peak#	Ret. Time	Area	Height	Area%	Height%
1	6,408	11892002	922802	91,089	95,043
2	8,837	1163305	48125	8,911	4,957
Total		13055307	970927	100,000	100,000

(1R,2R)-1-(benzo[d][1,3]dioxol-5-yl)-2-(4-fluorobenzyl)propane-1,3-diol:

<Chromatogram>

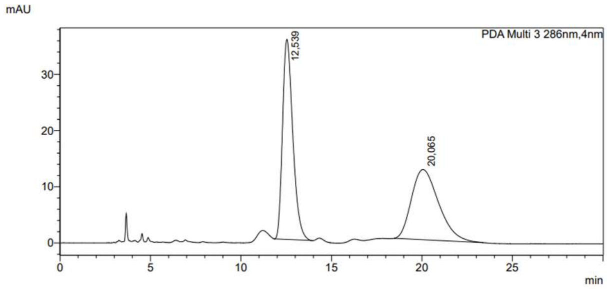

<Peak Table>

PDA Ch3 286nm

Peak#	Ret. Time	Area	Height	Area%	Height%
1	12,539	1408776	35628	51,849	74,007
2	20,065	1308314	12514	48,151	25,993
Total		2717091	48141	100,000	100,000

<Chromatogram>

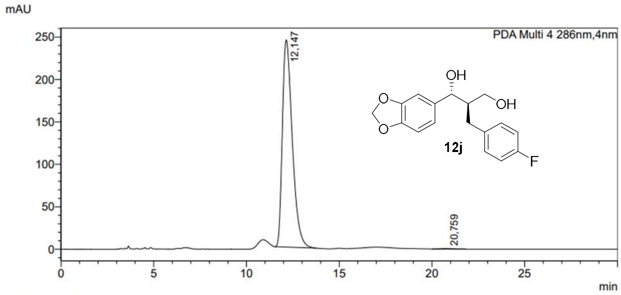

<Peak Table>

PDA Ch4 286nm

Peak#	Ret. Time	Area	Height	Area%	Height%
1	12,147	9070826	244158	99,734	99,777
2	20,759	24152	546	0,266	0,223
Total		9094978	244704	100,000	100,000

(1R,2R)-2-benzyl-1-phenylpropane-1,3-diol:

<Chromatogram>

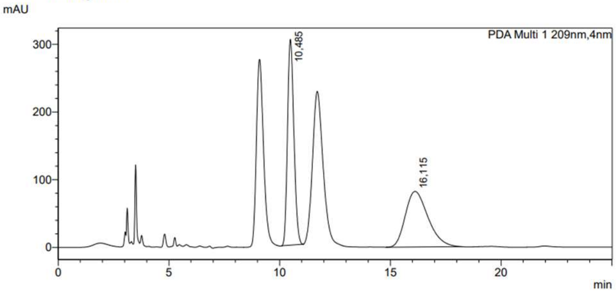

<Peak Table>

PDA Ch1 209nm

Peak#	Ret. Time	Area	Height	Area%	Height%
1	10,485	6089368	303661	50,924	78,700
2	16,115	5868430	82187	49,076	21,300
Total		11957798	385848	100,000	100,000

<Chromatogram>

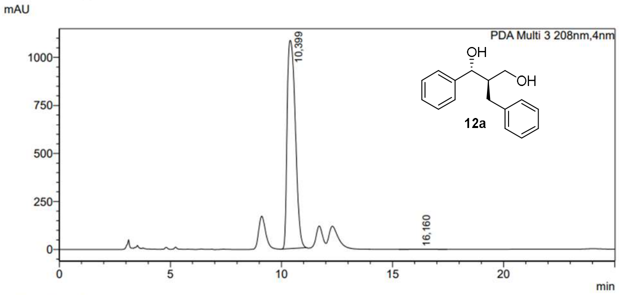

<Peak Table>

PDA Ch3 208nm

Peak#	Ret. Time	Area	Height	Area%	Height%
1	10,399	27824603	1083918	99,612	99,866
2	16,160	108336	1460	0,388	0,134
Total		27932939	1085377	100,000	100,000

(1R,2R)-2-(4-methoxybenzyl)-1-phenylpropane-1,3-diol:

<Chromatogram>

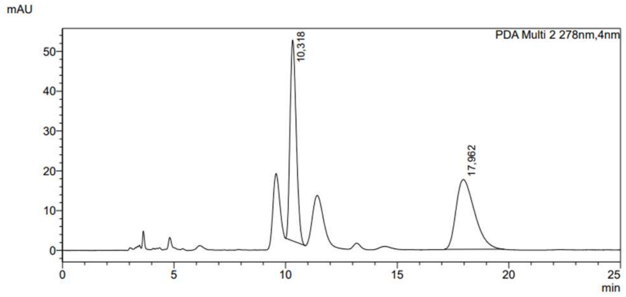

<Peak Table>

PDA Ch2 278nm

Peak#	Ret. Time	Area	Height	Area%	Height%
1	10,318	978062	50391	49,168	74,192
2	17,962	1011168	17529	50,832	25,808
Total		1989230	67920	100,000	100,000

<Chromatogram>

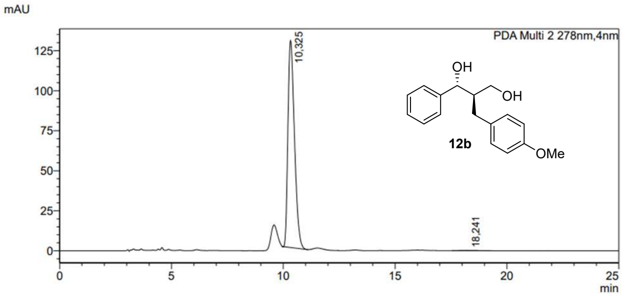

<Peak Table>

PDA Ch2 278nm

Peak#	Ret. Time	Area	Height	Area%	Height%
1	10,325	2624342	129198	99,566	99,851
2	18,241	11435	192	0,434	0,149
Total		2635777	129391	100,000	100,000

(1R,2R)-1-phenyl-2-(4-(trifluoromethyl)benzyl)propane-1,3-diol:

<Chromatogram>

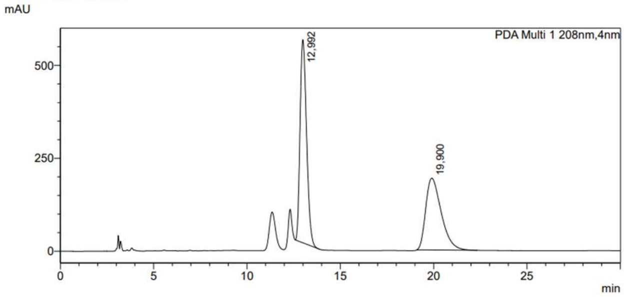

<Peak Table>

PDA Ch1 208nm

Peak#	Ret. Time	Area	Height	Area%	Height%
1	12,992	13218582	545656	54,215	73,865
2	19,900	11163286	193064	45,785	26,135
Total		24381867	738720	100,000	100,000

<Chromatogram>

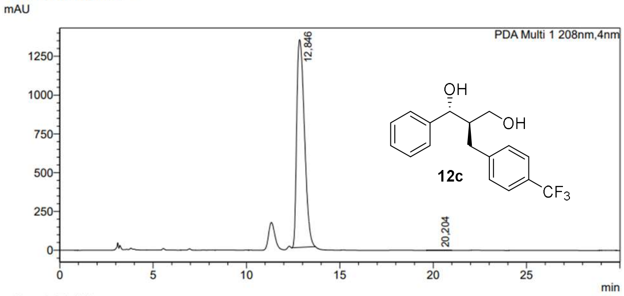

<Peak Table>

PDA Ch1 208nm

Peak#	Ret. Time	Area	Height	Area%	Height%
1	12,846	38500753	1338119	99,893	99,928
2	20,204	41266	958	0,107	0,072
Total		38542019	1339077	100,000	100,000

(1R,2R)-2-(2-fluorobenzyl)-1-phenylpropane-1,3-diol:

<Chromatogram>

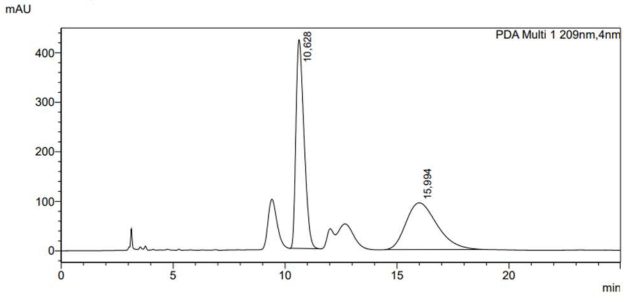

<Peak Table>

PDA Ch1 209nm

Peak#	Ret. Time	Area	Height	Area%	Height%
1	10,628	10600963	421215	53,819	81,704
2	15,994	9096467	94325	46,181	18,296
Total		19697430	515539	100,000	100,000

<Chromatogram>

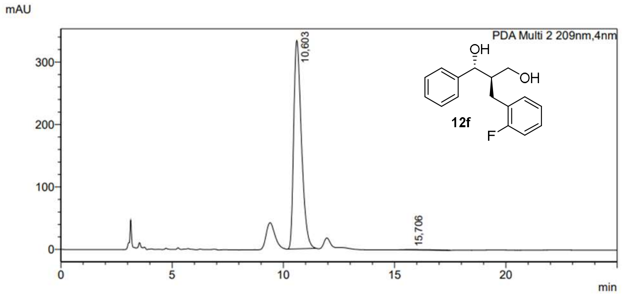

<Peak Table>

PDA Ch2 209nm

Peak#	Ret. Time	Area	Height	Area%	Height%
1	10,603	8037780	333451	99,361	99,824
2	15,706	51722	588	0,639	0,176
Total		8089502	334039	100,000	100,000

(1R,2R)-2-(3-fluorobenzyl)-1-phenylpropane-1,3-diol:

<Chromatogram>

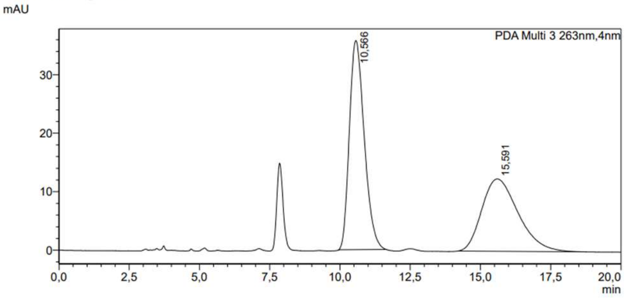

<Peak Table>

PDA Ch3 263nm

Peak#	Ret. Time	Area	Height	Area%	Height%
1	10,566	1318887	35764	54,186	74,298
2	15,591	1115094	12372	45,814	25,702
Total		2433981	48136	100,000	100,000

<Chromatogram>

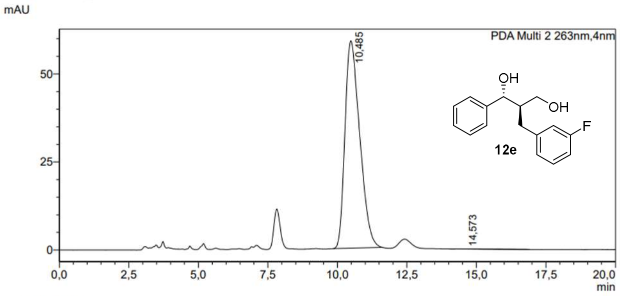

<Peak Table>

PDA Ch2 263nm

Peak#	Ret. Time	Area	Height	Area%	Height%
1	10,485	2174914	58906	99,706	99,900
2	14,573	6417	59	0,294	0,100
Total		2181332	58964	100,000	100,000

(1R,2R)-2-(4-fluorobenzyl)-1-phenylpropane-1,3-diol:

<Chromatogram>

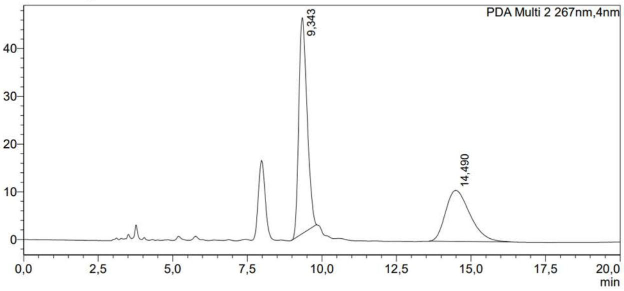

<Peak Table>

PDA Ch2 267nm

Peak#	Ret. Time	Area	Height	Area%	Height%
1	9,343	874499	45206	58,899	80,882
2	14,490	610245	10685	41,101	19,118
Total		1484744	55891	100,000	100,000

<Chromatogram>

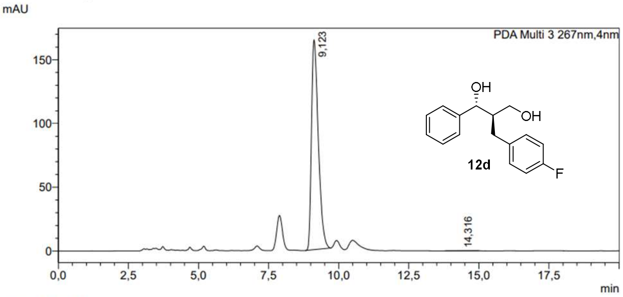

<Peak Table>

PDA Ch3 267nm

Peak#	Ret. Time	Area	Height	Conc.	Area%	Height%
1	9,123	2797082	164416	0,000	99,884	99,947
2	14,316	3259	87	0,000	0,116	0,053
Total		2800341	164503		100,000	100,000

(1R,2R)-2-benzyl-1-(4-bromophenyl)propane-1,3-diol:

<Chromatogram>

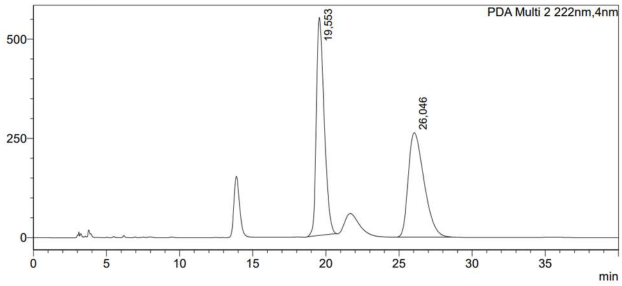

<Peak Table>

PDA Ch2 222nm

Peak#	Ret. Time	Area	Height	Area%	Height%
1	19,553	20547597	548595	50,975	67,587
2	26,046	19761532	263090	49,025	32,413
Total		40309129	811685	100,000	100,000

<Chromatogram>

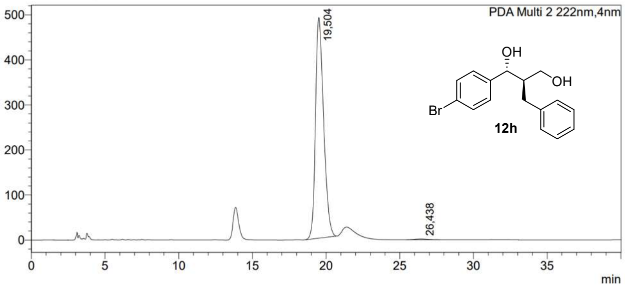

<Peak Table>

PDA Ch2 222nm

Peak#	Ret. Time	Area	Height	Area%	Height%
1	19,504	18774794	489852	99,445	99,662
2	26,438	104714	1663	0,555	0,338
Total		18879508	491515	100,000	100,000

(1R,2R)-1-(4-chlorophenyl)-2-(4-fluorobenzyl)propane-1,3-diol:

<Chromatogram>

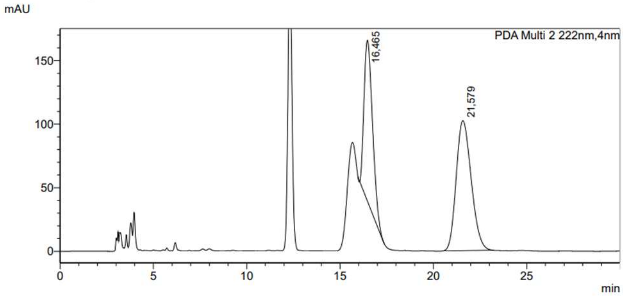

<Peak Table>

PDA Ch2 222nm

Peak#	Ret. Time	Area	Height	Area%	Height%
1	16,465	4002087	126467	41,114	55,287
2	21,579	5732140	102279	58,886	44,713
Total		9734227	228746	100,000	100,000

<Chromatogram>

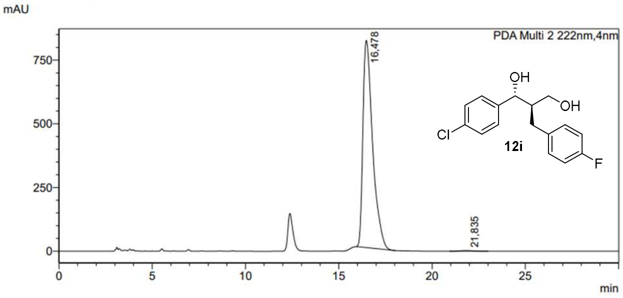

<Peak Table>

PDA Ch2 222nm

Peak#	Ret. Time	Area	Height	Area%	Height%
1	16,478	29433402	812871	99,598	99,712
2	21,835	118848	2346	0,402	0,288
Total		29552250	815218	100,000	100,000

(1R,2R)-2-benzyl-1-(4-methoxyphenyl)propane-1,3-diol:

<Chromatogram>

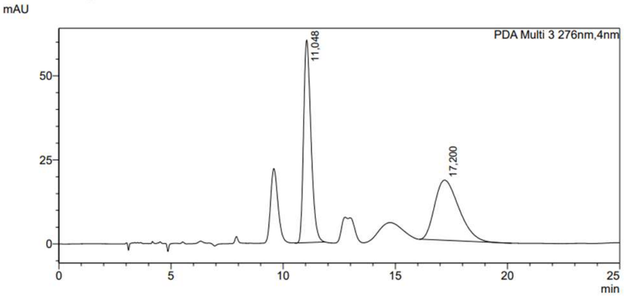

<Peak Table>

PDA Ch3 276nm

Peak#	Ret. Time	Area	Height	Area%	Height%
1	11,048	1376912	60197	50,598	77,090
2	17,200	1344359	17890	49,402	22,910
Total		2721271	78087	100,000	100,000

<Chromatogram>

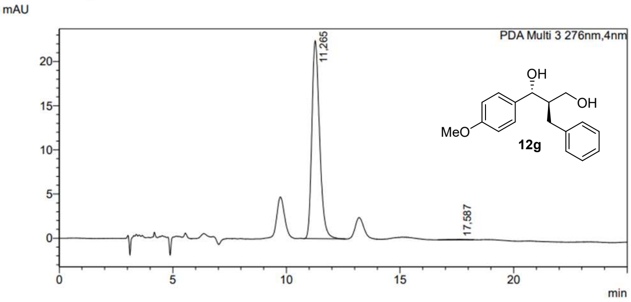

<Peak Table>

PDA Ch3 276nm

Peak#	Ret. Time	Area	Height	Area%	Height%
1	11,265	532119	22387	99,618	99,782
2	17,587	2040	49	0,382	0,218
Total		534159	22436	100,000	100,000

(2R,3R)-3-(benzo[d][1,3]dioxol-5-yl)-2-(4-fluorobenzyl)-3-hydroxypropyl 4-methylbenzenesulfonate:

<Chromatogram>

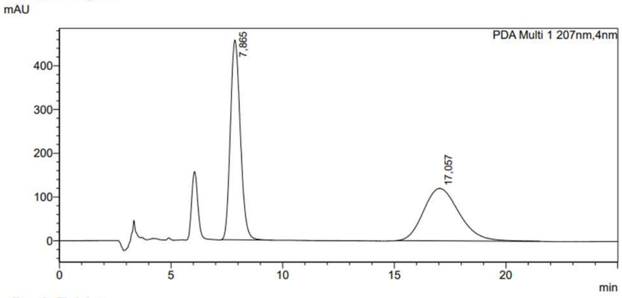

<Peak Table>

PDA Ch1 207nm

Peak#	Ret. Time	Area	Height	Area%	Height%
1	7,865	14159305	456443	51,519	79,208
2	17,057	13324512	119816	48,481	20,792
Total		27483817	576259	100,000	100,000

<Chromatogram>

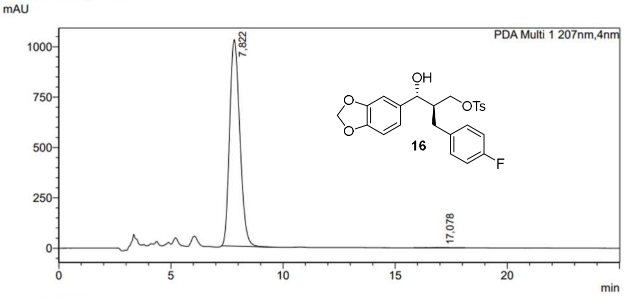

<Peak Table>

PDA Ch1 207nm

Peak#	Ret. Time	Area	Height	Area%	Height%
1	7,822	32516547	1024387	99,906	99,944
2	17,078	30461	571	0,094	0,056
Total		32547008	1024958	100,000	100,000

5-((2R,3R)-3-(4-fluorobenzyl)oxetan-2-yl)benzo[d][1,3]dioxole:

<Chromatogram>

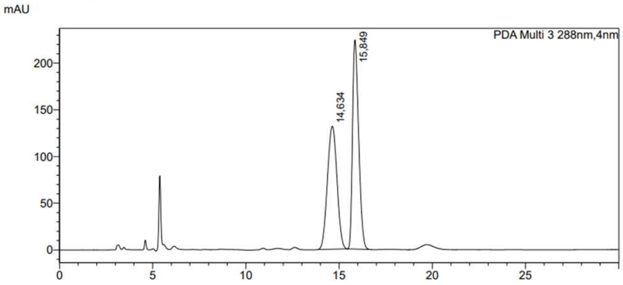

<Peak Table>

PDA Ch3 288nm

Peak#	Ret. Time	Area	Height	Area%	Height%
1	14,634	4695548	131790	47,765	37,063
2	15,849	5135023	223790	52,235	62,937
Total		9830571	355580	100,000	100,000

<Chromatogram>

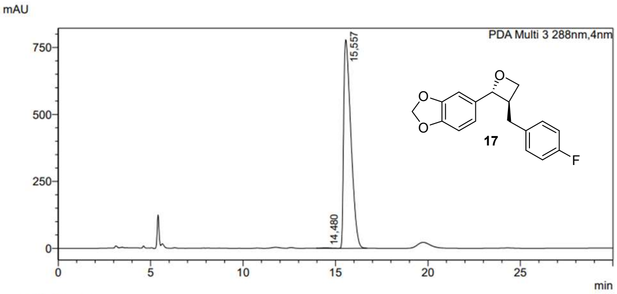

<Peak Table>

PDA Ch3 288nm

Peak#	Ret. Time	Area	Height	Area%	Height%
1	14,480	41227	1366	0,194	0,175
2	15,557	21218638	777977	99,806	99,825
Total		21259865	779342	100,000	100,000
